# First chromosome-level genome assembly of the colonial chordate model *Botryllus schlosseri* (Tunicata)

**DOI:** 10.1093/gigascience/giaf097

**Published:** 2025-09-18

**Authors:** Olivier De Thier, Marie Lebel, Mohammed M.Tawfeeq, Roland Faure, Philippe Dru, Simon Blanchoud, Alexandre Alié, Federico D Brown, Jean-François Flot, Stefano Tiozzo

**Affiliations:** Evolutionary Biology & Ecology, C.P. 160/12, Université libre de Bruxelles (ULB), Avenue F.D. Roosevelt 50, B-1050 Brussels, Belgium; Interuniversity Institute of Bioinformatics in Brussels – (IB)^2^, B-1050 Brussels, Belgium; CNRS, Sorbonne Université, Laboratoire de Biologie du Développement de Villefranche Sur-mer (LBDV - UMR7009), IMEV - 181 Chemin du Lazaret, F-06230 Villefranche-sur-Mer, France; Evolutionary Biology & Ecology, C.P. 160/12, Université libre de Bruxelles (ULB), Avenue F.D. Roosevelt 50, B-1050 Brussels, Belgium; Interuniversity Institute of Bioinformatics in Brussels – (IB)^2^, B-1050 Brussels, Belgium; Evolutionary Biology & Ecology, C.P. 160/12, Université libre de Bruxelles (ULB), Avenue F.D. Roosevelt 50, B-1050 Brussels, Belgium; Interuniversity Institute of Bioinformatics in Brussels – (IB)^2^, B-1050 Brussels, Belgium; CNRS, Sorbonne Université, Laboratoire de Biologie du Développement de Villefranche Sur-mer (LBDV - UMR7009), IMEV - 181 Chemin du Lazaret, F-06230 Villefranche-sur-Mer, France; Department of Biology, University of Fribourg, CH-1700 Fribourg, Switzerland; CNRS, Sorbonne Université, Laboratoire de Biologie du Développement de Villefranche Sur-mer (LBDV - UMR7009), IMEV - 181 Chemin du Lazaret, F-06230 Villefranche-sur-Mer, France; Departmento de Zoologia, Instituto de Biociências, Universidade de São Paulo, São Paulo - SP 05508-090, Brazil; Evolutionary Biology & Ecology, C.P. 160/12, Université libre de Bruxelles (ULB), Avenue F.D. Roosevelt 50, B-1050 Brussels, Belgium; Interuniversity Institute of Bioinformatics in Brussels – (IB)^2^, B-1050 Brussels, Belgium; CNRS, Sorbonne Université, Laboratoire de Biologie du Développement de Villefranche Sur-mer (LBDV - UMR7009), IMEV - 181 Chemin du Lazaret, F-06230 Villefranche-sur-Mer, France

**Keywords:** budding, regeneration, chimerism, ascidian, coloniality, model organism

## Abstract

**Background:**

*Botryllus schlosseri* (Tunicata) is a colonial, laboratory model tunicate recognized for its remarkable developmental diversity, its regenerative abilities, and its peculiar genetically determined allorecognition system governed by a polymorphic locus controlling chimerism and cell parasitism.

**Results:**

We report the first chromosome-level genome assembly of *B. schlosseri* subclade A1. By integrating long and short reads with Hi-C scaffolding, we produced both a phased diploid genome assembly and a conventional collapsed consensus sequence of 533 Mb. Of this total length, 96% belonged to 16 chromosome-scale scaffolds, with a BUSCO completeness score of 91.4%. We then compared our assembly with other high-quality tunicate genomes, revealing some synteny conservation but also extensive genomic rearrangements and a general loss of colinearity.

**Conclusions:**

The chromosome-level resolution of this assembly enhances our understanding of genome organization in colonial modular organisms. Comparative analyses highlight the dynamic nature of tunicate genomes, with conserved macrosynteny yet extensive microsyntenic rearrangements and scrambling, underscoring their rapid evolutionary trajectory. This high-quality genome assembly provides a valuable resource for exploring the unique biological features of colonial chordates, including their exceptional regenerative abilities and complex allorecognition system.

## Introduction

Each member of the colony is an individual animal, but the colony is another individual animal, not like the sum of its individuals [...]. So a man of individualistic reason, if he must ask, “Which is the animal?” must abandon his particular kind of reason and say, “Why, it’s two animals and they aren’t alike any more than the cells of my body are like me. I am much more than the sum of my cells, and, for all I know, they are much more than the division of me.”

—John Steinbeck, *The Log from the Sea of Cortez*

**Figure 1:**
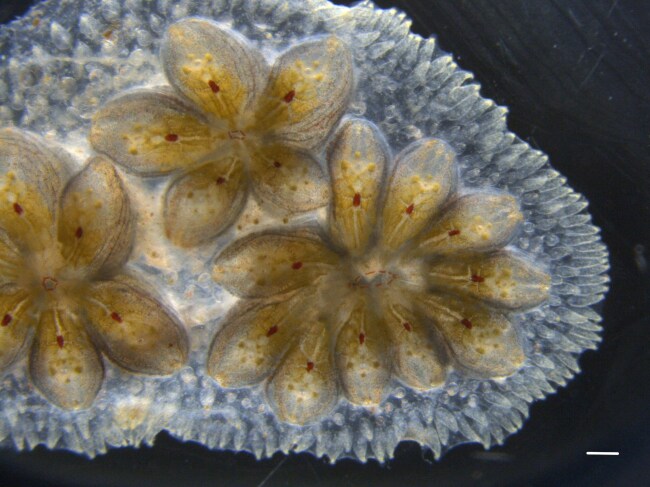
Colony of *Botryllus schlosseri* (photograph by Stefano Tiozzo). Scale bar: 1 mm.

In the subphylum Tunicata, the sister group of vertebrates [1], colonial species reproduce both sexually and asexually through various forms of budding. Through budding, new functional bodies emerge from adult somatic cells and tissues. Regardless of variations in budding modes among tunicate species [2] and of whether development occurs through asexual budding or sexually via embryogenesis, the basic body plan of adult tunicates is broadly conserved across the entire subphylum [3]. In colonial tunicates, asexually generated individuals generally remain physically connected, forming colonies. Colony formation, clonal reproduction, and modular organization have important physiological, ecological, and evolutionary implications. For example, modular organization supports rapid growth on hard, space-limited substrates, outperforming solitary forms. Morphological plasticity enables colony-level adaptation to predation, damage, or environmental changes. Furthermore, uniparental reproduction, including budding, likely provides a selective advantage for rapid colonization on invasion fronts or in disturbed habitats (reviewed in [4]). Like many other colonial tunicates, *Botryllus schlosseri* (Pallas, 1766) (NCBI:txid30301) can generate a functional adult body via 3 distinct developmental pathways. The first one involves sexual reproduction, where the fertilized egg passes through a larval stage and develops into an initial colony founder. The second pathway is asexual propagation, where the founder zooid continuously reproduces through palleal (aka peribranchial) budding, forming a colony of hundreds of zooids connected by the vascular system (a network of extracorporeal vessels within a cellulose-based extracellular matrix, the so-called tunic [5]; Fig. 1). Lastly, if all zooids and buds are removed from a *B. schlosseri* colony, new buds can regenerate from the vascular system in a process known as vascular budding, allowing asexual propagation and eventual colony reformation [6–8]. Zooids within a single colony are genetically identical clones. However, wild colonies often come into contact and fuse, resulting in chimeras where circulating cells carry different genotypes. These mixed pools of circulating cells contribute to sexual and, according to some authors, asexual and regenerative development [9–11]. During chimerism, donor cells may entirely replace the host’s germline or somatic cells, a phenomenon termed germ cell or somatic cell parasitism, respectively [10, 12, 13]. As a result, zooids within a chimeric colony are not always clonemates.

*Botryllus schlosseri* was introduced to laboratories over half a century ago [14] as a model to study asexual development, regeneration [15], allorecognition, and chimerism [16, 17]. Over recent decades, a dedicated scientific community has emerged, advancing breeding techniques and developing imaging and molecular biology tools to better study this species [8, 9, 18–21]. Several anatomical descriptions and staging methods have been proposed [5, 22], and extensive transcriptomic databases for various developmental stages and tissues have been generated [8, 23–27]. In 2013, a draft genome of *B. schlosseri* was published [28], but it lacked the completeness and continuity required by today’s assembly standards [29]. In this study, we present a high-quality, chromosome-level collapsed assembly as well as a chromosome-scale haplotype-resolved assembly for *B. schlosseri*. This new resource offers a robust platform for investigating the developmental and regenerative processes, complex allorecognition, chimerism, and cell parasitism of this colonial chordate.

## Results and discussion

### Sequencing and genome size estimation

Genomic DNA was extracted from a laboratory-reared colony, referred to as clone E*, derived from a single zygote and therefore nonchimeric. Sequencing libraries from clone E* yielded 489 million Illumina (short) paired-end 150-bp reads, 2.4 million PacBio HiFi (long) reads with an N50 length of $\sim$9.5 kb (max length of $\sim$50 kb), and 10.9 million ONT (long) reads with an N50 length of $\sim$10.3 kb (max length of $\sim$205 kb) (Table 1).

**Table 1:** Sequencing technologies used to sequence *B. schlosseri*’s genome (clone E*), and related read statistics

Technology	Total size (Gbp)	Number of reads	N50 (bp)	Coverage
Illumina	73.2	488,906,094	150	146
Illumina Hi-C	15.9	106,488,252	150	32
PacBio HiFi (round 1)	7.9	1,170,137	8,711	16
PacBio HiFi (round 2)	10.8	1,218,052	10,151	22
ONT (R9.4.1)	58.9	10,888,103	10,320	118

Based on *k*-mer analyses, the genome size was estimated to be around 500 Mbp with a heterozygosity of 3.63% (Supplementary Fig. S1), whereas Feulgen densitometry (a histochemical approach) yielded an estimate of $\sim$492 Mbp (using 1 pg = 978 Mbp; Supplementary Fig. S5). Both genome size estimates were concordant but notably smaller than a previous cytofluorimetry-based estimation of 725 Mb [30] and than the first genome assembly obtained by Voskoboynik et al. [28], which had a size of 580 Mbp.

**Figure 2:**
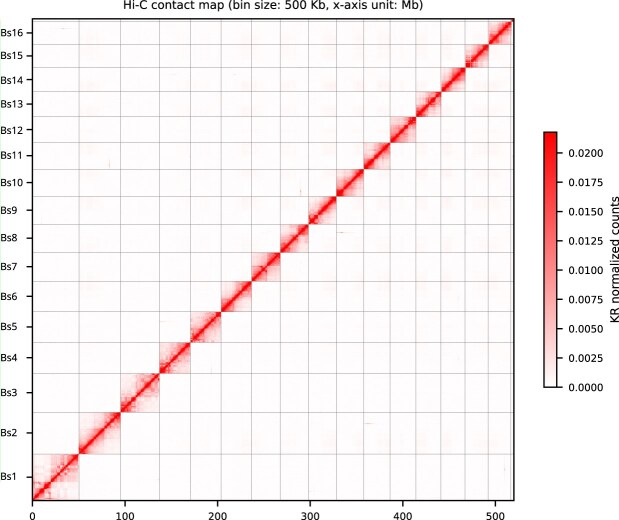
Hi-C heatmap of the collapsed assembly of the *Botryllus schlosseri* genome showing 16 chromosome-scale scaffolds. The figure was generated using the visualization module of HapHiC [36].

**Figure 3:**
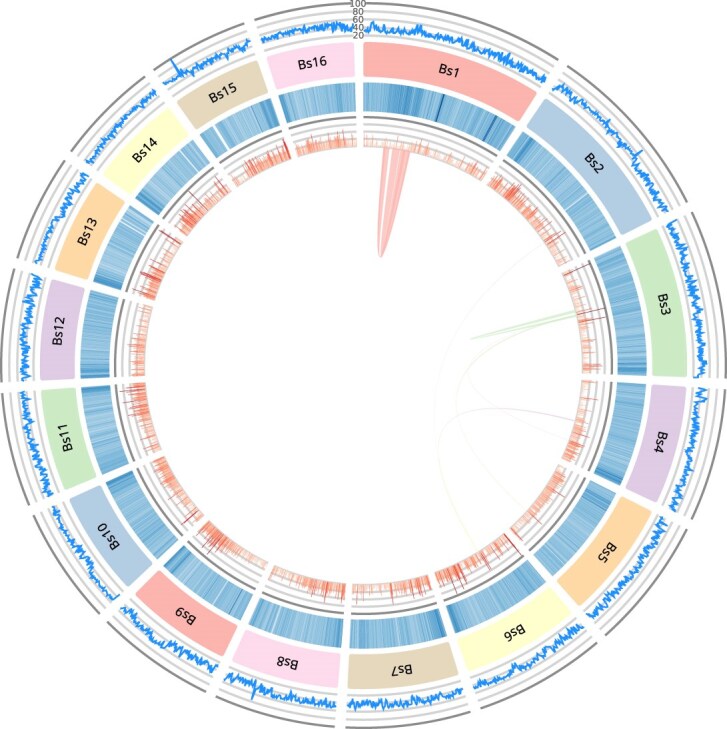
Circos plot of the distribution of several genomic characteristics along the 16 longest scaffolds (labeled Bs1 to Bs16) of the collapsed assembly (made using AccuSyn [37]). Each layer of the circle represents, from the inside to the outside, the synteny blocks detected by MCScanX [38], histograms of gene density, heatmaps of the presence of repetitive elements, the scaffold names in clockwise order, and the sequencing depth of HiFi reads.

**Figure 4:**
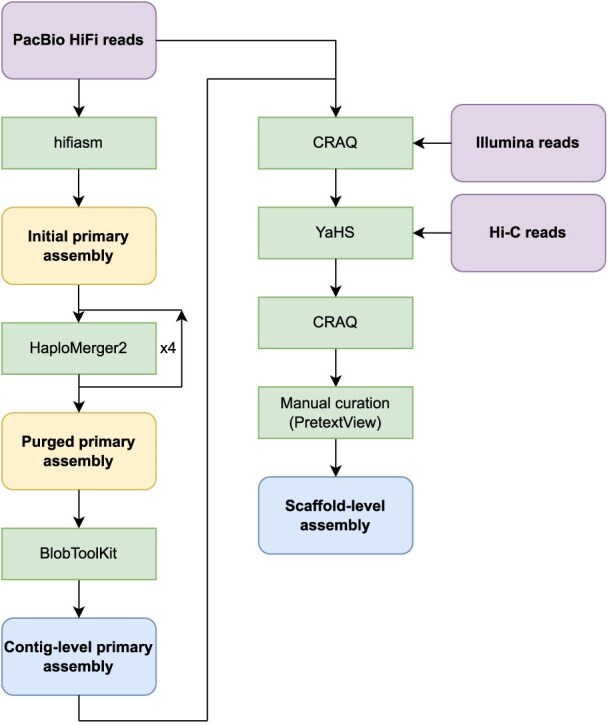
Assembly pipeline for the collapsed genome assembly (see Methods).

An initial collapsed genome assembly was obtained using hifiasm [31] (RRID:SCR_021069); it had a size of 570 Mbp and comprised 930 contigs with an N50 length of 4.9 Mbp. In this assembly, BlobToolKit (RRID:SCR_023351) identified 452 contigs (totaling 37 Mbp) as putative contamination and mitochondrial sequences (see next section), which were subsequently removed. Of these 37 Mbp, approximately half were attributed to members of the bacterial phylum Pseudomonadota (Supplementary Fig. S6). We identified 28 contigs that belonged to spore-forming unicellular parasites of the microsporidia group [32]. To our knowledge, this represents the first report of this fungal group in a tunicate species. However, we cannot rule out the possibility that these sequences may have been assigned incorrectly or originate from contaminants present in the water rather than from parasitized *Botryllus* tissues. The remaining contigs were corrected using CRAQ [33], which detects and breaks misassembled contigs; this raised the total number of contigs in the assembly from 478 to 516. We then performed Hi-C scaffolding using YaHS [34] (RRID:SCR_022965), which reduced the number of sequences to 256, before running CRAQ again on the scaffolded assembly: this time, 4 misassembled contigs were detected and broken. Finally, a manual curation was performed, resulting in an assembly made up of 16 major scaffolds, labeled Bs1 to Bs16, containing around 96% (513 Mbp) of the total sequence length (533 Mbp) (Table 2, Supplementary Table S3, Figs. 2 and 3). The number and relative lengths of these 16 major scaffolds were consistent with the published karyogram of *B. schlosseri* [35], with the exception of Bs16, which was notably longer in our assembly (Supplementary Fig. S14). The full assembly pipeline is summarized in Fig. 4 and detailed in the Methods section.

**Table 2:** Assembly statistics for all the scaffolds and for the 16 longest ones

Measure	All scaffolds	16 longest scaffolds
Length (Mbp)	533	513
No. of sequences	254	16
N50 (Mbp)	30	31
GC (%)	40.52	40.46
No. of annotated genes	22,275	21,677
BUSCO Complete	91.6%	91.4%
(Single, Duplicated)	(90.7%, 0.9%)	(90.7%, 0.7%)
BUSCO Fragmented	3.1%	3.1%
BUSCO Missing	5.3%	5.5%

**Figure 5:**
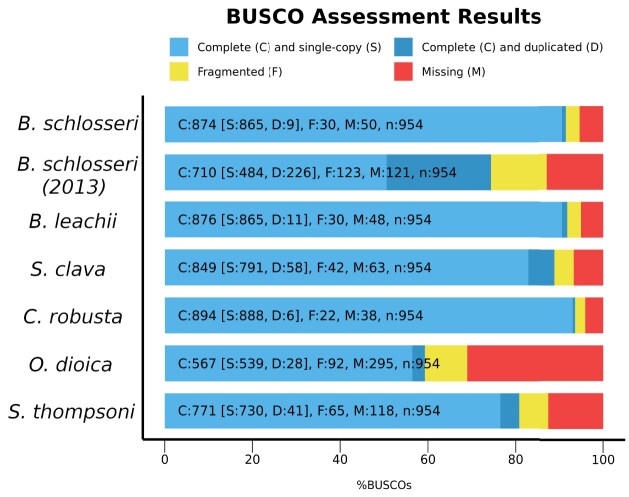
Orthology assignment in previous tunicate genome projects. Proportion of BUSCO genes detected or missed in the new genome assembly of *B. schlosseri* compared to the previous assembly (*B. schlosseri* [2013] [28]) and other reference genomes.

The completeness of our assembly was assessed using the BUSCO tool [39] (RRID:SCR_015008, v5.4.4) with the metazoa_odb10 dataset, which returned a genome completeness of 91.6% (including 0.9% of duplicated marker genes), compared to 74.4% (including 23.7% of duplicated marker genes) for the assembly by Voskoboynik et al. [28] (Fig. 5). The high duplication score of the previously available assembly indicates that its larger size (580 Mbp vs. 533 Mbp) was caused by incompletely collapsed haplotypes [40]. Synteny analysis performed using MCScanX [38] (RRID:SCR_022067) highlighted the presence of 2 large-scale genomic palindromes located within Bs1 and a smaller one in Bs3 (displayed in red and green in the innermost layer of Fig. 3). To find out whether these palindromes may have resulted from assembly artifacts caused by uncollapsed haplotypes [41], we checked the sequencing depth profiles across these regions (Supplementary Figs. S11–S13), as well as the localization of the duplicated BUSCO genes along the chromosomes, and did another run of CRAQ, this time using ONT as long reads (with higher coverage compared with the HiFi reads used in the previous rounds). There was no significant difference in the number of duplicated BUSCO genes within Bs1 and Bs3 compared to other genomic regions, and CRAQ did not detect structural errors in these scaffolds either. This suggests that the palindromes observed are real, with potential biological significance that will require further investigation.

### Molecular identification as subclade A1

*B. schlosseri* is considered a species complex comprising 5 genetically distinct clades (A to E), each representing a cryptic species with its own characteristic geographic distribution [42, 43]. Detailed analysis of cytochrome *c* oxidase subunit I (COI) mitochondrial sequences divides clade A into 3 distinct subclades: A1, A2, and A3 [44]. The complete mitochondrial DNA of clone E* was recovered and assembled as a single circular contig. Our mitogenome assembly shares 99.95% identity with the published mitochondrial sequence assigned to the *B. schlosseri* subclade A1 [44]. Notably, this subclade includes the sc6ab specimen used by Voskoboynik et al. [28] to generate the previous reference assembly of *B. schlosseri*. Our mitogenome assembly further shares 99.7% nucleotide identity with that reference sequence. Phylogenetic analyses based on a COI fragment used as DNA barcode for ascidians ([44]) confirmed that sample E* belongs to subclade A1 (Supplementary Fig. S7), a group that is both widely distributed and employed as a laboratory model worldwide.

### Structural and functional annotation

Using a *de novo* repeat library created by RepeatModeler (RRID:SCR_015027), RepeatMasker (RRID:SCR_012954) detected that around 63% of the novel *B. schlosseri* collapsed genome assembly consists of repetitive elements, which is close to the 65% of repeats found in the previously published assembly [28]. Most of these were interspersed repeats (see Table 3). A relatively high abundance of repetitive sequence was also reported in other colonial tunicates. For instance, *Salpa thompsoni* and *Salpa aspera*, both colonial species, possess a larger genome (742 Mb and 901 Mb, respectively) and an higher repeat content (ca. 80%) compared to solitary tunicates such as *Ciona robusta* (ca. 160 Mb, about 20–25% repeats) or *Oikopleura dioica*, which has a compact genome of 70 Mb with only ca. 15% repetitive content. This pattern suggests that colonial tunicates exhibit a greater genomic expansion and a larger repeat content than their solitary counterparts. Yet, the colonial *Botrylloides diegensis*, which carries a relatively small genome [45], and the solitary *S. clava*, with 46.6% repetitive elements, represent notable exceptions. Additional high-quality genome assemblies across a broader range of tunicate species will be essential to confidently assess the possible association between coloniality and repeat content [46–48].

**Table 3:** Classes of repeats in the *Botryllus schlosseri* genome. RepeatMasker summary table for the collapsed genome assembly of *Botryllus schlosseri* showing the percentages of identified repeat classes.

Repeat class	Percentage of genome
**Long Interspersed Nuclear Elements (LINEs)**	**4.52%**
LINE1	0.15%
LINE2	2.06%
**Long Terminal Repeats (LTRs)**	**1.34%**
**DNA elements**	**7.24%**
hAT-Charlie	2.96%
TcMar-Tigger	0.01%
**Unclassified**	**46.03%**
**Total interspersed repeats**	**59.12%**
**Simple repeats**	**3.94%**
**Low complexity**	**0.02%**
**Total**	**63.09%**

*Ab initio* genome annotation using the BRAKER3 pipeline [49] (RRID:SCR_018964) initially predicted 16,966 coding genes, after which refinement using the PASA pipeline [50, 51] (RRID:SCR_014656) finally retrieved 22,275 genes coding for 30,813 proteins (see Table 4). This number is significantly lower than originally predicted for *B. schlosseri* (38,730 predicted genes [28]), probably due to the incomplete collapse of the previous assembly. In terms of completeness of the annotation, BUSCO retrieved 92.4% complete (79.7% single, 12.7% duplicated) and 1.8% fragmented metazoan genes when given all predicted isoforms, whereas it retrieved 92% complete (91% single, 0.9% duplicated) and 1.8% fragmented metazoan marker genes when filtered to only keep the longest isoform. Running BUSCO directly on the scaffold sequences yielded similar results (data not shown).

**Table 4:** Gene predictions and annotation statistics

Type	Number	Mean size (bp)	% genome
Gene	22,275	8,566.13	35.78
mRNA	30,813	10,576.62	N/A
CDS	237,200	199.16	8.86
Exon	241,815	289.83	13.14
5′ UTR	21,386	432.29	1.73
3′ UTR	20,985	648.00	2.55
Total	574,474	1,143.44	N/A

**Figure 6:**
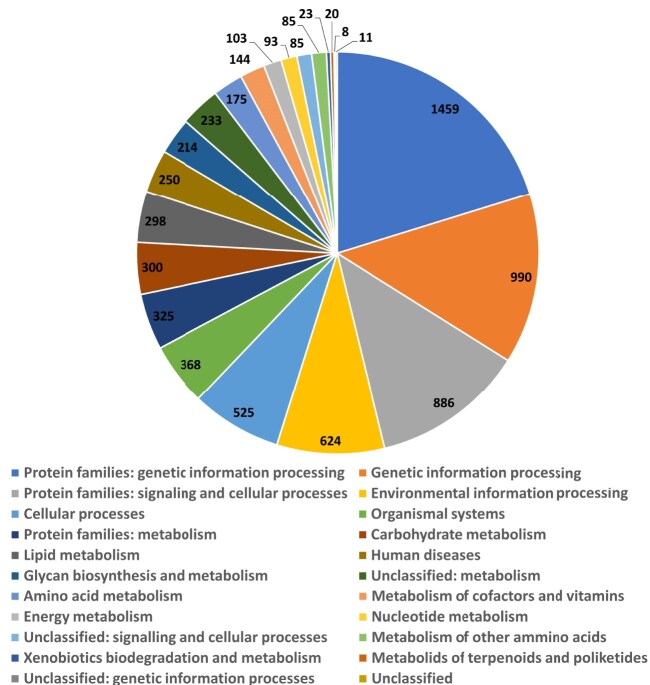
Pie chart of the assignation of the annotated genes of *Botryllus schlosseri* to KEGG functional categories using BlastKOALA [55].

The functional annotation and orthology assignment [52], coupled with annotation of protein domains, motifs, and functional sites [53, 54], were written into gff3 and Genbank files. KEGG route-mapping assigned 7,221 genes over the annotated entries and distributed them across 21 KEGG categories (Fig. 6). Among them, the most prevalent ones include KEGG hierarchies dealing with genetic information processing (2,449/7,219, 22.92%), such as DNA replication, repair, recombination, transcription, translation, and regulation of gene expression; signaling and cellular processes (886/7,219, 12.27%); and environmental information processing (674/7,219, 8.64%), such as various cellular processes and signaling pathways involved in sensing, transducing (i.e., MAPK signaling, PI3K-Akt signaling, and cAMP signaling), responses to external signals (i.e., G-protein coupled receptors, receptor tyrosine kinases, and cytokine receptors), intracellular communication, and cell motility. The KEGG annotations provided for *B. schlosseri* are consistent and coherent with the functional annotation of the published complete genomes of other ascidian tunicates, such as *Styela clava, Ciona robusta*, and *Oikopleura dioica* (Supplementary Fig. S8).

### Haplotype-resolved assembly

Given its heterozygosity level exceeding 3%, haplotype-resolved assemblies of *B. schlosseri* are crucial for studying differences between homologous chromosomes, such as structural variations. Using hifiasm with direct integration of Hi-C reads and subsequent scaffolding (Supplementary Fig. S9), we generated a pair of chromosome-scale, haplotype-resolved assemblies (haplotype 1 and haplotype 2), each organized into 16 major scaffolds (see Supplementary Fig. S10). With respective sizes of 496 Mbp and 494 Mbp, these assemblies are smaller than the collapsed assembly (533 Mbp). When considering only the 16 longest scaffolds, the sizes decrease to 480 Mbp for haplotype 1 and 464 Mbp for haplotype 2, compared to 513 Mbp for the collapsed assembly. Additionally, their BUSCO completeness scores are lower, with values of 90.9% and 91.2%, respectively, compared to 91.6% for the collapsed assembly. This is further reflected in their annotation results, where fewer genes were identified: 21,802 and 21,831 for haplotype 1 and haplotype 2, respectively, versus 22,275 for the collapsed assembly (see Supplementary Table S1). The observed differences in metrics, where the results for the haplotype-resolved assemblies are inferior to those for the collapsed assembly, may be attributed to misassemblies, particularly deletions. For example, when comparing the putative chromosome lengths (see Supplementary Table S2) for chromosomes 1 and 3, we observe a significant disparity in sizes between the 2 haplotypes, which may be attributed to incomplete sequence reconstructions during the assembly process. Such anomalies may additionally be observed when comparing the putative chromosome lengths of all assemblies with the karyogram of *B. schlosseri*, as described by Colombera [35] (see Supplementary Fig. S14). Notably, the sizes of the collapsed assembly appear to more closely match the expected distribution compared to the phased haplotypes. Furthermore, multiple structural variations between the 2 haplotypes, particularly small inversions (see Supplementary Figs. S15 and S16), seem to be present in the majority of the homologous chromosomes. However, as with the observed putative deletions, these may result from misassemblies and require further validation to enhance the quality of the haplotype-resolved assembly.

### Synteny analyses

**Figure 7:**
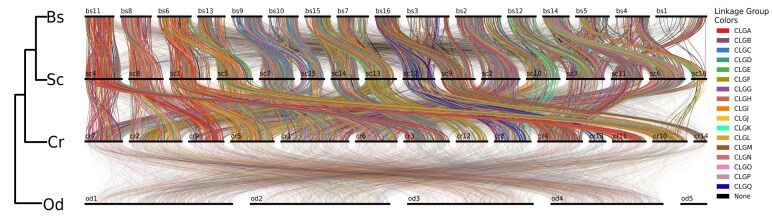
Synteny analyses using CLGs between *Botryllus schlosseri* (Bs), *Styela clava* (Sc), *Ciona robusta* (Cr), and *Oikopleura dioica* (Od). For each species, the horizontal black lines represent the chromosomes, while the colored vertical lines connect conserved orthologs between species pairs. Each color corresponds to one of the 17 ancestral CLGs identified in [58]. The opacity of the lines indicates the significance of the interaction between interspecies chromosomes, with solid colors representing significantly enriched conservation of synteny.

To assess macrosynteny conservation between *B. schlosseri* and other tunicates, we selected genomes that met 2 specific criteria: they were assembled at the chromosome level, ensuring comparable high-quality structural information, and they represented, as much as possible, the breadth of diversity within the tunicate subphylum. *S. clava* [56] belongs to the same order as *Botryllus* (Stolidobranchia), *C. robusta* [46] to a different order (Phlebobranchia), and *O. dioica* [47] to a different class of tunicates (Appendicularia) [57]. We used 17 groups of orthologous genes identified by Simakov et al. [58] as ancestral chordate linkage groups (CLGs). These groups of genes are thought to have remained physically linked since the divergence of the Olfactores lineage (which includes both vertebrates and tunicates) from cephalochordates. However, Oxford dot plots [59] revealed a general loss of syntenic equivalence [60] among tunicate genomes, even between *B. schlosseri* and *S. clava*, which share the same haploid chromosome number of 16. Despite this identical number of chromosomes, the comparison between the 2 stolidobranchs showed extensive chromosome rearrangements, including fissions and fusions with mixing [60, 61] (Fig. 7 and Supplementary Fig. S17). These rearrangements are even more pronounced in *C. robusta*, which has a haploid chromosome number of 14. The overall random distribution of ortholog pairs within blocks points to significant order scrambling, resulting in a loss of colinearity (i.e., the sequential order of genes along the same chromosome); the comparison with *O. dioica* shows a complete breakdown of both macrosynteny and colinearity, with CLGs fully scrambled and dispersed. The latter result is consistent with the very long and fast-evolving branch of Appendicularia compared to other tunicates [57], as well as with the extreme genome scrambling rate of Appendicularia compared to other tunicates and mammals [62]. The same analyses using a set of 29 linkage groups generally conserved among bilaterians, cnidarians, and sponges [60] yielded similar results (Supplementary Fig. S18). The extensive physical linkage of groups of orthologous genes has been shown to be conserved across highly divergent bilaterian phyla, including Chordata, Echinodermata, Mollusca, and Nemertea [60, 61]. Notably, our preliminary synteny analyses across 4 tunicate species reveal a highly dynamic genomic landscape, where syntenic equivalence, defined as one-to-one chromosomal correspondence regardless of gene order, is largely disrupted, even among species within the same family. Frequent chromosomal fission and fusion events further underscore the rapid evolutionary turnover of tunicate genomes. The increasing erosion of macrosynteny with phylogenetic distance suggests that patterns of conserved chromosomal linkage could serve as informative characters for phylogenetic inference. Interestingly, a similar pattern of genome rearrangements was recently reported in Bryozoa [61] and in clitellate annelids [63–65], pointing to a potential parallel and independent loss of the ancestral bilaterian genome architecture in these lineages and in tunicates. These observations raise compelling questions about the underlying mechanisms driving such rearrangements, which may reflect a relaxation of the selective constraints typically maintaining gene order in other metazoan groups [66].

### Hox gene analyses

**Figure 8:**
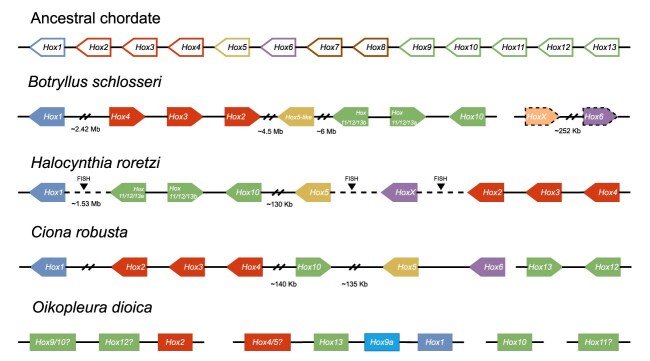
Representation of the Hox genes retrieved in the new assembly of *B. schlosseri* compared to the supposed original single Hox cluster of the chordate ancestor and other tunicates. Linked genes (present on the same scaffold) are connected by a solid line, while a dashed line is used when the linkage has been deduced using another method. When known, the transcription orientation is indicated by an arrow-shaped rectangle, which is surrounded by a dashed line when the Hox gene was retrieved with low confidence.

Hox genes are a subset of homeobox genes that play important developmental roles in the specification of body segments along the anterior-posterior axis. Their arrangement into a syntenic cluster colinear with gene expression is conserved across Bilateria, with some exceptions [67]. In the new collapsed assembly, we retrieved 10 *B. schlosseri* Hox genes, which is consistent with draft genomes of other ascidian tunicates [68]. Orthology of *B. schlosseri* Hox genes was assessed using phylogenetic analyses, as in Sekigami et al. [69], based on Hox tree topology among the tunicates *C. robusta* and *Halocynthia roretzi*, the cephalochordate *Branchiostoma lanceolatum*, and 3 vertebrate species. The names of the *B. schlosseri* Hox genes were assigned based on their proximity to the ones of *C. robusta* (Supplementary Figs. S19 and S20). However, most branches had low bootstrap support, and therefore including more tunicates as well as vertebrate species will be necessary to resolve the complex evolution of the Hox gene cluster across tunicates [68]. Although Hox genes are colinear between cephalochordates and vertebrates, it is not the case for tunicates [70]. In the tunicate species studied thus far, Hox clusters exhibit divergences in terms of colinearity and synteny relative to the ancestral chordate cluster [68]. In contrast to previous data [28, 45], our new assembly revealed that *B. schlosseri*’s Hox genes are less scattered than previously described, suggesting improved contiguity in the new genome assembly. Eight of them are grouped on the second largest scaffold (Bs2), yet for some of them at a relatively large distance, whereas 2 other ones are found on the 15th largest scaffold (Bs15) (Fig. 8). Comparison with 2 tunicate ascidians, belonging to the same (*H. roretzi* [69]) and a different (*C. robusta* [46]) order, revealed partially conserved synteny as well as inversions and transpositions across the 3 species (Fig. 8). These observations agree with the general trend of synteny conservation despite loss of colinearity observed for CLGs [58] and are also consistent with the phylogenetic relationships among the species sequenced [2, 57]. Yet, the limited availability of chromosome-level genome assemblies continues to hinder a clear picture of the evolutionary dynamics of the Hox clusters across tunicates. Altogether, these findings show that *B. schlosseri* follows the general tunicate trend of dispersed and rearranged Hox clusters, but with a more clustered configuration than previously thought. This could reflect lineage-specific retention of partial clustering and provides a more refined view of the dynamic genomic architecture in tunicates. While colinearity was clearly lost, partial synteny and clustering remain, offering a potential window into the mechanisms and consequences of Hox cluster disintegration during chordate evolution.

## Conclusion

Tunicate genomes are known for their rapid evolution, featuring high rates of molecular divergence and extensive genomic rearrangements, and they are generally remarkably compact compared to vertebrates, though genome size varies among tunicate species [71]. Additionally, while some tunicates exhibit high levels of repetitive elements, others show moderate repeat content [45, 66]. Despite these variations, tunicate genomes share conserved noncoding elements, reflecting deep regulatory constraints within this diverse subphylum [72]. Although solitary tunicates such as *Ciona* and *Oikopleura*, along with other species, have been instrumental in shaping our understanding of tunicate genomes, colonial tunicates remain relatively understudied at the genomic level. Colonial species also introduce unique biological questions related to allorecognition, asexual reproduction, and whole-body regeneration. As a widely used model for colonial tunicates, *B. schlosseri* provides an essential reference for studying these processes, making a high-quality genome assembly particularly valuable. Comparative synteny analyses highlight both conserved and highly rearranged genomic features across tunicates, reinforcing the notion of their exceptional genomic plasticity. By making this resource available, we aim to facilitate future research into the evolutionary and functional genomics of chordates, also highlighting unique adaptations that define tunicate biology.

## Methods

### Sampling, DNA isolation, and sequencing

Isogenic colonies of *B. schlosseri* were raised on glass slides in the marine-culture system described in Langenbacher et al. [21]. Genomic DNA was extracted from the colony labeled E* using Qiagen’s MagAttract HMW DNA Kit (67563). Libraries were prepared and sequencing was performed at Novogene for Illumina 2 × 150-bp paired-end (PE) reads, at the Next Generation Sequencing Platform of the University of Bern (Switzerland) and Leiden Genome Technology Center (Leiden, Netherlands) for HiFi PacBio long reads in round 1 and round 2, respectively (PacBio Sequel II, SMRT-bell library), and at UCAGenomix (Valbonne, France) for Oxford Nanopore (ONT) long reads (on a FLO-PRO002 flow cell with R9.4.1 pore proteins, using the SQK-LSK109 ligation sequencing kit). Nanopore base calling was performed using Guppy (RRID:SCR_023196, v3.2.10). A Hi-C library was prepared using the Arima High Coverage HiC Kit (A410110), followed by the Arima HiC+ Kit (A510008, A303011), and sequenced using Illumina (2 × 150 bp).

### Data preprocessing

PacBio HiFi reads were processed with HiFiAdapterFilt v2.0.1 [73] to remove adapter sequences, while Porechop (RRID:SCR_016967, v0.2.4) was used to trim basic adapters from ONT reads. For Illumina reads, quality trimming and adapter clipping were performed using Trimmomatic [74] (RRID:SCR_011848, v0.39), while quality check, prior to and after trimming, was done using FastQC (RRID:SCR_01458 v0.11.5).

### Genome size estimation

The genome size of colony E* was measured using an improved Feulgen protocol [75] by comparison with 2 standards of known C-values: *Periplaneta americana* (3.41 pg) [76] and *Lasius niger* (0.30 pg) [77]. In brief, the protocol steps included chopping the tissues of each specimen into tiny pieces using a sterilized razor blade with a few drops of 40% acetic acid, then leaving them for 48 hours in the dark, and immersing the processed slides into fixation reagent (85:10:5 volumes of methanol/formaldehyde/acetic acid), then hydrolyzing them (using hydrochloric acid 5M) and staining them (using Schiff’s reagent).

A digital camera (5 megapixels) mounted on a compound microscope with a 100× objective was used for imaging the slides. During the photography sessions, we maintained constant camera settings for exposure and gain, white balance calibration parameters, microscope light intensity, light condenser, and focal lens positions. In the image analysis protocol, we first outlined the nuclear boundary using the polygon tool in ImageJ [78], then extracted from ImageJ the area size of the nucleus (ASN) and the mean gray value of the nucleus (GVN). Next, we outlined in ImageJ a doughnut-shaped area surrounding the same nucleus and used it to extract the mean gray value of its background (GVB). This process was repeated for up to 30 nuclei per sample. The difference between GVB and GVN is an estimate of the average optical density (OD) of a nucleus; multiplying it by its ASN yields its integrated optical density (IOD), which is proportional to the amount of DNA in this nucleus.

Comparison of IOD values of the sample with those of the standards allows us to calculate the genome size of the sample, provided that 2 assumptions are verified: (i) all the nuclei of a given specimen contain about the same amount of DNA, and (ii) the IODs of nuclei of the standards are proportional to their known C-values. To check the first assumption of the method, we used a R script to plot for each specimen the 1/OD values of their nuclei versus their ASN values and verify that the resulting linear regression passed through the origin of the plot (Supplementary Fig. S3). To check the second assumption, we plotted the average IOD of each standard versus their known C-value and verified that the resulting line passed through the origin of the plot (Supplementary Fig. S4). As both assumptions of the method were met, we proceeded to estimating the C-value of the sample: for that, we divided the IOD of each nucleus of each standard by its known genome size, resulting in a set of 60 integrated optical densities divided by C-values (IOD/C). Finally, we used a R script to divide each of the 30 IODs of the sample by each of the 60 IOD/C values of the standards, then plotted the distribution of the resulting 1,800 estimated C-values of the sample and took the mode of its Gaussian kernel density as the most likely genome size.

A genome size estimation based on the *k*-mer spectrum of the Illumina reads was also performed using KMC v3.2.1 [79] and the GenomeScope2.0 [80] web server, with a *k*-mer size of 21 and a *k*-mer count cutoff of 100,000.

### Collapsed genome assembly

First, the PacBio HiFi reads were assembled into contigs using hifiasm [31] with the haplotype purging option disabled (option -l0 with hifiasm in HiFi-only assembly mode). Second, uncollapsed haplotypes were purged using multiple rounds of HaploMerger2 (release 20180603) [81] until the BUSCO duplication score stabilized. Third, nonmetazoan contigs were identified and removed from the assemblies using BlobToolKit v4.1.5 [82]. To this aim, contigs were aligned to the NCBI nucleotide database (accessed 18 March 2023) using BLAST [83] (RRID:SCR_001653, v2.13.0+) with the blastn command, as well as to the UniProt reference proteome database (accessed 23 March 2023) using DIAMOND [84] (RRID:SCR_016071, v2.1.6); contig HiFi coverage depth was computed using minimap2 v2.24-r1122 [85]. Using the “bestsumorder” rule of BlobToolKit, only the contigs assigned to the taxon “Chordata” or without a match (“no-hit”) were kept. Finally, a BLASTN search for fragments of the mitochondrial genome among the contigs was performed using the published complete mitochondrial genome of *B. schlosseri* (RefSeq NC_021463.1) [28] to remove contigs showing at least 80% coverage and identity with the query sequence.

To scaffold the assemblies, PacBio HiFi and Illumina reads were first mapped to the assemblies using minimap2. Putative misjoined regions were then identified and automatically split using CRAQ v1.0.9 [33] with default parameters, except for the addition of –break. Hi-C reads were subsequently mapped to the output of CRAQ using the Arima Genomics mapping pipeline script arima_mapping_pipeline.sh [86], and YaHS v1.2 [34] was run with default parameters to scaffold the assemblies. CRAQ was then applied to the results, and finally the scaffolds were manually curated using PretextMap (RRID:SCR_022023,v0.1.9) and PretextView (RRID:SCR_022024, v0.2.5). Metrics for the assemblies were computed using SeqKit v2.3.0 [87] (parameter stats -a). The quality and completeness were checked using KAT v2.4.2 [88] on *k*-mers from both PacBio HiFi and Illumina reads, as well as BUSCO v5.4.4 [89] (using the -m genome mode) with the metazoa_odb10 dataset.

### Haplotype-resolved assembly

Two haplotype-resolved assemblies (haplotype 1 and haplotype 2) were generated using hifiasm in Hi-C Integrated Assembly mode, which directly integrates Hi-C reads. To refine the assemblies, uncollapsed sequences were purged for haplotype 1 using purge_dups [90], and BlobToolKit was employed, as with the collapsed assembly, to filter out contamination, resulting in contig-level assemblies (see Supplementary Figs. S9 and S6). The scaffolding process for haplotype 1 and haplotype 2 followed the same method as for the collapsed assembly, with the final scaffolds ordered based on alignment to the collapsed assembly rather than by descending size (see Supplementary Fig. S15).

### Genome annotation

For all the assemblies, repetitive elements were identified using RepeatModeler and RepeatMasker pipeline. A *de novo* repeat library was generated using RepeatModeler2 v2.0.3 [91] and used as input for RepeatMasker (SCR_012954, v4.0.6) to detect, classify, and soft-mask repeats in the genomic sequences. RNA sequencing (RNA-seq) reads were aligned to the soft-masked assemblies using STAR v2.7.10b (default options) [92]. Based on the aligned transcripts, on a list of proteins from OrthoDB v11 for Metazoa [93] as extrinsic evidence and on the soft-masked assemblies, genes were predicted and annotated using the BRAKER3 v3.06 pipeline for RNA-seq and protein data without training or gene prediction with untranslated region (UTR) parameters [49, 94–106]. A refinement of the initial BRAKER3 structural annotation and the addition of UTRs were then performed with an implementation of the PASA pipeline v2.4.1 [50], together with EVidenceModeler (EVM) [51] (RRID:SCR_014659, v2.1.0). A third of the RNA-seq reads of the Rodriguez et al. [23] transcriptome was aligned again to the assemblies and their BRAKER3 annotation using STAR (MAX_INTRON_SIZE=20000) [92] (RRID:SCR_015899, v2.7.10b) and assembled with StringTie [107] (RRID:SCR_016323, v2.2.1) using the BRAKER3 annotation as a reference. The PASA alignment assembly step was then run as described on its GitHub Wiki with the transcripts assembled by StringTie and independently with Trinity assemblies of publicly available RNA-seq reads [8, 23, 25]. TransDecoder [108] was run within PASA to identify coding sequences within the assembled transcripts. A consensus annotation of coding sequences (CDSs) was found by EVM by leveraging both the transcripts and coding sequences identified for each RNA-seq by PASA (evidence weights: 1 for BRAKER3 input, 5 for PASA transcripts and TransDecoder CDSs). The gene models were refined, with addition of the UTRs and isoforms, by running the PASA genome annotation step sequentially with each previously generated PASA database (using EVM output as the first reference, then the output of the previous PASA genome annotation run). Functional annotation was performed starting from the structural annotation obtained with the BRAKER3-PASA pipeline. Eggnog-mapper [52, 109] and Interproscan [53, 54] were used for orthology-based annotation (nr, KEGG, Gene Ontology terms) and for protein domains prediction, respectively. Both approaches were used as input for the Funannotate pipeline (RRID:SCR_023039, v1.8.15), yielding a gff3 and a GenBank file with functional annotations.

### Mitochondrial genome assembly

The mitochondrial genome was reconstructed using NOVOPlasty [110] (RRID:SCR_017335, v4.3.1). A COI fragment from *B. schlosseri* clade A1 (GenBank MT731471.1) was used as a seed in combination with our Illumina reads as input.

### Comparative genomics analyses

The genome assemblies and annotations for the comparison of the collapsed assembly with other tunicate species were retrieved from ANISEED [111] for *Botrylloides leachii, C. robusta*, and for the first assembly of *B. schlosseri*, while *O. dioica* originates from [47], *S. thompsoni* from [48], and *S. clava* from [56]. Macrosynteny analyses were performed using the odp tool [59]. For each species, analyses were based on the longest protein isoforms generated from their annotation file using the scripts agat_sp_keep_longest_isoform.pl and agat_sp_extract_sequences.pl from AGAT (RRID:SCR_027223, v0.7.0).

### Phylogenetic analyses

COI fragments were retrieved from [44] and aligned with MUSCLE [112]. A maximum likelihood tree was generated using MEGA5 [113] with the model HKY+I+G followed by 1,000 bootstrap replicates. Phylogenetic analyses of *B. schlosseri* Hox genes were performed using sequences retrieved from Sekigami et al. [69]. First, the sequences were aligned using MUSCLE [112], as implemented in AliView [114], and then IQ-TREE 2 [115] was used to build a maximum likelihood phylogeny with the best-fit model JTT+R6 [116, 117], selected by ModelFinder [118], following the Bayesian information criterion [119] and with 10,000 ultrafast bootstrap replicates [120]. The same alignment was used to build a Bayesian tree using MrBayes (RRID:SCR_012067, V.3.2.7) (gamma-distributed rate variation across sites; mixed AA substitution models).

## Additional Files

**Supplementary Fig. S1**. Genomescope2.0 results obtained with the Illumina reads, a *k*-mer length of 21, and a maximum counts of 100,000.

**Supplementary Fig. S2**. Output of the KAT comp tool comparing the *k*-mers found in the Illumina and HiFi reads to those present in the collapsed (top), haplotype 1 (middle), and haplotype 2 (bottom) assemblies of *B. schlosseri*. The *k*-mer completeness, based on the highest peak (corresponding here to heterozygous *k*-mers), is respectively (from top to bottom) 53.03%, 47.94%, and 46.92%. A perfectly correct haploid representation should have a *k*-mer completeness of 50%.

**Supplementary Fig. S3**. Linear regressions confirming that the total amount of DNA coloration per nucleus is constant for each species, regardless of nuclear size.

**Supplementary Fig. S4**. Linear regression confirming that the integrated optical density of each standard is proportional to its known C-value.

**Supplementary Fig. S5**. Genome size histogram of *Botryllus schlosseri* obtained using Feulgen microphotodensitometry.

**Supplementary Fig. S6**. BlobPlots of the assemblies of *B. schlosseri. Initial* refers to results obtained before filtering out contamination. *Kept* represents the contigs retained in the assemblies before scaffolding, while *Removed* represents those discarded as contamination.

**Supplementary Fig. S7**. Maximum likelihood tree of *Botryllus schlosseri* clades and subclades reconstructed from COI sequences [44]. Branches shows boostrap values. Accession ID are indicated between parentheses.

**Supplementary Fig. S8**. Comparison of the percentage of genes of *Botryllus schlosseri, Ciona robusta, Oikopleura dioica*, and *Styela clava* assigned to different KEGG functional categories by BlastKOALA [55].

**Supplementary Fig. S9**. Assembly pipeline used to generate the contig-level assemblies of haplotype 1 and haplotype 2. The downstream steps (not shown) to produce scaffold-level assemblies are identical to those used for the collapsed assembly.

**Supplementary Fig. S10**. Hi-C heatmaps of the haplotype 1 (left) and haplotype 2 (right) assemblies, showing 16 chromosome-scale scaffolds for both.

**Supplementary Fig. S11**. Representation of the 2 largest palindromic regions on the sequence Bs1, based on the syntenic blocks identified by MCScanX [38] (shown in green and purple). Coverage was calculated using ONT reads, and the curve, which was smoothed using a rolling mean with a window size of 100,000 bp, does not show major deviations in the palindromic regions compared to the average coverage across the entire sequence (indicated by the dashed horizontal line). The gene names marking the start and end of each region are labeled. For example, the block extending from gene Boschl.Bs1.g184.t1 to Boschl.Bs1.g237.t1 (first green rightward arrow) is syntenic with the block from Boschl.Bs1.g370.t1 to Boschl.Bs1.g433.t1 (second green leftward arrow) in reverse order.

**Supplementary Fig. S12**. Representation of the large palindromic region on sequence Bs3. In (a), it is plotted in the same manner as in Supplementary Fig. S11. In (b), the same data are shown without smoothing the coverage curve and without restricting the coverage scaling to 200$\times$. The large peak around position 16.6 Mb corresponds to a region highly enriched in monomers likely to be centromeric repeats and is located between 2 putative topologically associating domains (see Supplementary Fig. S13).

**Supplementary Fig. S13**. (a) Tandem repeat region sizes along the sequence Bs3, based on monomers likely to be centromeric repeats and identified using quarTeT CentroMiner [123] on the collapsed assembly. A long repetitive region is observed between 16 and 17 Mb. (b) Zoom-in on the Hi-C heatmap of sequence Bs3, spanning from 12 to 22 Mb and displayed with PretextView (RRID:SCR_022024, v0.2.5), where 2 putative topologically associating domains (TADs) have been manually highlighted with red lines. The gap between the 2 putative TADs extends approximately from 16.514 to 16.595 Mb.

**Supplementary Fig. S14**. Comparisons between the 16 longest scaffolds from the collapsed, haplotype 1, and haplotype 2 assemblies and the karyogram of Colombera [35]. The lengths of the bars were calculated as the proportion (in percentage) of each chromosome’s length relative to the total genome length. The order of scaffolds for haplotype 1 and haplotype 2 is based on the sizes of the scaffolds in descending order, rather than their alignment to the collapsed assembly.

**Supplementary Fig. S15**. D-GENIES [124] dot plots of the final alignments: haplotype 1 vs. the collapsed assembly (top), haplotype 2 vs. the collapsed assembly (middle), and haplotype 1 vs. haplotype 2 (bottom). These were used to assess synteny and guide scaffold ordering.

**Supplementary Fig. S16**. AccuSyn [37] representation of syntenic blocks identified using MCScanX [38] between the 16 largest scaffolds of haplotype 1 (left, with scaffold names ending in “A”) and haplotype 2 (right, with scaffold names ending in “B”) assemblies. Inverted blocks are highlighted in red.

**Supplementary Fig. S17**. Investigation of synteny conservation among tunicate genomes. In the first column, dot plots depict the chromosome-scale scaffolds of *Botryllus schlosseri* (x-axis) plotted against those of *Styela clava, Ciona robusta*, and *Oikopleura dioica* (y-axis). Each dot in the plot represents an ortholog, specifically a reciprocal best diamond blastp match between 2 species. The units of the x- and y-axes are the number of orthologous proteins: 9,813, 5,772, and 4,064 orthologs found between the 16 chromosome-scale scaffolds of *B. schlosseri* and the 16 of *S. clava*, the 14 of *C. robusta*, and the 5 of *O. dioica*, respectively. If there were chromosome breaks, Fisher’s exact test (FET) was used to calculate the significance of the interactions between the subchromosomal pieces. Otherwise, FET was calculated on whole chromosomes. The opacity of the dots depicts the significance of FET. Dots that are a solid color are in cells with a FET *P* value less than or equal to 0.05. Dots that are translucent are in cells with a FET *P* value greater than 0.05. Dx and Dy values allow us to pinpoint places where there may be sudden breaks in synteny [58]. The second column of the figure depicts the same information as the first one, but plotted following chromosome base pair coordinates rather than gene index. This is better suited for visualizing gene-poor regions of the chromosomes.

**Supplementary Fig. S18**. Synteny conservation of bilaterian, cnidarian, and sponge linkage groups (BCnS LGs) between *Botryllus schlosseri* (Bs), *Styela clava* (Sc), *Ciona robusta* (Cr), and *Oikopleura dioica* (Od). For each species, the horizontal black lines represent the chromosomes, while the colored vertical lines connect conserved orthologs between species pairs. Each color corresponds to 1 of the 29 ancestral BCnS LGs identified in [60]. The opacity of the lines indicates the significance of the interaction between interspecies chromosomes, with solid colors representing significantly enriched conservation of synteny.

**Supplementary Fig. S19**. Phylogenetic analyses of Hox gene candidates of *Botryllus schlosseri*. The ML tree was generated using IQ-TREE 2 [115] by adding the *B. schlosseri* sequences to the alignment of Sekigami et al. [69] and keeping the homeodomains as well as the flanking 20 N-terminal and 7 C-terminal amino acids. Ultrafast bootstrap values are shown in red.

**Supplementary Fig. S20**. Phylogenetic analyses of Hox genes candidates of *Botryllus schlosseri*. The Bayesian tree was generated using MrBayes [125] by adding the *B. schlosseri* sequences to the alignment of Sekigami et al. [69] and keeping the homeodomains as well as the flanking 20 N-terminal and 7 C-terminal amino acids. Posterior probabilities are shown in red.

**Supplementary Table S1**. Metrics for the collapsed, haplotype 1, and haplotype 2 assemblies.

**Supplementary Table S2**. Comparison of the putative chromosome sizes (in kbp) across the 3 different assemblies. The putative chromosomes correspond to the 16 longest scaffolds, ordered in descending size for the collapsed assembly. For the haplotype 1 and haplotype 2 assemblies, the scaffold order is based on their alignment to the collapsed assembly, with percentages in parentheses indicating their size relative to the reference collapsed assembly.

**Supplementary Table S3**. Assembly statistics of the new collapsed assembly of *Botryllis schlosseri* compared to the existing chromosome-level reference assemblies of *Styela clava, Ciona robusta*, and *Oikopleura dioica*.

giaf097_de_thier_et_al_2025_supp_materials

giaf097_Authors_Response_To_Reviewer_Comments_Original_Submission

giaf097_GIGA-D-25-00071_original_submission

giaf097_GIGA-D-25-00071_Revision_1

giaf097_Reviewer_1_Report_Original_Submission Jerome Hui -- 3/26/2025

giaf097_Reviewer_1_Report_Revision_1 Jerome Hui -- 5/19/2025

giaf097_Reviewer_2_Report_Original_Submission Tilman Schell -- 3/26/2025

giaf097_Reviewer_3_Report_Original_Submission Cristian Canestro -- 4/2/2025

## Abbreviations

ASN: area size of the nucleus; BLAST: Basic Local Alignment Search Tool; BUSCO: Benchmarking Universal Single-Copy Orthologs; CDS: coding sequences; CLG: chordate linkage group; EVM: EVidenceModeler; GVN: gray value of the nucleus; IOD: integrated optical density; KEGG: Kyoto Encyclopedia of Genes and Genomes; NCBI: National Center for Biotechnology Information; OD: optical density; ONT: Oxford Nanopore; PE: paired-end; RNA-seq: RNA sequencing; UTR: untranslated region.

## Data Availability

The genomic and transcriptomic sequence data generated in this study are available under the BioProject accessions: PRJNA1225683. The gene expression data utilized in this study are available from The Gene Expression Omnibus (https://www.ncbi.nlm.nih.gov/geo/) under the following accessions: GSE62112, GSE193805. All additional supporting data are available in the *GigaScience* repository, GigaDB [121], and in Octopus [122].
