# Supplementary material for: First chromosome-level genome assembly of the colonial chordate model Botryllus schlosseri (Tunicata)
Source: Gigascience. 2025 Sep 18;14:giaf097. doi: 10.1093/gigascience/giaf097 (PMC12448946; doi:10.1093/gigascience/giaf097)
Supplement: giaf097_de_thier_et_al_2025_supp_materials [file giaf097_de_thier_et_al_2025_supp_materials.pdf]

Supplementary Tables

Assembly			
	**Collapsed**	**Haplotype 1**	**Haplotype 2**
Total length (Mbp) (Chr. only)	533 (513)	496 (480)	494 (464)
No. scaffolds	254	219	410
N50 (Mbp)	30 6	29	29
GC (%)	40.52	40.52	40.53
BUSCO	C:91.6%; S:90.7%; D:0.9% F:3.1%; M:5.3%	C:90.9%; S:89.9%; D:1.0% F:3.6%; M:5.5%	C:91.2%; S:88.7%; D:2.5% F:3.1%; M:5.7%
**Annotation**			
	**Collapsed**	**Haplotype 1**	**Haplotype 2**
No. genes	22,275	21,802	21,831
No. mRNAs	30,813	30,298	30,361
BUSCO	C:92.4%; S:79.7%; D:12.7% F:1.8%; M:5.8%	C:91.6%; S:80.4%; D:11.2% F:1.9%; M:6.5%	C:92.1%; S:79.1%; D:13.0% F:1.6%; M:6.3%

Table S1. Metrics for the collapsed, haplotype 1, and haplotype 2 assemblies.

Chromosome	Collapsed	Haplotype 1	Haplotype 2
1	50,110	41,841 (83%)	28,053 (56%)
2	44,705	40,598 (91%)	45,038 (101%)
3	41,815	38,360 (92%)	29,155 (70%)
4	33,037	30,504 (92%)	32,607 (99%)
5	32,846	28,210 (86%)	28,942 (88%)
6	32,755	31,460 (96%)	32,176 (98%)
7	30,789	29,986 (97%)	29,040 (94%)
8	30,038	28,423 (95%)	28,982 (96%)
9	29,634	27,923 (94%)	30,153 (102%)
10	29,082	28,687 (99%)	27,860 (96%)
11	28,207	29,008 (103%)	27,153 (96%)
12	27,998	26,703 (95%)	26,494 (95%)
13	26,676	25,071 (94%)	25,027 (94%)
14	25,737	25,698 (100%)	25,542 (99%)
15	24,956	24,523 (98%)	23,565 (94%)
16	24,314	22,647 (93%)	23,761 (98%)

Table S2. Comparison of the putative chromosome sizes (in kbp) across the three different assemblies. The putative chromosomes correspond to the 16 longest scaffolds, ordered in descending size for the collapsed assembly. For the haplotype 1 and haplotype 2 assemblies, the scaffold order is based on their alignment to the collapsed assembly, with percentages in parentheses indicating their size relative to the reference collapsed assembly.

Measure	*B. schlosseri*	*S. clava*	*C. robusta*	*O. dioica*
Length (Mbp)	533	340	115	64
No. of sequences	254	211	1,272	19
N50 (Mbp)	30	21	5	16
GC (%)	40.52	35.27	34.72	41.04
No. of annotated genes	22,275	19,966	16,406	17,259
BUSCO Complete (Single, Duplicated)	91.6% (90.7%, 0.9%)	90.5% (84.3%, 6.2%)	93.7% (93.1%, 0.6%)	59.4% (56.5%, 2.9%)
BUSCO Fragmented	3.1%	3.8%	2.3%	9.6%
BUSCO Missing	5.3%	5.7%	4.0%	31.0%

Table S3. Assembly statistics of the new collapsed assembly of *Botryllis schlosseri* compared to the existing chromosome-level reference assemblies of *Styela clava*, *Ciona robusta* and *Oikopleura dioica*.

Supplementary Figures

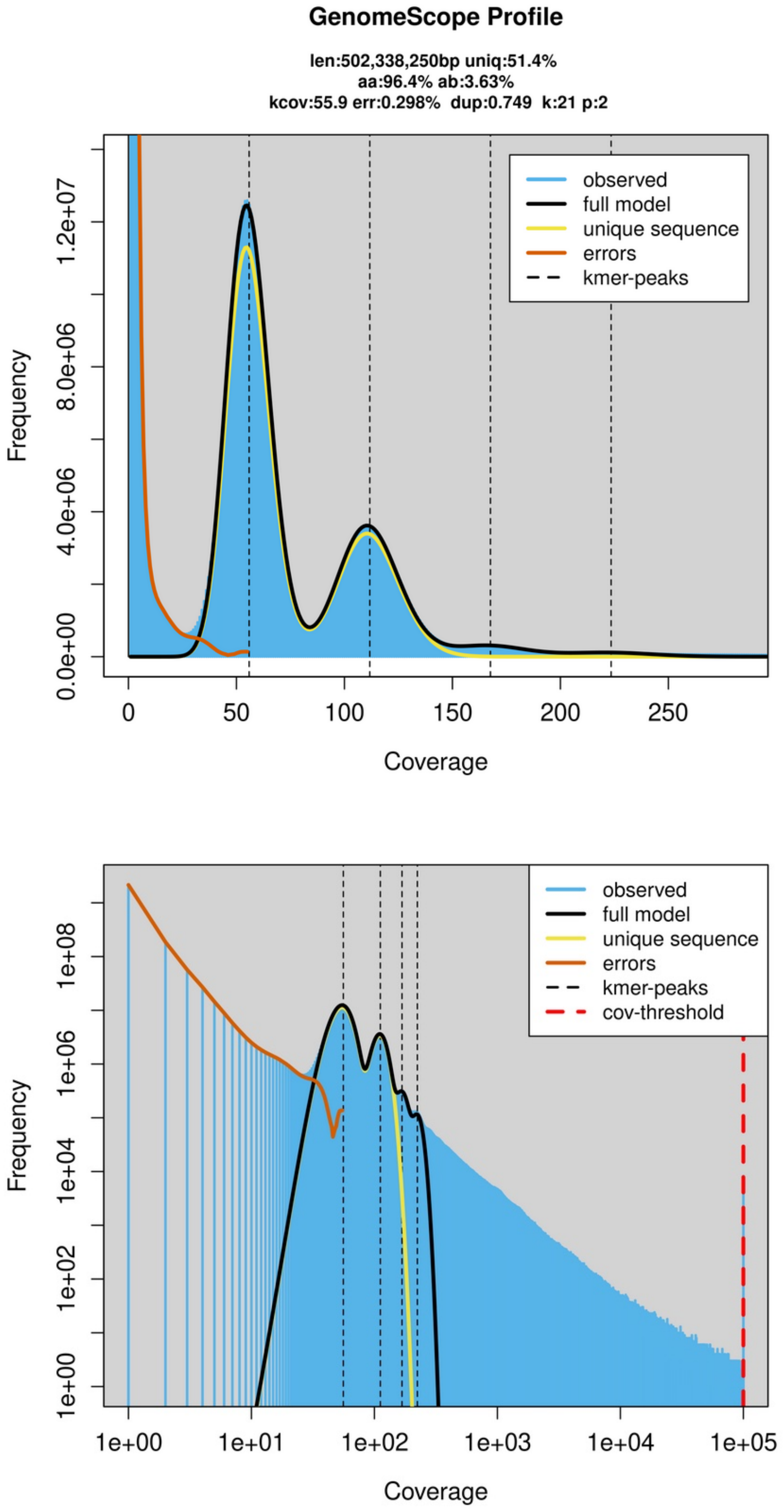

Figure S1. Genomescope2.0 results obtained with the Illumina reads; a k-mer length of 21 and a maximum counts of 100,000.

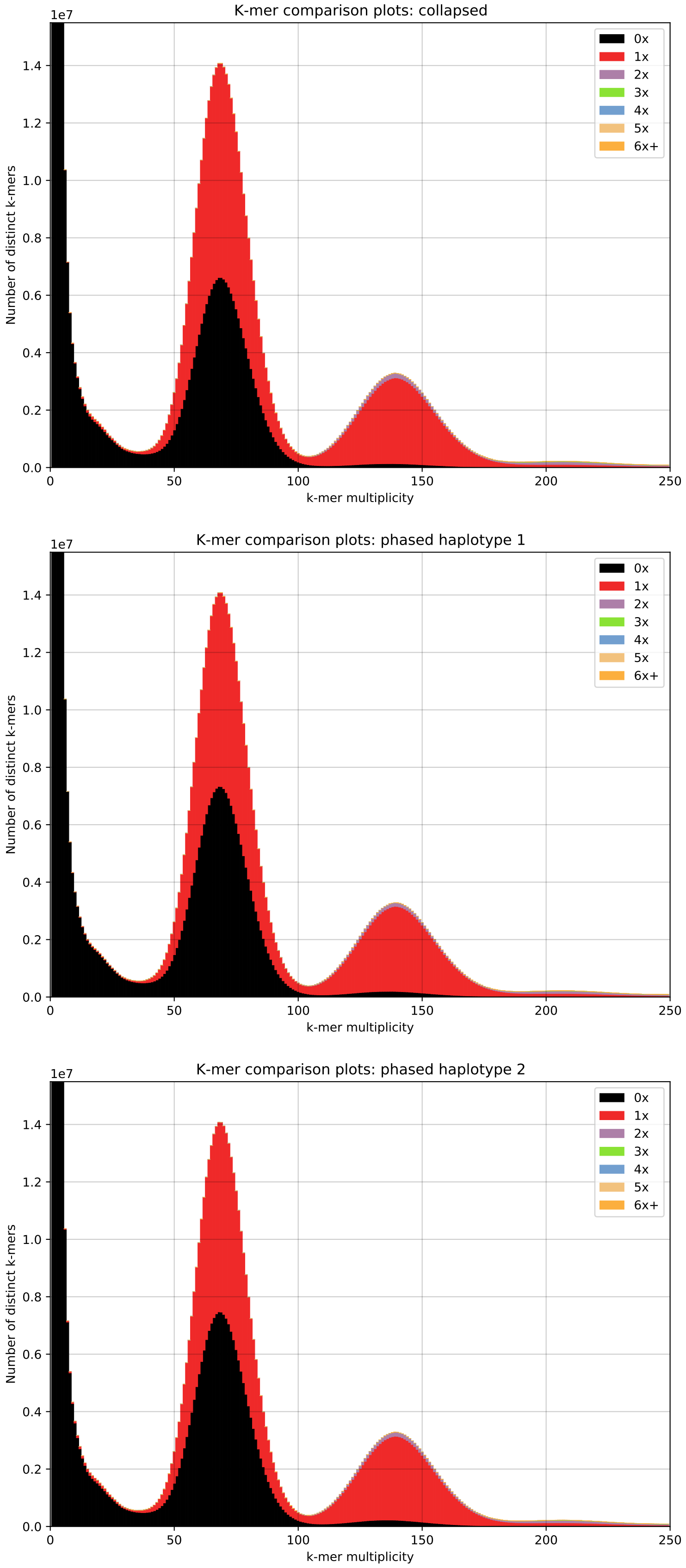

Figure S2. Output of the KAT comp tool comparing the k-mers found in the Illumina and HiFi reads to those present in the collapsed (top), haplotype 1 (middle) and haplotype 2 (bottom) assemblies of *B. schlosseri*. The k-mer completeness, based on the highest peak (corresponding here to heterozygous k-mers), is respectively (from top to bottom) 53.03%, 47.94%, and 46.92%. A perfectly correct haploid representation should have a k-mer completeness of 50%.

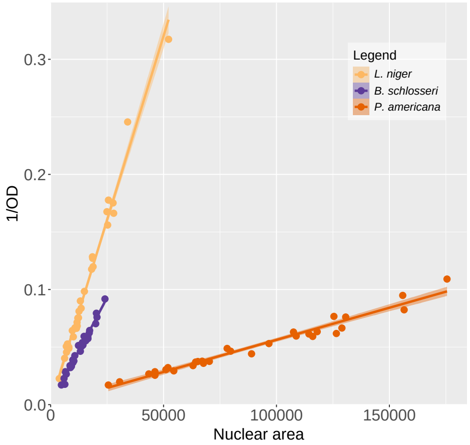

Figure S3. Linear regressions confirming that the total amount of DNA coloration per nucleus is constant for each species, regardless of nuclear size.

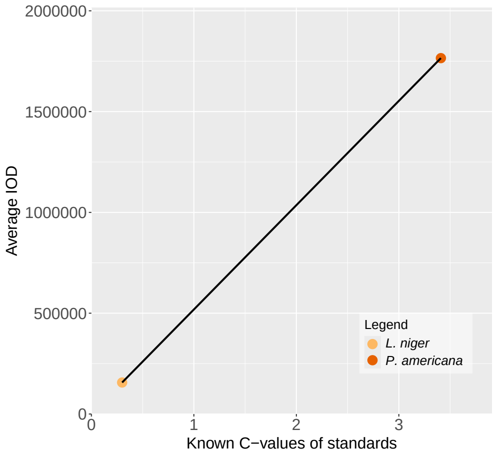

Figure S4. Linear regression confirming that the integrated optical density of each standard is proportional to its known C-value

.

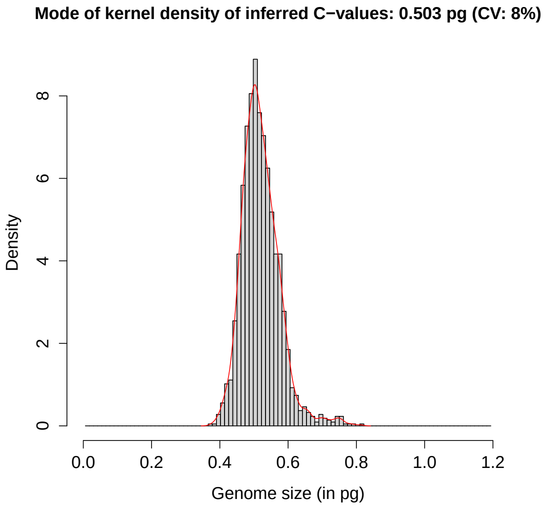

Figure S5. Genome size histogram of *Botryllus schlosseri* obtained using Feulgen microphotodensitometry.

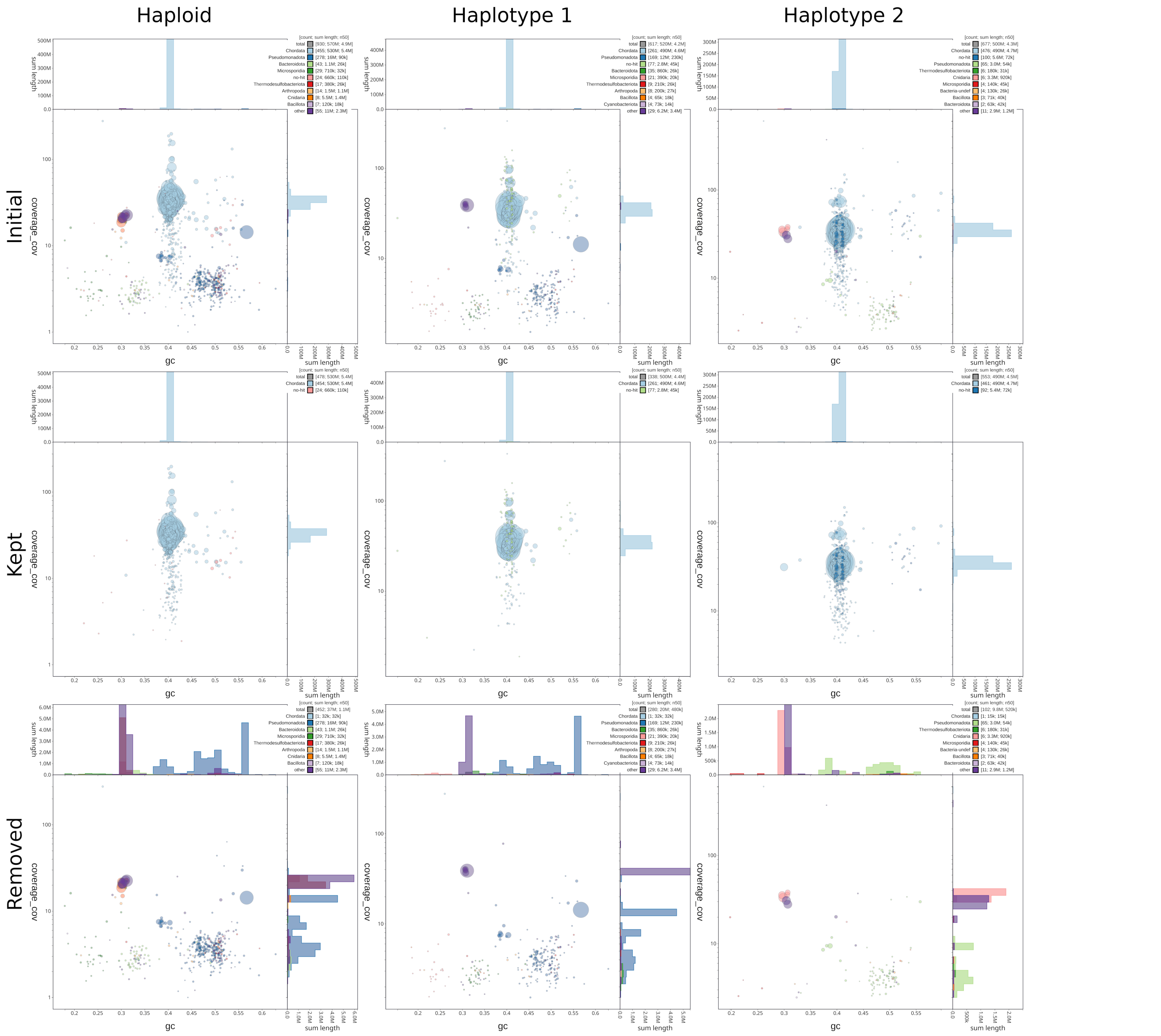

Figure S6. BlobPlots of the assemblies of *B. schlosseri*. **Initial** refers to results obtained before filtering out contamination. **Kept** represents the contigs retained in the assemblies before scaffolding, while **Removed** represents those discarded as contamination.

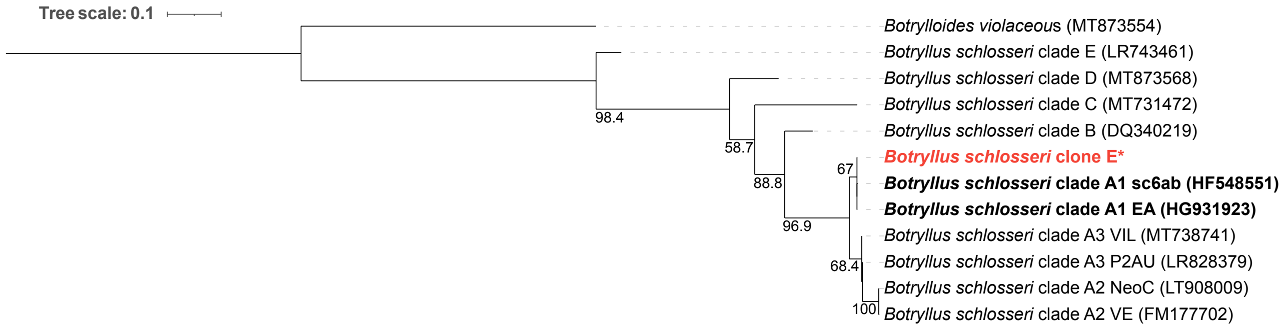

Figure S7. Maximum-likelihood tree of *Botryllus schlosseri* clades and sub-clades reconstructed from COI sequences [44]. Branches shows boostrap values. Accession ID are indicated between parenthesis.

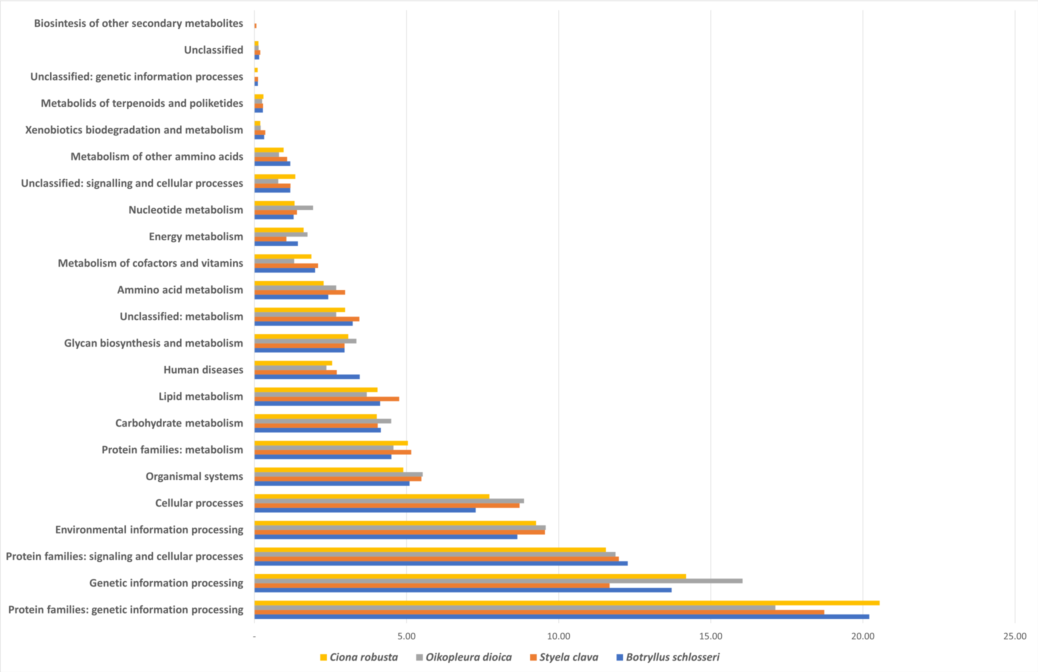

Figure S8. Comparison of the percentage of genes of *Botryllus schlosseri*, *Ciona robusta*, *Oikopleura dioica* and *Styela clava* assigned to different KEGG functional categories by BlastKOALA [55].

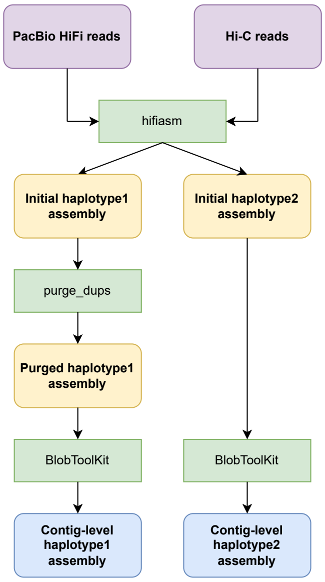

Figure S9. Assembly pipeline used to generate the contig-level assemblies of haplotype 1 and haplotype 2. The downstream steps (not shown) to produce scaffold-level assemblies are identical to those used for the collapsed assembly.

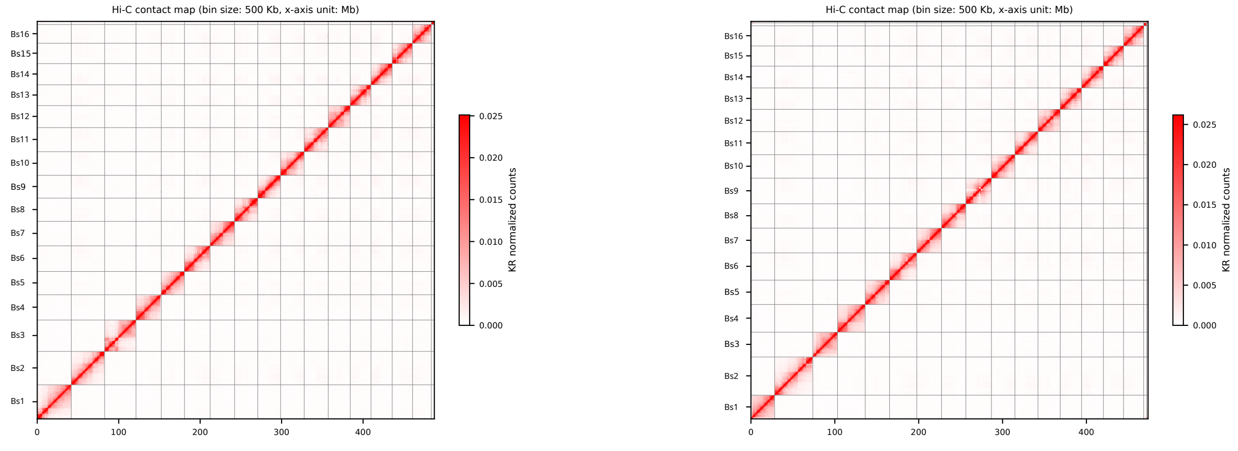

(a) Haplotype 1

(b) Haplotype 2

Figure S10. Hi-C heatmaps of the haplotype 1 (left) and haplotype 2 (right) assemblies, showing sixteen chromosome-scale scaffolds for both.

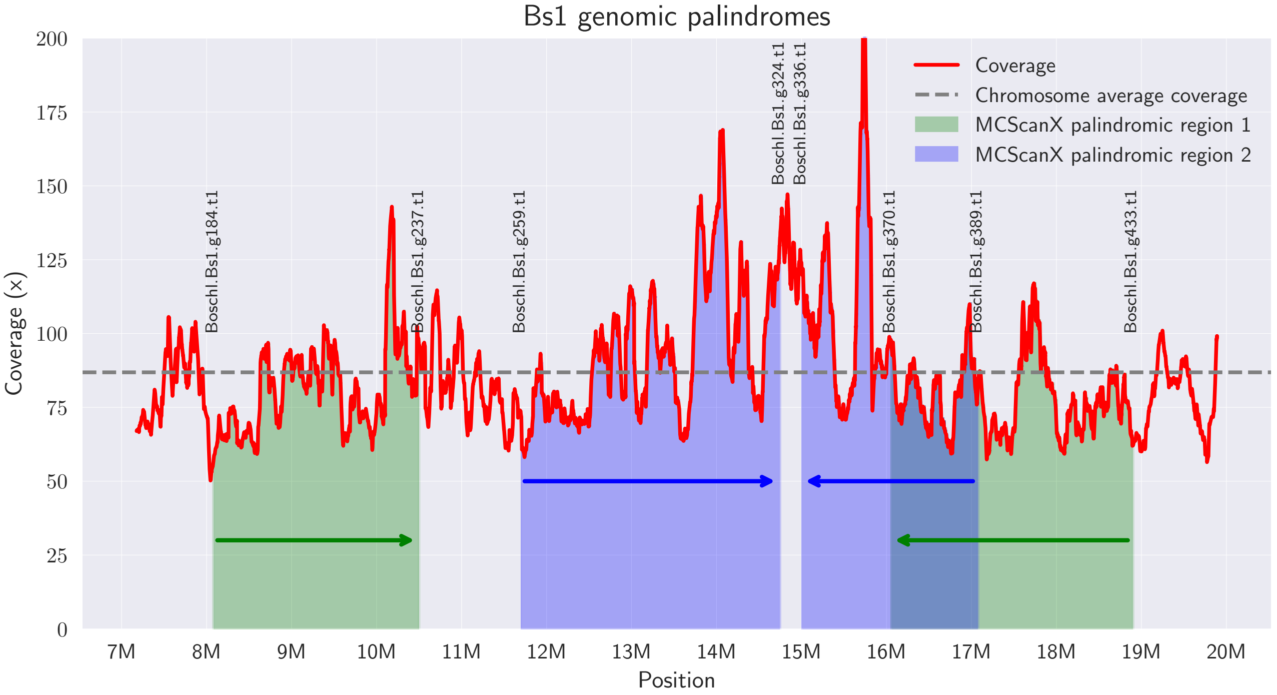

Figure S11. Representation of the two largest palindromic regions on the sequence Bs1, based on the syntenic blocks identified by MCScanX [38] (shown in green and purple). Coverage was calculated using ONT reads and the curve, which was smoothed using a rolling mean with a window size of 100,000 bp, does not show major deviations in the palindromic regions compared to the average coverage across the entire sequence (indicated by the dashed horizontal line). The gene names marking the start and end of each region are labeled. For example, the block extending from gene Boschl.Bs1.g184.t1 to Boschl.Bs1.g237.t1 (first green rightward arrow) is syntenic with the block from Boschl.Bs1.g370.t1 to Boschl.Bs1.g433.t1 (second green leftward arrow) in reverse order.

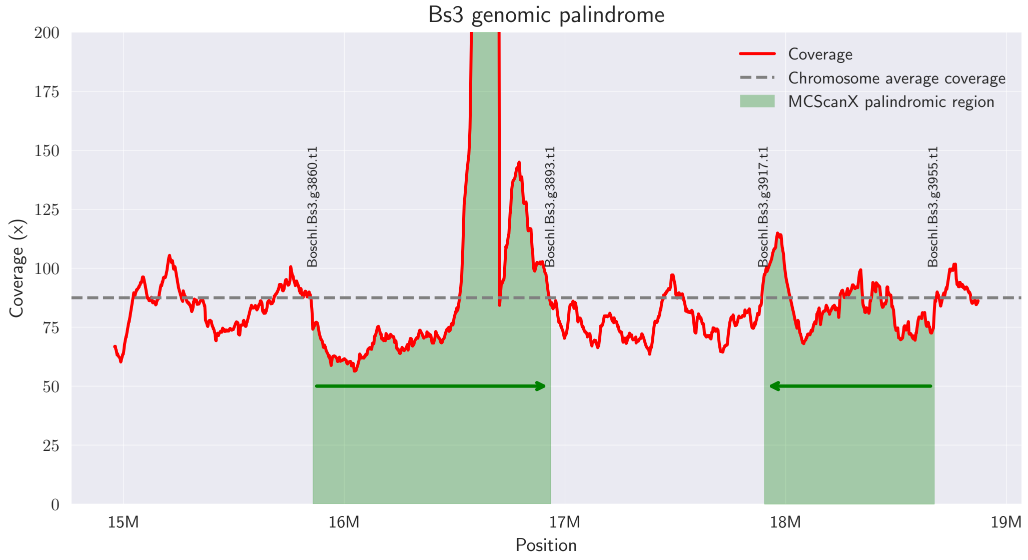

(a)

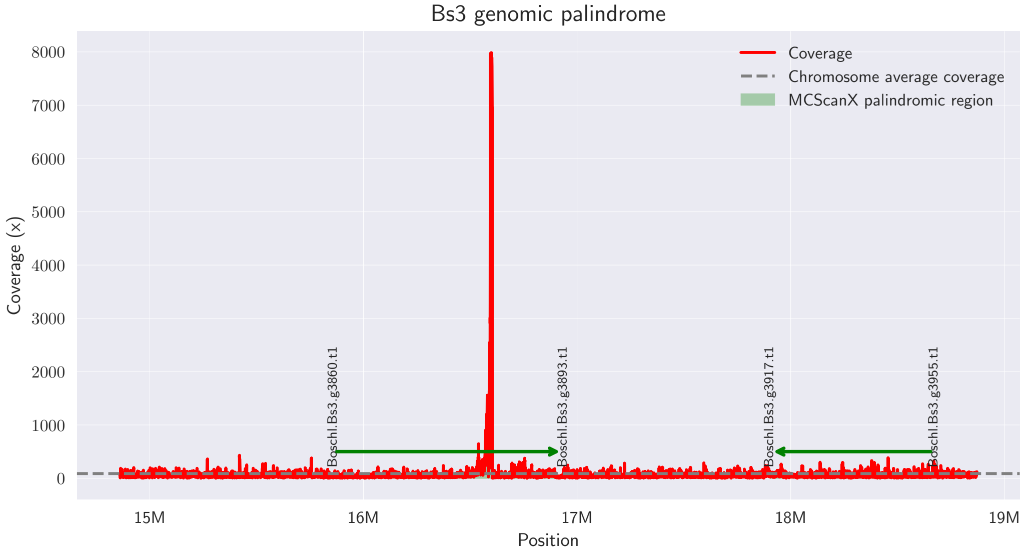

(b)

Figure S12. Representation of the large palindromic region on sequence Bs3. In **(a)**, it is plotted in the same manner as in Supplementary Figure S11. In **(b)**, the same data are shown without smoothing the coverage curve and without restricting the coverage scaling to 200×. The large peak around position 16.6 Mb corresponds to a region highly enriched in monomers likely to be centromeric repeats, and is located between two putative topologically associating domains (see Supplementary Figure S13).

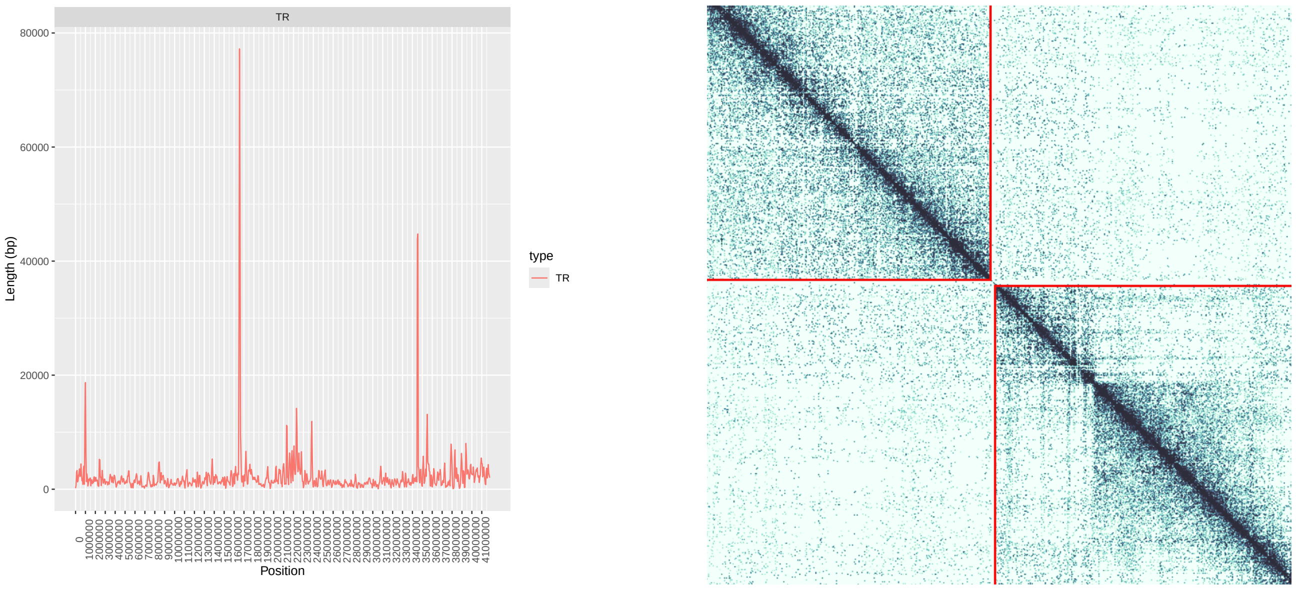

(a) **(b)**

Figure S13. **(a)** Tandem repeat region sizes along the sequence Bs3, based on monomers likely to be centromeric repeats and identified using quarTeT CentroMiner [123] on the collapsed assembly. A long repetitive region is observed between 16 and 17 Mb. **(b)** Zoom-in on the Hi-C heatmap of sequence Bs3, spanning from 12 Mb to 22 Mb and displayed with PretextView (RRID:SCR_022024, v0.2.5), where two putative topologically associating domains (TADs) have been manually highlighted with red lines. The gap between the two putative TADs extends approximately from 16.514 Mb to 16.595 Mb.

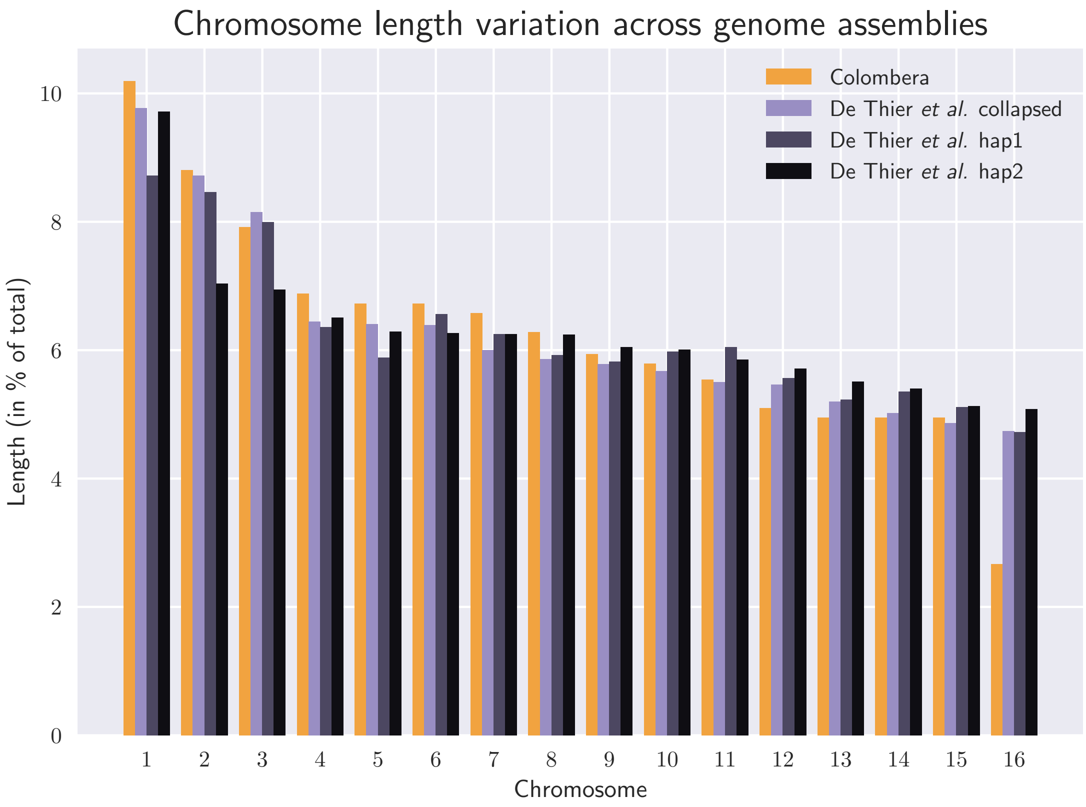

Figure S14. Comparisons between the 16 longest scaffolds from the collapsed, haplotype 1, and haplotype 2 assemblies and the karyogram of Colombera [35]. The lengths of the bars were calculated as the proportion (in percentage) of each chromosome's length relative to the total genome length. The order of scaffolds for haplotype 1 and haplotype 2 is based on the sizes of the scaffolds in descending order, rather than their alignment to the collapsed assembly.

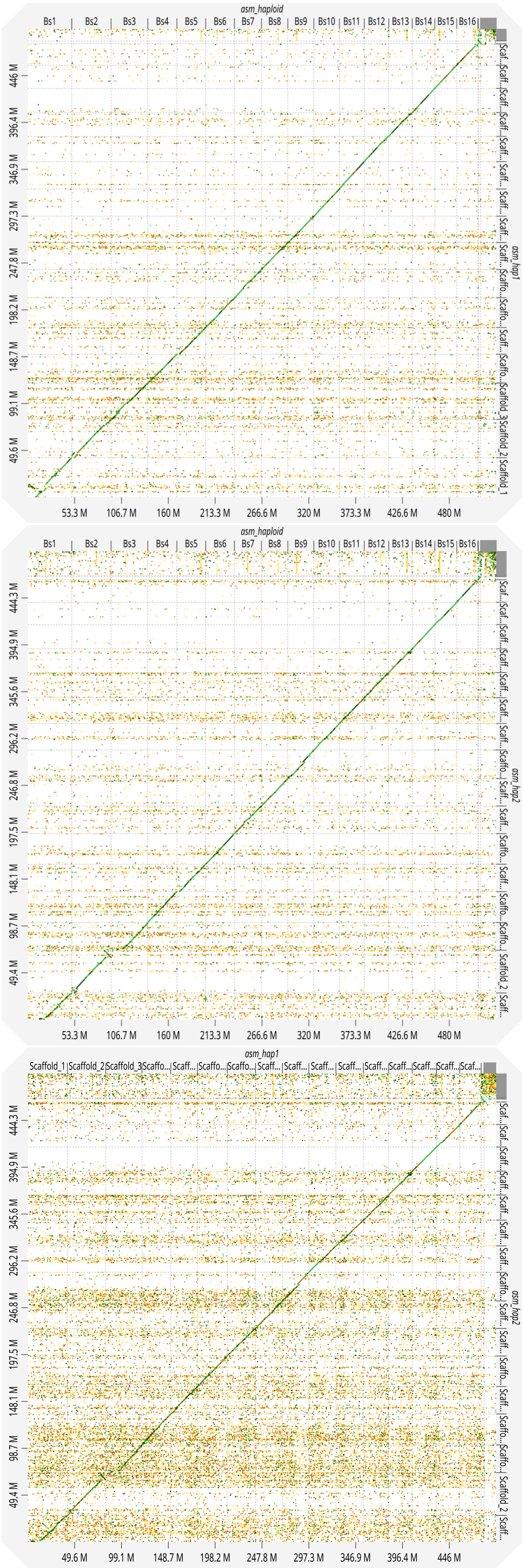

Figure S15. D-GENIES [124] dot plots of the final alignments: haplotype 1 vs. the collapsed assembly (top), haplotype 2 vs. the collapsed assembly (middle), and haplotype 1 vs. haplotype 2 (bottom). These were used to assess synteny and guide scaffold ordering.

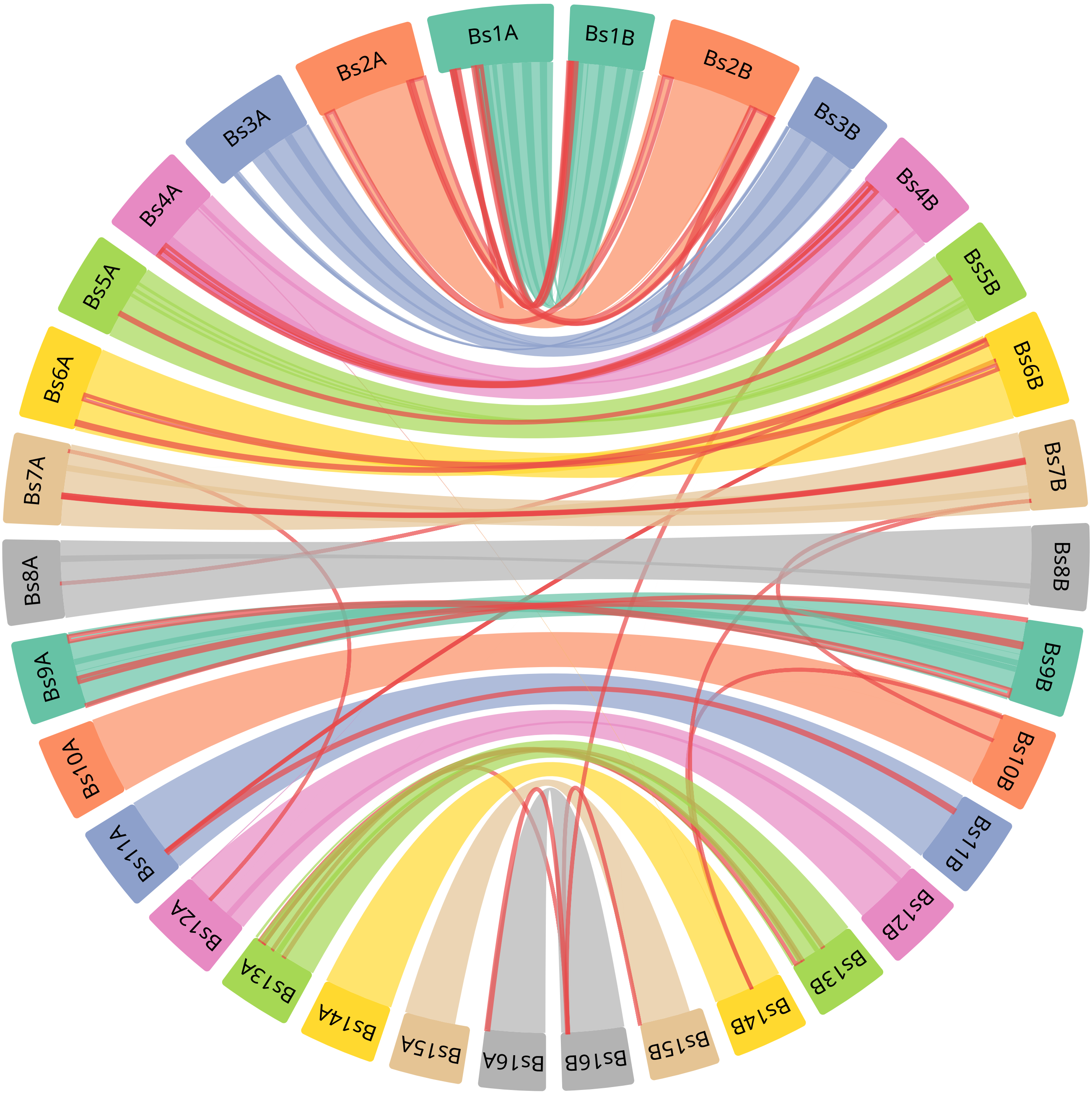

Figure S16. AccuSyn [37] representation of syntenic blocks identified using MCScanX [38] between the 16 largest scaffolds of haplotype 1 (left, with scaffold names ending in 'A') and haplotype 2 (right, with scaffold names ending in 'B') assemblies. Inverted blocks are highlighted in red.

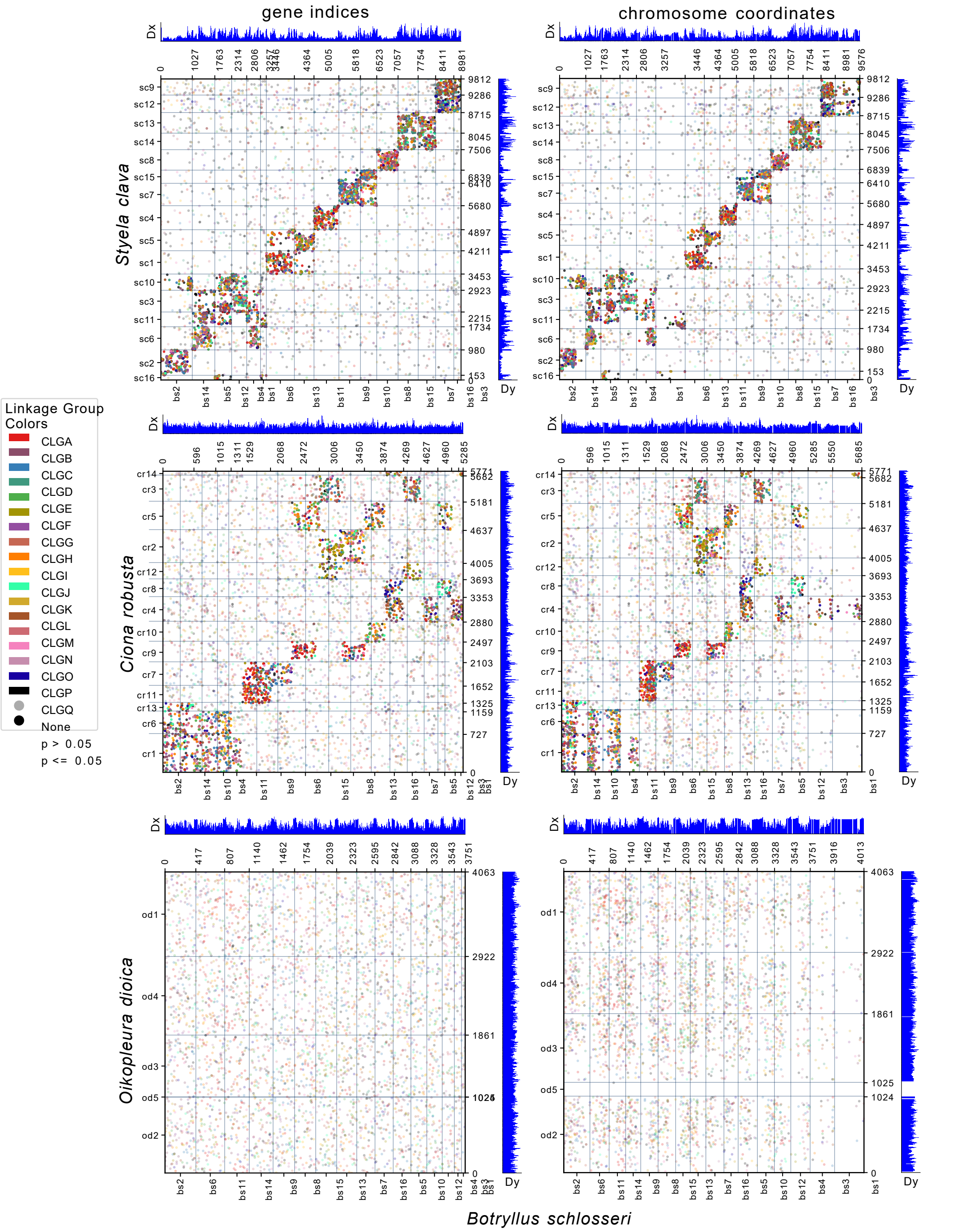

Figure S17. Investigation of synteny conservation among tunicate genomes. In the first column, dot plots depict the chromosome-scale scaffolds of *Botryllus schlosseri* (x-axis) plotted against those of *Styela clava*, *Ciona robusta* and *Oikopleura dioica* (y-axis). Each dot in the plot represents an ortholog, specifically a reciprocal best diamond blastp match between two species. The units of the x- and y-axes are the number of orthologous proteins: 9813, 5772 and 4064 orthologs found between the 16 chromosome-scale scaffolds of *B. schlosseri* and the 16 of *S. clava*, the 14 of *C. robusta* and the 5 of *O. dioica*, respectively. If there were chromosome breaks, Fisher's exact test (FET) was used to calculate the significance of the interactions between the sub-chromosomal pieces. Otherwise, FET was calculated on whole chromosomes. The opacity of the dots depicts the significance of FET. Dots that are a solid color are in cells with a FET p-value less than or equal to 0.05. Dots that are translucent are in cells with a FET p-value greater than 0.05. Dx and Dy values allow to pinpoint places where there may be sudden breaks in synteny [58] The second column of the figure depicts the same information as the first one, but plotted following chromosome base pair coordinates rather than gene index. This is better suited for visualizing gene-poor regions of the chromosomes.

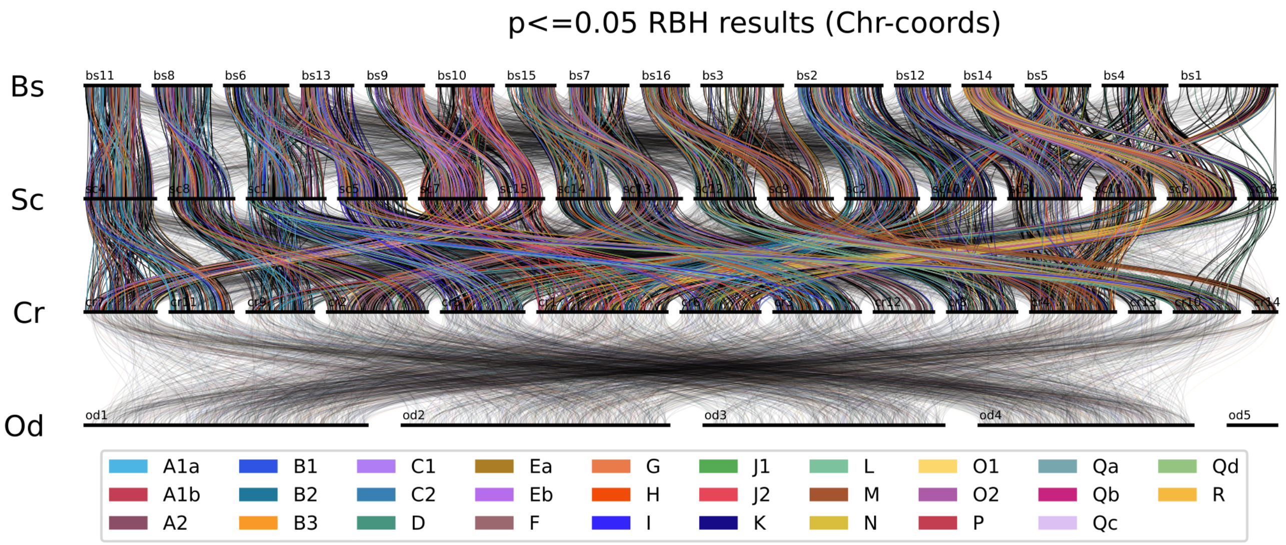

Figure S18. Synteny conservation of bilaterians, cnidarians and sponges linkage groups (BCnS LGs) between *Botryllus schlosseri* (Bs), *Styela clava* (Sc), *Ciona robusta* (Cr) and *Oikopleura dioica* (Od). For each species, the horizontal black lines represent the chromosomes, while the colored vertical lines connect conserved orthologs between species pairs. Each color corresponds to one of the 29 ancestral BCnS LGs identified in [60]. The opacity of the lines indicates the significance of the interaction between inter-species chromosomes, with solid colors representing significantly enriched conservation of synteny.

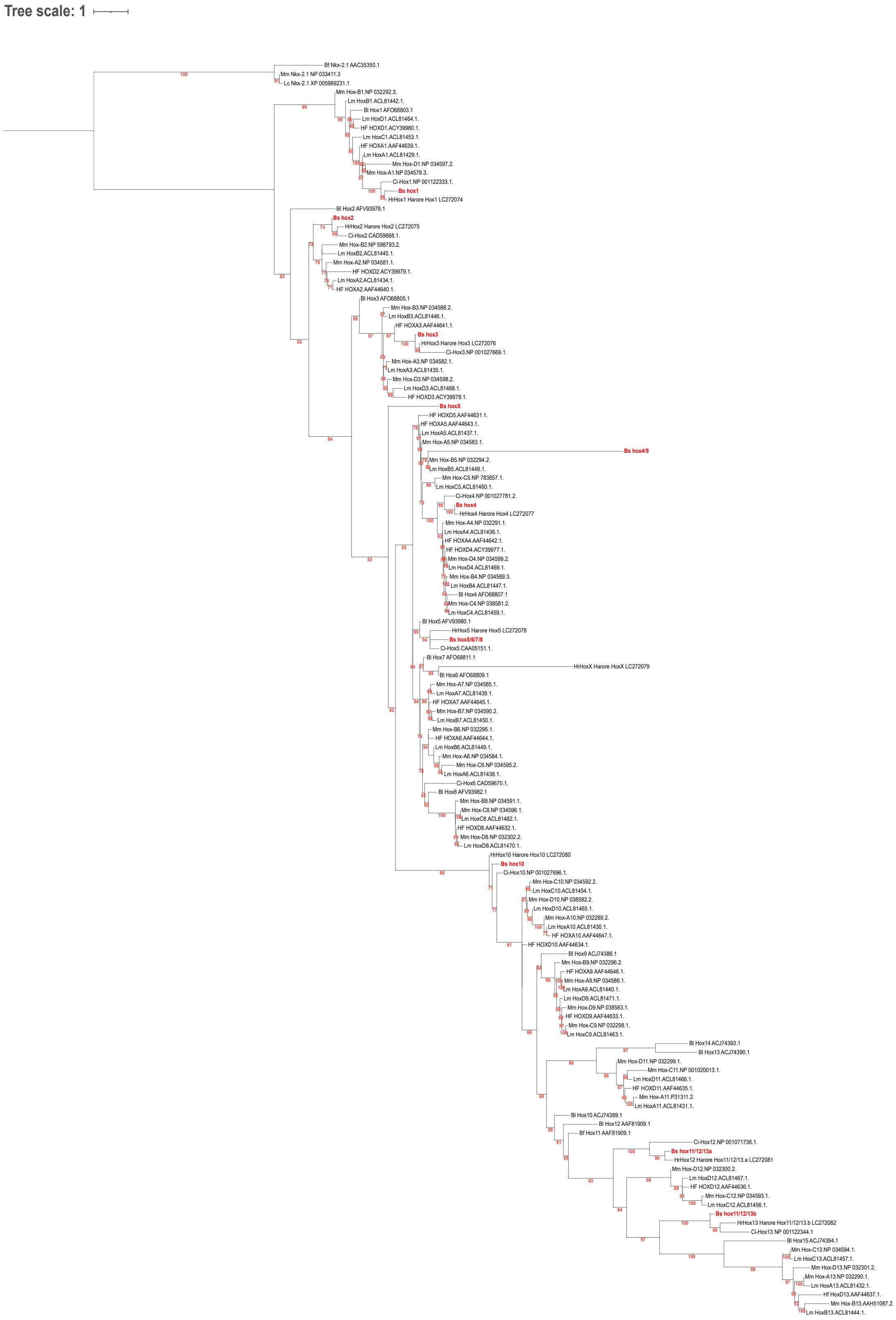

Figure S19. Phylogenetic analyses of Hox genes candidates of *Botryllus schlosseri*. The ML tree was generated using IQ-TREE 2 [115] by adding the *B. schlosseri* sequences to the alignment of Sekigami et al. 2017 [69] and keeping the homeodomains as well as the flanking 20 N-terminal and seven C-terminal aminoacids. Ultrafast bootstrap values are shown in red.

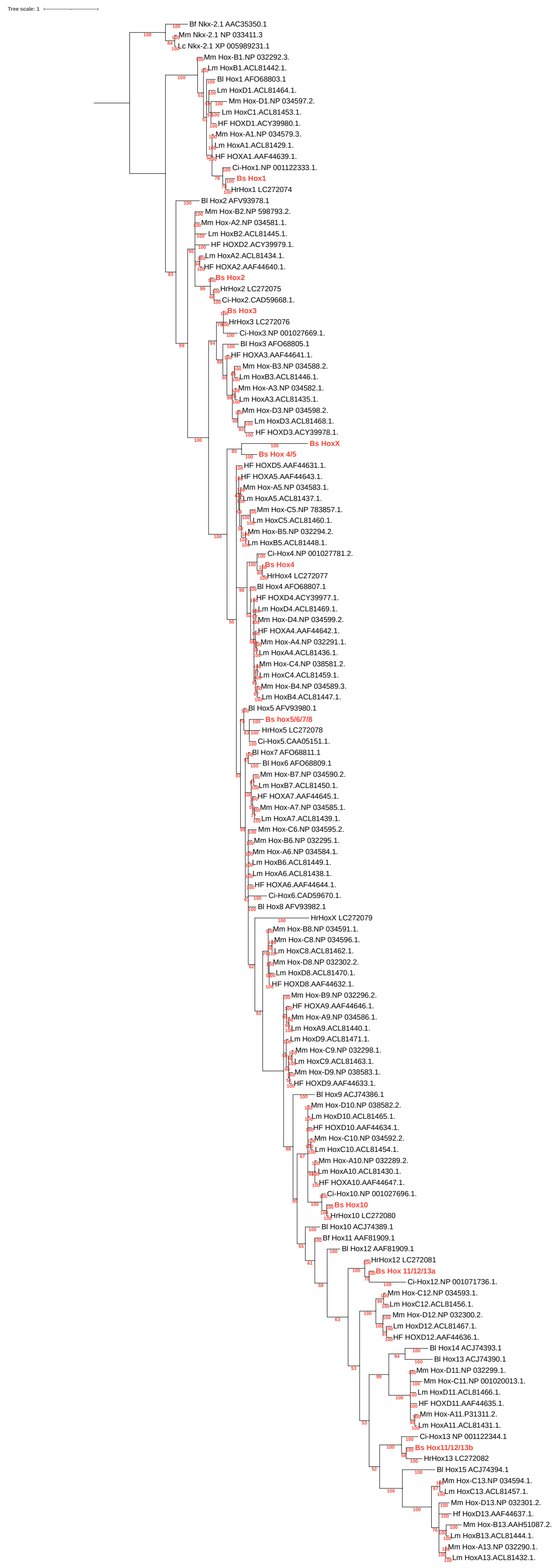

Figure S20. Phylogenetic analyses of Hox genes candidates of *Botryllus schlosseri*. The Bayesian tree was generated using MrBayes [125] by adding the *B. schlosseri* sequences to the alignment of Sekigami et al. 2017 [69] and keeping the homeodomains as well as the flanking 20 N-terminal and seven C-terminal aminoacids. Posterior probabilities are shown in red.
