# Supplementary material for: First chromosome-level genome assembly of the colonial chordate model Botryllus schlosseri (Tunicata)
Source: Gigascience. 2025 Sep 18;14:giaf097. doi: 10.1093/gigascience/giaf097 (PMC12448946; doi:10.1093/gigascience/giaf097)
Supplement: giaf097_GIGA-D-25-00071_original_submission [file giaf097_giga-d-25-00071_original_submission.pdf]

GigaScience

First chromosome-level genome assembly of the colonial chordate model Botryllus schlosseri (Tunicata)

--Manuscript Draft--

**Manuscript Number:**	GIGA-D-25-00071
**Full Title:**	First chromosome-level genome assembly of the colonial chordate model Botryllus schlosseri (Tunicata)
**Article Type:**	Research
**Funding Information:**	Agence Nationale de la Recherche (ANR-14-CE02-0019-01) — Dr. Stefano Tiozzo<br>Agence Nationale de la Recherche (ANR- 24-CE02-2277) — Dr. Stefano Tiozzo<br>Centre National de la Recherche Scientifique (INSB-DBM) — Dr. Stefano Tiozzo<br>Fundação de Amparo à Pesquisa do Estado de São Paulo (15/50164-5 & 19/06927-5) — Dr. Federico D. Brown
**Abstract:**	Background: Botryllus schlosseri (Tunicata) is a laboratory model, colonial tunicate recognized for its remarkable developmental diversity, regenerative abilities, and a peculiar genetically determined allorecognition system governed by a polymorphic locus controlling chimerism and cell parasitism. Results: We report the first chromosome-level genome assembly of B. schlosseri sub-clade A1. By integrating long and short reads with Hi-C scaffolding, we produced both a phased diploid genome assembly and a conventional haploid consensus sequence of 533 Mb. Of this total length, 96% belonged to 16 chromosome-scale scaffolds, with a BUSCO completeness score of 91.6%. We then compared our assembly with other high-quality tunicate genomes, revealing some synteny conservation but also extensive genomic rearrangements and a general loss of colinearity. Conclusions: The chromosome-level resolution of this assembly enhances our understanding of genome organization in colonial modular organisms. Comparative analyses highlight the dynamic nature of tunicate genomes, with conserved macrosynteny yet extensive microsyntenic rearrangements and scrambling, underscoring their rapid evolutionary trajectory. This high-quality genome assembly provides a valuable resource for exploring the unique biological features of colonial chordates, including its exceptional regenerative abilities and complex allorecognition system.
**Corresponding Author:**	Stefano Tiozzo<br>CNRS: Centre National de la Recherche Scientifique<br>Villefranche sur Mer, PACA FRANCE
**Corresponding Author Secondary Information:**	
**Corresponding Author's Institution:**	CNRS: Centre National de la Recherche Scientifique
**Corresponding Author's Secondary Institution:**	
**First Author:**	Olivier De Thier
**First Author Secondary Information:**	
**Order of Authors:**	Olivier De Thier<br>Marie Lebel<br>Mohammed M. Tawfeeq

Powered by Editorial Manager® and ProduXion Manager® from Aries Systems Corporation

	Roland Faure
	Philippe Dru
	Simon Blanchoud
	Alexandre Alie
	Federico D. Brown
	Jean-Francois Flot
	Stefano Tiozzo
**Order of Authors Secondary Information:**	
**Additional Information:**	
**Question**	**Response**
Are you submitting this manuscript to a special series or article collection?	No
**Experimental design and statistics**<br><br>Full details of the experimental design and statistical methods used should be given in the Methods section, as detailed in our Minimum Standards Reporting Checklist. Information essential to interpreting the data presented should be made available in the figure legends.<br><br>Have you included all the information requested in your manuscript?	Yes
**Resources**<br><br>A description of all resources used, including antibodies, cell lines, animals and software tools, with enough information to allow them to be uniquely identified, should be included in the Methods section. Authors are strongly encouraged to cite Research Resource Identifiers (RRIDs) for antibodies, model organisms and tools, where possible.<br><br>Have you included the information requested as detailed in our Minimum Standards Reporting Checklist?	Yes
**Availability of data and materials**	Yes

Powered by Editorial Manager® and ProduXion Manager® from Aries Systems Corporation

All datasets and code on which the conclusions of the paper rely must be either included in your submission or deposited in publicly available repositories (where available and ethically appropriate), referencing such data using a unique identifier in the references and in the "Availability of Data and Materials" section of your manuscript.<br><br>Have you have met the above requirement as detailed in our Minimum Standards Reporting Checklist?	

Powered by Editorial Manager® and ProduXion Manager® from Aries Systems Corporation

Manuscript complete

Click here to access/download;Manuscript;zzfkxjgxqdcxpzgmfkrmrcbdkcyyz

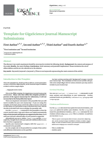

GigaScience, 2023, 1–25

doi: xx.xxxx/xxxx
Manuscript in Preparation
Paper

PAPER

First chromosome-level genome assembly of the colonial chordate model *Botryllus schlosseri* (Tunicata)

Olivier De Thier[1,2], Marie Lebel[3], Mohammed M.Tawfeeq[1,2], Roland Faure[1,2], Philippe Dru[3], Simon Blanchoud[4], Alexandre Alié[3], Federico D. Brown[5], Jean-François Flot[1,2*] and Stefano Tiozzo[3*]

[1]Evolutionary Biology & Ecology, C.P. 160/12, Université libre de Bruxelles (ULB), Avenue F.D. Roosevelt 50, B-1050 Brussels, Belgium and [2]Interuniversity Institute of Bioinformatics in Brussels – (IB)^2, Brussels, Belgium and [3]CNRS, Laboratoire de Biologie du Développement de Villefranche Sur-mer (LBDV), Sorbonne Université, Paris, France and [4]Department of Biology, University of Fribourg, Fribourg, Switzerland and [5]Departmento de Zoologia, Instituto de Biociências, Universidade de São Paulo, São Paulo - SP, Brazil

[*]jean-francois.flot@ulb.be; stefano.tiozzo@imev-mer.fr

Abstract

Background: *Botryllus schlosseri* (Tunicata) is a laboratory model, colonial tunicate recognized for its remarkable developmental diversity, regenerative abilities, and a peculiar genetically determined allorecognition system governed by a polymorphic locus controlling chimerism and cell parasitism. **Results**: We report the first chromosome-level genome assembly of *B. schlosseri* sub-clade A1. By integrating long and short reads with Hi-C scaffolding, we produced both a phased diploid genome assembly and a conventional haploid consensus sequence of 533 Mb. Of this total length, 96% belonged to 16 chromosome-scale scaffolds, with a BUSCO completeness score of 91.6%. We then compared our assembly with other high-quality tunicate genomes, revealing some synteny conservation but also extensive genomic rearrangements and a general loss of colinearity. **Conclusions**: The chromosome-level resolution of this assembly enhances our understanding of genome organization in colonial modular organisms. Comparative analyses highlight the dynamic nature of tunicate genomes, with conserved macrosynteny yet extensive microsyntenic rearrangements and scrambling, underscoring their rapid evolutionary trajectory. This high-quality genome assembly provides a valuable resource for exploring the unique biological features of colonial chordates, including its exceptional regenerative abilities and complex allorecognition system.

Key words: budding; regeneration; chimerism; ascidian; coloniality; model organism

Introduction

There are colonies of pelagic tunicates [. . .]. Each member of the colony is an individual animal, but the colony is another individual animal, not like the sum of its individuals [. . .]. So a man of individualistic reason, if he must ask, "Which is the animal?" must abandon his particular kind of reason and say, "Why, it's two animals and they aren't alike any more than the cells of my body are like me. I am much more than the sum of my cells, and, for all I know, they are much more than the division of me"

—John Steinbeck *The Log from the Sea of Cortez*

In the sub-phylum Tunicata, the sister group of vertebrates [1], colonial species reproduce both sexually and asexually through various forms of budding. Through budding, new functional bodies emerge from adult somatic cells and tissues. Regardless of variations in budding modes among tunicate species [2] and of whether development occurs through asexual budding or sexually via embryogenesis, the basic body plan of adult tunicates is highly conserved across the entire sub-phylum [3]. In colonial tunicates, the

Compiled on: February 19, 2025.
Draft manuscript prepared by the author.

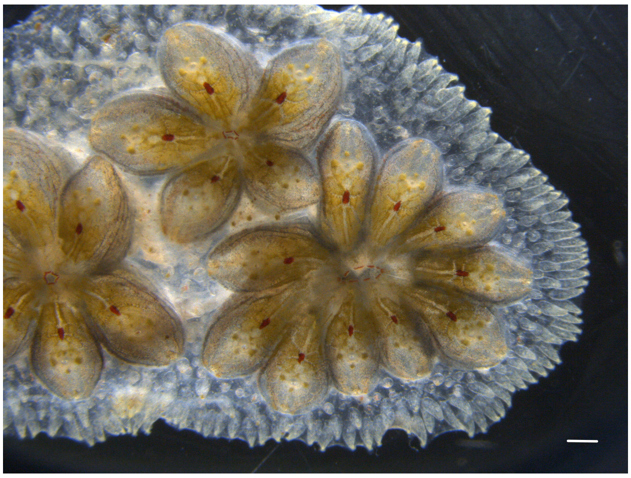

Figure 1. Colony of *Botryllus schlosseri*. Scale bar 1mm.

asexually generated individuals generally remain physically connected, forming colonies. Colony formation, clonal reproduction, and modular organization have important physiological, ecological, and evolutionary implications. For example, the modular organization supports rapid growth on hard, space-limited substrates, outperforming solitary forms. Morphological plasticity enables colony-level adaptation to predation, damage, or environmental changes. Furthermore, uniparental reproduction, including asexual reproduction, likely provides a selective advantage for rapid colonization on invasion fronts or in disturbed habitats (reviewed in [4]). Like many other colonial tunicates, *Botryllus schlosseri* can generate a functional adult body via three distinct developmental pathways. The first involves sexual reproduction, where the fertilized egg passes through a larval stage and develops into the initial colony founder. The second pathway is asexual propagation, where the founder zooid continuously reproduces through palleal (aka peribranchial) budding, forming a colony of hundreds of zooids connected by a network of extra-corporeal vessels within a cellulose-based extracellular matrix, or "tunic" [5] (Figure 1). Lastly, if all zooids and buds are removed from a *B. schlosseri* colony, new buds can regenerate from the vascular system in a process known as vascular budding, allowing asexual propagation and eventual colony reformation [6, 7, 8]. Zooids within a single colony are genetically identical clones. However, wild colonies often come into contact and fuse, resulting in chimeras where circulating cells carry different genotypes. These mixed pools of circulating cells contribute to sexual and, according to some authors, to asexual and regenerative development [9, 10, 11]. During chimerism, donor cells may entirely replace the host's germline or somatic cells, a phenomenon termed germ cell or somatic cell parasitism, respectively [12, 13, 11]. As a result, zooids within a chimeric colony are not always clonemates.

Botryllus schlosseri was introduced to laboratories over half a century ago [14] as a model to study asexual development, regeneration [15], allorecognition and chimerism [16, 17]. Over recent decades, a dedicated scientific community has emerged, advancing breeding techniques and developing imaging and molecular biology tools to better study this species [18, 19, 20, 9, 21, 8]. Several anatomical descriptions and staging methods have been proposed [22, 5] and extensive transcriptomic databases for various developmental stages and tissues have been generated [23, 24, 25, 26, 27, 8]. In 2013, a draft genome of *B. schlosseri* was published [28] but it lacked the completeness and continuity required by today's assembly standards [29]. In this study, we present high-quality, chromosome-level, haploid and phased genome assemblies for *B. schlosseri*. This new resource offers a robust platform for investigating the developmental and regenerative processes, complex allorecognition, chimerism, and cell parasitism defined by the biology of this colonial chordate.

Results and Discussion

Sequencing and genome size estimation

Genomic DNA extracted from a laboratory-reared, non-chimeric colony derived from a single zygote (referred to as clone E*) yielded 489 million Illumina (short) paired-end 150 bp reads, 2.4 million PacBio HiFi (long) reads with a N50 length of ~9.5 kb (max length of ~50 kb) and 7.4 million ONT (long) reads with a N50 length of ~10.5 kb (max length of ~205 kb) (Table 1).

Technology	Tot. size (Gbp)	Number of reads	N50 (bp)	Coverage
Illumina	73.2	488,906,094	150	146
Illumina Hi-C	15.9	106,488,252	150	32
PacBio HiFi (round 1)	7.9	1,170,137	8,711	16
PacBio HiFi (round 2)	10.8	1,218,052	10,151	22
ONT (R9.4.1)	58.9	10,888,103	10,320	118

Table 1. Sequencing technology applied to sequence *B. schlosseri* genome (clone E*) and relative read statistics.

Based on k-mer analyses, the genome size was estimated to be around 500 Mbp (~444 Mbp using a maximum k-mer count of 10,000 and ~515 Mbp using a maximum k-mer count of 10,000,000) with a heterozygosity of 3.63% (Figures S1 and S2), whereas a chemical approach using Feulgen densitometry estimate was ~499 Mbp (using 1 pg = 978 Mbp) (Figure S3), which is less than a previous cytofluorimetric-based estimation of 725 Mb [30] and than the first genome assembly obtained by Voskoboynik *et al.* [28], which had a size of 580 Mbp.

Haploid genome assembly

An initial purged primary assembly was obtained using hifiasm [31]; it had a size of 570 Mbp and comprised 930 contigs with an N50 length of 4.9 Mbp. In this assembly, the analysis of BlobToolKit results identified 452 contigs, totaling 37 Mbp, as putative contaminations and mitochondrial DNA (see next section), which were subsequently removed. Of these 37 Mbp, approximately half were attributed to members of the bacterial phylum Pseudomonadota (Figure S4). We identified 28 contigs that belong to spore-forming unicellular parasites of the microsporidia group [32]. This represents the first report of this fungal group in a tunicate species. However, we cannot rule out the possibility that these sequences originate from contaminants present in the water, rather than from parasitized *Botryllus* tissues. The remaining contigs were corrected using CRAQ [33], which detects and breaks misassembled contigs; this raised the total number of contigs in the assembly from 478 to 516. We then performed Hi-C scaffolding using YaHS [34], which reduced the number of contigs to 256, before running CRAQ again on the scaffolded assembly (this time, 4 misassembled contigs were detected and broken). Finally, a manual curation was performed, resulting in an assembly made up of 16 major scaffolds containing around 96% (513 Mbp) of the total sequence length (533 Mbp) (Table 2, Figures 2 and 3). The full assembly pipeline is summarized in Figure 4 and detailed in the Material and Methods section.

The completeness of our assembly was assessed using the Benchmarking Universal Single-Copy Orthologs (BUSCO) tool [38], which returned a genome completeness of 91.6% (including 0.9% of duplicated marker genes), compared to 74.4% (including 23.7% of duplicated genes) for the assembly of Voskoboynik *et al.* (Figure 5). This high duplication score of the previously available assembly indicates that their larger assembly size (580 Mbp vs. 533 Mbp)

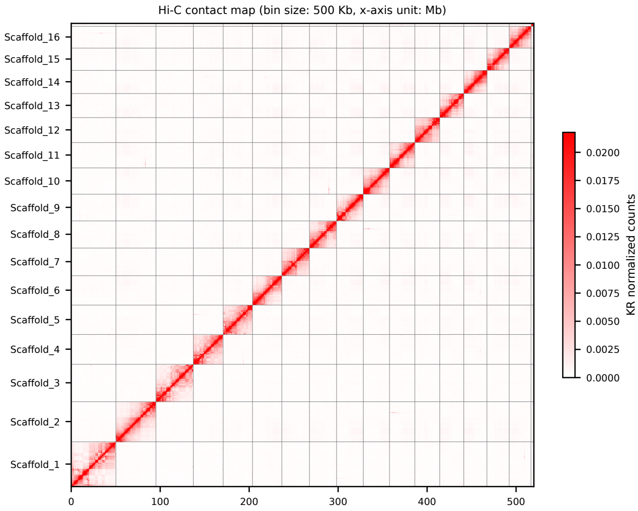

Figure 2. Hi-C heatmap of the haploid assembly of the *Botryllus schlosseri* genome showing sixteen chromosome-scale scaffolds. The figure was generated using the visualization module of HapHiC [35].

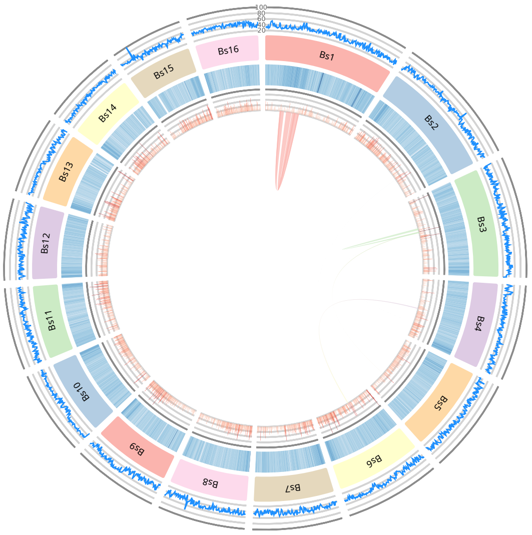

Figure 3. Circos plot of the distribution of several genomic characteristics along the 16 longest scaffolds of the haploid assembly and represented as chromosomes named Bs1 to Bs16 (made with AccuSyn [36]). Each layer of the circle represents, from the inside to the outside: the syntenic blocks retrieved with MCScanX [37]; the histogram of the gene density; a heatmap of the presence of repetitive elements; the reference chromosomes in clockwise orientation and the read coverage using the HiFi reads.

Measure	All scaffolds	16 longest scaffolds
Length (Mbp)	533	513
No. of scaffolds	254	16
N50 (Mbp)	30	31
GC (%)	40.52	40.46
No. of annotated genes	22,275	21,677
BUSCO Complete (Single, Duplicated)	91.6% (90.7%, 0.9%)	91.4% (90.7%, 0.7%)
BUSCO Fragmented	3.1%	3.1%
BUSCO Missing	5.3%	5.5%

Table 2. Assembly statistics for all the scaffolds and for the 16 longest ones.

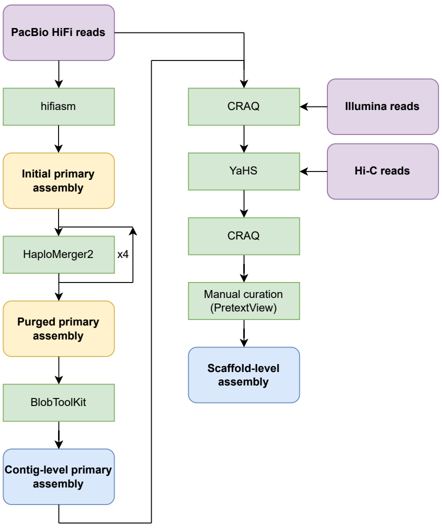

Figure 4. Assembly pipeline for the haploid genome assembly (see Material and Methods.)

was caused by incompletely collapsed haplotypes [39]. Synteny analysis performed with MCScanX [37] highlighted the presence of two large-scale genomic palindromes located within Bs1 and a smaller one in Bs3 (Figure 3). To find out whether these palindromes may result from assembly artifacts [40], we checked the localization of the duplicated BUSCO genes along the chromosomes and did another run of CRAQ, but this time using ONT as long reads (with higher coverage compared with the HiFi reads used in the previous rounds). There was no significant difference in the number of duplicated BUSCO genes within Bs1 and Bs3 compared to the others, and CRAQ did not detect structural errors in this scaffold either. These observations suggest that the palindromes observed are real, with potential biological significance that will require further investigation.

The laboratory model Sub-clade A1

The complete mitochondrial DNA was recovered and assembled as a single circular contig using our Illumina reads with NOVOPlasty [41]. Our mitogenome shares 99.95% identity with the published mitochondrial sequence assigned to the *B.schlosseri* sub-clade A1 [42]. Notably, this sub-clade includes the sc6ab specimen, which Voskoboynik *et al.* [28] used to generate the previous reference assembly of *B. schlosseri*. Our mitogenome assembly further shares 99.7% nucleotide identity with that reference sequence. Phylogenetic analyses based on a COI fragment used as DNA barcode for ascidians ([42]) confirm that sample E* belongs to sub-clade A1 (Figure S5), a group that is both widely distributed and employed as a laboratory model worldwide.

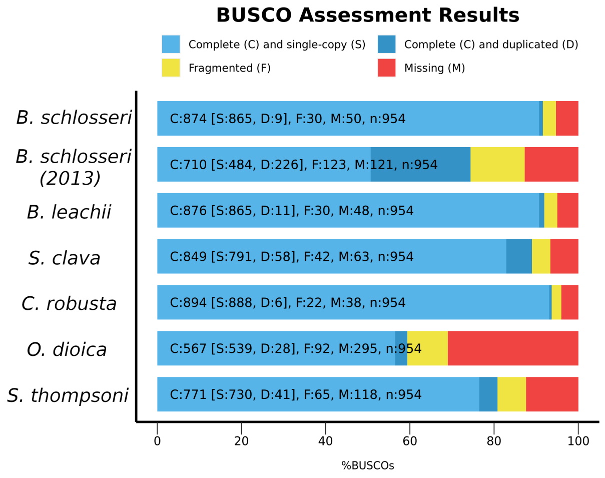

Figure 5. Orthology assignment in previous tunicate genome projects. Proportion of BUSCO genes detected or missed in the new genome assembly of *B. schlosseri* compared to the previous assembly (*B. schlosseri (2013)* [28]) and other reference genomes.

Repeat class	Percent of genome
**LINEs**	**4.52%**
LINE1	0.15%
LINE2	2.06%
**LTR elements**	**1.34%**
**DNA elements**	**7.24%**
hAT-Charlie	2.96%
TcMar-Tigger	0.01%
**Unclassified**	**46.03%**
**Total interspersed repeats**	**59.12%**
**Simple repeats**	**3.94%**
**Low complexity**	**0.02%**
**Total**	**63.09%**

Table 3. Classes of repeats in the *Botryllus schlosseri* genome. RepeatMasker summary table output for the haploid genome of *Botryllus schlosseri* showing the different classes in percentages of identified repeats.

Structural and functional annotation

RepeatMasker, using a *de novo* repeat library created by RepeatModeler, detected that around 63% of the novel *B. schlosseri* haploid genome assembly consists of repetitive elements, which is close to the 65% of repeats found in the previously published assembly [28]. Most of these were interspersed repeats (see Table 3). *Ab initio* genome annotation using the BRAKER3 pipeline [43] initially predicted 16,966 coding genes, after which refinement using the PASA pipeline [44, 45] finally retrieved 22,275 genes coding for 30,813 proteins (see Table 4). This number is significantly lower than originally predicted for *B. schlosseri* (38,730 predicted genes [28]), probably due to the incomplete collapse of the previous assembly. In terms of completeness of the annotation, BUSCO retrieved 92.4% complete (79.7% single, 12.7% duplicated) and 1.8% fragmented metazoan genes when given all predicted isoforms, whereas it retrieved 92% complete (91% single, 0.9% duplicated) and 1.8% fragmented of those genes when filtered to only keep their longest isoform. This is consistent with the results obtained by running BUSCO directly on the scaffold sequences.

The functional annotation and orthology assignment [46], coupled with annotation of protein domains, motifs, and functional sites [47, 48], were written into gff3 and Genbank files (available upon publication). KEGG route-mapping assigned 7,221 genes over the annotated entries and distributed them across 21 KEGG categories (Figure 6). Among them, the most prevalent ones include KEGG hierarchies dealing with genetic information processing (2449/7219, 22,92%), such as DNA replication, repair, recombination, transcription, translation and regulation of gene expression; signaling and cellular processes (886/7219, 12,27%); and environmental information processing (674/7219, 8,64%) such as various cellular processes and signaling pathways involved in sensing, transducing (i.e. MAPK signaling, PI3K-Akt signaling and cAMP signaling), responding to external signals (i.e. G-protein coupled receptors, receptor tyrosine kinases, and cytokine receptors), intracellular communication and cell motility. The KEGG annotations provided for *B. schlosseri* are consistent and coherent with the functional annotation of the published complete genomes of other ascidian tunicates such as *Styela plicata*, *Ciona robusta* and *Oikopleura dioica* (Figure S6).

Type	Number	Mean size (bp)	% genome
Gene	22275	8566.13	35.78
mRNA	30813	10576.62	N/A
cds	237200	199.16	8.86
Exon	241815	289.83	13.14
5' UTR	21386	432.29	1.73
3' UTR	20985	648.00	2.55
Total	574474	1143.44	N/A

Table 4. Gene predictions and annotation statistics.

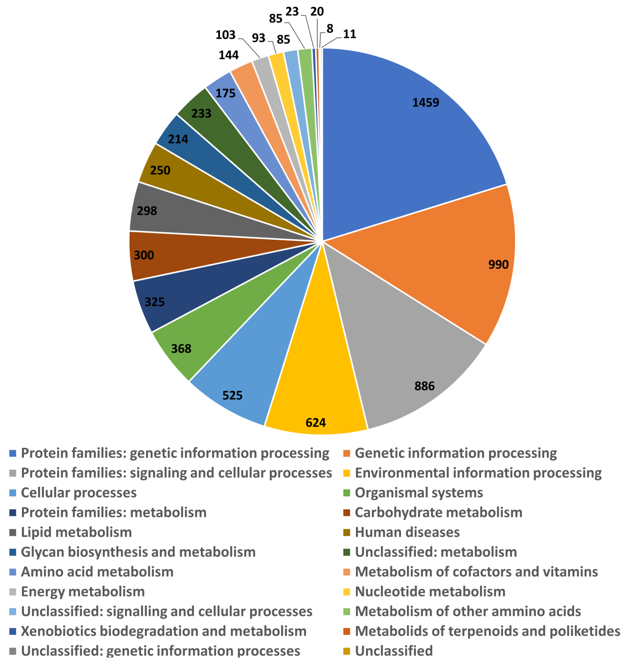

Figure 6. Pie chart of the assignation of the annotated genes of *Botryllus schlosseri* to KEGG functional categories using BlastKOALA [49].

Haplotype-resolved assembly

With a heterozygosity level exceeding 3%, haplotype-resolved assemblies of *B. schlosseri* are crucial for studying differences between homologous chromosomes, such as structural variations. Using hifiasm with direct integration of Hi-C reads and subsequent scaffolding (Figure S7), we generated a pair of haplotype-resolved assemblies (haplotype 1 and haplotype 2), each organized into 16 major scaffolds (see Figure S8). With respective sizes of 496 Mbp and 494 Mbp, these assemblies are smaller than the haploid collapsed assembly (533 Mbp). When considering only the 16 longest scaffolds, the sizes decrease to 480 Mbp for haplotype 1 and 464 Mbp for haplotype 2, compared to 513 Mbp for the haploid assembly. Additionally, their BUSCO completeness scores are lower, with values of 90.9% and 91.2%, respectively, compared to 91.6% for the haploid assembly. This is further reflected in their annotation results, where fewer genes were identified: 21,802 and 21,831 for haplotype 1 and haplotype 2, respectively, versus 22,275 for the haploid assembly (see Table S1). The observed differences in metrics, where the results for the haplotype-resolved assemblies are inferior to those for the haploid assembly, may be attributed to misassemblies, particularly deletions. For example, when comparing the putative chromosome lengths (see Table S2) for chromosomes 1 and 3, we observe a significant disparity in sizes between the two haplotypes, which may be attributed to incomplete sequence reconstructions during the assembly process. Such anomalies may additionally be observed when comparing the putative chromosome lengths of all assemblies with the karyogram of *B. schlosseri*, as described by Colombera [50] (see Figure S9). Notably, the sizes of the haploid assembly appear to more closely match the expected distribution compared to the haplotypes. Also, it can be seen that Colombera's observation of a smaller size for the 16th chromosome compared to the others is not reflected in any of the assemblies. Furthermore, multiple structural variations between the two haplotypes, particularly small inversions (see Figures S10 and S11), seem to be present in the majority of the homologous chromosomes. However, as with the observed putative deletions, these may result from misassemblies and require further validation to enhance the quality of the haplotype-resolved assembly.

Synteny analyses

To assess macrosynteny conservation between *Botryllus schlosseri* and other tunicates, we selected genomes that met two specific criteria: they were assembled at the chromosome level, ensuring comparable high-quality structural information, and they represented, as much as possible, the breadth of diversity within the tunicate subphylum. *Styela clava* [51] belongs to the same order as *Botryllus* (Stolidobranchia), *Ciona robusta* [52] to a different order (Phlebobranchia), and *Oikopleura dioica* [53] to a different class of tunicates (Appendicularia) [54]. We used 17 groups of orthologous genes identified by Simakov *et al.* as ancestral chordate linkage groups (CLGs) [55]. These groups of genes are thought to have remained physically linked since the divergence of the Olfactores lineage (which includes both vertebrates and tunicates) from cephalochordate. However, Oxford dot plots [56] revealed a general loss of syntenic equivalence [57] among tunicate genomes, even between *B. schlosseri* and *S. clava*, which share the same haploid chromosome number of 16. Despite this identical number of chromosomes, the comparison between the two stolidobranchs showed extensive chromosome rearrangements, including fissions and fusions with mixing [58, 57] (Figures 7 and S12). These rearrangements are even more pronounced in *C.robusta*, which has a haploid chromosome number of 14. The overall random distribution of ortholog pairs within blocks points to significant order scrambling, resulting in a loss of colinearity(*i.e.* the sequential order of genes along the same chromosome), the comparison with *Oikopleura dioica* shows a complete breakdown of both macrosynteny and colinearity, with CLGs fully scrambled and dispersed. The latter result is consistent with the very long and fast-evolving branch of the Appendicularia when compared to the other tunicates [54] as well as the extreme genome scrambling rate of Appendicularia compared to other tunicates and mammals [59]. The same analyses using a set of 29 linkage groups generally conserved among bilaterians, cnidarians and sponges [57] yielded similar results (Figure S13).

Hox gene analyses

Hox genes are a subset of homeobox genes that play important developmental roles in the specification of body segments along the anterior-posterior axis. Their arrangement into a syntenic cluster colinear with gene expression is conserved across Bilateria, with some exceptions [60]. In the current haploid assembly, we retrieved ten *B. schlosseri Hox* genes, which is consistent with draft genomes of other ascidian tunicates [61]. Orthology of *B. schlosseri Hox* genes was assessed using phylogenetic analyses as in Sekigami *et al.* [62], based on *Hox* tree topology among the tunicates *Ciona robusta* and *Halocytnthia roretzi*, the cephalochordate *Branchiostoma lanceaolatum* and three vertebrate species. The names of the *B. schlosseri Hox* genes were assigned based on their proximity to the ones of *C. robusta* (Figure S14). However, most branches had low bootstrap support, and therefore including more tunicates as well as vertebrate species will be necessary to resolve the complex evolution of the *Hox* gene cluster across tunicates [61].

Although *Hox* genes are colinear between cephalochordates and vertebrates, it is not the case for tunicates [63]. In the tunicate species studied thus far, *Hox* clusters exhibit divergences in terms of colinearity and synteny relative to the ancestral chordate cluster [61]. In contrast to previous data [28, 64], our new assembly revealed that *B. schlosseri*'s *Hox* genes are not scattered. Instead, eight of them were clustered on the second largest scaffold (Bs2), whereas two other ones are found on the 15th largest scaffold (Bs15). Comparison with two tunicate ascidians, belonging to the same (*H. roretzi* [62]) and a different (*C. robusta* [52]) order, revealed partially conserved synteny as well as inversions and transpositions across the three species (Figure 8). These observations agree with the general trend of synteny conservation despite loss of colinearity observed for CLGs [55] and are also consistent with the phylogenetic relationships among the species sequenced [2, 54].

Conclusion

Tunicate genomes are known for their rapid evolution, featuring high rates of molecular divergence, extensive genomic rearrangements, and generally remarkably compact compared to vertebrates, though genome size varies among tunicate species [65]. Additionally, while some tunicates exhibit high levels of repetitive elements, others show moderate repeat content [64], [66]. Despite these variations, tunicate genomes share conserved non-coding elements, reflecting deep regulatory constraints within this diverse subphylum [67]. Although solitary tunicates such as *Ciona* and *Oikopleura*, along with other species, have been instrumental in shaping our understanding of tunicate genomes, colonial tunicates remain relatively understudied at the genomic level. Colonial species also introduce unique biological questions related to allorecognition, asexual reproduction, and whole-body regeneration. As a widely used model for colonial tunicates, *B. schlosseri* provides an essential reference for studying these processes, making a high-quality genome assembly particularly valuable. Comparative synteny analyses highlight both conserved and highly rearranged genomic features across tunicates, reinforcing the notion of their exceptional genomic plasticity. By making this resource available, we aim to facilitate future research into the evolutionary and functional ge-

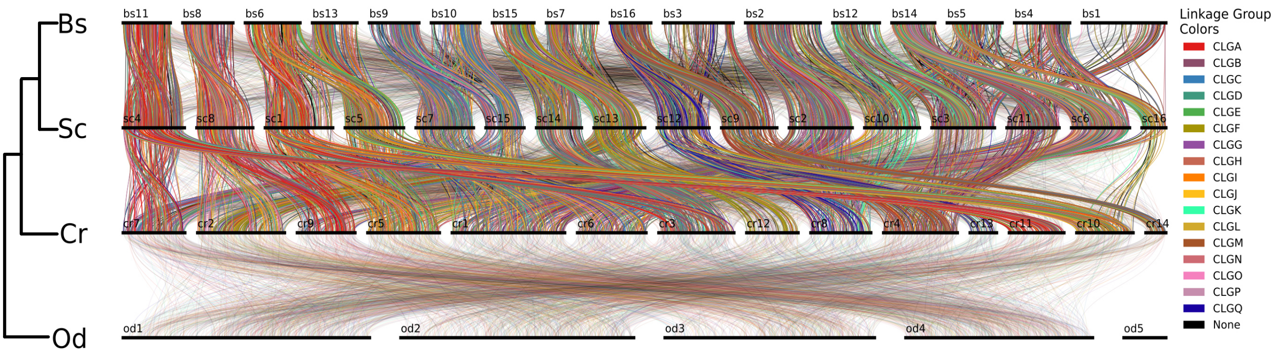

Figure 7. Synteny analyses using chordate linkage groups (CLGs) [55, 57] between *Botryllus schlosseri* (Bs), *Styela clava* (Sc), *Ciona robusta* (Cr) and *Oikopleura dioica* (Od).

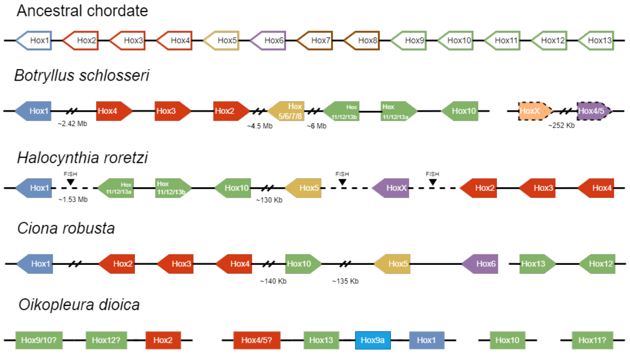

Figure 8. Representation of the *Hox* genes retrieved in the new assembly of *B. schlosseri* compared to the supposed original single Hox cluster of the chordate ancestor and other tunicates. Linked genes (present on the same scaffold) are connected by a solid line while a dashed line is used when the linkage has been deduced with another method. When known, the transcription orientation is given by an arrow-shaped rectangle, which is surrounded by a dashed line when the Hox gene was retrieved with low confidence.

nomics of chordates, also highlighting unique adaptations that define tunicate biology.

Methods

Sampling, DNA isolation, and sequencing

Isogenic colonies of *Botryllus schlosseri* were raised on glass slides in the marine-culture system described in Langenbacher *et al.* [21]. Genomic DNA was extracted from the colony labeled E* using Qiagen's MagAttract HMW DNA Kit (67563). Libraries were prepared and sequencing was performed at Novogene (Cambridge, UK) for Illumina 2x150 bp paired-end (PE) reads, at the Leiden Genome Technology Center (Leiden, Netherlands) for HiFi PacBio long reads and at UCAGenomix (Valbonne, France) for Oxford Nanopore (ONT) long reads (on a FLO-PRO002 flow cell with R9.4.1 pore proteins, using the SQK-LSK109 ligation sequencing kit). Nanopore base calling was performed using Guppy v3.2.10. A Hi-C library was prepared using the Arima High Coverage HiC Kit (A410110) followed by the Arima HiC+ Kit (A510008, A303011) and sequenced using Illumina (2x150 bp).

RNA-seq data

Illumina PE RNA-seq reads were retrieved from the published datasets of Rodriguez *et al.* [23] and Ricci *et al.* [25, 8], for a total of about 239 Gbp.

Data preprocessing

PacBio HiFi reads were processed with HiFiAdapterFilt v2.0.1 [68] to remove adapter sequences, while Porechop v0.2.4 (`https://github.com/rrwick/Porechop`) was used to trim basic adapters from ONT reads. For Illumina reads, quality trimming and adapter clipping were performed using Trimmomatic v0.39 [69] while quality check, prior to and after trimming, was done using FastQC v0.11.5 [70].

Genome size estimation

The genome size of colony E* was measured using the improved Feulgen protocol of M.Tawfeeq *et al.* (preprint in prep.). In brief, the protocol steps included: chopping the tissue into tiny pieces using a sterilized razor blade with a few drops of 40% glacial acetic then leaving it for 48 hours in the dark; immersing the processed slides into the fixation reagent (85:10:5 volumes of methanol:formaldehyde:acetic acid); then hydrolysing them (using hydrochloric acid) and staining them (using Schiff's reagent). We used three standards of known C-values: *Periplaneta americana* (3.41 pg) [71], *Lasius niger* (0.33 pg) (JF Flot, unpublished data), and *Ambystoma mexicanum* (32 pg) [72]. A digital camera (5 megapixels) mounted on a compound microscope was used for imaging the slides, and nuclei measurements were performed using ImageJ [73].

A genome size estimation based on the k-mer spectrum of the Illumina reads was also performed using KMC v3.2.1 [74] and the GenomeScope2.0 [75] web server, with a k-mer size of 21 and maximum k-mer counts of 10,000 and 10,000,000.

Haploid genome assembly

First, the PacBio HiFi reads were assembled into contigs using hifiasm v0.19.5-r592 [31] with the haplotype purging option disabled (option -l0 with hifiasm in HiFi-only Assembly mode). Second, uncollapsed haplotypes were purged using multiple rounds of HapLoMerger2 (release 20180603) [76] until the BUSCO duplication score stabilized. Third, non-metazoan contigs were identified and removed from the assemblies using BlobToolKit v4.1.5 [77]. To this aim, contigs were aligned to the NCBI nucleotide database (accessed 2023 March 18) using BLAST+ [78] with the blastn command, and also to the UniProt reference proteome database (accessed in 2023 March 23) using DIAMOND v2.1.6 [79]; contig HiFi coverage depth was computed using minimap2 v2.24-r1122 [80]. Using the "bestsumorder" rule of BlobToolKit, only the contigs assigned to the taxon "Chordata" or without a match ("no-hit") were kept. Finally, a BLASTN search for fragments of the mitochondrial genome among the contigs was performed using the published complete mitochondrial genome of *B. schlosseri* (RefSeq NC_021463.1) [28]. To scaffold the assemblies, PacBio HiFi and Illumina reads were first mapped to the assemblies with minimap2 and putative

misjoined regions were identified and automatically split using CRAQ v1.0.9 [33] with default parameters except for the addition of –break. Hi-C reads were subsequently mapped to the output of CRAQ using the Arima Genomics mapping pipeline script arima_mapping_pipeline.sh [81] (https://github.com/ArimaGenomics/mapping_pipeline), and YaHS v1.2 [34] was run with default parameters to scaffold the assemblies. CRAQ was then applied to the results, and finally the scaffolds were manually curated using PretextMap v0.1.9 [82] and PretextView v0.2.5 [83]. Metrics for the assemblies were computed with SeqKit v2.3.0 [84] (parameter stats -a). The quality and completeness were checked using KAT v2.4.2 [85] on k-mers from both PacBio HiFi and Illumina reads, and BUSCO v5.4.4 [86] (using the -m genome mode) with the metazoa_odb10 dataset.

Haplotype-resolved assembly

Two haplotype-resolved assemblies (haplotype 1 and haplotype 2) were generated using hifiasm in Hi-C Integrated Assembly mode, which directly integrates Hi-C reads. To refine the assemblies, uncollapsed sequences were purged for haplotype 1 using purge_dups [87], and BlobToolKit was employed, as with the haploid assembly, to filter out contamination, resulting in contig-level assemblies (see Figures S7 and S4). The scaffolding process for haplotype 1 and haplotype 2 followed the same method as for the haploid assembly, with the final scaffolds ordered based on alignment to the haploid assembly rather than by descending size (see Figure S10).

Genome annotation

For all the assemblies, repetitive elements were identified using RepeatModeler and RepeatMasker pipeline. A *de novo* repeat library was generated using RepeatModeler2 v2.0.3 [88] and used as input for RepeatMasker v4.0.6 [89] to detect, classify and soft-mask repeats in the genomic sequences. RNA-seq reads were aligned to the soft-masked assemblies using STAR v2.7.10b (default options) [90]. Based on the aligned transcripts, a list of proteins from OrthoDB v11 [91] for Metazoa (https://bioinf.uni-greifswald.de/bioinf/partitioned_odb11/) as extrinsic evidence and the soft-masked assemblies, gene prediction and annotation were done using the BRAKER3 v3.06 pipeline for RNA-Seq and protein data without training or gene prediction with untranslated regions (UTRs) parameters [92, 93, 94, 95, 96, 97, 98, 99, 100, 101, 102, 103, 104, 43]. A refinement of the initial BRAKER3 structural annotation and the addition of UTRs were then performed with an implementation the PASA pipeline v2.4.1 [44] in conjugation with EVidenceModeler (EVM) v2.1.0 [45]. A third of the RNA-seq reads of the Rodriguez *et al.* (2014) transcriptome [23] were aligned again to the assemblies and their BRAKER3 annotation using STAR v2.7.10b (MAX_INTRON_SIZE=20000) [90] and assembled with StringTie v2.2.1 [105] using the BRAKER3 annotation as a reference. PASA alignment assembly step was then run as described on the Github Wiki with the transcripts assembled by StringTie and independently with Trinity assemblies of the Rodriguez *et al.* (2014) RNAseq [23], Ricci *et al.* (2016) RNAseq [25] and Ricci *et al.* (2022) RNAseq [8]. TransDecoder [106] was run within PASA to identify coding sequences within the assembled transcripts. A consensus annotation of coding sequences (CDSs) was found by EVM by leveraging both the transcripts and coding sequences identified for each RNAseq by PASA (evidence weights: 1 for BRAKER3 input, 5 for PASA transcripts and TransDecoder CDSs). The gene models were refined, with addition of the UTRs and isoforms by running PASA genome annotation step sequentially with each previously generated PASA database (using EVM output as the first reference, then the output of the previous PASA genome annotation run). Functional annotation was performed starting from the structural annotation obtained with BRAKER3-PASA pipeline. Eggnog-mapper [46, 107] pipeline combined with Interproscan [47, 48] was used for orthology-based annotation (nr, KEGG, GO terms) and for protein domains prediction respectively. Both approaches were used as input for the Funannotate v1.8.15 pipeline [108], yielding a gff3 and a Genbank file with functional annotations (available upon request).

Comparative genomics analyses

The genome assemblies and annotations for the comparison of the haploid assembly with other tunicate species were retrieved from ANISEED [109] for *Botrylloides leachii*, *Ciona robusta*, and for the first assembly of *Botryllus schlosseri*, while *Oikopleura dioica* originates from [53], *Salpa thompsoni* from [110] and *Styela clava* from [51]. Macrosynteny analyses were performed using the odp tool [56]. For each species, analyses were based on the longest protein isoforms generated from their annotation file using AGAT's [111] scripts agat_sp_keep_longest_isoform.pl and agat_sp_extract_sequences.pl -p.

Phylogenetic analyses

COI fragments were retrieved from [42], aligned with MUSCLE [112]. A maximum-likelihood tree was generated using Mega5 [113] with the model HKY+I+G followed by 1000 bootstrap replicates. Phylogenetic analyses of *Botryllus schlosseri Hox* genes were performed using sequences retrieved from Sekigami *et al.* [62]. First, the sequences were aligned using MUSCLE [112] as implemented in AliView [114], then IQ-TREE 2 [115] was used to build a maximum-likelihood phylogeny with the best-fit model JTT+R6 [116, 117] selected by ModelFinder [118] following the Bayesian information criterion [119] and with 10,000 ultrafast bootstrap replicates [120].

Data availability

All sequencing data and assemblies generated in this study have been deposited in the NCBI BioProject database under the accession number PRJNA1225683 . The dataset is fully accessible to the research community for further analysis and can be retrieved from NCBI BioProject upon publication. The data sets supporting the results of this article are available in the Zenodo repository,[available upon publication] .

Declarations

Consent for publication

Not applicable.

Competing Interests

The author(s) declare that they have no competing interests.

Funding

This work was supported by ANR (ANR-14-CE02-0019-01 and ANR-24-CE02-2277), INSB-DBM and Sorbonne University AAP Emergence 2021 to ST and FAPESP 15/50164-5 & 19/06927-5 to FDB.

Author's Contributions

ODT carried out the majority of the assembly and analyses. ST, ODT, and JFF conceived the project and drafted the manuscript with the contribution of ML. MMT conducted the Feulgen analyses. SB

assisted with the initial stages of the assembly and provided part of the HiFi dataset. ML and PD handled the annotation and contributed to the analyses. AA, FDB, and RF provided valuable technical and scientific insights. ST and JFF supervised the research. All authors reviewed and approved the final version of the manuscript.

Acknowledgements

We would like to thank EMBRC-France and in particular Laurent Gilletta for isolating isogenic colonies and maintaining the aquaculture system. We would also like to thank the Next Generation Sequencing Platform of the University of Bern (Switzerland) for providing part of the HiFi sequencing. We thank Aaron Reinke for pointing out the presence of macrosporidia sequences in the BloobToolKit analyses, Vitoria Tobias Santos for filtering part of the RNAseq dataset used for the annotation, and Lino Ometto for useful scientific exchange.

References

1. Delsuc F, Brinkmann H, Chourrout D, Philippe H. Tunicates and not cephalochordates are the closest living relatives of vertebrates. Nature 2006;439(7079):965–968.
2. Alié A, Hiebert LS, Scelzo M, Tiozzo S. The eventful history of nonembryonic development in tunicates. Journal of Experimental Zoology Part B: Molecular and Developmental Evolution 2020;.
3. Stolfi A, Brown FD. Tunicata. In: Wanninger A, editor. Evolutionary Developmental Biology of Invertebrates 6: Deuterostomia Vienna: Springer; 2015.p. 135–204.
4. Hiebert LS, Simpson C, Tiozzo S. Coloniality, clonality, and modularity in animals: The elephant in the room. Journal of Experimental Zoology Part B: Molecular and Developmental Evolution 2021;336(3):198–211.
5. Manni L, Gasparini F, Hotta K, Ishizuka KJ, Ricci L, Tiozzo S, et al. Ontology for the asexual development and anatomy of the colonial chordate *Botryllus schlosseri*. PLoS ONE 2014;9(5):e96434.
6. Sabbadin A, Zaniolo G, Majone F. Determination of polarity and bilateral asymmetry in palleal and vascular buds of the ascidian *Botryllus schlosseri*. Developmental Biology 1975;46(1):79–87.
7. Nourizadeh S, Kassmer S, Rodriguez D, Hiebert LS, De Tomaso AW. Whole body regeneration and developmental competition in two botryllid ascidians. EvoDevo 2021-12-15;12(1):15.
8. Ricci L, Salmon B, Olivier C, Andreoni-Pham R, Chaurasia A, Alié A, et al. The onset of whole-body regeneration in *Botryllus schlosseri*: morphological and molecular characterization. Frontiers in Cell and Developmental Biology 2022-02-14;0:173.
9. Laird DJ, De Tomaso AW, Weissman IL. Stem cells are units of natural selection in a colonial ascidian. Cell 2005;123(7):1351–1360.
10. Brown FD, Tiozzo S, Roux MM, Ishizuka K, Swalla BJ, De Tomaso AW. Early lineage specification of long-lived germline precursors in the colonial ascidian *Botryllus schlosseri*. Development 2009;136(20):3485–3494.
11. Laird DJ, De Tomaso AW. Predatory stem cells in the non-zebrafish chordate, *Botryllus schlosseri*. Zebrafish 2005;1(4):357–361.
12. Pancer Z, Gershon H, Rinkevich B. Coexistence and possible parasitism of somatic and germ cell lines in chimeras of the colonial urochordate *Botryllus schlosseri*. The Biological Bulletin 1995;189(2):106–112.
13. Stoner DS, Weissman IL. Somatic and germ cell parasitism in a colonial ascidian: possible role for a highly polymorphic allorecognition system. Proceedings of the National Academy of Sciences of the United States of America 1996;93(26):15254–15259.
14. Manni L, Anselmi C, Cima F, Gasparini F, Voskoboynik A, Martini M, et al. Sixty years of experimental studies on the blastogenesis of the colonial tunicate *Botryllus schlosseri*. Developmental Biology 2019;448(2):293–308.
15. Kassmer SH, Rodriguez D, De Tomaso AW. Colonial ascidians as model organisms for the study of germ cells, fertility, whole body regeneration, vascular biology and aging. Current Opinion in Genetics & Development 2016;39:101–106.
16. Taketa DA, De Tomaso AW. *Botryllus schlosseri* allorecognition: tackling the enigma. Developmental & Comparative Immunology 2015;48(1):254–265.
17. Nydam ML. Evolution of allorecognition in the tunicata. Biology 2020-06-01;9(6):1–13.
18. Epelbaum A, Therriault TW, Paulson A, Pearce CM. Botryllid tunicates: Culture techniques and experimental procedures. Aquatic Invasions 2009;4(1):111–120.
19. Gasparini F, Manni L, Cima F, Zaniolo G, Burighel P, Caicci F, et al. Sexual and asexual reproduction in the colonial ascidian *Botryllus schlosseri*. Genesis 2015;53(1):105–120.
20. Wawrzyniak MK, Matas Serrato LA, Blanchoud S. Long-term monitoring data logs of a recirculating artificial seawater based colonial ascidian aquaculture. Data in Brief 2021-10;38:107372.
21. Langenbacher AD, Rodriguez D, Di Maio A, De Tomaso AW. Whole-mount fluorescent *in situ* hybridization staining of the colonial tunicate *B otryllus schlosseri*. genesis 2015;53(1):194–201.
22. Manni L, Zaniolo G, Cima F, Burighel P, Ballarin L. *Botryllus schlosseri*: A model ascidian for the study of asexual reproduction. Developmental Dynamics 2007;236(2):335–352.
23. Rodriguez D, Sanders EN, Farell K, Langenbacher AD, Taketa DA, Hopper MR, et al. Analysis of the basal chordate *Botryllus schlosseri* reveals a set of genes associated with fertility. BMC genomics 2014;15(1):1183.
24. Campagna D, Gasparini F, Franchi N, Vitulo N, Ballin F, Manni L, et al. Transcriptome dynamics in the asexual cycle of the chordate *Botryllus schlosseri*. BMC Genomics 2016;17(1):275.
25. Ricci L, Chaurasia A, Lapébie P, Dru P, Helm RR, Copley RR, et al. Identification of differentially expressed genes from multipotent epithelia at the onset of an asexual development. Scientific Reports 2016 Jun;6:27357.
26. Rosental B, Kowarsky M, Seita J, Corey DM, Ishizuka KJ, Palmeri KJ, et al. Complex mammalian-like haematopoietic system found in a colonial chordate. Nature 2018;564(7736):425–429.
27. Kowarsky M, Anselmi C, Hotta K, Burighel P, Zaniolo G, Caicci F, et al. Sexual and asexual development: two distinct programs producing the same tunicate. Cell Reports 2021;34(4):108681.
28. Voskoboynik A, Neff NF, Sahoo D, Newman AM, Pushkarev D, Koh W, et al. The genome sequence of the colonial chordate, *Botryllus schlosseri*. eLife 2013;2:e00569.
29. Lawniczak MKN, Durbin R, Flicek P, Lindblad-Toh K, Wei X, Archibald JM, et al. Standards recommendations for the Earth BioGenome Project. Proceedings of the National Academy of Sciences 2022;119(4):e2115639118.
30. De Tomaso AW, Saito Y, Ishizuka KJ, Palmeri KJ, Weissman IL. Mapping the genome of a model protochordate. I. A low resolution genetic map encompassing the fusion/histocompatibility (Fu/HC) locus of *Botryllus schlosseri*. Genetics 1998;149(1):277–287.
31. Cheng H, Concepcion GT, Feng X, Zhang H, Li H. Haplotype-resolved de novo assembly using phased assembly graphs with hifiasm. Nature Methods 2021;18(2):170–175.
32. Bojko J, Reinke AW, Stentiford GD, Williams B, Rogers MSJ, Bass D. Microsporidia: a new taxonomic, evolutionary, and ecological synthesis. Trends in Parasitology 2022;38(8):642–

659.
33. Li K, Xu P, Wang J, Yi X, Jiao Y. Identification of errors in draft genome assemblies at single-nucleotide resolution for quality assessment and improvement. Nature Communications 2023;14(1):6556.
34. Zhou C, McCarthy SA, Durbin R. YaHS: yet another Hi-C scaffolding tool. Bioinformatics 2023;39(1):btac808.
35. Zeng X, Yi Z, Zhang X, Du Y, Li Y, Zhou Z, et al. Chromosome-level scaffolding of haplotype-resolved assemblies using Hi-C data without reference genomes. Nature Plants 2024;10(8):1184–1200.
36. Bandi V, Gutwin C, Siri JN, Neufeld E, Sharpe A, Parkin I. Visualization tools for genomic conservation. In: Edwards D, editor. Plant Bioinformatics: Methods and Protocols New York, NY: Springer US; 2022.p. 285–308.
37. Wang Y, Tang H, DeBarry JD, Tan X, Li J, Wang X, et al. MCScanX: a toolkit for detection and evolutionary analysis of gene synteny and collinearity. Nucleic Acids Research 2012;40(7):e49.
38. Simão FA, Waterhouse RM, Ioannidis P, Kriventseva EV, Zdobnov EM. BUSCO: assessing genome assembly and annotation completeness with single-copy orthologs. Bioinformatics 2015;31(19):3210–3212.
39. Guiglielmoni N, Houtain A, Derzelle A, Van Doninck K, Flot JF. Overcoming uncollapsed haplotypes in long-read assemblies of non-model organisms. BMC Bioinformatics 2021;22(1):303.
40. Simion P, Narayan J, Houtain A, Derzelle A, Baudry L, Nicolas E, et al. Chromosome-level genome assembly reveals homologous chromosomes and recombination in asexual rotifer *Adineta vaga*. Science Advances 2021;7(41):eabg4216.
41. Dierckxsens N, Mardulyn P, Smits G. NOVOPlasty: de novo assembly of organelle genomes from whole genome data. Nucleic Acids Research 2017;45(4):e18.
42. Salonna M, Gasparini F, Huchon D, Montesanto F, Haddas-Sasson M, Ekins M, et al. An elongated COI fragment to discriminate botryllid species and as an improved ascidian DNA barcode. Scientific Reports 2021;11(1):4078.
43. Gabriel L, Brůna T, Hoff KJ, Ebel M, Lomsadze A, Borodovsky M, et al. BRAKER3: Fully automated genome annotation using RNA-Seq and protein evidence with GeneMark-ETP, AUGUSTUS and TSEBRA. bioRxiv 2023;p. 2023.06.10.544449.
44. Haas BJ. Improving the *Arabidopsis* genome annotation using maximal transcript alignment assemblies. Nucleic Acids Research 2003;31(19):5654–5666.
45. Haas BJ, Salzberg SL, Zhu W, Pertea M, Allen JE, Orvis J, et al. Automated eukaryotic gene structure annotation using EVidenceModeler and the Program to Assemble Spliced Alignments. Genome Biology 2008;9(1):R7.
46. Cantalapiedra CP, Hernández-Plaza A, Letunic I, Bork P, Huerta-Cepas J. eggNOG-mapper v2: functional annotation, orthology assignments, and domain prediction at the metagenomic scale. Molecular Biology and Evolution 2021;38(12):5825–5829.
47. Blum M, Chang HY, Chuguransky S, Grego T, Kandasaamy S, Mitchell A, et al. The InterPro protein families and domains database: 20 years on. Nucleic Acids Research 2021;49(D1):D344–D354.
48. Jones P, Binns D, Chang HY, Fraser M, Li W, McAnulla C, et al. InterProScan 5: genome-scale protein function classification. Bioinformatics 2014;30(9):1236–1240.
49. Kanehisa M, Sato Y, Morishima K. BlastKOALA and GhostKOALA: KEGG tools for functional characterization of genome and metagenome sequences. Journal of Molecular Biology 2016;428(4):726–731.
50. Colombera D. The karyology of the colonial ascidian *Botryllus schlosseri* (Pallas). Caryologia 1969;22(4):339–349.
51. Wei J, Zhang J, Lu Q, Ren P, Guo X, Wang J, et al. Genomic basis of environmental adaptation in the leathery sea squirt (*Styela clava*). Molecular Ecology Resources 2020;20(5):1414–1431.
52. Satou Y, Nakamura R, Yu D, Yoshida R, Hamada M, Fujie M, et al. A nearly complete genome of *Ciona intestinalis* type A (*C. robusta*) reveals the contribution of inversion to chromosomal evolution in the genus *Ciona*. Genome Biology and Evolution 2019;11(11):3144–3157.
53. Bliznina A, Masunaga A, Mansfield MJ, Tan Y, Liu AW, West C, et al. Telomere-to-telomere assembly of the genome of an individual Oikopleura dioica from Okinawa using Nanopore-based sequencing. BMC Genomics 2021;22(1):222.
54. Delsuc F, Philippe H, Tsagkogeorga G, Simion P, Tilak MK, Turon X, et al. A phylogenomic framework and timescale for comparative studies of tunicates. BMC Biology 2018;16(1):1–14.
55. Simakov O, Marlétaz F, Yue JX, O'Connell B, Jenkins J, Brandt A, et al. Deeply conserved synteny resolves early events in vertebrate evolution. Nature Ecology & Evolution 2020-06;4(6):820–830.
56. Schultz DT, Haddock SHD, Bredeson JV, Green RE, Simakov O, Rokhsar DS. Ancient gene linkages support ctenophores as sister to other animals. Nature 2023;618(7963):110–117.
57. Simakov O, Bredeson J, Berkoff K, Marletaz F, Mitros T, Schultz DT, et al. Deeply conserved synteny and the evolution of metazoan chromosomes. Science Advances 2022;8(5):eabi5884.
58. Lewin TD, Liao IJY, Chen ME, Bishop JDD, Holland PWH, Luo YJ. Fusion, fission, and scrambling of the bilaterian genome in Bryozoa. Genome Research 2025;35(1):78–92.
59. Plessy C, Mansfield MJ, Bliznina A, Masunaga A, West C, Tan Y, et al. Extreme genome scrambling in marine planktonic *Oikopleura dioica* cryptic species. Genome Research 2024;.
60. Monteiro AS, Ferrier DEK. Hox genes are not always Colinear. International Journal of Biological Sciences 2006;p. 95–103.
61. DeBiasse MB, Colgan WN, Harris L, Davidson B, Ryan JF. Inferring tunicate relationships and the evolution of the tunicate Hox cluster with the genome of *Corella inflata*. Genome Biology and Evolution 2020;12(6):948–964.
62. Sekigami Y, Kobayashi T, Omi A, Nishitsuji K, Ikuta T, Fujiyama A, et al. Hox gene cluster of the ascidian, *Halocynthia roretzi*, reveals multiple ancient steps of cluster disintegration during ascidian evolution. Zoological Letters 2017;3:17.
63. Gaunt SJ. Seeking sense in the Hox gene cluster. Journal of Developmental Biology 2022;10(4):48.
64. Blanchoud S, Rutherford K, Zondag L, Gemmell NJ, Wilson MJ. De novo draft assembly of the *Botrylloides leachii* genome provides further insight into tunicate evolution. Scientific Reports 2018;8(1):5518.
65. Caputi L. Evolutionary Genomics of Tunicates. Science Reviews Biology 2024;3(2):22–32.
66. Berna L, Alvarez-Valin F. Evolutionary genomics of fast evolving tunicates. Genome Biology and Evolution 2014;6(7):1724–1738.
67. Sanges R, Hadzhiev Y, Gueroult-Bellone M, Roure A, Ferg M, Meola N, et al. Highly conserved elements discovered in vertebrates are present in non-syntenic loci of tunicates, act as enhancers and can be transcribed during development. Nucleic Acids Research 2013;41(6):3600–3618.
68. Sim SB, Corpuz RL, Simmonds TJ, Geib SM. HiFiAdapterFilt, a memory efficient read processing pipeline, prevents occurrence of adapter sequence in PacBio HiFi reads and their negative impacts on genome assembly. BMC Genomics 2022;23(1):157.
69. Bolger AM, Lohse M, Usadel B. Trimmomatic: a flexible trimmer for Illumina sequence data. Bioinformatics 2014;30(15):2114–2120.
70. Andrews S, FastQC: A quality control tool for high throughput sequence data. Available online at: http://www.bioinformatics.babraham.ac.uk/projects/fastqc/; 2010.

71. Wang L, Xiong Q, Saelim N, Wang L, Nong W, Wan ATY, et al. Genome assembly and annotation of *Periplaneta americana* reveal a comprehensive cockroach allergen profile. Allergy 2023;78(4):1088–1103.
72. Nowoshilow S, Schloissnig S, Fei JF, Dahl A, Pang AWC, Pippel M, et al. The axolotl genome and the evolution of key tissue formation regulators. Nature 2018;554(7690):50–55.
73. Schneider CA, Rasband WS, Eliceiri KW. NIH Image to ImageJ: 25 years of image analysis. Nature Methods 2012;9(7):671–675.
74. Kokot M, Długosz M, Deorowicz S. KMC 3: counting and manipulating k-mer statistics. Bioinformatics 2017;33(17):2759–2761.
75. Ranallo-Benavidez TR, Jaron KS, Schatz MC. GenomeScope 2.0 and Smudgeplot for reference-free profiling of polyploid genomes. Nature Communications 2020;11(1):1432.
76. Huang S, Kang M, Xu A. HaploMerger2: rebuilding both haploid sub-assemblies from high-heterozygosity diploid genome assembly. Bioinformatics 2017;33(16):2577–2579.
77. Challis R, Richards E, Rajan J, Cochrane G, Blaxter M. BlobToolKit – interactive quality assessment of genome assemblies. G3: Genes|Genomes|Genetics 2020;10(4):1361–1374.
78. Camacho C, Coulouris G, Avagyan V, Ma N, Papadopoulos J, Bealer K, et al. BLAST+: architecture and applications. BMC Bioinformatics 2009;10(1):421.
79. Buchfink B, Reuter K, Drost HG. Sensitive protein alignments at tree-of-life scale using DIAMOND. Nature Methods 2021;18(4):366–368.
80. Li H. New strategies to improve minimap2 alignment accuracy. Bioinformatics 2021;37(23):4572–4574.
81. Ghurye J, Rhie A, Walenz BP, Schmitt A, Selvaraj S, Pop M, et al. Integrating Hi-C links with assembly graphs for chromosome-scale assembly. PLOS Computational Biology 2019;15(8):e1007273.
82. Harry E, PretextMap (Paired REad TEXTure Mapper): Converts SAM formatted read pairs into genome contact maps. https://github.com/wtsi-hpag/PretextMap; 2023.
83. Harry E, PretextView (Paired REad TEXTure Viewer): A Desktop Application for Viewing Pretext Contact Maps. https://github.com/wtsi-hpag/PretextView; 2023.
84. Shen W, Le S, Li Y, Hu F. SeqKit: a cross-platform and ultrafast toolkit for FASTA/Q file manipulation. PLoS ONE 2016;11(10):e0163962.
85. Mapleson D, Garcia Accinelli G, Kettleborough G, Wright J, Clavijo BJ. KAT: a K-mer analysis toolkit to quality control NGS datasets and genome assemblies. Bioinformatics 2017;33(4):574–576.
86. Manni M, Berkeley MR, Seppey M, Simão FA, Zdobnov EM. BUSCO update: novel and streamlined workflows along with broader and deeper phylogenetic coverage for scoring of eukaryotic, prokaryotic, and viral genomes. Molecular Biology and Evolution 2021;38(10):4647–4654.
87. Guan D, McCarthy SA, Wood J, Howe K, Wang Y, Durbin R. Identifying and removing haplotypic duplication in primary genome assemblies. Bioinformatics 2020;36(9):2896–2898.
88. Flynn JM, Hubley R, Goubert C, Rosen J, Clark AG, Feschotte C, et al. RepeatModeler2 for automated genomic discovery of transposable element families. Proceedings of the National Academy of Sciences 2020;117(17):9451–9457.
89. Smit AFA, Hubley R, Green P, RepeatMasker Open-4.0. 2013-2015 <http://www.repeatmasker.org>;.
90. Dobin A, Davis CA, Schlesinger F, Drenkow J, Zaleski C, Jha S, et al. STAR: ultrafast universal RNA-seq aligner. Bioinformatics 2013;29(1):15–21.
91. Kuznetsov D, Tegenfeldt F, Manni M, Seppey M, Berkeley M, Kriventseva EV, et al. OrthoDB v11: annotation of orthologs in the widest sampling of organismal diversity. Nucleic Acids Research 2023;51(D1):D445–D451.
92. Lomsadze A. Gene identification in novel eukaryotic genomes by self-training algorithm. Nucleic Acids Research 2005;33(20):6494–6506.
93. Stanke M, Schöffmann O, Morgenstern B, Waack S. Gene prediction in eukaryotes with a generalized hidden Markov model that uses hints from external sources. BMC Bioinformatics 2006;7(1):62.
94. Lomsadze A, Burns PD, Borodovsky M. Integration of mapped RNA-Seq reads into automatic training of eukaryotic gene finding algorithm. Nucleic Acids Research 2014;42(15):e119–e119.
95. Gotoh O. A space-efficient and accurate method for mapping and aligning cDNA sequences onto genomic sequence. Nucleic Acids Research 2008;36(8):2630–2638.
96. Iwata H, Gotoh O. Benchmarking spliced alignment programs including Spaln2, an extended version of Spaln that incorporates additional species-specific features. Nucleic Acids Research 2012;40(20):e161–e161.
97. Buchfink B, Xie C, Huson DH. Fast and sensitive protein alignment using DIAMOND. Nature Methods 2015;12(1):59–60.
98. Brůna T, Lomsadze A, Borodovsky M. GeneMark-EP+: eukaryotic gene prediction with self-training in the space of genes and proteins. NAR Genomics and Bioinformatics 2020;2(2):lqaa026.
99. Pertea G, Pertea M. GFF Utilities: GffRead and GffCompare. F1000Research 2020;9:ISCB Comm J–304.
100. Kovaka S, Zimin AV, Pertea GM, Razaghi R, Salzberg SL, Pertea M. Transcriptome assembly from long-read RNA-seq alignments with StringTie2. Genome Biology 2019;20(1):278.
101. Stanke M, Diekhans M, Baertsch R, Haussler D. Using native and syntenically mapped cDNA alignments to improve de novo gene finding. Bioinformatics 2008;24(5):637–644.
102. Hoff KJ, Lomsadze A, Borodovsky M, Stanke M, Kollmar M. Whole-genome annotation with BRAKER. In: Gene prediction: methods and protocols No. 1962 in Methods in Molecular Biology, Springer; 2019.p. 65–95.
103. Hoff KJ, Lange S, Lomsadze A, Borodovsky M, Stanke M. BRAKER1: unsupervised RNA-Seq-based genome annotation with GeneMark-ET and AUGUSTUS. Bioinformatics 2016;32(5):767–769.
104. Brůna T, Hoff KJ, Lomsadze A, Stanke M, Borodovsky M. BRAKER2: automatic eukaryotic genome annotation with GeneMark-EP+ and AUGUSTUS supported by a protein database. NAR Genomics and Bioinformatics 2021;3(1):lqaa108.
105. Pertea M, Pertea GM, Antonescu CM, Chang TC, Mendell JT, Salzberg SL. StringTie enables improved reconstruction of a transcriptome from RNA-seq reads. Nature Biotechnology 2015;33(3):290–295.
106. Haas BJ, Transdecoder; 2025. http://www.ncbi.nlm.nih.gov/pmc/articles/PMC3875132/, accessed: 2024-12-12.
107. Huerta-Cepas J, Szklarczyk D, Heller D, Hernández-Plaza A, Forslund SK, Cook H, et al. eggNOG 5.0: a hierarchical, functionally and phylogenetically annotated orthology resource based on 5090 organisms and 2502 viruses. Nucleic Acids Research 2019;47(D1):D309–D314.
108. Palmer JM, Stajich JE, Funannotate; 2023. https://github.com/nextgenusfs/funannotate, original-date: 2015-12-18T20:21:15Z.
109. Dardaillon J, Dauga D, Simion P, Faure E, Onuma TA, DeBiasse MB, et al. ANISEED 2019: 4D exploration of genetic data for an extended range of tunicates. Nucleic Acids Research 2020;48(D1):D668–D675.
110. Castellano KR, Batta-Lona P, Bucklin A, O'Neill RJ. Salpa genome and developmental transcriptome analyses reveal molecular flexibility enabling reproductive success in a rapidly changing environment. Scientific Reports 2023;13(1):21056.

111. Dainat J, AGAT: Another Gff Analysis Toolkit to handle annotations in any GTF/GFF format. (Version v0.7.0). Zenodo. https://www.doi.org/10.5281/zenodo.3552717; 2024.
112. Edgar RC. MUSCLE: multiple sequence alignment with high accuracy and high throughput. Nucleic Acids Research 2004;32(5):1792–1797.
113. Tamura K, Peterson D, Peterson N, Stecher G, Nei M, Kumar S. MEGA5: Molecular Evolutionary Genetics Analysis Using Maximum Likelihood, Evolutionary Distance, and Maximum Parsimony Methods. Molecular Biology and Evolution 2011;28(10):2731–2739.
114. Larsson A. AliView: a fast and lightweight alignment viewer and editor for large datasets. Bioinformatics 2014;30(22):3276–3278.
115. Minh BQ, Schmidt HA, Chernomor O, Schrempf D, Woodhams MD, von Haeseler A, et al. IQ-TREE 2: new models and efficient methods for phylogenetic inference in the genomic era. Molecular Biology and Evolution 2020;37(5):1530–1534.
116. Jones DT, Taylor WR, Thornton JM. The rapid generation of mutation data matrices from protein sequences. Bioinformatics 1992;8(3):275–282.
117. Yang Z. A space-time process model for the evolution of DNA sequences. Genetics 1995;139(2):993–1005.
118. Kalyaanamoorthy S, Minh BQ, Wong TKF, von Haeseler A, Jermiin LS. ModelFinder: fast model selection for accurate phylogenetic estimates. Nature Methods 2017;14(6):587–589.
119. Schwarz G. Estimating the dimension of a model. The Annals of Statistics 1978;6(2):461–464.
120. Hoang DT, Chernomor O, von Haeseler A, Minh BQ, Vinh LS. UFBoot2: improving the ultrafast bootstrap approximation. Molecular Biology and Evolution 2018;35(2):518–522.
121. Cabanettes F, Klopp C. D-GENIES: dot plot large genomes in an interactive, efficient and simple way. PeerJ 2018;6:e4958.

Supplementary Figures

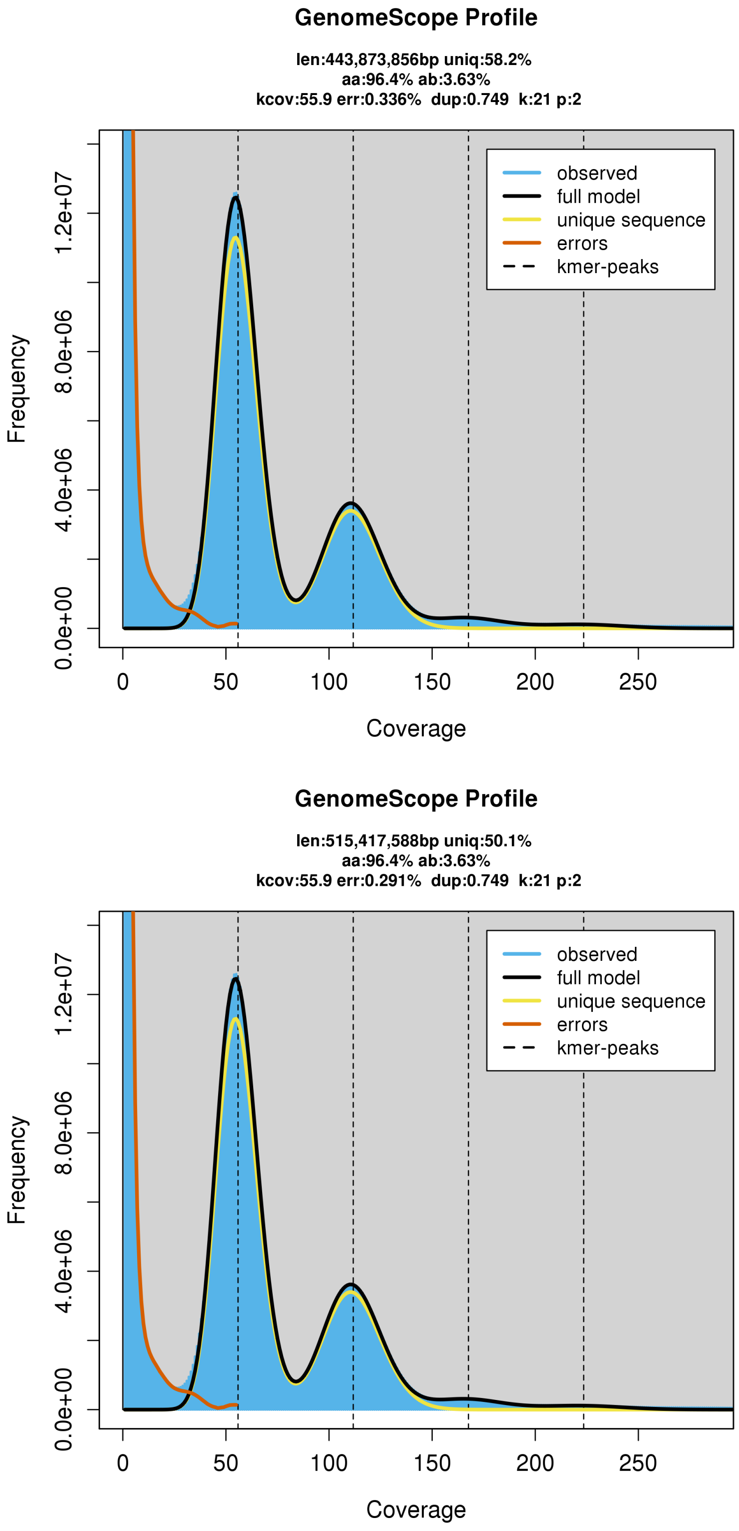

Figure S1. Genomescope2.0 results obtained with the Illumina reads and using 21-mers with a maximum counts of 10,000 (top) and 10,000,000 (bottom).

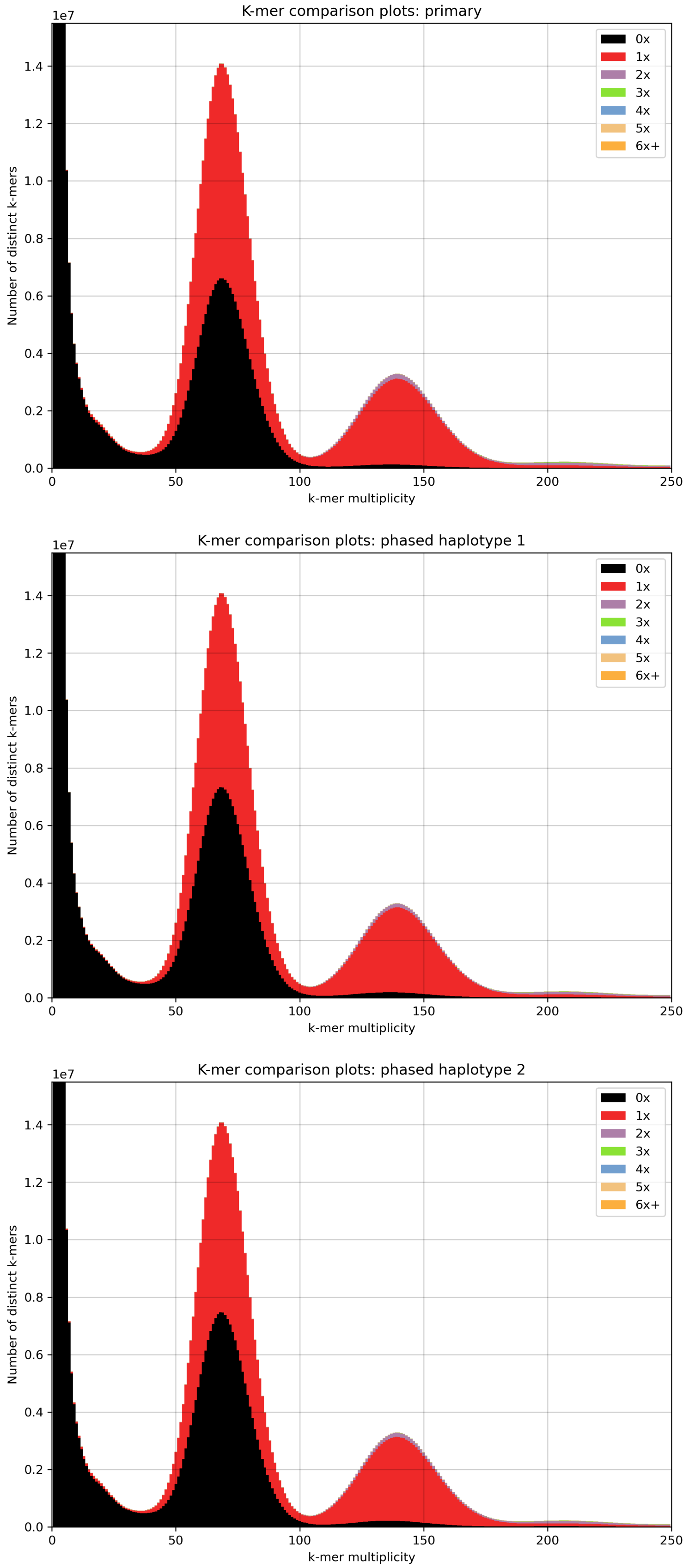

Figure S2. Output of the KAT comp tool comparing k-mers generated from a concatenation of the Illumina and HiFi reads to those generated from the haploid (top), haplotype 1 (middle) and haplotype 2 (bottom) assemblies of *B. schlosseri*. The k-mer completeness, based on the highest peak representing heterozygous regions, is respectively (from top to bottom) 53.03%, 47.94%, and 46.92%. A perfectly correct haploid representation should have a completeness of 50%.

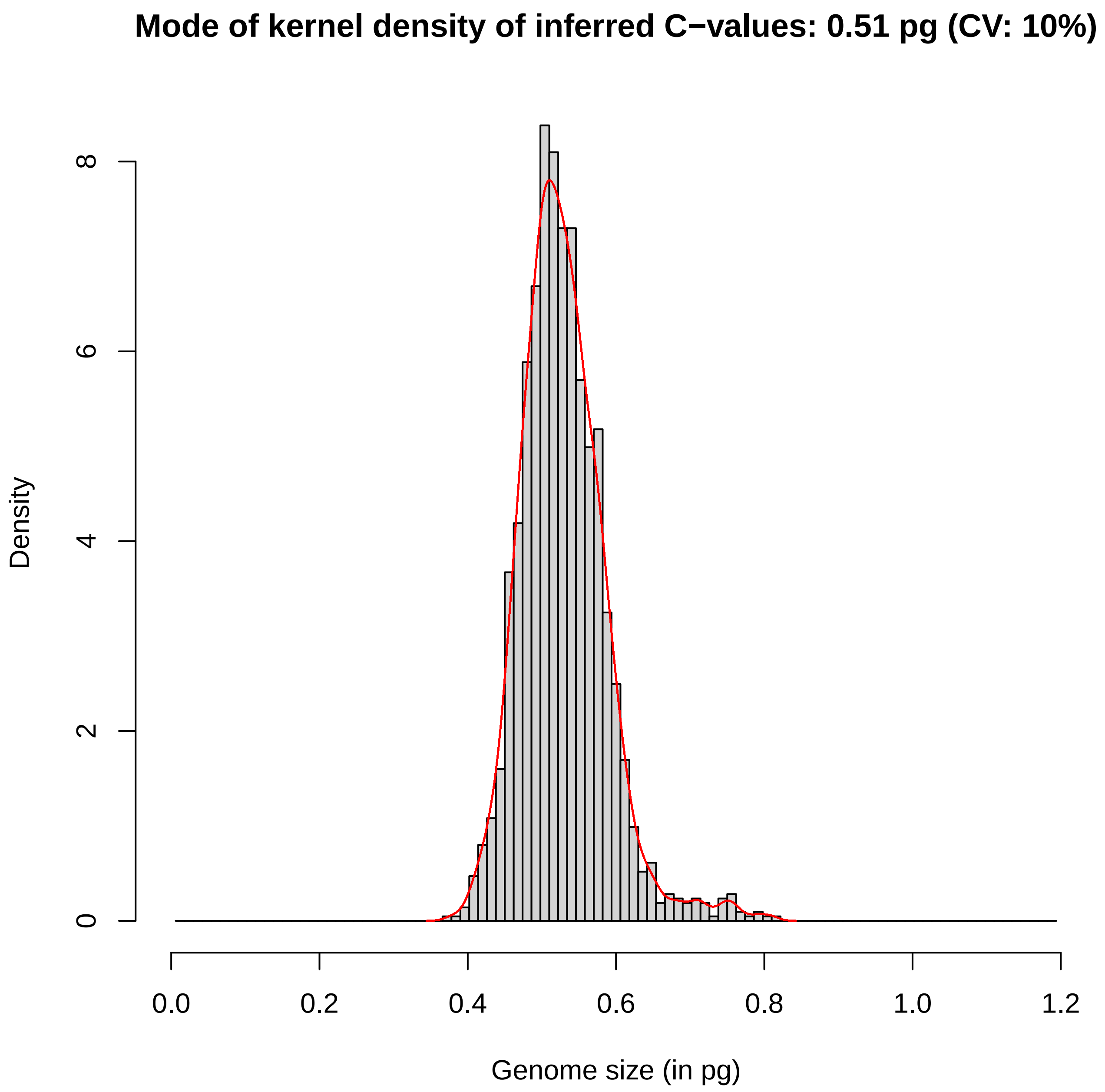

Figure S3. Genome size of *Botryllus schlosseri* measured using Feulgen microphotodensitometry.

Assembly			
	**Haploid**	**Haplotype 1**	**Haplotype 2**
Total length (Mbp) (Chr. only)	533 (513)	496 (480)	494 (464)
No. scaffolds	254	219	410
N50 (Mbp)	30 6	29	29
GC (%)	40.52	40.52	40.53
BUSCO	C:91.6%; S:90.7%; D:0.9% F:3.1%; M:5.3%	C:90.9%; S:89.9%; D:1.0% F:3.6%; M:5.5%	C:91.2%; S:88.7%; D:2.5% F:3.1%; M:5.7%
**Annotation**			
	**Haploid**	**Haplotype 1**	**Haplotype 2**
No. genes	22,275	21,802	21,831
No. mRNAs	30,813	30,298	30,361
BUSCO	C:92.4%; S:79.7%; D:12.7% F:1.8%; M:5.8%	C:91.6%; S:80.4%; D:11.2% F:1.9%; M:6.5%	C:92.1%; S:79.1%; D:13.0% F:1.6%; M:6.3%

Table S1. Metrics for the haploid, haplotype 1, and haplotype 2 assemblies.

Chromosome	Haploid	Haplotype 1	Haplotype 2
1	50,110	41,841 (83%)	28,053 (56%)
2	44,705	40,598 (91%)	45,038 (101%)
3	41,815	38,360 (92%)	29,155 (70%)
4	33,037	30,504 (92%)	32,607 (99%)
5	32,846	28,210 (86%)	28,942 (88%)
6	32,755	31,460 (96%)	32,176 (98%)
7	30,789	29,986 (97%)	29,040 (94%)
8	30,038	28,423 (95%)	28,982 (96%)
9	29,634	27,923 (94%)	30,153 (102%)
10	29,082	28,687 (99%)	27,860 (96%)
11	28,207	29,008 (103%)	27,153 (96%)
12	27,998	26,703 (95%)	26,494 (95%)
13	26,676	25,071 (94%)	25,027 (94%)
14	25,737	25,698 (100%)	25,542 (99%)
15	24,956	24,523 (98%)	23,565 (94%)
16	24,314	22,647 (93%)	23,761 (98%)

Table S2. Comparison of the putative chromosome sizes (in kbp) across the three different assemblies. The putative chromosomes correspond to the 16 longest scaffolds, ordered in descending size for the haploid assembly. For the haplotype 1 and haplotype 2 assemblies, the scaffold order is based on their alignment to the haploid assembly, with percentages in parentheses indicating their size relative to the reference haploid assembly.

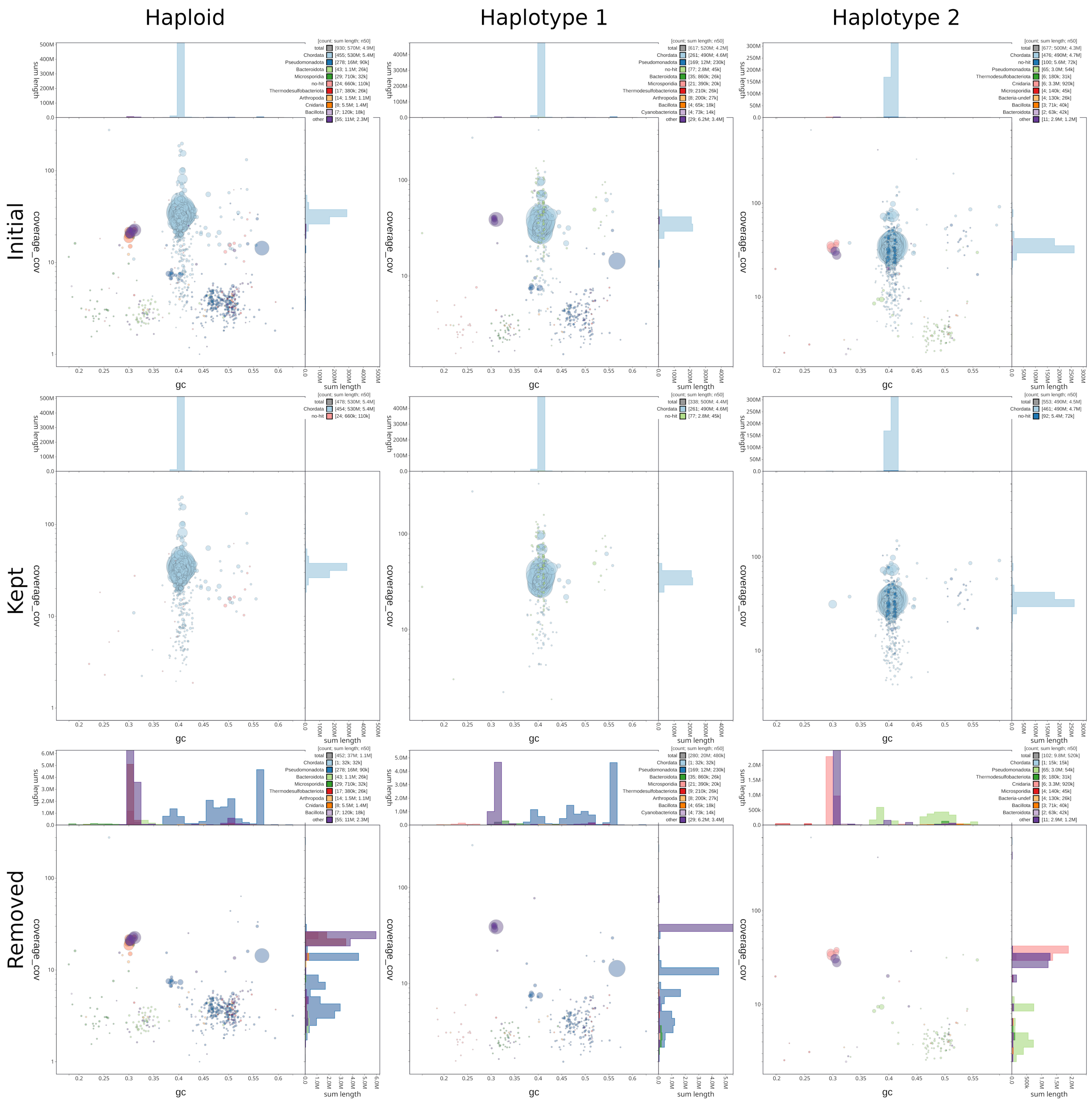

Figure S4. BlobPlots of the assemblies of *B. schlosseri*. **Initial** refers to results obtained before filtering out contamination. **Kept** represents the contigs retained in the assemblies before scaffolding, while **Removed** represents those discarded as contamination.

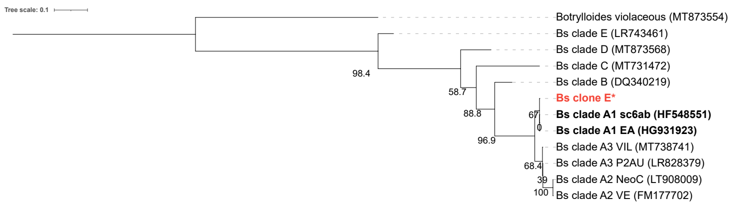

Figure S5. ML tree of *Botryllus schlosseri* clades and sub-clades reconstructed from COI alignment [42] Branches shows boostrap values. Accession ID between parenthesis.

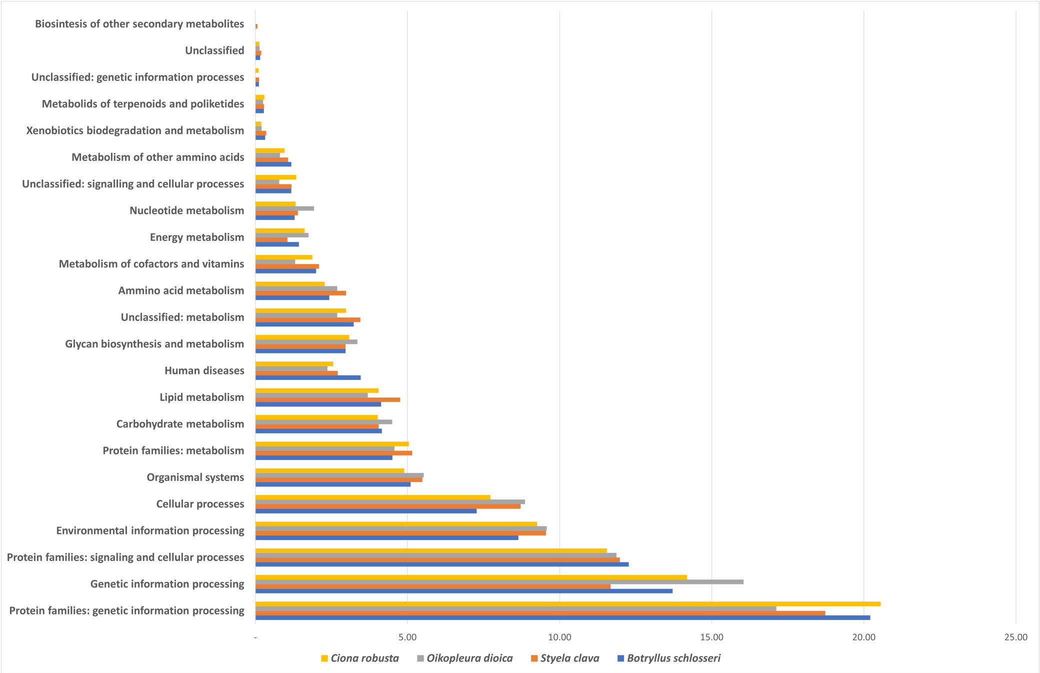

Figure S6. Comparison of the percentage of genes of *Botryllus schlosseri*, *Ciona robusta*, *Oikopleura dioica* and *Styela clava* assigned to different KEGG functional categories by BlastKOALA [49]

.

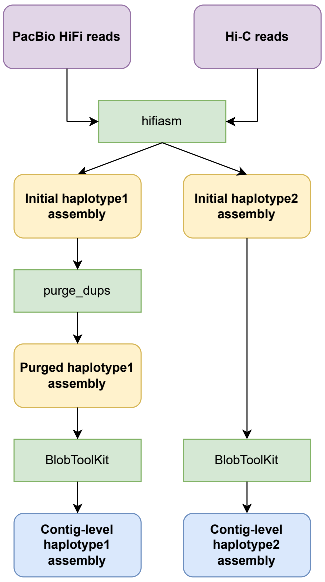

Figure S7. Assembly pipeline used to generate the contig-level assemblies of haplotype 1 and haplotype 2. The downstream steps (not shown) to produce scaffold-level assemblies are identical to those used for the haploid assembly.

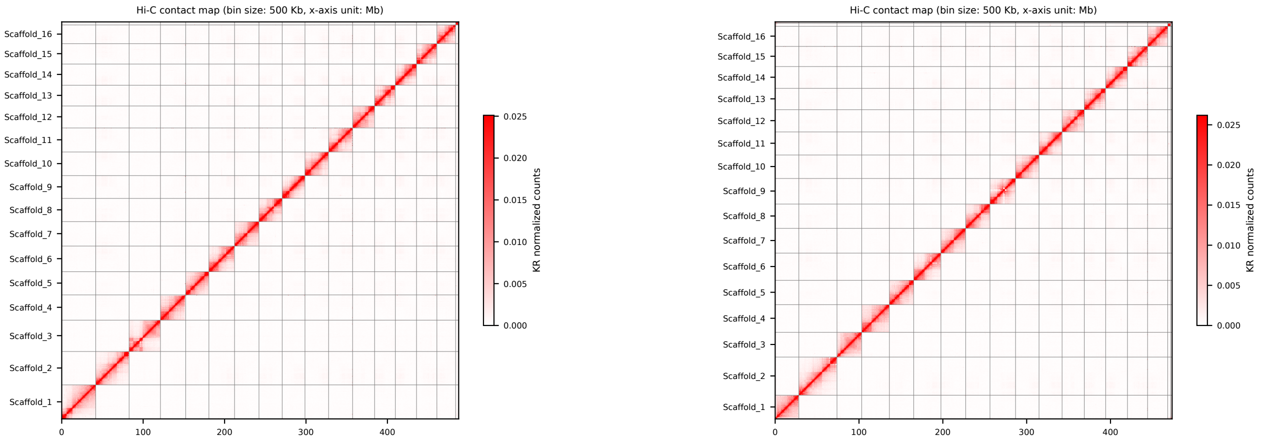

(a) Haplotype 1

(b) Haplotype 2

Figure S8. Hi-C heatmaps of the haplotype 1 (left) and haplotype 2 (right) assemblies showing sixteen chromosome-scale scaffolds for both.

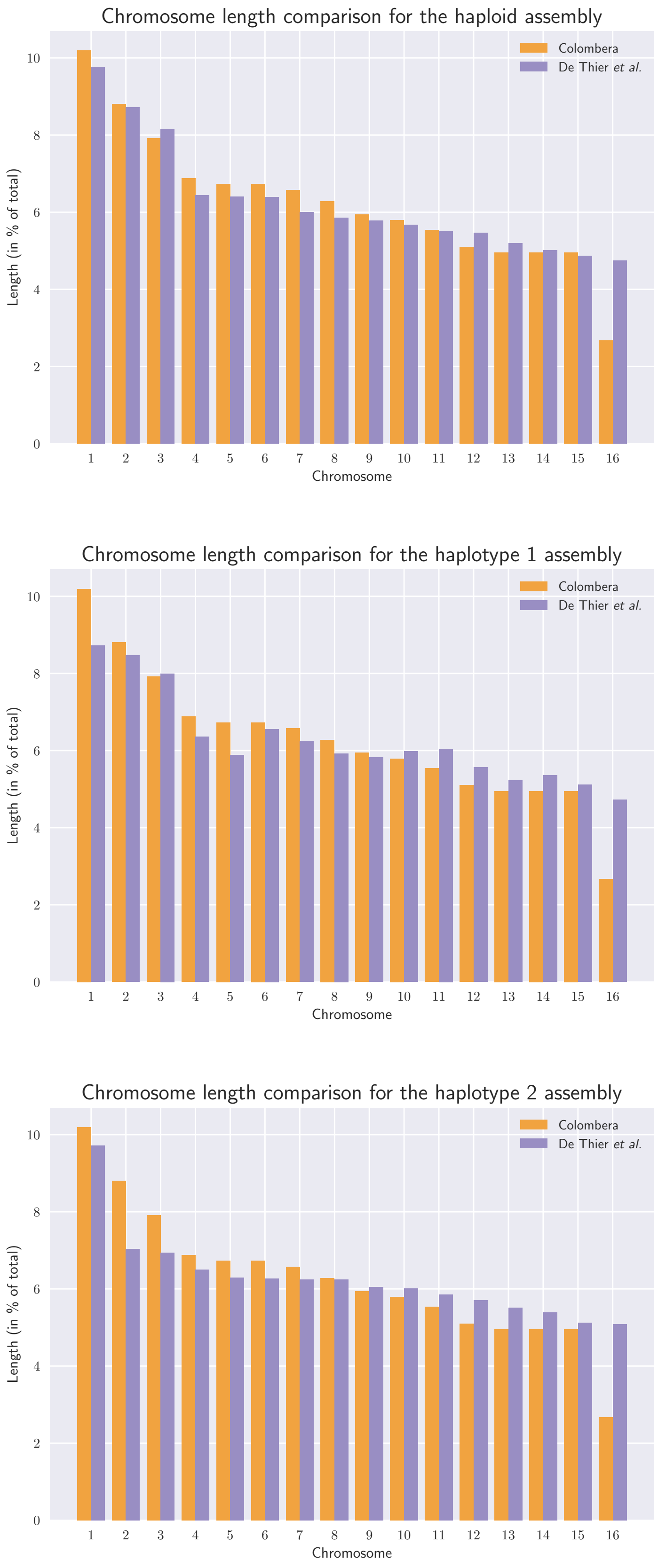

Figure S9. Comparisons between the 16 longest scaffolds from the haploid, haplotype 1, and haplotype 2 assemblies and the karyogram of Colombera [50]. The lengths of the bars were calculated as the proportion (in percentage) of each chromosome's length relative to the total genome length. The order of scaffolds for haplotype 1 and haplotype 2 is based on the sizes of the scaffolds in descending order, rather than their alignment to the haploid assembly.

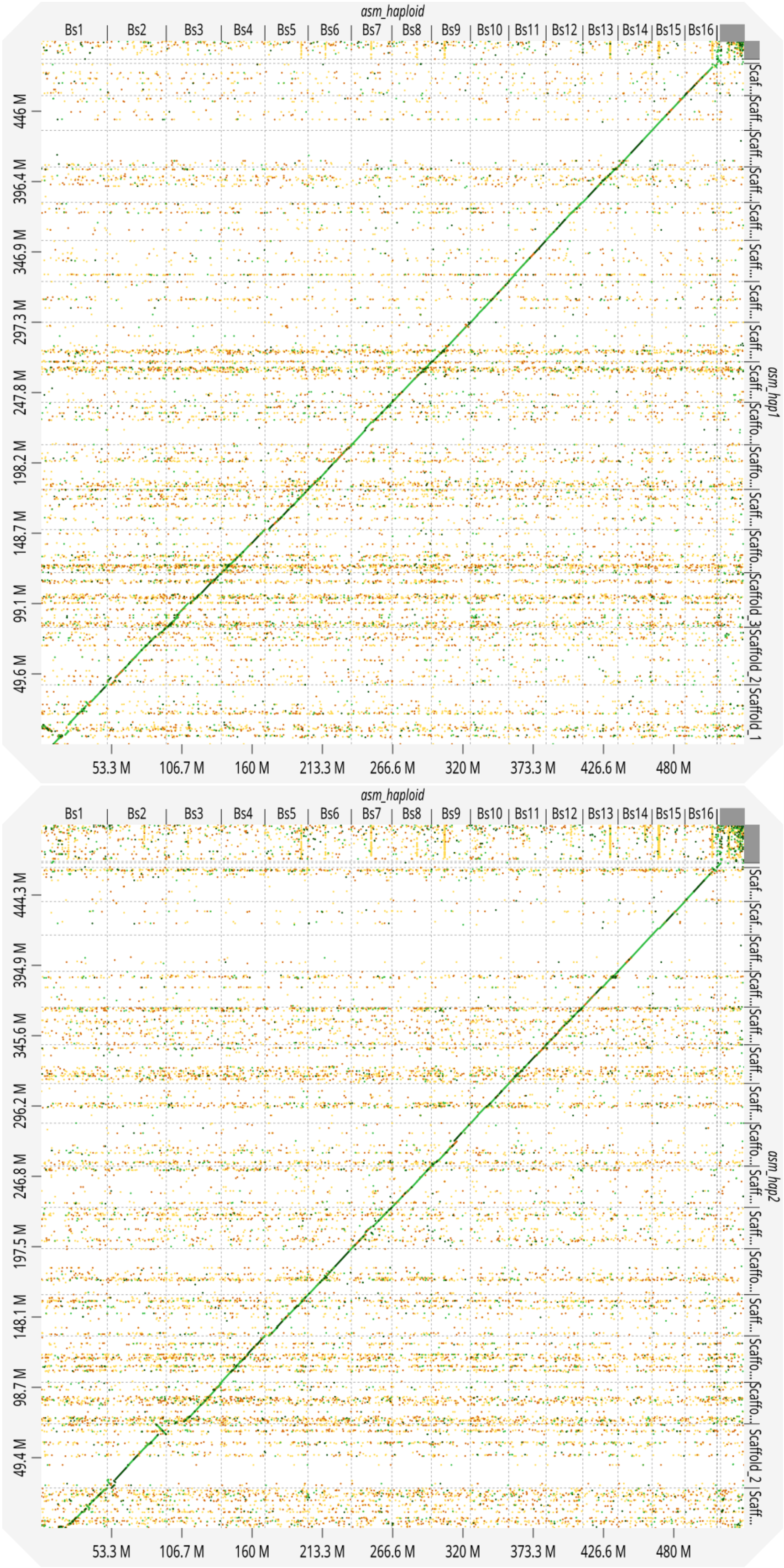

Figure S10. D-GENIES [121] dot plots of the final alignments of haplotype 1 (top) and haplotype 2 (bottom) on the haploid assembly, and used to order their scaffolds based on the latter.

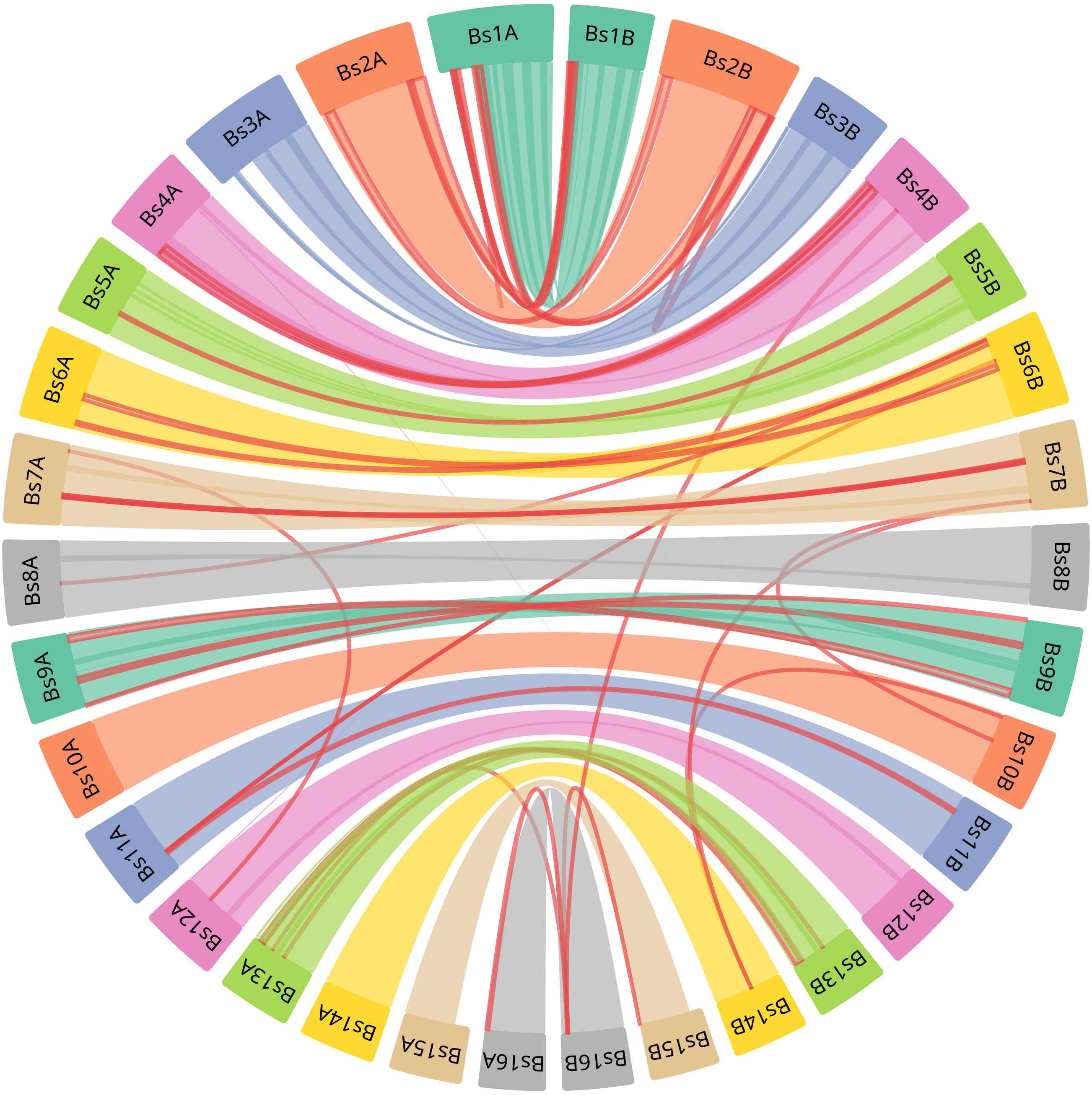

Figure S11. AccuSyn [36] representation of syntenic blocks identified using MCScanX [37] between the 16 largest scaffolds of haplotype 1 (left, with scaffold names ending in 'A') and haplotype 2 (right, with scaffold names ending in 'B') assemblies. Inverted blocks are highlighted in red.

.

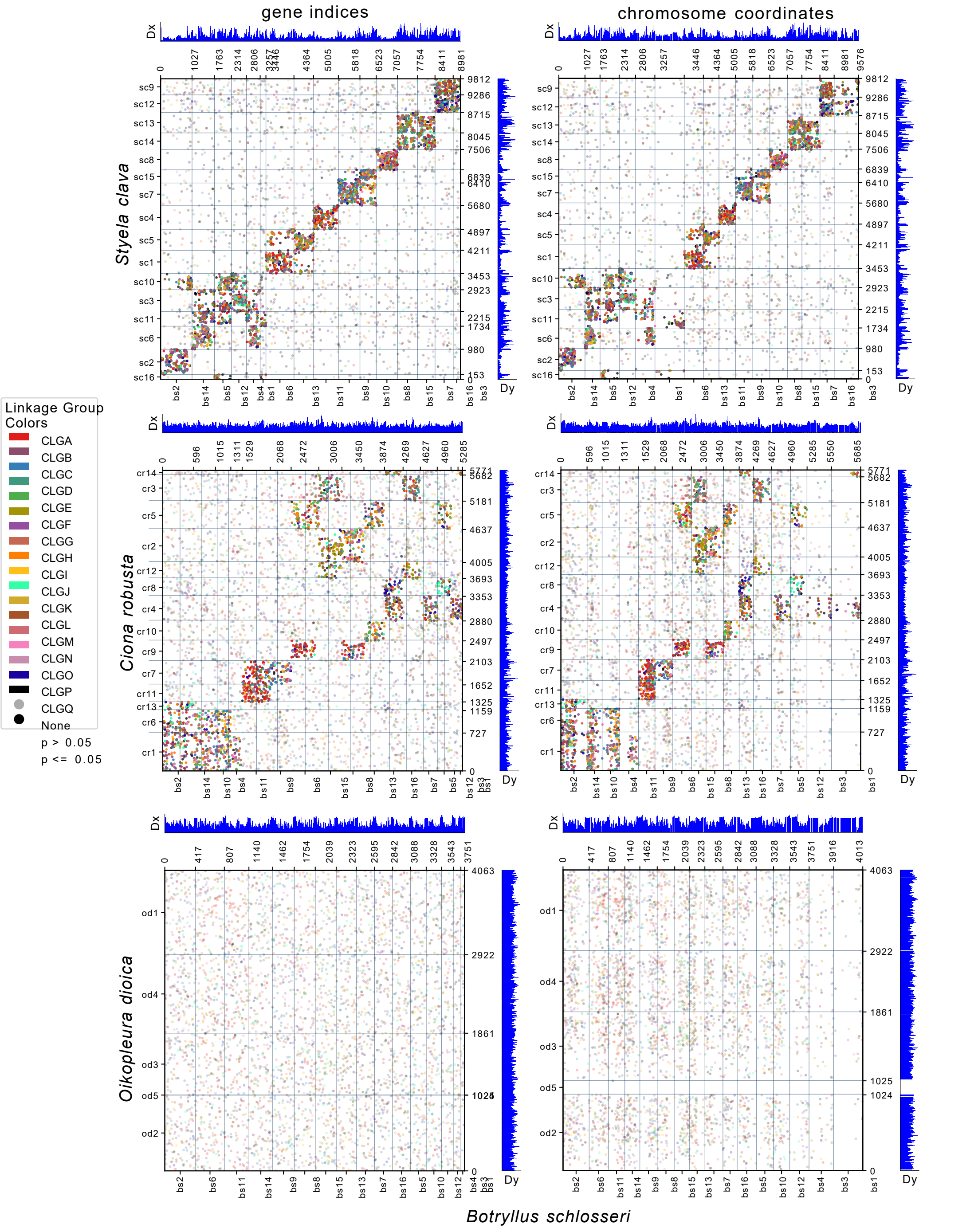

Figure S12. Investigation of synteny conservation among tunicate genomes. In the first column, dot plots depict the chromosome-scale scaffolds of *Botryllus schlosseri* (x-axis) plotted against those of *Styela clava*, *Ciona robusta* and *Oikopleura dioica* (y-axis). Each dot in the plot represents an ortholog, specifically a reciprocal best diamond blastp match between two species. The units of the x- and y-axes are the number of orthologous proteins: 9813, 5772 and 4064 orthologs found between the 16 *B. schlosseri* chromosome-scale scaffolds and the 16 of *S.plicata*, the 14 of *C.robusta* and the 5 of *O.dioica*, respectively. If there were chromosome breaks, Fisher's exact test (FET) was used to calculate the significance of the interactions between the sub-chromosomal pieces. Otherwise, FET was calculated on whole chromosomes. The opacity of the dots depicts the significance of FET. Dots that are a solid color are in cells with a FET p-value less than or equal to 0.05. Dots that are translucent are in cells with a FET p-value greater than 0.05. Dx and Dy values allow to pinpoint places where there may be sudden breaks in synteny [55] The second column of the figure depicts the same information as the first one, but plotted following chromosome base pair coordinates rather than gene index. This is better suited for visualizing gene-poor regions of the chromosomes.

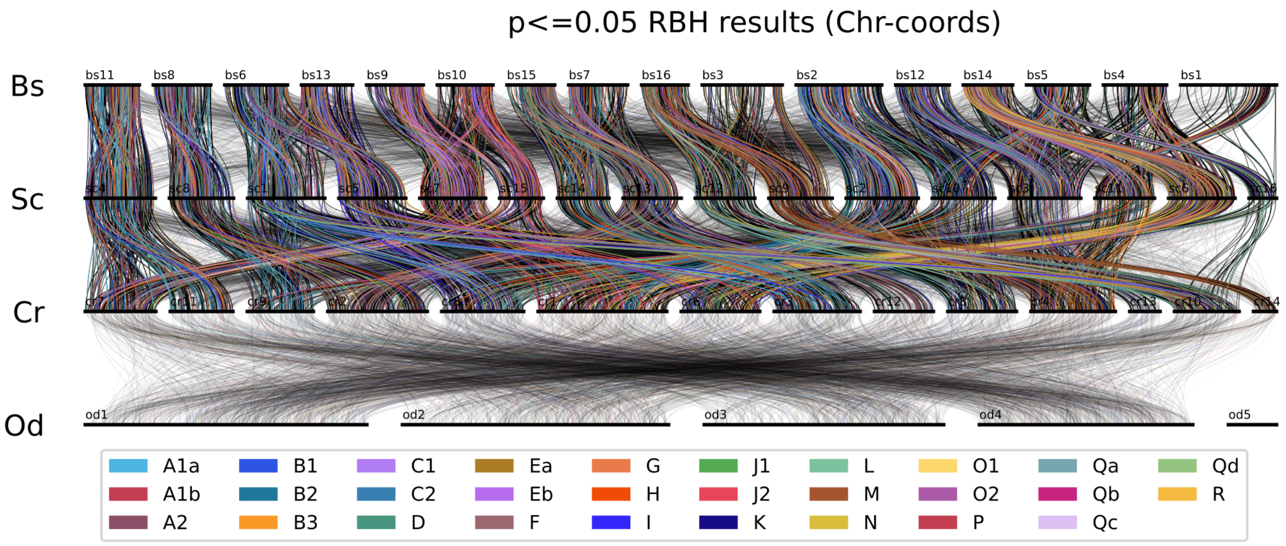

Figure S13. Synteny conservation of bilaterians, cnidarians and sponges groups [57] between *Botryllus schlosseri* (Bs), *Styela clava* (Sc), *Ciona robusta* (Cr) and *Oikopleura dioica* (Od).

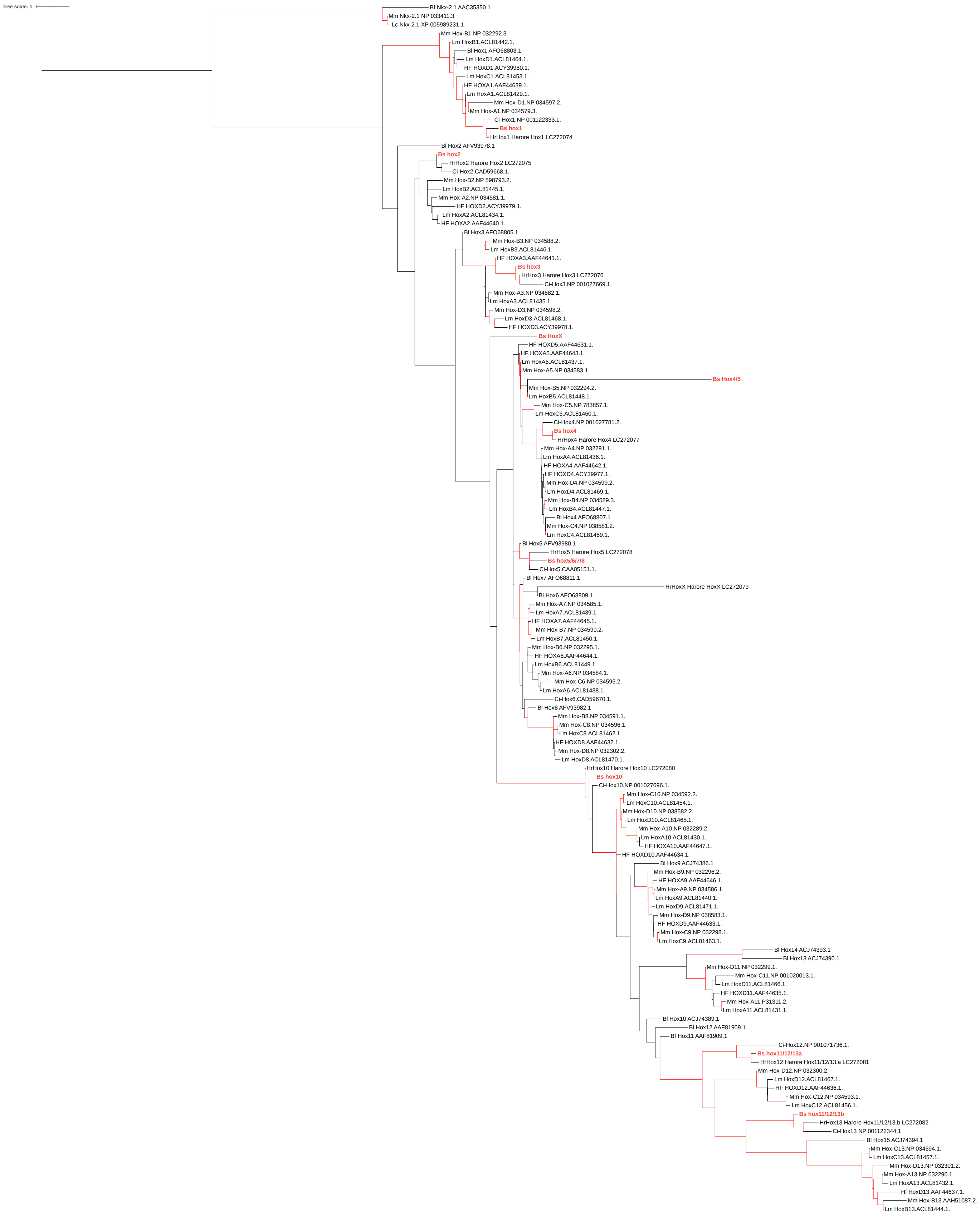

Figure S14. Phylogenetic analyses of *Hox* genes candidates of *Botryllus schlosseri*. The ML tree was generated using IQ-TREE 2 [115] by adding the *B. schlosseri* sequences to the alignment of Sekigami et al. 2017 [62] and keeping the homeodomains as well as the flanking 20 N-terminal and seven C-terminal amminoacids. Branches with ultra bootstrap values >90% are shown in red.
