# Supplementary material for: First chromosome-level genome assembly of the colonial chordate model Botryllus schlosseri (Tunicata)
Source: Gigascience. 2025 Sep 18;14:giaf097. doi: 10.1093/gigascience/giaf097 (PMC12448946; doi:10.1093/gigascience/giaf097)
Supplement: giaf097_GIGA-D-25-00071_Revision_1 [file giaf097_giga-d-25-00071_revision_1.pdf]

GigaScience

First chromosome-level genome assembly of the colonial chordate model Botryllus schlosseri (Tunicata)

--Manuscript Draft--

**Manuscript Number:**	GIGA-D-25-00071R1
**Full Title:**	First chromosome-level genome assembly of the colonial chordate model Botryllus schlosseri (Tunicata)
**Article Type:**	Data Note
**Funding Information:**	Agence Nationale de la Recherche (ANR-14-CE02-0019-01) — Dr. Stefano Tiozzo<br>Agence Nationale de la Recherche (ANR- 24-CE02-2277) — Dr. Stefano Tiozzo<br>Centre National de la Recherche Scientifique (INSB-DBM) — Dr. Stefano Tiozzo<br>Fundação de Amparo à Pesquisa do Estado de São Paulo (15/50164-5 & 19/06927-5) — Dr. Federico D. Brown<br>Fonds De La Recherche Scientifique - FNRS (T.0078.23) — Dr. Jean-Francois Flot
**Abstract:**	Background: Botryllus schlosseri (Tunicata) is a colonial, laboratory model tunicate recognized for its remarkable developmental diversity, its regenerative abilities, and its peculiar genetically determined allorecognition system governed by a polymorphic locus controlling chimerism and cell parasitism. Results: We report the first chromosome-level genome assembly of B. schlosseri sub-clade A1. By integrating long and short reads with Hi-C scaffolding, we produced both a phased diploid genome assembly and a conventional collapsed consensus sequence of 533 Mb. Of this total length, 96% belonged to 16 chromosome-scale scaffolds, with a BUSCO completeness score of 91.4%. We then compared our assembly with other high-quality tunicate genomes, revealing some synteny conservation but also extensive genomic rearrangements and a general loss of colinearity. Conclusions: The chromosome-level resolution of this assembly enhances our understanding of genome organization in colonial modular organisms. Comparative analyses highlight the dynamic nature of tunicate genomes, with conserved macrosynteny yet extensive microsyntenic rearrangements and scrambling, underscoring their rapid evolutionary trajectory. This high-quality genome assembly provides a valuable resource for exploring the unique biological features of colonial chordates, including their exceptional regenerative abilities and complex allorecognition system.
**Corresponding Author:**	Stefano Tiozzo<br>CNRS: Centre National de la Recherche Scientifique<br>Villefranche sur Mer, PACA FRANCE
**Corresponding Author Secondary Information:**	
**Corresponding Author's Institution:**	CNRS: Centre National de la Recherche Scientifique
**Corresponding Author's Secondary Institution:**	
**First Author:**	Olivier De Thier
**First Author Secondary Information:**	

Powered by Editorial Manager® and ProduXion Manager® from Aries Systems Corporation

**Order of Authors:**	Olivier De Thier
	Marie Lebel
	Mohammed M. Tawfeeq
	Roland Faure
	Philippe Dru
	Simon Blanchoud
	Alexandre Alie
	Federico D. Brown
	Jean-Francois Flot
	Stefano Tiozzo
**Order of Authors Secondary Information:**	
**Response to Reviewers:**	Replies to reviewers' comments (see also attached file)<br><br>We sincerely appreciate the reviewers' insightful and constructive feedback. Below, we respond<br>to each comment point by point, outlining the corresponding revisions and clarifications made<br>to enhance the manuscript. In addition to addressing the reviewers' comments, we made a<br>minor revision to the section on genome size estimation using Feulgen staining ; this is because<br>a genome assembly of the Lasius niger standard we used is about to be published, indicating a<br>genome size of 0.30 pg instead of the value of 0.32 pg assumed previously. We also added two<br>extra figures to that part. Finally, we took the opportunity to correct a few typos and refine<br>some imprecise definitions. For ease of review, all modifications in the manuscript main text<br>are highlighted in red.<br><br>Reviewer 1<br>In this manuscript, De Thier and colleagues reported the chromosomal level genome assembly<br>of tunicate Botryllus schlosseri (Pallas, 1766) sub-clade A1. The methods used in this study<br>are standard. B. schlosseri has been used as laboratory model in certain places to understand<br>asexual development and regeneration for decades. Despite there was a draft quality genome<br>published a decade ago (eLife 2013, 2:e00569), the authors here produced a high-quality phased<br>genome based on modern technologies. In terms of genomic resources for this laboratory model,<br>this is important and useful. The authors have also carried out analyses, including repeats,<br>synteny, and Hox cluster genes. I also think some of these results are interesting. Below are<br>my comments and suggestions for the authors to consider which hopefully can further improve<br>the manuscript.<br><br>1. Given the authors merged the results and discussion into one section, I would expect more<br>discussion for several parts, including: - a. Repeats - For now, the analysis is quite standard<br>and the main text is relatively descriptive. The question to me is what have we learnt

Powered by Editorial Manager® and ProduXion Manager® from Aries Systems Corporation

from
understanding the repeats from B. schlosseri genome? The authors should tell the
readers. - b.
Synteny analyses - This is an interesting finding. Extensive chromosomal
rearrangement has
also been discovered in other animals in recent. Can the authors further discuss these
events?
- c. Hox gene analyses - Again, it is quite descriptive. Tunicates are well known for
dispersed
Hox cluster for decades. So what have we learnt from the situation of B. schlosseri
which I
would be glad to see if the authors can discuss them.

Given the editors’ decision to classify the manuscript as a ’Data Note’ article due to the
"lack
of biological validation", we initially revised the discussion to align to this format,
resulting in
a more concise discussion. Nonetheless, we followed reviewer’s suggestions and
expanded the
discussion to emphasize the significance of repetitive elements in B. schlosseri,
particularly their
potential role in genome plasticity and evolutionary adaptation. We also compared our
findings
to recent studies reporting chromosomal rearrangements in tunicates and other
metazoans.
Additionally, we provide further context on the organization of the Hox cluster in B.
schlosseri,
highlighting its implications in comparison to other tunicate species.

2. Figure S14 - The authors should also show the bootstrap values on the key nodes.

Bootstrap values have been added on the former Figure S14, now Figure S19

- In addition, the authors should also use one more method to construct the Hox gene
tree in
addition to Maximum Likelihood method.

A tree built using a Bayesian approach has been added as Figure S20

Reviewer 2
De Thier et al. present a high-quality chromosome scale de novo assembly of the
tunicate
Botryllus schlosseri from mainly PacBio HiFi and Arima Hi-C reads. Further WGS
Illumina
and ONT data was applied to resolve assembly errors or support the correctness of the
assembly structure. Structural and functional annotations are conducted thoroughly.
Downstream
analyses include a synteny comparison of different Tunicata based on ancestral
linkage groups
and Hox genes.
The manuscript is well written and methods are mostly described to ensure
reproducibility.
Despite the good shape of the manuscript, I would like to give some remarks, which
should be
addressed in a revised manuscript before publication.
General remarks
I like the quote in the beginning of the introduction.
The authors conducted downstream analyses with different related tunicate genome
assemblies
on chromosome level. For assembly metrics, there is a comparison regarding BUSCO
assessment
only. I would point out the high quality of the B. schlosseri assembly in Table 2 and 4
by

Powered by Editorial Manager® and ProduXion Manager® from Aries Systems Corporation

comparison with the other chromosome level and annotated tunicate genome assemblies as
well.

Table S3 has been added, comparing the metrics of B.schlosseri, Styela clava, Ciona robusta,
and Oikopleura dioica.

I am not an expert regarding tunicates, so please excuse my basic, curiosity driven question: In
the results section "The laboratory model Sub-clade A1" you state that a part of COI is used
as a barcode to differentiate ascidian species. In the introduction you state that wild colonies
are able to fuse resulting in mixed genotypes. Since sample E* derived from the wild at some
point, it might be theoretically possible to have not only mixed nuclear genotypes but mixed
mitotypes too. Depending on how old sample E* is and how fast fixation of a mitotype can
happen within a colony, this might be reflected in your data. Furthermore, this thought could
be expanded to nuclear genotypes, which could hamper scientific findings.

Clone E* is isogenic and, barring the unlikely accumulation of somatic mutations, possesses
a single mitochondrial genome. We have revised the text to clarify our rationale for selecting
clone E*

Contamination filtering was based on a sequence similarity search and taxonomic assignment
of blobtools only. Despite blobtools/blobtoolkit was applied I was not able to find a blobplot in
the supplemental files. I would like to encourage the authors to add blobplots before and after
contamination filtering at least to the supplement. In my opinion, blobplots are most powerful
when considering GC content and coverage in the first place - especially, when dealing with
taxa, which are underrepresented in public databases. Therefore, using taxonomic assignment
only for contamination filtering might generate false positives (e.g. conserved sequences across
the tree of life with taxonomic assignment different than Chordata but with similar GC and
coverage as the target) and false negatives (e.g. short sequences of the assembly, which couldn't
be assigned with different GC and coverage as the target).

The Blobplots for the collapsed as well as for each of the two haplotypes (before and after
filtering) are in Figure S6

In the paragraphs "Results and Discussion" (Haplotype-resolved assembly) as well as
in "Methods" (Haploid genome assembly) you use the term "haploid assembly" multiple times. I find
this term misleading, since the genome is not haploid and the assembly represents
both haplotypes at the same time. I assume that primary contigs from hifiasm were used to generate
this assembly. Therefore, I would suggest to e.g. call this assembly "based on primary
contigs", "non phased", "haplotype mixed" or "haplotype unresolved" (as opposite to "haplotype
2resolved").

The term "haploid assembly" has been replaced with "collapsed assembly" to prevent misinterpretation (following the terminology advocated by Heng Li, https://lh3.github.io/2021/04/17/concepts-in-phased-assemblies).

Particular remarks
Results and Discussion
Sequencing and genome size estimation
Table 1 Please specify what "round 1" and "round 2" are referring to. Was one library sequenced twice or were two different libraries created and sequenced?

This information has been updated in the "Methods" section

Haploid genome assembly
“We identified 28 contigs that belong to spore-forming unicellular parasites of the microsporidia group [32]. This represents the first report of this fungal group in a tunicate species.” Is this identification based on blobtools taxonomic assignment? This is not described in the methods.
Furthermore, can you rule out that identification or taxonomic assignment is false positive? If not you should tune down the second sentence and maybe discuss this.

The text has been modified accordingly

“We then performed Hi-C scaffolding using YaHS [34], which reduced the number of contigs to 256, before [...]” Technically, scaffolding with yahs can only increase the number of contigs because original (hifiasm) contigs are split because of the Hi-C signal (at least as long the option –no-contig-ec isn’t applied). I would substitute "contigs" with "sequences".

The text have been modified accordingly

“Finally, a manual curation was performed, resulting in an assembly made up of 16 major scaffolds [...]” Is there any previous study on the karyotype of B. schlosseri? If so, citing it here would strengthen your results. Otherwise, I would recommend to state the karyotypes or the number of chromosome scale scaffolds of other tunicates here and discuss, if your findings are in line.

Previous studies discussing B.schlosseri have been cited (Colombera 1969), discussed on page 4 and cited in Figure S14

Table 2 Please substitute "No. of scaffolds" with "No. of sequences". Please add the contig N50 values. As pointed out above, I would like to see a comparison to the other chromosome level tunicate genome assemblies here, instead of showing basically the same stats twice.

The text have been modified accordingly

“[. . .] highlighted the presence of two large-scale genomic palindromes located within Bs1 and a smaller one in Bs3 (Figure 3).” The figure shows the presence but maybe you can

highlight
them in the figure and the caption even more? “To find out whether these palindromes may
result from assembly artifacts [40], we checked the localization of the duplicated BUSCO genes
along the chromosomes and did another run of CRAQ [...]” You could support your findings by
showing an even coverage distribution within the palindromes, which is similar to the coverage
distribution of whole assembly. Either as a histogram or a zoomed in version of the read
coverage across reference as in the outer layer of the circos plot could show this nicely.

The text have been modified to highlight them more. The sequencing depth profiles have been
added as supplementary figures.

Methods
Sampling, DNA isolation, and sequencing
"HiFi PacBio long reads" Please provide more details on how PacBio libraries (was it actually
one library sequenced twice or two different libraries?) were created and sequenced. Were low
or ultra-low protocols used? On which machine was sequencing conducted?

These information have been updated in the text

RNA-seq data
3Is downloading public data a method? In any case you should cite the original papers and
provide a list of accession numbers (supplement) but I would remove this paragraph and add
the information to the paragraph "Genome annotation", e.g. "Public available RNA-seq reads
[23, 25, 8] were aligned to the soft-masked assemblies [...]"

The text have been modified accordingly

Data preprocessing
Depending on how the PacBio libraries were created and which PacBio machine was utilized
for sequencing, you should state how HiFi calling was conducted (e.g. Sequel II) and how PCR
adapter and duplicates were filtered out (e.g. ultra-low).

The information has been updated in the session "Methods"

Haploid genome assembly
“To this aim, contigs were aligned to the NCBI nucleotide database (accessed 2023 March 18)
using BLAST+ [78]” Please state the version of BLAST+.

v2.13.0+, the text have been modified accordingly

“Finally, a BLASTN search for fragments of the mitochondrial genome among the contigs
was performed using the published complete mitochondrial genome of B. schlosseri (RefSeq
NC_021463.1) [28].”
Were the fragments filtered out based on the blast search? Please explain what was done
in detail. Which hits were considered (e.g. cutoffs)? The mitochondrial genome of E* was

Powered by Editorial Manager® and ProduXion Manager® from Aries Systems Corporation

assembled with NOVOPlasty, which is by the way not stated in the methods but in the results
only. Was the assembled mt genome of E* added to the assembly, once the fragments were
filtered out?

The mitochondrial genome assembly has been added in its own subsection in the session "Methods". BLAST cutoffs to identify the mtDNA fragments have been added, the mtDNA assembly
and identified fragments were removed from the assemblies (the text have been modified accordingly).

Haplotype-resolved assembly
If I understand correctly, the rapid curation pipeline was applied but no dual-curation was
conducted. When aiming for haplotype-resolved assemblies, I would recommend to apply this
method, e.g. concatenating both haplotypes and creating a combined contact map of haplotype
1 and 2, which can be curated as usual, with the advantage of being able to exchange (parts
of) sequences between the haplotypes. In some cases phasing from hifiasm is not correct and
can be easily corrected with this approach.

The steps to generate the haplotype 1 and haplotype 2 assemblies were similar to those used for
the unphased assembly, following the initial assembly and haplotype purging. Each haplotype
assembly was treated individually thereafter. An attempt was made to scaffold both haplotypes
together by concatenating them prior to scaffolding, but the results were less satisfactory:
• Only 30 major scaffolds were produced (instead of the expected 32), as 2 scaffolds contained merged sequences from both haplotype 1 and haplotype 2.
• After manual curation to confidently recover 2×16 major scaffolds, the total assemblies
sizes were 439 Mbp and 444 Mbp, both smaller than the expected 500 Mbp, and also smaller than the size obtained by scaffolding the haplotypes individually (480 Mbp and 464 Mbp).
• Structural variations and discrepancies in putative chromosome lengths, relative to the
B. schlosseri karyogram, were still observed. This suggests that concatenation did not resolve potential misassemblies in our case.

Reviewer 3
In this MS entitled 'First chromosome-level genome assembly of the colonial chordate model
Botryllus schlosseri (Tunicata)', Olivier De Thier and colleagues report the first chromosomescale assembly of this colonial ascidian specie, paying special attention to differences with
previous published assemblies and importantly between haplotypes. The MS is very well written, very easy and pleasant to read. This provides data of great quality and very relevant
not only for the ascidian/tunicate community, but to the field of genome structural evolution.
I firmly recommend it for publication, although I think that the authors could discuss it in deeper detail. Specially, I miss for instance a more elaborate discussion of the results in our
understanding of the similarities and differences between clades that have been published in
the last years (I have not been able to find some relevant articles in this regard cited in the

bibliography).

We have revised the text and included three bibliographical references to better clarify the distinctions between clades. A detailed discussion of their differences and geographic distribution,
however, lies beyond the scope of this manuscript.

I also feel that a deeper analysis of the differences between haplotypes could be very interesting,
unless they are artifactual effects of the assemblies. As mentioned below, unless this is part
of a longer story for a different MS beyond the scope of this one, I encourage the authors to
validate some of the differences they find between haplotypes, and try to correlate the structural
variations, with differences in gene counts between haplotypes, and to explore whether these
differences could be correlated with aspects of biological relevance. I miss, for instance, Venn
diagrams with gene contents between previous assemblies, and the haplotypes/haploid genome
here reported. In any case,

Since it remains uncertain which differences between haplotypes arise from assembly or phasing
artifacts, and which reflect true biological variation, we chose not to expand this analysis further
at this stage. A more comprehensive examination and characterization of haplotype differences
will be addressed in a future study.

I firmly recommend this MS for publications, since most of my suggestions are not intended to
interrogate the results of the MS, but to improve it, but I also understand that some may go
beyond the scope of this MS.
Minor points: Introduction Page 1: "the basic body plan of adult tunicates is highly conserved
across the entire subphylum [3]". This sentence, which could be OK for ascidians, probably
provides a highly simplified vision of Tunicate adult morphologies, specially comparing the
divergent morphologies of Thaliaceans and Appendicularians. Please, elaborate the sentence.

The main text has been modified

To understand the comparisons between the data of this MS and previously reported genomes,
it seems crucial to understand well the meaning of the "clades and subclades". Please, include
in the introduction (or where needed), how are defined those clades, which are their origins and
biological/geographical differences, . . . and all the critical information that will specially help
non-tunicate readers to understand the results.

The results-discussion section "The laboratory model Sub-clade A1" has been modified accordingly.

Results: The authors refer to the presence of large-scale genomic palindromes in Bs1 and Bs3.
But it is unclear what are these structures. I suggest to please provide some more detailed

explanation about the palindromic nature of these regions.

Additional details on the palindromic nature of these regions, along with their coverage, are now provided in Supplementary Figures S11, S12, and S13.

The data of haplotype-resolved assemblies is very interesting. I wonder if it is possible to 5somehow measure the amount of heterozygosity between haplotype 1 and 2, and those versus the previous versions of the genome, to better understand intra and inter-variation between subclades?

A rough estimation of heterozygosity between two haplotypes can be obtained by mapping one haplotype assembly onto the other, performing variant calling, and dividing the number of identified SNPs by the size of the smaller haplotype assembly. However, this mapping-based approach is likely to underestimate the true heterozygosity level, as it may miss divergent regions (such as structural variants between haplotypes or collapsed regions if we consider the unphased assembly). In contrast, a k-mer-based approach is more suitable and less biased for this task, although it requires high-quality reads and sufficient even sequencing coverage (at least 25x for Genomescope). For instance, we estimated a heterozygosity level of 1.28% by counting SNPs called after mapping haplotype 2 onto haplotype 1; 1.6% by calling SNPs from Illumina reads mapped to the unphased assembly; and 3.63% using a k-mer-based approach. Attempting to estimate the heterozygosity level from the previous genome assembly by Voskoboynik et al. is unlikely to yield an accurate value for comparison, as their assembly was generated using reads from different colonies (Sc6a-b and 356a) and involved different sequencing technologies, resulting in varying levels of coverage and evenness.

The differences of the size of some regions between Colombera and this study, and even between haplotypes 1 and 2, are very interesting. I would find more informative to merge the three graphs of Figure S9 into one single graph, so we can also easily compare the different in sizes of the haplotypes with the haploid.

The three graphs have been merged in a single figure in the text.

If some of those differences are actually due to deletions, that would deserve further analysis.
If this analysis is not part of another ongoing project that will be published somewhere else,
I suggest identifying with a dot-plot some of those differences, specially between haplotypes,
and validate with long-reads crossing those regions whether some of the deletions are real or artifactual. Please, include the dotplot graph together with the two haplotypes in figure S10.

Former Figure S10 (now Figure S15) has been modified accordingly

Powered by Editorial Manager® and ProduXion Manager® from Aries Systems Corporation

In those cases that could be real, it would be very interesting what genes are gone, and if
those are not placed somewhere else in the genome as result of translocations, or those genes
are actually gone and could explain some of the differences reported in the gen count between
haplotypes.

A more in-depth examination and description of haplotype differences will be addressed in a
future study.

The authors mentioned the presence of multiple structural variations, although some of which
could be artifactual of miss-assemblies. Interestingly, the plot of the synteny blocks between
the two haplotypes in figure S11 shows some of those structural variations, including cases of:
- deletions: for instance, there are "blank" regions in Bs1A and Bs3A with no lines, which
may reflect areas that are not present in the haplotype B. - duplications and translocations
within chromosomes or between chromosomes of different haplotypes. Just looking to this plot,
I wonder how the distribution of chromosomes between haplotypes is done. For instance, I see
that Bs7B shares a duplicated synteny block with chromosomes Bs10B and Bs14B, but not
with Bs10A and Bs10B, which means that the duplications are intra-haplotype present in B
but not in A. But I wonder if it is possible that Bs10B and Bs14B could be in fact switched to
haplotype A, and therefore there would be no duplication nor deletion in one of the haplotypes,
just a simple translocation. I may be wrong in the interpretation, but I'm curious to understand
the graph.

The interpretation of the structural variations from the graph seems correct. However, without
sequencing reads from the two parental genomes of clone E*, which are unfortunately
now untraceable, and without using techniques such as trio binning (https://doi.org/10.1038/nbt.
64277), we cannot accurately separate chromosomes into haplotype-specific sets corresponding
to each parent. As a result, haplotypes A and B consist of arbitrarily selected chromosomes
from both parents, and we cannot verify whether translocations between haplotypes should be
favored over duplications within a haplotype as the more likely explanation.

In any case, again, as mentioned above, it would be worthy to validate some of those variations
with long reads, which could illuminate the biological relevance between the haplotypes and
discard potential artifactual errors of the assemblies.
I notice that in figures 7 and S13, some lines are thicker than others. Is this because many
"thin" lines are overlapped, and they look like a "thick" line. Otherwise, the visual effect of
different thicknesses could be misleading. Please, clarify.

The "thick" lines correspond to significant interactions between species, a more

Powered by Editorial Manager® and ProduXion Manager® from Aries Systems Corporation

	detailed explanation has been added in the figure captions.<br><br>In the analysis of the Hox cluster the authors say "[. . .] our new assembly revealed that B.<br>schlosseri's Hox genes are not scattered. Instead, eight of them were clustered on the second<br>largest scaffold (Bs2), whereas two other ones are found on the 15th largest scaffold (Bs15)."<br>Generally, the description of the Hox gene in a cluster refers to the fact they are in the vicinity,<br>with near not many other genes in between Hox genes. Therefore, I would not describe that<br>eight Hox genes are clustered by the simple fact that they are in the same chromosome (maybe<br>even in different arms).<br><br>We agree with the Reviewer correction. The text has been modified accordingly
**Additional Information:**	
**Question**	**Response**
Are you submitting this manuscript to a special series or article collection?	No
**Experimental design and statistics**<br><br>Full details of the experimental design and statistical methods used should be given in the Methods section, as detailed in our Minimum Standards Reporting Checklist. Information essential to interpreting the data presented should be made available in the figure legends.<br><br>Have you included all the information requested in your manuscript?	Yes
**Resources**<br><br>A description of all resources used, including antibodies, cell lines, animals and software tools, with enough information to allow them to be uniquely identified, should be included in the Methods section. Authors are strongly encouraged to cite Research Resource Identifiers (RRIDs) for antibodies, model organisms and tools, where possible.<br><br>Have you included the information requested as detailed in our Minimum Standards Reporting Checklist?	Yes

**Availability of data and materials**<br><br>All datasets and code on which the conclusions of the paper rely must be either included in your submission or deposited in publicly available repositories (where available and ethically appropriate), referencing such data using a unique identifier in the references and in the "Availability of Data and Materials" section of your manuscript.<br><br>Have you have met the above requirement as detailed in our Minimum Standards Reporting Checklist?	Yes
GigaScience has policies and guidelines in place for the use of generative AI-writing tools such as ChatGPT. If you have used such writing tools to assist with writing the manuscript this must be declared and cited in the text. Authors should not list AI-writing tools and other AI-assisted technologies as an author or co-author and should acknowledge that they are fully responsible for text generated or refined by AI-writing tools.<p><br><br>A summary of use (particularly in the introduction or among methods) needs to be included at the end of the paper, and the outputs should also be included as a supplementary file hosted in GigaDB or other open repositories. Please read our guidelines for more information. <p><br><br>By submitting to GigaScience, you are aware of the journal's AI-writing tools policy, and if you have declared use of such tools below, you have acknowledged this where appropriate in your manuscript and have made a summary of use and outputs available. <p><br><b>AI-assisted writing tools have been used in the preparation of this manuscript?	

Powered by Editorial Manager® and ProduXion Manager® from Aries Systems Corporation

LaTeX - Main Document (TeX file)
Click here to access/download;LaTeX - Main Document (TeX file);main.tex

```
This is pdfTeX, Version 3.141592653-2.6-1.40.26 (TeX Live 2024)
(preloaded format=pdflatex 2024.8.2)  25 JUL 2025 04:52
entering extended mode
 restricted \write18 enabled.
 %&-line parsing enabled.
**main.tex
(./main.tex
LaTeX2e <2024-06-01> patch level 2
L3 programming layer <2024-05-27>
(./oup-contemporary.cls
Document Class: oup-contemporary 2023/06/12, v1.2
(c:/texlive/2024/texmf-dist/tex/latex/base/article.cls
Document Class: article 2024/02/08 v1.4n Standard LaTeX document class
(c:/texlive/2024/texmf-dist/tex/latex/base/size10.clo
File: size10.clo 2024/02/08 v1.4n Standard LaTeX file (size option)
)
\c@part=\count194
\c@section=\count195
\c@subsection=\count196
\c@subsubsection=\count197
\c@paragraph=\count198
\c@subparagraph=\count199
\c@figure=\count266
\c@table=\count267
\abovecaptionskip=\skip49
\belowcaptionskip=\skip50
\bibindent=\dimen141
) (c:/texlive/2024/texmf-dist/tex/generic/iftex/ifxetex.sty
Package: ifxetex 2019/10/25 v0.7 ifxetex legacy package. Use iftex
instead.
(c:/texlive/2024/texmf-dist/tex/generic/iftex/iftex.sty
Package: iftex 2022/02/03 v1.0f TeX engine tests
)) (c:/texlive/2024/texmf-dist/tex/latex/base/inputenc.sty
Package: inputenc 2024/02/08 v1.3d Input encoding file
\inpenc@prehook=\toks17
\inpenc@posthook=\toks18
) (c:/texlive/2024/texmf-dist/tex/latex/base/fontenc.sty
Package: fontenc 2021/04/29 v2.0v Standard LaTeX package
) (c:/texlive/2024/texmf-dist/tex/latex/lm/lmodern.sty
Package: lmodern 2015/05/01 v1.6.1 Latin Modern Fonts
LaTeX Font Info:    Overwriting symbol font `operators' in version
`normal'
(Font)              OT1/cmr/m/n --> OT1/lmr/m/n on input line 22.
LaTeX Font Info:    Overwriting symbol font `letters' in version `normal'
(Font)              OML/cmm/m/it --> OML/lmm/m/it on input line 23.
LaTeX Font Info:    Overwriting symbol font `symbols' in version `normal'
(Font)              OMS/cmsy/m/n --> OMS/lmsy/m/n on input line 24.
LaTeX Font Info:    Overwriting symbol font `largesymbols' in version
`normal'
(Font)              OMX/cmex/m/n --> OMX/lmex/m/n on input line 25.
LaTeX Font Info:    Overwriting symbol font `operators' in version `bold'
(Font)              OT1/cmr/bx/n --> OT1/lmr/bx/n on input line 26.
LaTeX Font Info:    Overwriting symbol font `letters' in version `bold'
(Font)              OML/cmm/b/it --> OML/lmm/b/it on input line 27.
```

```
LaTeX Font Info:    Overwriting symbol font `symbols' in version `bold'
(Font)                  OMS/cmsy/b/n --> OMS/lmsy/b/n on input line 28.
LaTeX Font Info:    Overwriting symbol font `largesymbols' in version
`bold'
(Font)                  OMX/cmex/m/n --> OMX/lmex/m/n on input line 29.
LaTeX Font Info:    Overwriting math alphabet `\mathbf' in version
`normal'
(Font)                  OT1/cmr/bx/n --> OT1/lmr/bx/n on input line 31.
LaTeX Font Info:    Overwriting math alphabet `\mathsf' in version
`normal'
(Font)                  OT1/cmss/m/n --> OT1/lmss/m/n on input line 32.
LaTeX Font Info:    Overwriting math alphabet `\mathit' in version
`normal'
(Font)                  OT1/cmr/m/it --> OT1/lmr/m/it on input line 33.
LaTeX Font Info:    Overwriting math alphabet `\mathtt' in version
`normal'
(Font)                  OT1/cmtt/m/n --> OT1/lmtt/m/n on input line 34.
LaTeX Font Info:    Overwriting math alphabet `\mathbf' in version `bold'
(Font)                  OT1/cmr/bx/n --> OT1/lmr/bx/n on input line 35.
LaTeX Font Info:    Overwriting math alphabet `\mathsf' in version `bold'
(Font)                  OT1/cmss/bx/n --> OT1/lmss/bx/n on input line 36.
LaTeX Font Info:    Overwriting math alphabet `\mathit' in version `bold'
(Font)                  OT1/cmr/bx/it --> OT1/lmr/bx/it on input line 37.
LaTeX Font Info:    Overwriting math alphabet `\mathtt' in version `bold'
(Font)                  OT1/cmtt/m/n --> OT1/lmtt/m/n on input line 38.
) (c:/texlive/2024/texmf-dist/tex/generic/iftex/ifpdf.sty
Package: ifpdf 2019/10/25 v3.4 ifpdf legacy package. Use iftex instead.
) (c:/texlive/2024/texmf-dist/tex/latex/microtype/microtype.sty
Package: microtype 2024/03/29 v3.1b Micro-typographical refinements (RS)
(c:/texlive/2024/texmf-dist/tex/latex/graphics/keyval.sty
Package: keyval 2022/05/29 v1.15 key=value parser (DPC)
\KV@toks@=\toks19
) (c:/texlive/2024/texmf-dist/tex/latex/etoolbox/etoolbox.sty
Package: etoolbox 2020/10/05 v2.5k e-TeX tools for LaTeX (JAW)
\etb@tempcnta=\count268
)
\MT@toks=\toks20
\MT@tempbox=\box52
\MT@count=\count269
LaTeX Info: Redefining \noprotrusionifhmode on input line 1061.
LaTeX Info: Redefining \leftprotrusion on input line 1062.
\MT@prot@toks=\toks21
LaTeX Info: Redefining \rightprotrusion on input line 1081.
LaTeX Info: Redefining \textls on input line 1392.
\MT@outer@kern=\dimen142
LaTeX Info: Redefining \textmicrotypecontext on input line 2013.
\MT@listname@count=\count270
(c:/texlive/2024/texmf-dist/tex/latex/microtype/microtype-pdftex.def
File: microtype-pdftex.def 2024/03/29 v3.1b Definitions specific to
pdftex (RS)

LaTeX Info: Redefining \lsstyle on input line 902.
LaTeX Info: Redefining \lslig on input line 902.
\MT@outer@space=\skip51
```

```
)
Package microtype Info: Loading configuration file microtype.cfg.
(c:/texlive/2024/texmf-dist/tex/latex/microtype/microtype.cfg
File: microtype.cfg 2024/03/29 v3.1b microtype main configuration file
(RS)
)) (c:/texlive/2024/texmf-dist/tex/latex/euler/euler.sty
Package: euler 1995/03/05 v2.5
Package: `euler' v2.5 <1995/03/05> (FJ and FMi)
LaTeX Font Info:    Redeclaring symbol font `letters' on input line 35.
LaTeX Font Info:    Encoding `OML' has changed to `U' for symbol font
(Font)              `letters' in the math version `normal' on input line
35.
LaTeX Font Info:    Overwriting symbol font `letters' in version `normal'
(Font)                  OML/lmm/m/it --> U/eur/m/n on input line 35.
LaTeX Font Info:    Encoding `OML' has changed to `U' for symbol font
(Font)              `letters' in the math version `bold' on input line
35.
LaTeX Font Info:    Overwriting symbol font `letters' in version `bold'
(Font)                  OML/lmm/b/it --> U/eur/m/n on input line 35.
LaTeX Font Info:    Overwriting symbol font `letters' in version `bold'
(Font)                  U/eur/m/n --> U/eur/b/n on input line 36.
LaTeX Font Info:    Redeclaring math symbol \Gamma on input line 47.
LaTeX Font Info:    Redeclaring math symbol \Delta on input line 48.
LaTeX Font Info:    Redeclaring math symbol \Theta on input line 49.
LaTeX Font Info:    Redeclaring math symbol \Lambda on input line 50.
LaTeX Font Info:    Redeclaring math symbol \Xi on input line 51.
LaTeX Font Info:    Redeclaring math symbol \Pi on input line 52.
LaTeX Font Info:    Redeclaring math symbol \Sigma on input line 53.
LaTeX Font Info:    Redeclaring math symbol \Upsilon on input line 54.
LaTeX Font Info:    Redeclaring math symbol \Phi on input line 55.
LaTeX Font Info:    Redeclaring math symbol \Psi on input line 56.
LaTeX Font Info:    Redeclaring math symbol \Omega on input line 57.
\symEulerFraktur=\mathgroup4
LaTeX Font Info:    Overwriting symbol font `EulerFraktur' in version
`bold'
(Font)                  U/euf/m/n --> U/euf/b/n on input line 63.
LaTeX Info: Redefining \oldstylenums on input line 85.
\symEulerScript=\mathgroup5
LaTeX Font Info:    Overwriting symbol font `EulerScript' in version
`bold'
(Font)                  U/eus/m/n --> U/eus/b/n on input line 93.
LaTeX Font Info:    Redeclaring math symbol \aleph on input line 97.
LaTeX Font Info:    Redeclaring math symbol \Re on input line 98.
LaTeX Font Info:    Redeclaring math symbol \Im on input line 99.
LaTeX Font Info:    Redeclaring math delimiter \vert on input line 101.
LaTeX Font Info:    Redeclaring math delimiter \backslash on input line
103.
LaTeX Font Info:    Redeclaring math symbol \neg on input line 106.
LaTeX Font Info:    Redeclaring math symbol \wedge on input line 108.
LaTeX Font Info:    Redeclaring math symbol \vee on input line 110.
LaTeX Font Info:    Redeclaring math symbol \setminus on input line 112.
LaTeX Font Info:    Redeclaring math symbol \sim on input line 113.
LaTeX Font Info:    Redeclaring math symbol \mid on input line 114.
```

```
LaTeX Font Info:    Redeclaring math delimiter \arrowvert on input line
116.
LaTeX Font Info:    Redeclaring math symbol \mathsection on input line
117.
\symEulerExtension=\mathgroup6
LaTeX Font Info:    Redeclaring math symbol \coprod on input line 125.
LaTeX Font Info:    Redeclaring math symbol \prod on input line 125.
LaTeX Font Info:    Redeclaring math symbol \sum on input line 125.
LaTeX Font Info:    Redeclaring math symbol \intop on input line 130.
LaTeX Font Info:    Redeclaring math symbol \ointop on input line 131.
LaTeX Font Info:    Redeclaring math symbol \braceld on input line 132.
LaTeX Font Info:    Redeclaring math symbol \bracerd on input line 133.
LaTeX Font Info:    Redeclaring math symbol \bracelu on input line 134.
LaTeX Font Info:    Redeclaring math symbol \braceru on input line 135.
LaTeX Font Info:    Redeclaring math symbol \infty on input line 136.
LaTeX Font Info:    Redeclaring math symbol \nearrow on input line 153.
LaTeX Font Info:    Redeclaring math symbol \searrow on input line 154.
LaTeX Font Info:    Redeclaring math symbol \nwarrow on input line 155.
LaTeX Font Info:    Redeclaring math symbol \swarrow on input line 156.
LaTeX Font Info:    Redeclaring math symbol \Leftrightarrow on input line
157.
LaTeX Font Info:    Redeclaring math symbol \Leftarrow on input line 158.
LaTeX Font Info:    Redeclaring math symbol \Rightarrow on input line
159.
LaTeX Font Info:    Redeclaring math symbol \leftrightarrow on input line
160.
LaTeX Font Info:    Redeclaring math symbol \leftarrow on input line 161.
LaTeX Font Info:    Redeclaring math symbol \rightarrow on input line
163.
LaTeX Font Info:    Redeclaring math delimiter \uparrow on input line
166.
LaTeX Font Info:    Redeclaring math delimiter \downarrow on input line
168.
LaTeX Font Info:    Redeclaring math delimiter \updownarrow on input line
170.
LaTeX Font Info:    Redeclaring math delimiter \Uparrow on input line
172.
LaTeX Font Info:    Redeclaring math delimiter \Downarrow on input line
174.
LaTeX Font Info:    Redeclaring math delimiter \Updownarrow on input line
176.
LaTeX Font Info:    Redeclaring math symbol \leftharpoonup on input line
177.
LaTeX Font Info:    Redeclaring math symbol \leftharpoondown on input
line 178.

LaTeX Font Info:    Redeclaring math symbol \rightharpoonup on input line
179.
LaTeX Font Info:    Redeclaring math symbol \rightharpoondown on input
line 180
.
LaTeX Font Info:    Redeclaring math delimiter \lbrace on input line 182.
LaTeX Font Info:    Redeclaring math delimiter \rbrace on input line 184.
\symcmmigroup=\mathgroup7
```

```
LaTeX Font Info:    Overwriting symbol font `cmmigroup' in version `bold'
(Font)                  OML/cmm/m/it --> OML/cmm/b/it on input line 200.
LaTeX Font Info:    Redeclaring math accent \vec on input line 201.
LaTeX Font Info:    Redeclaring math symbol \triangleleft on input line
202.
LaTeX Font Info:    Redeclaring math symbol \triangleright on input line
203.
LaTeX Font Info:    Redeclaring math symbol \star on input line 204.
LaTeX Font Info:    Redeclaring math symbol \lhook on input line 205.
LaTeX Font Info:    Redeclaring math symbol \rhook on input line 206.
LaTeX Font Info:    Redeclaring math symbol \flat on input line 207.
LaTeX Font Info:    Redeclaring math symbol \natural on input line 208.
LaTeX Font Info:    Redeclaring math symbol \sharp on input line 209.
LaTeX Font Info:    Redeclaring math symbol \smile on input line 210.
LaTeX Font Info:    Redeclaring math symbol \frown on input line 211.
LaTeX Font Info:    Redeclaring math accent \grave on input line 245.
LaTeX Font Info:    Redeclaring math accent \acute on input line 246.
LaTeX Font Info:    Redeclaring math accent \tilde on input line 247.
LaTeX Font Info:    Redeclaring math accent \ddot on input line 248.
LaTeX Font Info:    Redeclaring math accent \check on input line 249.
LaTeX Font Info:    Redeclaring math accent \breve on input line 250.
LaTeX Font Info:    Redeclaring math accent \bar on input line 251.
LaTeX Font Info:    Redeclaring math accent \dot on input line 252.
LaTeX Font Info:    Redeclaring math accent \hat on input line 254.
) (c:/texlive/2024/texmf-dist/tex/latex/merriweather/merriweather.sty
Package: merriweather 2022/09/20 (Bob Tennent) Supports
Merriweather(Sans) font
s for all LaTeX engines.
(c:/texlive/2024/texmf-dist/tex/generic/iftex/ifluatex.sty
Package: ifluatex 2019/10/25 v1.5 ifluatex legacy package. Use iftex
instead.
) (c:/texlive/2024/texmf-dist/tex/latex/base/textcomp.sty
Package: textcomp 2024/04/24 v2.1b Standard LaTeX package
) (c:/texlive/2024/texmf-dist/tex/latex/xkeyval/xkeyval.sty
Package: xkeyval 2022/06/16 v2.9 package option processing (HA)
(c:/texlive/2024/texmf-dist/tex/generic/xkeyval/xkeyval.tex
(c:/texlive/2024/te
xmf-dist/tex/generic/xkeyval/xkvutils.tex
\XKV@toks=\toks22
\XKV@tempa@toks=\toks23
)
\XKV@depth=\count271
File: xkeyval.tex 2014/12/03 v2.7a key=value parser (HA)
)) (c:/texlive/2024/texmf-dist/tex/latex/base/fontenc.sty
Package: fontenc 2021/04/29 v2.0v Standard LaTeX package
LaTeX Font Info:    Trying to load font information for T1+lmr on input
line 11
6.
(c:/texlive/2024/texmf-dist/tex/latex/lm/t1lmr.fd
File: t1lmr.fd 2015/05/01 v1.6.1 Font defs for Latin Modern
)) (c:/texlive/2024/texmf-dist/tex/latex/fontaxes/fontaxes.sty
Package: fontaxes 2020/07/21 v1.0e Font selection axes
LaTeX Info: Redefining \upshape on input line 29.
LaTeX Info: Redefining \itshape on input line 31.
```

```
LaTeX Info: Redefining \slshape on input line 33.
LaTeX Info: Redefining \swshape on input line 35.
LaTeX Info: Redefining \scshape on input line 37.
LaTeX Info: Redefining \sscshape on input line 39.
LaTeX Info: Redefining \ulcshape on input line 41.
LaTeX Info: Redefining \textsw on input line 47.
LaTeX Info: Redefining \textssc on input line 48.
LaTeX Info: Redefining \textulc on input line 49.
)) (c:/texlive/2024/texmf-dist/tex/latex/mathastext/mathastext.sty
Package: mathastext 2024/07/27 v1.4b Use the text font in math mode (JFB)

Package mathastext Info: Starting the math mode configuration.
\mst@exists@muskip=\muskip17
\mst@forall@muskip=\muskip18
\mst@prime@muskip=\muskip19
\mst@do@nonletters=\toks24
\mst@undo@nonletters=\toks25
\mst@do@easynonletters=\toks26
\mst@undo@easynonletters=\toks27
\symmtoperatorfont=\mathgroup8
\symmtletterfont=\mathgroup9
(   mathastext:   ) ! and ?
(   mathastext:   ) punctuation: , . : ; and \colon
LaTeX Info: Redefining \relbar on input line 1201.
LaTeX Info: Redefining \rightarrowfill on input line 1202.
LaTeX Info: Redefining \leftarrowfill on input line 1205.
(   mathastext:   ) + and =
LaTeX Info: Redefining \Relbar on input line 1298.
(   mathastext:   ) adding = ; and + to \nfss@catcodes
(   mathastext:   ) parentheses ( ) [ ] and slash /
(   mathastext:   ) alldelims: < > \backslash \setminus | \vert \mid \{
\}
LaTeX Font Info:    Redeclaring math symbol \setminus on input line 1364.
LaTeX Info: Redefining \models on input line 1383.
(   mathastext:   ) \# \mathdollar \% \&
(   mathastext:   ) \imath and \jmath
LaTeX Font Info:    Overwriting math alphabet `\Mathnormalbold' in
version `nor
mal'
(Font)                  T1/Merriwthr-OsF/b/it --> T1/Merriwthr-OsF/b/it
on inpu
t line 2863.
LaTeX Font Info:    Overwriting math alphabet `\Mathnormalbold' in
version `bol
d'
(Font)                  T1/Merriwthr-OsF/b/it --> T1/Merriwthr-OsF/b/it
on inpu
t line 2863.
LaTeX Font Info:    Overwriting symbol font `mtletterfont' in version
`normal'
(Font)                  T1/Merriwthr-OsF/m/it --> T1/Merriwthr-OsF/m/it
on inpu
t line 2863.
```

```
LaTeX Font Info:    Overwriting symbol font `mtletterfont' in version
`bold'
(Font)                  T1/Merriwthr-OsF/m/it --> T1/Merriwthr-OsF/b/it
on inpu
t line 2863.
LaTeX Font Info:    Overwriting symbol font `mtoperatorfont' in version
`normal
'
(Font)                  T1/Merriwthr-OsF/m/n --> T1/Merriwthr-OsF/m/n on
input
line 2863.
LaTeX Font Info:    Overwriting symbol font `mtoperatorfont' in version
`bold'
(Font)                  T1/Merriwthr-OsF/m/n --> T1/Merriwthr-OsF/b/n on
input
line 2863.
LaTeX Font Info:    Overwriting math alphabet `\Mathbf' in version
`normal'
(Font)                  T1/Merriwthr-OsF/b/n --> T1/Merriwthr-OsF/b/n on
input
line 2863.
LaTeX Font Info:    Overwriting math alphabet `\Mathbf' in version `bold'
(Font)                  T1/Merriwthr-OsF/b/n --> T1/Merriwthr-OsF/b/n on
input
line 2863.
LaTeX Font Info:    Overwriting math alphabet `\Mathit' in version
`normal'
(Font)                  T1/Merriwthr-OsF/m/it --> T1/Merriwthr-OsF/m/it
on inpu
t line 2863.
LaTeX Font Info:    Overwriting math alphabet `\Mathit' in version `bold'
(Font)                  T1/Merriwthr-OsF/m/it --> T1/Merriwthr-OsF/b/it
on inpu
t line 2863.
LaTeX Font Info:    Overwriting math alphabet `\Mathsf' in version
`normal'
(Font)                  T1/MerriwthrSans-OsF/m/n --> T1/MerriwthrSans-
OsF/m/n o
n input line 2863.
LaTeX Font Info:    Overwriting math alphabet `\Mathsf' in version `bold'
(Font)                  T1/MerriwthrSans-OsF/m/n --> T1/MerriwthrSans-
OsF/b/n o
n input line 2863.
LaTeX Font Info:    Overwriting math alphabet `\Mathtt' in version
`normal'
(Font)                  T1/lmtt/m/n --> T1/lmtt/m/n on input line 2863.
LaTeX Font Info:    Overwriting math alphabet `\Mathtt' in version `bold'
(Font)                  T1/lmtt/m/n --> T1/lmtt/b/n on input line 2863.
(   mathastext:   ) Latin letters in the `normal', resp. `bold',
(   mathastext:   ) math versions are now set up to use the fonts
(   mathastext:   ) T1/Merriwthr-OsF/m/it, resp. T1/Merriwthr-OsF/b/it.
(   mathastext:   ) Other characters (digits, ...) and \log-like names
will be
(   mathastext:   ) typeset with the n shape.
```

```
(   mathastext:   ) \hbar
(   mathastext:   ) minus as endash
(   mathastext:   ) The italic option is in effect.
(   mathastext:   ) \HUGE has been (re)-defined.
(   mathastext:   ) mathastext has declared larger sizes for subscripts.
(   mathastext:   ) To keep LaTeX defaults, use option
`defaultmathsizes'.

Package mathastext Info: Loading is complete.  You can now use
\Mathastext to
(mathastext)             modify the normal and bold math versions.  Use
it
(mathastext)             with optional argument or use \MTDeclareVersion
to
(mathastext)             declare additional math versions.
) (c:/texlive/2024/texmf-dist/tex/latex/relsize/relsize.sty
Package: relsize 2013/03/29 ver 4.1
) (c:/texlive/2024/texmf-dist/tex/latex/ragged2e/ragged2e.sty
Package: ragged2e 2023/06/22 v3.6 ragged2e Package
\CenteringLeftskip=\skip52
\RaggedLeftLeftskip=\skip53
\RaggedRightLeftskip=\skip54
\CenteringRightskip=\skip55
\RaggedLeftRightskip=\skip56
\RaggedRightRightskip=\skip57
\CenteringParfillskip=\skip58
\RaggedLeftParfillskip=\skip59
\RaggedRightParfillskip=\skip60
\JustifyingParfillskip=\skip61
\CenteringParindent=\skip62
\RaggedLeftParindent=\skip63
\RaggedRightParindent=\skip64
\JustifyingParindent=\skip65
) (c:/texlive/2024/texmf-dist/tex/latex/xcolor/xcolor.sty
Package: xcolor 2023/11/15 v3.01 LaTeX color extensions (UK)
(c:/texlive/2024/texmf-dist/tex/latex/graphics-cfg/color.cfg
File: color.cfg 2016/01/02 v1.6 sample color configuration
)
Package xcolor Info: Driver file: pdftex.def on input line 274.
(c:/texlive/2024/texmf-dist/tex/latex/graphics-def/pdftex.def
File: pdftex.def 2024/04/13 v1.2c Graphics/color driver for pdftex
) (c:/texlive/2024/texmf-dist/tex/latex/graphics/mathcolor.ltx)
Package xcolor Info: Model `cmy' substituted by `cmy0' on input line
1350.
Package xcolor Info: Model `hsb' substituted by `rgb' on input line 1354.
Package xcolor Info: Model `RGB' extended on input line 1366.
Package xcolor Info: Model `HTML' substituted by `rgb' on input line
1368.
Package xcolor Info: Model `Hsb' substituted by `hsb' on input line 1369.
Package xcolor Info: Model `tHsb' substituted by `hsb' on input line
1370.
Package xcolor Info: Model `HSB' substituted by `hsb' on input line 1371.
Package xcolor Info: Model `Gray' substituted by `gray' on input line
1372.
```

```
Package xcolor Info: Model `wave' substituted by `hsb' on input line
1373.
) (c:/texlive/2024/texmf-dist/tex/latex/colortbl/colortbl.sty
Package: colortbl 2024/07/06 v1.0i Color table columns (DPC)
(c:/texlive/2024/texmf-dist/tex/latex/tools/array.sty
Package: array 2024/06/14 v2.6d Tabular extension package (FMi)
\col@sep=\dimen143
\ar@mcellbox=\box53
\extrarowheight=\dimen144
\NC@list=\toks28
\extratabsurround=\skip66
\backup@length=\skip67
\ar@cellbox=\box54
)
\everycr=\toks29
\minrowclearance=\skip68
\rownum=\count272
) (c:/texlive/2024/texmf-dist/tex/latex/graphics/graphicx.sty
Package: graphicx 2021/09/16 v1.2d Enhanced LaTeX Graphics (DPC,SPQR)
(c:/texlive/2024/texmf-dist/tex/latex/graphics/graphics.sty
Package: graphics 2024/05/23 v1.4g Standard LaTeX Graphics (DPC,SPQR)
(c:/texlive/2024/texmf-dist/tex/latex/graphics/trig.sty
Package: trig 2023/12/02 v1.11 sin cos tan (DPC)
) (c:/texlive/2024/texmf-dist/tex/latex/graphics-cfg/graphics.cfg
File: graphics.cfg 2016/06/04 v1.11 sample graphics configuration
)
Package graphics Info: Driver file: pdftex.def on input line 106.
)
\Gin@req@height=\dimen145
\Gin@req@width=\dimen146
) (c:/texlive/2024/texmf-dist/tex/latex/xpatch/xpatch.sty
(c:/texlive/2024/texm
f-dist/tex/latex/l3kernel/expl3.sty
Package: expl3 2024-05-27 L3 programming layer (loader)
(c:/texlive/2024/texmf-dist/tex/latex/l3backend/l3backend-pdftex.def
File: l3backend-pdftex.def 2024-05-08 L3 backend support: PDF output
(pdfTeX)
\l__color_backend_stack_int=\count273
\l__pdf_internal_box=\box55
))
Package: xpatch 2020/03/25 v0.3a Extending etoolbox patching commands
(c:/texlive/2024/texmf-dist/tex/latex/l3packages/xparse/xparse.sty
Package: xparse 2024-05-08 L3 Experimental document command parser
)) (c:/texlive/2024/texmf-dist/tex/latex/environ/environ.sty
Package: environ 2014/05/04 v0.3 A new way to define environments
(c:/texlive/2024/texmf-dist/tex/latex/trimspaces/trimspaces.sty
Package: trimspaces 2009/09/17 v1.1 Trim spaces around a token list
)
\@envbody=\toks30
) (c:/texlive/2024/texmf-dist/tex/latex/lastpage/lastpage.sty
Package: lastpage 2024/07/07 v2.1c lastpage: 2.09 or 2e? (HMM)
(c:/texlive/2024/texmf-dist/tex/latex/lastpage/lastpage2e.sty
Package: lastpage2e 2024/07/07 v2.1c Decide which 2e lastpage version to
use (H
```

```
MM)
(c:/texlive/2024/texmf-dist/tex/latex/lastpage/lastpagemodern.sty
Package: lastpagemodern 2024-07-07 v2.1c Refers to last page's name (HMM;
JPG)
\c@lastpagecount=\count274
)
)) (c:/texlive/2024/texmf-dist/tex/latex/graphics/rotating.sty
Package: rotating 2016/08/11 v2.16d rotated objects in LaTeX
(c:/texlive/2024/texmf-dist/tex/latex/base/ifthen.sty
Package: ifthen 2024/03/16 v1.1e Standard LaTeX ifthen package (DPC)
)
\c@r@tfl@t=\count275
\rotFPtop=\skip69
\rotFPbot=\skip70
\rot@float@box=\box56
\rot@mess@toks=\toks31
) (c:/texlive/2024/texmf-dist/tex/latex/graphics/lscape.sty
Package: lscape 2020/05/28 v3.02 Landscape Pages (DPC)
) (c:/texlive/2024/texmf-dist/tex/latex/tools/afterpage.sty
Package: afterpage 2023/07/04 v1.08 After-Page Package (DPC)
\AP@output=\toks32
\AP@partial=\box57
\AP@footins=\box58
) (c:/texlive/2024/texmf-dist/tex/latex/textpos/textpos.sty
Package: textpos 2022/07/23 v1.10.1
Package textpos Info: choosing support for LaTeX3 on input line 60.
\TP@textbox=\box59
\TP@holdbox=\box60
\TPHorizModule=\dimen147
\TPVertModule=\dimen148
\TP@margin=\dimen149
\TP@absmargin=\dimen150
Grid set 16 x 16 = 37.34424pt x 52.81541pt
\TPboxrulesize=\dimen151
\TP@ox=\dimen152
\TP@oy=\dimen153
\TP@tbargs=\toks33
TextBlockOrigin set to 0pt x 0pt
) (c:/texlive/2024/texmf-dist/tex/latex/url/url.sty
\Urlmuskip=\muskip20
Package: url 2013/09/16  ver 3.4  Verb mode for urls, etc.
) (c:/texlive/2024/texmf-dist/tex/latex/newfloat/newfloat.sty
Package: newfloat 2023/10/01 v1.2 Defining new floating environments (AR)
Package newfloat Info: `rotating' package detected.
) (c:/texlive/2024/texmf-dist/tex/latex/mdframed/mdframed.sty
Package: mdframed 2013/07/01 1.9b: mdframed
(c:/texlive/2024/texmf-dist/tex/latex/kvoptions/kvoptions.sty
Package: kvoptions 2022-06-15 v3.15 Key value format for package options
(HO)
(c:/texlive/2024/texmf-dist/tex/generic/ltxcmds/ltxcmds.sty
Package: ltxcmds 2023-12-04 v1.26 LaTeX kernel commands for general use
(HO)
) (c:/texlive/2024/texmf-dist/tex/latex/kvsetkeys/kvsetkeys.sty
Package: kvsetkeys 2022-10-05 v1.19 Key value parser (HO)
```

```
)) (c:/texlive/2024/texmf-dist/tex/latex/zref/zref-abspage.sty
Package: zref-abspage 2023-09-14 v2.35 Module abspage for zref (HO)
(c:/texlive/2024/texmf-dist/tex/latex/zref/zref-base.sty
Package: zref-base 2023-09-14 v2.35 Module base for zref (HO)
(c:/texlive/2024/texmf-dist/tex/generic/infwarerr/infwarerr.sty
Package: infwarerr 2019/12/03 v1.5 Providing info/warning/error messages
(HO)
) (c:/texlive/2024/texmf-dist/tex/generic/kvdefinekeys/kvdefinekeys.sty
Package: kvdefinekeys 2019-12-19 v1.6 Define keys (HO)
) (c:/texlive/2024/texmf-dist/tex/generic/pdftexcmds/pdftexcmds.sty
Package: pdftexcmds 2020-06-27 v0.33 Utility functions of pdfTeX for
LuaTeX (HO
)
Package pdftexcmds Info: \pdf@primitive is available.
Package pdftexcmds Info: \pdf@ifprimitive is available.
Package pdftexcmds Info: \pdfdraftmode found.
) (c:/texlive/2024/texmf-dist/tex/generic/etexcmds/etexcmds.sty
Package: etexcmds 2019/12/15 v1.7 Avoid name clashes with e-TeX commands
(HO)
) (c:/texlive/2024/texmf-dist/tex/latex/auxhook/auxhook.sty
Package: auxhook 2019-12-17 v1.6 Hooks for auxiliary files (HO)
)
Package zref Info: New property list: main on input line 767.
Package zref Info: New property: default on input line 768.
Package zref Info: New property: page on input line 769.
)
\c@abspage=\count276
Package zref Info: New property: abspage on input line 67.
) (c:/texlive/2024/texmf-dist/tex/latex/needspace/needspace.sty
Package: needspace 2010/09/12 v1.3d reserve vertical space
)
\mdf@templength=\skip71
\c@mdf@globalstyle@cnt=\count277
\mdf@skipabove@length=\skip72
\mdf@skipbelow@length=\skip73
\mdf@leftmargin@length=\skip74
\mdf@rightmargin@length=\skip75
\mdf@innerleftmargin@length=\skip76
\mdf@innerrightmargin@length=\skip77
\mdf@innertopmargin@length=\skip78
\mdf@innerbottommargin@length=\skip79
\mdf@splittopskip@length=\skip80
\mdf@splitbottomskip@length=\skip81
\mdf@outermargin@length=\skip82
\mdf@innermargin@length=\skip83
\mdf@linewidth@length=\skip84
\mdf@innerlinewidth@length=\skip85
\mdf@middlelinewidth@length=\skip86
\mdf@outerlinewidth@length=\skip87
\mdf@roundcorner@length=\skip88
\mdf@footenotedistance@length=\skip89
\mdf@userdefinedwidth@length=\skip90
\mdf@needspace@length=\skip91
\mdf@frametitleaboveskip@length=\skip92
```

```
\mdf@frametitlebelowskip@length=\skip93
\mdf@frametitlerulewidth@length=\skip94
\mdf@frametitleleftmargin@length=\skip95
\mdf@frametitlerightmargin@length=\skip96
\mdf@shadowsize@length=\skip97
\mdf@extratopheight@length=\skip98
\mdf@subtitleabovelinewidth@length=\skip99
\mdf@subtitlebelowlinewidth@length=\skip100
\mdf@subtitleaboveskip@length=\skip101
\mdf@subtitlebelowskip@length=\skip102
\mdf@subtitleinneraboveskip@length=\skip103
\mdf@subtitleinnerbelowskip@length=\skip104
\mdf@subsubtitleabovelinewidth@length=\skip105
\mdf@subsubtitlebelowlinewidth@length=\skip106
\mdf@subsubtitleaboveskip@length=\skip107
\mdf@subsubtitlebelowskip@length=\skip108
\mdf@subsubtitleinneraboveskip@length=\skip109
\mdf@subsubtitleinnerbelowskip@length=\skip110
(c:/texlive/2024/texmf-dist/tex/latex/mdframed/md-frame-0.mdf
File: md-frame-0.mdf 2013/07/01\ 1.9b: md-frame-0
)
\mdf@frametitlebox=\box61
\mdf@footnotebox=\box62
\mdf@splitbox@one=\box63
\mdf@splitbox@two=\box64
\mdf@splitbox@save=\box65
\mdfsplitboxwidth=\skip111
\mdfsplitboxtotalwidth=\skip112
\mdfsplitboxheight=\skip113
\mdfsplitboxdepth=\skip114
\mdfsplitboxtotalheight=\skip115
\mdfframetitleboxwidth=\skip116
\mdfframetitleboxtotalwidth=\skip117
\mdfframetitleboxheight=\skip118
\mdfframetitleboxdepth=\skip119
\mdfframetitleboxtotalheight=\skip120
\mdffootnoteboxwidth=\skip121
\mdffootnoteboxtotalwidth=\skip122
\mdffootnoteboxheight=\skip123
\mdffootnoteboxdepth=\skip124
\mdffootnoteboxtotalheight=\skip125
\mdftotallinewidth=\skip126
\mdfboundingboxwidth=\skip127
\mdfboundingboxtotalwidth=\skip128
\mdfboundingboxheight=\skip129
\mdfboundingboxdepth=\skip130
\mdfboundingboxtotalheight=\skip131
\mdf@freevspace@length=\skip132
\mdf@horizontalwidthofbox@length=\skip133
\mdf@verticalmarginwhole@length=\skip134
\mdf@horizontalspaceofbox=\skip135
\mdfsubtitleheight=\skip136
\mdfsubsubtitleheight=\skip137
\c@mdfcountframes=\count278
```

```
****** mdframed patching \endmdf@trivlist

****** -- success******

\mdf@envdepth=\count279
\c@mdf@env@i=\count280
\c@mdf@env@ii=\count281
\c@mdf@zref@counter=\count282
Package zref Info: New property: mdf@pagevalue on input line 895.
) (c:/texlive/2024/texmf-dist/tex/latex/titlesec/titlesec.sty
Package: titlesec 2023/10/27 v2.16 Sectioning titles
\ttl@box=\box66
\beforetitleunit=\skip138
\aftertitleunit=\skip139
\ttl@plus=\dimen154
\ttl@minus=\dimen155
\ttl@toksa=\toks34
\titlewidth=\dimen156
\titlewidthlast=\dimen157
\titlewidthfirst=\dimen158
) (c:/texlive/2024/texmf-dist/tex/latex/koma-script/scrextend.sty
Package: scrextend 2023/07/07 v3.41 KOMA-Script package (extend other
classes w
ith features of KOMA-Script classes)
(c:/texlive/2024/texmf-dist/tex/latex/koma-script/scrkbase.sty
Package: scrkbase 2023/07/07 v3.41 KOMA-Script package (KOMA-Script-
dependent b
asics and keyval usage)
(c:/texlive/2024/texmf-dist/tex/latex/koma-script/scrbase.sty
Package: scrbase 2023/07/07 v3.41 KOMA-Script package (KOMA-Script-
independent
basics and keyval usage)
(c:/texlive/2024/texmf-dist/tex/latex/koma-script/scrlfile.sty
Package: scrlfile 2023/07/07 v3.41 KOMA-Script package (file load hooks)
(c:/texlive/2024/texmf-dist/tex/latex/koma-script/scrlfile-hook.sty
Package: scrlfile-hook 2023/07/07 v3.41 KOMA-Script package (using LaTeX
hooks)

(c:/texlive/2024/texmf-dist/tex/latex/koma-script/scrlogo.sty
Package: scrlogo 2023/07/07 v3.41 KOMA-Script package (logo)
)))
Applying: [2021/05/01] Usage of raw or classic option list on input line
252.
Already applied: [0000/00/00] Usage of raw or classic option list on
input line
 368.
))
Package scrextend Info: unexpected definition of `\@makefnmark'.
(scrextend)             Trying to patch it on input line 1762.
Package scrextend Info: patch seems to be successfull on input line 1762.
) (c:/texlive/2024/texmf-dist/tex/latex/tools/calc.sty
Package: calc 2023/07/08 v4.3 Infix arithmetic (KKT,FJ)
\calc@Acount=\count283
```

```
\calc@Bcount=\count284
\calc@Adimen=\dimen159
\calc@Bdimen=\dimen160
\calc@Askip=\skip140
\calc@Bskip=\skip141
LaTeX Info: Redefining \setlength on input line 80.
LaTeX Info: Redefining \addtolength on input line 81.
\calc@Ccount=\count285
\calc@Cskip=\skip142
) (c:/texlive/2024/texmf-dist/tex/latex/geometry/geometry.sty
Package: geometry 2020/01/02 v5.9 Page Geometry
(c:/texlive/2024/texmf-dist/tex/generic/iftex/ifvtex.sty
Package: ifvtex 2019/10/25 v1.7 ifvtex legacy package. Use iftex instead.
)
\Gm@cnth=\count286
\Gm@cntv=\count287
\c@Gm@tempcnt=\count288
\Gm@bindingoffset=\dimen161
\Gm@wd@mp=\dimen162
\Gm@odd@mp=\dimen163
\Gm@even@mp=\dimen164
\Gm@layoutwidth=\dimen165
\Gm@layoutheight=\dimen166
\Gm@layouthoffset=\dimen167
\Gm@layoutvoffset=\dimen168
\Gm@dimlist=\toks35
) (c:/texlive/2024/texmf-dist/tex/latex/preprint/authblk.sty
Package: authblk 2001/02/27 1.3 (PWD)
\affilsep=\skip143
\@affilsep=\skip144
\c@Maxaffil=\count289
\c@authors=\count290
\c@affil=\count291
) (c:/texlive/2024/texmf-dist/tex/latex/footmisc/footmisc.sty
Package: footmisc 2023/07/05 v6.0f a miscellany of footnote facilities
\FN@temptoken=\toks36
\footnotemargin=\dimen169
\@outputbox@depth=\dimen170
Package footmisc Info: Declaring symbol style bringhurst on input line
696.
Package footmisc Info: Declaring symbol style chicago on input line 704.
Package footmisc Info: Declaring symbol style wiley on input line 713.
Package footmisc Info: Declaring symbol style lamport-robust on input
line 724.

Package footmisc Info: Declaring symbol style lamport* on input line 744.
Package footmisc Info: Declaring symbol style lamport*-robust on input
line 765
.
) (c:/texlive/2024/texmf-dist/tex/latex/fancyhdr/fancyhdr.sty
Package: fancyhdr 2024/07/23 v4.3.1 Extensive control of page headers and
foote
rs
\f@nch@headwidth=\skip145
```

```
\f@nch@O@elh=\skip146
\f@nch@O@erh=\skip147
\f@nch@O@olh=\skip148
\f@nch@O@orh=\skip149
\f@nch@O@elf=\skip150
\f@nch@O@erf=\skip151
\f@nch@O@olf=\skip152
\f@nch@O@orf=\skip153
) (c:/texlive/2024/texmf-dist/tex/generic/alphalph/alphalph.sty
Package: alphalph 2019/12/09 v2.6 Convert numbers to letters (HO)
(c:/texlive/2024/texmf-dist/tex/generic/intcalc/intcalc.sty
Package: intcalc 2019/12/15 v1.3 Expandable calculations with integers
(HO)
))
\c@authorfn=\count292
(c:/texlive/2024/texmf-dist/tex/latex/abstract/abstract.sty
Package: abstract 2009/06/08 v1.2a configurable abstracts
\abstitleskip=\skip154
\absleftindent=\skip155
\absrightindent=\skip156
\absparindent=\skip157
\absparsep=\skip158
)
Package newfloat Info: New float `keypoints' with options
`placement=t!,name=kp
t' on input line 304.
\c@keypoints=\count293
\newfloat@ftype=\count294
Package newfloat Info: float type `keypoints'=8 on input line 304.
(c:/texlive/2024/texmf-dist/tex/latex/enumitem/enumitem.sty
Package: enumitem 2019/06/20 v3.9 Customized lists
\labelindent=\skip159
\enit@outerparindent=\dimen171
\enit@toks=\toks37
\enit@inbox=\box67
\enit@count@id=\count295
\enitdp@description=\count296
) (c:/texlive/2024/texmf-dist/tex/latex/quoting/quoting.sty
Package: quoting 2014/01/28 v0.1c Consolidated environment for displayed
text
\quo@toppartop=\skip160
) (c:/texlive/2024/texmf-dist/tex/latex/sttools/stfloats.sty
Package: stfloats 2017/03/27 v3.3 Improve float mechanism and
baselineskip sett
ings
\@dblbotnum=\count297
\c@dblbotnumber=\count298
) (c:/texlive/2024/texmf-dist/tex/latex/booktabs/booktabs.sty
Package: booktabs 2020/01/12 v1.61803398 Publication quality tables
\heavyrulewidth=\dimen172
\lightrulewidth=\dimen173
\cmidrulewidth=\dimen174
\belowrulesep=\dimen175
\belowbottomsep=\dimen176
```

```
\aboverulesep=\dimen177
\abovetopsep=\dimen178
\cmidrulesep=\dimen179
\cmidrulekern=\dimen180
\defaultaddspace=\dimen181
\@cmidla=\count299
\@cmidlb=\count300
\@aboverulesep=\dimen182
\@belowrulesep=\dimen183
\@thisruleclass=\count301
\@lastruleclass=\count302
\@thisrulewidth=\dimen184
) (c:/texlive/2024/texmf-dist/tex/latex/tools/tabularx.sty
Package: tabularx 2023/12/11 v2.12a `tabularx' package (DPC)
\TX@col@width=\dimen185
\TX@old@table=\dimen186
\TX@old@col=\dimen187
\TX@target=\dimen188
\TX@delta=\dimen189
\TX@cols=\count303
\TX@ftn=\toks38
)
\enitdp@tablenotes=\count304
(c:/texlive/2024/texmf-dist/tex/latex/caption/caption.sty
Package: caption 2023/08/05 v3.6o Customizing captions (AR)
(c:/texlive/2024/texmf-dist/tex/latex/caption/caption3.sty
Package: caption3 2023/07/31 v2.4d caption3 kernel (AR)
\caption@tempdima=\dimen190
\captionmargin=\dimen191
\caption@leftmargin=\dimen192
\caption@rightmargin=\dimen193
\caption@width=\dimen194
\caption@indent=\dimen195
\caption@parindent=\dimen196
\caption@hangindent=\dimen197
Package caption Info: Standard document class detected.
)
\c@caption@flags=\count305
\c@continuedfloat=\count306
Package caption Info: rotating package is loaded.
Package caption Info: scrextend package is loaded.
\caption@addmargin@hsize=\dimen198
\caption@addmargin@linewidth=\dimen199
) (c:/texlive/2024/texmf-dist/tex/latex/natbib/natbib.sty
Package: natbib 2010/09/13 8.31b (PWD, AO)
\bibhang=\skip161
\bibsep=\skip162
LaTeX Info: Redefining \cite on input line 694.
\c@NAT@ctr=\count307
)) (c:/texlive/2024/texmf-dist/tex/latex/hyperref/hyperref.sty
Package: hyperref 2024-07-10 v7.01j Hypertext links for LaTeX
(c:/texlive/2024/texmf-dist/tex/generic/pdfescape/pdfescape.sty
Package: pdfescape 2019/12/09 v1.15 Implements pdfTeX's escape features
(HO)
```

```
) (c:/texlive/2024/texmf-dist/tex/latex/hycolor/hycolor.sty
Package: hycolor 2020-01-27 v1.10 Color options for hyperref/bookmark
(HO)
) (c:/texlive/2024/texmf-dist/tex/latex/hyperref/nameref.sty
Package: nameref 2023-11-26 v2.56 Cross-referencing by name of section
(c:/texlive/2024/texmf-dist/tex/latex/refcount/refcount.sty
Package: refcount 2019/12/15 v3.6 Data extraction from label references
(HO)
) (c:/texlive/2024/texmf-
dist/tex/generic/gettitlestring/gettitlestring.sty
Package: gettitlestring 2019/12/15 v1.6 Cleanup title references (HO)
)
\c@section@level=\count308
) (c:/texlive/2024/texmf-dist/tex/generic/stringenc/stringenc.sty
Package: stringenc 2019/11/29 v1.12 Convert strings between diff.
encodings (HO
)
)
\@linkdim=\dimen256
\Hy@linkcounter=\count309
\Hy@pagecounter=\count310
(c:/texlive/2024/texmf-dist/tex/latex/hyperref/pd1enc.def
File: pd1enc.def 2024-07-10 v7.01j Hyperref: PDFDocEncoding definition
(HO)
Now handling font encoding PD1 ...
... no UTF-8 mapping file for font encoding PD1
)
\Hy@SavedSpaceFactor=\count311
(c:/texlive/2024/texmf-dist/tex/latex/hyperref/puenc.def
File: puenc.def 2024-07-10 v7.01j Hyperref: PDF Unicode definition (HO)
Now handling font encoding PU ...
... no UTF-8 mapping file for font encoding PU
)
Package hyperref Info: Hyper figures OFF on input line 4157.
Package hyperref Info: Link nesting OFF on input line 4162.
Package hyperref Info: Hyper index ON on input line 4165.
Package hyperref Info: Plain pages OFF on input line 4172.
Package hyperref Info: Backreferencing OFF on input line 4177.
Package hyperref Info: Implicit mode ON; LaTeX internals redefined.
Package hyperref Info: Bookmarks ON on input line 4424.
\c@Hy@tempcnt=\count312
LaTeX Info: Redefining \url on input line 4763.
\XeTeXLinkMargin=\dimen257
(c:/texlive/2024/texmf-dist/tex/generic/bitset/bitset.sty
Package: bitset 2019/12/09 v1.3 Handle bit-vector datatype (HO)
(c:/texlive/2024/texmf-dist/tex/generic/bigintcalc/bigintcalc.sty
Package: bigintcalc 2019/12/15 v1.5 Expandable calculations on big
integers (HO
)
))
\Fld@menulength=\count313
\Field@Width=\dimen258
\Fld@charsize=\dimen259
Package hyperref Info: Hyper figures OFF on input line 6042.
```

```
Package hyperref Info: Link nesting OFF on input line 6047.
Package hyperref Info: Hyper index ON on input line 6050.
Package hyperref Info: backreferencing OFF on input line 6057.
Package hyperref Info: Link coloring OFF on input line 6062.
Package hyperref Info: Link coloring with OCG OFF on input line 6067.
Package hyperref Info: PDF/A mode OFF on input line 6072.
(c:/texlive/2024/texmf-dist/tex/latex/base/atbegshi-ltx.sty
Package: atbegshi-ltx 2021/01/10 v1.0c Emulation of the original atbegshi
package with kernel methods
)
\Hy@abspage=\count314
\c@Item=\count315
\c@Hfootnote=\count316
)
Package hyperref Info: Driver (autodetected): hpdftex.
(c:/texlive/2024/texmf-dist/tex/latex/hyperref/hpdftex.def
File: hpdftex.def 2024-07-10 v7.01j Hyperref driver for pdfTeX
(c:/texlive/2024/texmf-dist/tex/latex/base/atveryend-ltx.sty
Package: atveryend-ltx 2020/08/19 v1.0a Emulation of the original
atveryend pac
kage
with kernel methods
)
\HyAnn@Count=\count317
\Fld@listcount=\count318
\c@bookmark@seq@number=\count319
(c:/texlive/2024/texmf-dist/tex/latex/rerunfilecheck/rerunfilecheck.sty
Package: rerunfilecheck 2022-07-10 v1.10 Rerun checks for auxiliary files
(HO)
(c:/texlive/2024/texmf-dist/tex/generic/uniquecounter/uniquecounter.sty
Package: uniquecounter 2019/12/15 v1.4 Provide unlimited unique counter
(HO)
)
Package uniquecounter Info: New unique counter `rerunfilecheck' on input
line 2
85.
)
\Hy@SectionHShift=\skip163
) (c:/texlive/2024/texmf-dist/tex/latex/anyfontsize/anyfontsize.sty
Package: anyfontsize 2007/11/22 anyfontsize.sty by pts
) (c:/texlive/2024/texmf-dist/tex/latex/siunitx/siunitx.sty
Package: siunitx 2024-06-24 v3.3.19 A comprehensive (SI) units package
\l__siunitx_number_uncert_offset_int=\count320
\l__siunitx_number_exponent_fixed_int=\count321
\l__siunitx_number_min_decimal_int=\count322
\l__siunitx_number_min_integer_int=\count323
\l__siunitx_number_round_precision_int=\count324
\l__siunitx_number_lower_threshold_int=\count325
\l__siunitx_number_upper_threshold_int=\count326
\l__siunitx_number_group_first_int=\count327
\l__siunitx_number_group_size_int=\count328
\l__siunitx_number_group_minimum_int=\count329
\l__siunitx_angle_tmp_dim=\dimen260
\l__siunitx_angle_marker_box=\box68
```

```
\l__siunitx_angle_unit_box=\box69
\l__siunitx_compound_count_int=\count330
(c:/texlive/2024/texmf-dist/tex/latex/translations/translations.sty
Package: translations 2022/02/05 v1.12 internationalization of LaTeX2e
packages
 (CN)
) (c:/texlive/2024/texmf-dist/tex/latex/amsmath/amstext.sty
Package: amstext 2021/08/26 v2.01 AMS text
(c:/texlive/2024/texmf-dist/tex/latex/amsmath/amsgen.sty
File: amsgen.sty 1999/11/30 v2.0 generic functions
\@emptytoks=\toks39
\ex@=\dimen261
))
\l__siunitx_table_tmp_box=\box70
\l__siunitx_table_tmp_dim=\dimen262
\l__siunitx_table_column_width_dim=\dimen263
\l__siunitx_table_integer_box=\box71
\l__siunitx_table_decimal_box=\box72
\l__siunitx_table_uncert_box=\box73
\l__siunitx_table_before_box=\box74
\l__siunitx_table_after_box=\box75
\l__siunitx_table_before_dim=\dimen264
\l__siunitx_table_carry_dim=\dimen265
\l__siunitx_unit_tmp_int=\count331
\l__siunitx_unit_position_int=\count332
\l__siunitx_unit_total_int=\count333
) (c:/texlive/2024/texmf-dist/tex/latex/makecell/makecell.sty
Package: makecell 2009/08/03 V0.1e Managing of Tab Column Heads and Cells
\rotheadsize=\dimen266
\c@nlinenum=\count334
\TeXr@lab=\toks40
)
\c@mainfigure=\count335
(c:/texlive/2024/texmf-dist/tex/latex/caption/subcaption.sty
Package: subcaption 2023/07/28 v1.6b Sub-captions (AR)
Package caption Info: New subtype `subfigure' on input line 238.
\c@subfigure=\count336
Package caption Info: New subtype `subtable' on input line 238.
\c@subtable=\count337
Package caption Info: New subtype `subkeypoints' on input line 238.
\c@subkeypoints=\count338
)
\c@suppfigure=\count339
(c:/texlive/2024/texmf-dist/tex/generic/ulem/ulem.sty
\UL@box=\box76
\UL@hyphenbox=\box77
\UL@skip=\skip164
\UL@hook=\toks41
\UL@height=\dimen267
\UL@pe=\count340
\UL@pixel=\dimen268
\ULC@box=\box78
Package: ulem 2019/11/18
\ULdepth=\dimen269
```

```
) (c:/texlive/2024/texmf-dist/tex/latex/orcidlink/orcidlink.sty
Package: orcidlink 2024/06/26 v1.1.0 Support ORCID's three different ID
formats
.
(c:/texlive/2024/texmf-dist/tex/latex/pgf/frontendlayer/tikz.sty
(c:/texlive/20
24/texmf-dist/tex/latex/pgf/basiclayer/pgf.sty (c:/texlive/2024/texmf-
dist/tex/
latex/pgf/utilities/pgfrcs.sty (c:/texlive/2024/texmf-
dist/tex/generic/pgf/util
ities/pgfutil-common.tex
\pgfutil@everybye=\toks42
\pgfutil@tempdima=\dimen270
\pgfutil@tempdimb=\dimen271
) (c:/texlive/2024/texmf-dist/tex/generic/pgf/utilities/pgfutil-latex.def
\pgfutil@abb=\box79
) (c:/texlive/2024/texmf-dist/tex/generic/pgf/utilities/pgfrcs.code.tex
(c:/tex
live/2024/texmf-dist/tex/generic/pgf/pgf.revision.tex)
Package: pgfrcs 2023-01-15 v3.1.10 (3.1.10)
))
Package: pgf 2023-01-15 v3.1.10 (3.1.10)
(c:/texlive/2024/texmf-dist/tex/latex/pgf/basiclayer/pgfcore.sty
(c:/texlive/20
24/texmf-dist/tex/latex/pgf/systemlayer/pgfsys.sty
(c:/texlive/2024/texmf-dist/
tex/generic/pgf/systemlayer/pgfsys.code.tex
Package: pgfsys 2023-01-15 v3.1.10 (3.1.10)
(c:/texlive/2024/texmf-dist/tex/generic/pgf/utilities/pgfkeys.code.tex
\pgfkeys@pathtoks=\toks43
\pgfkeys@temptoks=\toks44

(c:/texlive/2024/texmf-
dist/tex/generic/pgf/utilities/pgfkeyslibraryfiltered.co
de.tex
\pgfkeys@tmptoks=\toks45
))
\pgf@x=\dimen272
\pgf@y=\dimen273
\pgf@xa=\dimen274
\pgf@ya=\dimen275
\pgf@xb=\dimen276
\pgf@yb=\dimen277
\pgf@xc=\dimen278
\pgf@yc=\dimen279
\pgf@xd=\dimen280
\pgf@yd=\dimen281
\w@pgf@writea=\write3
\r@pgf@reada=\read2
\c@pgf@counta=\count341
\c@pgf@countb=\count342
\c@pgf@countc=\count343
\c@pgf@countd=\count344
\t@pgf@toka=\toks46
```

```
\t@pgf@tokb=\toks47
\t@pgf@tokc=\toks48
\pgf@sys@id@count=\count345
(c:/texlive/2024/texmf-dist/tex/generic/pgf/systemlayer/pgf.cfg
File: pgf.cfg 2023-01-15 v3.1.10 (3.1.10)
)
Driver file for pgf: pgfsys-pdftex.def
(c:/texlive/2024/texmf-dist/tex/generic/pgf/systemlayer/pgfsys-pdftex.def
File: pgfsys-pdftex.def 2023-01-15 v3.1.10 (3.1.10)
(c:/texlive/2024/texmf-dist/tex/generic/pgf/systemlayer/pgfsys-common-
pdf.def
File: pgfsys-common-pdf.def 2023-01-15 v3.1.10 (3.1.10)
)))
(c:/texlive/2024/texmf-
dist/tex/generic/pgf/systemlayer/pgfsyssoftpath.code.tex
File: pgfsyssoftpath.code.tex 2023-01-15 v3.1.10 (3.1.10)
\pgfsyssoftpath@smallbuffer@items=\count346
\pgfsyssoftpath@bigbuffer@items=\count347
)
(c:/texlive/2024/texmf-
dist/tex/generic/pgf/systemlayer/pgfsysprotocol.code.tex
File: pgfsysprotocol.code.tex 2023-01-15 v3.1.10 (3.1.10)
)) (c:/texlive/2024/texmf-
dist/tex/generic/pgf/basiclayer/pgfcore.code.tex
Package: pgfcore 2023-01-15 v3.1.10 (3.1.10)
(c:/texlive/2024/texmf-dist/tex/generic/pgf/math/pgfmath.code.tex
(c:/texlive/2
024/texmf-dist/tex/generic/pgf/math/pgfmathutil.code.tex)
(c:/texlive/2024/texm
f-dist/tex/generic/pgf/math/pgfmathparser.code.tex
\pgfmath@dimen=\dimen282
\pgfmath@count=\count348
\pgfmath@box=\box80
\pgfmath@toks=\toks49
\pgfmath@stack@operand=\toks50
\pgfmath@stack@operation=\toks51
) (c:/texlive/2024/texmf-
dist/tex/generic/pgf/math/pgfmathfunctions.code.tex)
(c:/texlive/2024/texmf-
dist/tex/generic/pgf/math/pgfmathfunctions.basic.code.te
x)
(c:/texlive/2024/texmf-
dist/tex/generic/pgf/math/pgfmathfunctions.trigonometric
.code.tex)
(c:/texlive/2024/texmf-
dist/tex/generic/pgf/math/pgfmathfunctions.random.code.t
ex)
(c:/texlive/2024/texmf-
dist/tex/generic/pgf/math/pgfmathfunctions.comparison.co
de.tex)
(c:/texlive/2024/texmf-
dist/tex/generic/pgf/math/pgfmathfunctions.base.code.tex
)
```

```
(c:/texlive/2024/texmf-
dist/tex/generic/pgf/math/pgfmathfunctions.round.code.te
x)
(c:/texlive/2024/texmf-
dist/tex/generic/pgf/math/pgfmathfunctions.misc.code.tex
)
(c:/texlive/2024/texmf-
dist/tex/generic/pgf/math/pgfmathfunctions.integerarithm
etics.code.tex) (c:/texlive/2024/texmf-
dist/tex/generic/pgf/math/pgfmathcalc.co
de.tex) (c:/texlive/2024/texmf-
dist/tex/generic/pgf/math/pgfmathfloat.code.tex
\c@pgfmathroundto@lastzeros=\count349
)) (c:/texlive/2024/texmf-dist/tex/generic/pgf/math/pgfint.code.tex)
(c:/texliv
e/2024/texmf-dist/tex/generic/pgf/basiclayer/pgfcorepoints.code.tex
File: pgfcorepoints.code.tex 2023-01-15 v3.1.10 (3.1.10)
\pgf@picminx=\dimen283
\pgf@picmaxx=\dimen284
\pgf@picminy=\dimen285
\pgf@picmaxy=\dimen286
\pgf@pathminx=\dimen287
\pgf@pathmaxx=\dimen288
\pgf@pathminy=\dimen289
\pgf@pathmaxy=\dimen290
\pgf@xx=\dimen291
\pgf@xy=\dimen292
\pgf@yx=\dimen293
\pgf@yy=\dimen294
\pgf@zx=\dimen295
\pgf@zy=\dimen296
)
(c:/texlive/2024/texmf-
dist/tex/generic/pgf/basiclayer/pgfcorepathconstruct.cod
e.tex
File: pgfcorepathconstruct.code.tex 2023-01-15 v3.1.10 (3.1.10)
\pgf@path@lastx=\dimen297
\pgf@path@lasty=\dimen298
)
(c:/texlive/2024/texmf-
dist/tex/generic/pgf/basiclayer/pgfcorepathusage.code.te
x
File: pgfcorepathusage.code.tex 2023-01-15 v3.1.10 (3.1.10)
\pgf@shorten@end@additional=\dimen299
\pgf@shorten@start@additional=\dimen300
) (c:/texlive/2024/texmf-
dist/tex/generic/pgf/basiclayer/pgfcorescopes.code.tex
File: pgfcorescopes.code.tex 2023-01-15 v3.1.10 (3.1.10)
\pgfpic=\box81
\pgf@hbox=\box82
\pgf@layerbox@main=\box83
\pgf@picture@serial@count=\count350
)
```

```
(c:/texlive/2024/texmf-
dist/tex/generic/pgf/basiclayer/pgfcoregraphicstate.code
.tex
File: pgfcoregraphicstate.code.tex 2023-01-15 v3.1.10 (3.1.10)
\pgflinewidth=\dimen301
)
(c:/texlive/2024/texmf-
dist/tex/generic/pgf/basiclayer/pgfcoretransformations.c
ode.tex
File: pgfcoretransformations.code.tex 2023-01-15 v3.1.10 (3.1.10)
\pgf@pt@x=\dimen302
\pgf@pt@y=\dimen303
\pgf@pt@temp=\dimen304
) (c:/texlive/2024/texmf-
dist/tex/generic/pgf/basiclayer/pgfcorequick.code.tex
File: pgfcorequick.code.tex 2023-01-15 v3.1.10 (3.1.10)
) (c:/texlive/2024/texmf-
dist/tex/generic/pgf/basiclayer/pgfcoreobjects.code.te
x
File: pgfcoreobjects.code.tex 2023-01-15 v3.1.10 (3.1.10)
)
(c:/texlive/2024/texmf-
dist/tex/generic/pgf/basiclayer/pgfcorepathprocessing.co
de.tex
File: pgfcorepathprocessing.code.tex 2023-01-15 v3.1.10 (3.1.10)
) (c:/texlive/2024/texmf-
dist/tex/generic/pgf/basiclayer/pgfcorearrows.code.tex
File: pgfcorearrows.code.tex 2023-01-15 v3.1.10 (3.1.10)
\pgfarrowsep=\dimen305
) (c:/texlive/2024/texmf-
dist/tex/generic/pgf/basiclayer/pgfcoreshade.code.tex
File: pgfcoreshade.code.tex 2023-01-15 v3.1.10 (3.1.10)
\pgf@max=\dimen306
\pgf@sys@shading@range@num=\count351
\pgf@shadingcount=\count352
) (c:/texlive/2024/texmf-
dist/tex/generic/pgf/basiclayer/pgfcoreimage.code.tex
File: pgfcoreimage.code.tex 2023-01-15 v3.1.10 (3.1.10)
)
(c:/texlive/2024/texmf-
dist/tex/generic/pgf/basiclayer/pgfcoreexternal.code.tex
File: pgfcoreexternal.code.tex 2023-01-15 v3.1.10 (3.1.10)
\pgfexternal@startupbox=\box84
) (c:/texlive/2024/texmf-
dist/tex/generic/pgf/basiclayer/pgfcorelayers.code.tex
File: pgfcorelayers.code.tex 2023-01-15 v3.1.10 (3.1.10)
)
(c:/texlive/2024/texmf-
dist/tex/generic/pgf/basiclayer/pgfcoretransparency.code
.tex
File: pgfcoretransparency.code.tex 2023-01-15 v3.1.10 (3.1.10)
)
(c:/texlive/2024/texmf-
dist/tex/generic/pgf/basiclayer/pgfcorepatterns.code.tex
```

```
File: pgfcorepatterns.code.tex 2023-01-15 v3.1.10 (3.1.10)
) (c:/texlive/2024/texmf-
dist/tex/generic/pgf/basiclayer/pgfcorerdf.code.tex
File: pgfcorerdf.code.tex 2023-01-15 v3.1.10 (3.1.10)
))) (c:/texlive/2024/texmf-
dist/tex/generic/pgf/modules/pgfmoduleshapes.code.te
x
File: pgfmoduleshapes.code.tex 2023-01-15 v3.1.10 (3.1.10)
\pgfnodeparttextbox=\box85
) (c:/texlive/2024/texmf-
dist/tex/generic/pgf/modules/pgfmoduleplot.code.tex
File: pgfmoduleplot.code.tex 2023-01-15 v3.1.10 (3.1.10)
)
(c:/texlive/2024/texmf-dist/tex/latex/pgf/compatibility/pgfcomp-version-
0-65.st
y
Package: pgfcomp-version-0-65 2023-01-15 v3.1.10 (3.1.10)
\pgf@nodesepstart=\dimen307
\pgf@nodesepend=\dimen308
)
(c:/texlive/2024/texmf-dist/tex/latex/pgf/compatibility/pgfcomp-version-
1-18.st
y
Package: pgfcomp-version-1-18 2023-01-15 v3.1.10 (3.1.10)
)) (c:/texlive/2024/texmf-dist/tex/latex/pgf/utilities/pgffor.sty
(c:/texlive/2
024/texmf-dist/tex/latex/pgf/utilities/pgfkeys.sty
(c:/texlive/2024/texmf-dist/
tex/generic/pgf/utilities/pgfkeys.code.tex)) (c:/texlive/2024/texmf-
dist/tex/la
tex/pgf/math/pgfmath.sty (c:/texlive/2024/texmf-
dist/tex/generic/pgf/math/pgfma
th.code.tex)) (c:/texlive/2024/texmf-
dist/tex/generic/pgf/utilities/pgffor.code
.tex
Package: pgffor 2023-01-15 v3.1.10 (3.1.10)
\pgffor@iter=\dimen309
\pgffor@skip=\dimen310
\pgffor@stack=\toks52
\pgffor@toks=\toks53
)) (c:/texlive/2024/texmf-
dist/tex/generic/pgf/frontendlayer/tikz/tikz.code.tex
Package: tikz 2023-01-15 v3.1.10 (3.1.10)

(c:/texlive/2024/texmf-
dist/tex/generic/pgf/libraries/pgflibraryplothandlers.co
de.tex
File: pgflibraryplothandlers.code.tex 2023-01-15 v3.1.10 (3.1.10)
\pgf@plot@mark@count=\count353
\pgfplotmarksize=\dimen311
)
\tikz@lastx=\dimen312
\tikz@lasty=\dimen313
\tikz@lastxsaved=\dimen314
```

```
\tikz@lastysaved=\dimen315
\tikz@lastmovetox=\dimen316
\tikz@lastmovetoy=\dimen317
\tikzleveldistance=\dimen318
\tikzsiblingdistance=\dimen319
\tikz@figbox=\box86
\tikz@figbox@bg=\box87
\tikz@tempbox=\box88
\tikz@tempbox@bg=\box89
\tikztreelevel=\count354
\tikznumberofchildren=\count355
\tikznumberofcurrentchild=\count356
\tikz@fig@count=\count357
(c:/texlive/2024/texmf-
dist/tex/generic/pgf/modules/pgfmodulematrix.code.tex
File: pgfmodulematrix.code.tex 2023-01-15 v3.1.10 (3.1.10)
\pgfmatrixcurrentrow=\count358
\pgfmatrixcurrentcolumn=\count359
\pgf@matrix@numberofcolumns=\count360
)
\tikz@expandcount=\count361

(c:/texlive/2024/texmf-
dist/tex/generic/pgf/frontendlayer/tikz/libraries/tikzli
brarytopaths.code.tex
File: tikzlibrarytopaths.code.tex 2023-01-15 v3.1.10 (3.1.10)
)))
(c:/texlive/2024/texmf-
dist/tex/generic/pgf/frontendlayer/tikz/libraries/tikzli
brarysvg.path.code.tex
File: tikzlibrarysvg.path.code.tex 2023-01-15 v3.1.10 (3.1.10)

(c:/texlive/2024/texmf-
dist/tex/generic/pgf/libraries/pgflibrarysvg.path.code.t
ex
File: pgflibrarysvg.path.code.tex 2023-01-15 v3.1.10 (3.1.10)
(c:/texlive/2024/texmf-
dist/tex/generic/pgf/modules/pgfmoduleparser.code.tex
File: pgfmoduleparser.code.tex 2023-01-15 v3.1.10 (3.1.10)
\pgfparserdef@arg@count=\count362
)
\pgf@lib@svg@last@x=\dimen320
\pgf@lib@svg@last@y=\dimen321
\pgf@lib@svg@last@c@x=\dimen322
\pgf@lib@svg@last@c@y=\dimen323
\pgf@lib@svg@count=\count363
\pgf@lib@svg@max@num=\count364
))
\@curXheight=\skip165
)
Package translations Info: No language package found. I am going to use
`englis
h' as default language. on input line 98.
```

```
LaTeX Font Info:    Trying to load font information for T1+Merriwthr-OsF
on inp
ut line 98.
(c:/texlive/2024/texmf-dist/tex/latex/merriweather/T1Merriwthr-OsF.fd
File: T1Merriwthr-OsF.fd 2020/08/30 (autoinst) Font definitions for
T1/Merriwth
r-OsF.
)
LaTeX Font Info:    Font shape `T1/Merriwthr-OsF/m/n' will be
(Font)              scaled to size 7.5pt on input line 98.
(./main.aux)
\openout1 = `main.aux'.

LaTeX Font Info:    Checking defaults for OML/cmm/m/it on input line 98.
LaTeX Font Info:    ... okay on input line 98.
LaTeX Font Info:    Checking defaults for OMS/cmsy/m/n on input line 98.
LaTeX Font Info:    ... okay on input line 98.
LaTeX Font Info:    Checking defaults for OT1/cmr/m/n on input line 98.
LaTeX Font Info:    ... okay on input line 98.
LaTeX Font Info:    Checking defaults for T1/cmr/m/n on input line 98.
LaTeX Font Info:    ... okay on input line 98.
LaTeX Font Info:    Checking defaults for TS1/cmr/m/n on input line 98.
LaTeX Font Info:    ... okay on input line 98.
LaTeX Font Info:    Checking defaults for OMX/cmex/m/n on input line 98.
LaTeX Font Info:    ... okay on input line 98.
LaTeX Font Info:    Checking defaults for U/cmr/m/n on input line 98.
LaTeX Font Info:    ... okay on input line 98.
LaTeX Font Info:    Checking defaults for PD1/pdf/m/n on input line 98.
LaTeX Font Info:    ... okay on input line 98.
LaTeX Font Info:    Checking defaults for PU/pdf/m/n on input line 98.
LaTeX Font Info:    ... okay on input line 98.
LaTeX Info: Redefining \microtypecontext on input line 98.
Package microtype Info: Applying patch `item' on input line 98.
Package microtype Info: Applying patch `toc' on input line 98.
Package microtype Info: Applying patch `eqnum' on input line 98.
Package microtype Info: Applying patch `footnote' on input line 98.
Package microtype Info: Applying patch `verbatim' on input line 98.
Package microtype Info: Generating PDF output.
Package microtype Info: Character protrusion enabled (level 2).
Package microtype Info: Using default protrusion set `alltext'.
Package microtype Info: Automatic font expansion enabled (level 2),
(microtype)             stretch: 20, shrink: 20, step: 1, non-selected.
Package microtype Info: Using default expansion set `alltext-nott'.
LaTeX Info: Redefining \showhyphens on input line 98.
Package microtype Info: No adjustment of tracking.
Package microtype Info: No adjustment of interword spacing.
Package microtype Info: No adjustment of character kerning.
Package microtype Info: Loading generic protrusion settings for font
family
(microtype)             `Merriwthr-OsF' (encoding: T1).
(microtype)             For optimal results, create family-specific
settings.
(microtype)             See the microtype manual for details.
LaTeX Font Info:    Redeclaring symbol font `operators' on input line 98.
```

```
LaTeX Font Info:    Encoding `OT1' has changed to `T1' for symbol font
(Font)              `operators' in the math version `normal' on input
line 98.
LaTeX Font Info:    Overwriting symbol font `operators' in version
`normal'
(Font)                  OT1/lmr/m/n --> T1/Merriwthr-OsF/m/up on input
line 98.

LaTeX Font Info:    Encoding `OT1' has changed to `T1' for symbol font
(Font)              `operators' in the math version `bold' on input line
98.
LaTeX Font Info:    Overwriting symbol font `operators' in version `bold'
(Font)                  OT1/lmr/bx/n --> T1/Merriwthr-OsF/m/up on input
line 98
.
LaTeX Font Info:    Overwriting symbol font `operators' in version `bold'
(Font)                  T1/Merriwthr-OsF/m/up --> T1/Merriwthr-OsF/b/up
on inpu
t line 98.
LaTeX Font Info:    Redeclaring math alphabet \mathbf on input line 98.
LaTeX Font Info:    Overwriting math alphabet `\mathbf' in version
`normal'
(Font)                  OT1/lmr/bx/n --> T1/Merriwthr-OsF/b/up on input
line 98
.
LaTeX Font Info:    Overwriting math alphabet `\mathbf' in version `bold'
(Font)                  OT1/lmr/bx/n --> T1/Merriwthr-OsF/b/up on input
line 98
.
LaTeX Font Info:    Redeclaring math alphabet \mathsf on input line 98.
LaTeX Font Info:    Overwriting math alphabet `\mathsf' in version
`normal'
(Font)                  OT1/lmss/m/n --> T1/MerriwthrSans-OsF/m/up on
input lin
e 98.
LaTeX Font Info:    Overwriting math alphabet `\mathsf' in version `bold'
(Font)                  OT1/lmss/bx/n --> T1/MerriwthrSans-OsF/m/up on
input li
ne 98.
LaTeX Font Info:    Redeclaring math alphabet \mathit on input line 98.
LaTeX Font Info:    Overwriting math alphabet `\mathit' in version
`normal'
(Font)                  OT1/lmr/m/it --> T1/Merriwthr-OsF/m/it on input
line 98
.
LaTeX Font Info:    Overwriting math alphabet `\mathit' in version `bold'
(Font)                  OT1/lmr/bx/it --> T1/Merriwthr-OsF/m/it on input
line 9
8.
LaTeX Font Info:    Redeclaring math alphabet \mathtt on input line 98.
LaTeX Font Info:    Overwriting math alphabet `\mathtt' in version
`normal'
(Font)                  OT1/lmtt/m/n --> T1/lmtt/m/up on input line 98.
LaTeX Font Info:    Overwriting math alphabet `\mathtt' in version `bold'
```

```
(Font)                  OT1/lmtt/m/n --> T1/lmtt/m/up on input line 98.
LaTeX Font Info:    Overwriting math alphabet `\mathsf' in version `bold'
(Font)                  T1/MerriwthrSans-OsF/m/up --> T1/MerriwthrSans-
OsF/b/up
 on input line 98.
LaTeX Font Info:    Overwriting math alphabet `\mathit' in version `bold'
(Font)                  T1/Merriwthr-OsF/m/it --> T1/Merriwthr-OsF/b/it
on inpu
t line 98.
\c@mv@tabular=\count365
\c@mv@boldtabular=\count366
(c:/texlive/2024/texmf-dist/tex/context/base/mkii/supp-pdf.mkii
[Loading MPS to PDF converter (version 2006.09.02).]
\scratchcounter=\count367
\scratchdimen=\dimen324
\scratchbox=\box90
\nofMPsegments=\count368
\nofMParguments=\count369
\everyMPshowfont=\toks54
\MPscratchCnt=\count370
\MPscratchDim=\dimen325
\MPnumerator=\count371
\makeMPintoPDFobject=\count372
\everyMPtoPDFconversion=\toks55
) (c:/texlive/2024/texmf-dist/tex/latex/epstopdf-pkg/epstopdf-base.sty
Package: epstopdf-base 2020-01-24 v2.11 Base part for package epstopdf
Package epstopdf-base Info: Redefining graphics rule for `.eps' on input
line 4
85.
(c:/texlive/2024/texmf-dist/tex/latex/latexconfig/epstopdf-sys.cfg
File: epstopdf-sys.cfg 2010/07/13 v1.3 Configuration of (r)epstopdf for
TeX Liv
e
))
*geometry* driver: auto-detecting
*geometry* detected driver: pdftex
*geometry* verbose mode - [ preamble ] result:
* driver: pdftex
* paper: a4paper
* layout: <same size as paper>
* layoutoffset:(h,v)=(0.0pt,0.0pt)
* modes: includefoot twoside
* h-part:(L,W,R)=(54.64pt, 488.22787pt, 54.64pt)
* v-part:(T,H,B)=(66.0pt, 745.04684pt, 34.0pt)
* \paperwidth=597.50787pt
* \paperheight=845.04684pt
* \textwidth=488.22787pt
* \textheight=715.04684pt
* \oddsidemargin=-17.62999pt
* \evensidemargin=-17.62999pt
* \topmargin=-47.76999pt
* \headheight=17.5pt
* \headsep=24.0pt
* \topskip=10.0pt
```

```
* \footskip=30.0pt
* \marginparwidth=48.0pt
* \marginparsep=10.0pt
* \columnsep=18.0pt
* \skip\footins=22.0pt plus 2.0pt
* \hoffset=0.0pt
* \voffset=0.0pt
* \mag=1000
* \@twocolumntrue
* \@twosidetrue
* \@mparswitchtrue
* \@reversemarginfalse
* (1in=72.27pt=25.4mm, 1cm=28.453pt)

Package caption Info: Begin \AtBeginDocument code.
Package caption Info: hyperref package is loaded.
Package caption Info: End \AtBeginDocument code.
Package hyperref Info: Link coloring OFF on input line 98.
(./main.out) (./main.out)
\@outlinefile=\write4
\openout4 = `main.out'.


(c:/texlive/2024/texmf-dist/tex/latex/translations/translations-basic-
dictionar
y-english.trsl
File: translations-basic-dictionary-english.trsl (english translation
file `tra
nslations-basic-dictionary')
)
Package translations Info: loading dictionary `translations-basic-
dictionary' f
or `english'. on input line 98.
\@gscitedetails=\box91
\@gscitedetailsheight=\skip166
\@gsheadbox=\box92
\@gsheadboxheight=\skip167
LaTeX Font Info:    Font shape `T1/Merriwthr-OsF/b/n' will be
(Font)              scaled to size 6.5pt on input line 98.
LaTeX Font Info:    Calculating math sizes for size <7.5> on input line
98.

LaTeX Font Warning: Font shape `T1/Merriwthr-OsF/m/up' undefined
(Font)              using `T1/Merriwthr-OsF/m/n' instead on input line
98.

LaTeX Font Info:    Font shape `T1/Merriwthr-OsF/m/up' will be
(Font)              scaled to size 6.24973pt on input line 98.
LaTeX Font Info:    Font shape `T1/Merriwthr-OsF/m/up' will be
(Font)              scaled to size 5.24997pt on input line 98.
LaTeX Font Info:    Trying to load font information for U+eur on input
line 98.

(c:/texlive/2024/texmf-dist/tex/latex/amsfonts/ueur.fd
```

File: ueur.fd 2013/01/14 v3.01 Euler Roman
) (c:/texlive/2024/texmf-dist/tex/latex/microtype/mt-eur.cfg
File: mt-eur.cfg 2006/07/31 v1.1 microtype config. file: AMS Euler Roman
(RS)
)
LaTeX Font Info: Trying to load font information for OMS+lmsy on input
line
98.
(c:/texlive/2024/texmf-dist/tex/latex/lm/omslmsy.fd
File: omslmsy.fd 2015/05/01 v1.6.1 Font defs for Latin Modern
)
LaTeX Font Info: Trying to load font information for OMX+lmex on input
line
98.
(c:/texlive/2024/texmf-dist/tex/latex/lm/omxlmex.fd
File: omxlmex.fd 2015/05/01 v1.6.1 Font defs for Latin Modern
)
LaTeX Font Info: External font `lmex10' loaded for size
(Font)   <7.5> on input line 98.
LaTeX Font Info: External font `lmex10' loaded for size
(Font)   <6.24973> on input line 98.
LaTeX Font Info: External font `lmex10' loaded for size
(Font)   <5.24997> on input line 98.
LaTeX Font Info: Trying to load font information for U+euf on input
line 98.

(c:/texlive/2024/texmf-dist/tex/latex/amsfonts/ueuf.fd
File: ueuf.fd 2013/01/14 v3.01 Euler Fraktur
) (c:/texlive/2024/texmf-dist/tex/latex/microtype/mt-euf.cfg
File: mt-euf.cfg 2006/07/03 v1.1 microtype config. file: AMS Euler
Fraktur (RS)

)
LaTeX Font Info: Trying to load font information for U+eus on input
line 98.

(c:/texlive/2024/texmf-dist/tex/latex/amsfonts/ueus.fd
File: ueus.fd 2013/01/14 v3.01 Euler Script
) (c:/texlive/2024/texmf-dist/tex/latex/microtype/mt-eus.cfg
File: mt-eus.cfg 2006/07/28 v1.2 microtype config. file: AMS Euler Script
(RS)
)
LaTeX Font Info: Trying to load font information for U+euex on input
line 98
.
(c:/texlive/2024/texmf-dist/tex/latex/amsfonts/ueuex.fd
File: ueuex.fd 2013/01/14 v3.01 Euler extra symbols
)
LaTeX Font Info: Font shape `T1/Merriwthr-OsF/m/n' will be
(Font)   scaled to size 6.24973pt on input line 98.
LaTeX Font Info: Font shape `T1/Merriwthr-OsF/m/n' will be
(Font)   scaled to size 5.24997pt on input line 98.
LaTeX Font Info: Font shape `T1/Merriwthr-OsF/m/it' will be
(Font)   scaled to size 7.5pt on input line 98.

```
LaTeX Font Info:    Font shape `T1/Merriwthr-OsF/m/it' will be
(Font)              scaled to size 6.24973pt on input line 98.
LaTeX Font Info:    Font shape `T1/Merriwthr-OsF/m/it' will be
(Font)              scaled to size 5.24997pt on input line 98.
LaTeX Font Info:    Font shape `T1/Merriwthr-OsF/m/n' will be
(Font)              scaled to size 8.0pt on input line 98.
LaTeX Font Info:    Font shape `T1/Merriwthr-OsF/m/it' will be
(Font)              scaled to size 8.0pt on input line 98.
LaTeX Font Info:    Font shape `T1/Merriwthr-OsF/b/it' will be
(Font)              scaled to size 8.0pt on input line 98.
TextBlockOrigin set to 4pc+6.64pt x 4pc+6pt

Overfull \hbox (14.11896pt too wide) in paragraph at lines 114--114
\T1/Merriwthr-OsF/m/n/7.5 (-20) Figures/gigascience-
 []


Overfull \hbox (54.64pt too wide) in paragraph at lines 114--114
[][]
 []

LaTeX Font Info:    Font shape `T1/Merriwthr-OsF/m/n' will be
(Font)              scaled to size 14.0pt on input line 114.
LaTeX Font Info:    Font shape `T1/Merriwthr-OsF/m/n' will be
(Font)              scaled to size 8.99997pt on input line 114.
LaTeX Font Info:    Calculating math sizes for size <14> on input line
114.
LaTeX Font Info:    Font shape `T1/Merriwthr-OsF/m/up' will be
(Font)              scaled to size 14.0pt on input line 114.
LaTeX Font Info:    Font shape `T1/Merriwthr-OsF/m/up' will be
(Font)              scaled to size 11.66617pt on input line 114.
LaTeX Font Info:    Font shape `T1/Merriwthr-OsF/m/up' will be
(Font)              scaled to size 9.79996pt on input line 114.
LaTeX Font Info:    External font `lmex10' loaded for size
(Font)              <14> on input line 114.
LaTeX Font Info:    External font `lmex10' loaded for size
(Font)              <11.66617> on input line 114.
LaTeX Font Info:    External font `lmex10' loaded for size
(Font)              <9.79996> on input line 114.
LaTeX Font Info:    Font shape `T1/Merriwthr-OsF/m/n' will be
(Font)              scaled to size 11.66617pt on input line 114.
LaTeX Font Info:    Font shape `T1/Merriwthr-OsF/m/n' will be
(Font)              scaled to size 9.79996pt on input line 114.
LaTeX Font Info:    Font shape `T1/Merriwthr-OsF/m/it' will be
(Font)              scaled to size 14.0pt on input line 114.
LaTeX Font Info:    Font shape `T1/Merriwthr-OsF/m/it' will be
(Font)              scaled to size 11.66617pt on input line 114.
LaTeX Font Info:    Font shape `T1/Merriwthr-OsF/m/it' will be
(Font)              scaled to size 9.79996pt on input line 114.
LaTeX Font Info:    Font shape `T1/Merriwthr-OsF/b/n' will be
(Font)              scaled to size 18.0pt on input line 114.
LaTeX Font Info:    Font shape `T1/Merriwthr-OsF/b/it' will be
(Font)              scaled to size 18.0pt on input line 114.
LaTeX Font Info:    Font shape `T1/Merriwthr-OsF/m/n' will be
```

```
(Font)              scaled to size 13.0pt on input line 114.
LaTeX Font Info:    Calculating math sizes for size <13> on input line
114.
LaTeX Font Info:    Font shape `T1/Merriwthr-OsF/m/up' will be
(Font)              scaled to size 13.0pt on input line 114.
LaTeX Font Info:    Font shape `T1/Merriwthr-OsF/m/up' will be
(Font)              scaled to size 10.83287pt on input line 114.
LaTeX Font Info:    Font shape `T1/Merriwthr-OsF/m/up' will be
(Font)              scaled to size 9.09996pt on input line 114.
LaTeX Font Info:    External font `lmex10' loaded for size
(Font)              <13> on input line 114.
LaTeX Font Info:    External font `lmex10' loaded for size
(Font)              <10.83287> on input line 114.
LaTeX Font Info:    External font `lmex10' loaded for size
(Font)              <9.09996> on input line 114.
LaTeX Font Info:    Font shape `T1/Merriwthr-OsF/m/n' will be
(Font)              scaled to size 10.83287pt on input line 114.
LaTeX Font Info:    Font shape `T1/Merriwthr-OsF/m/n' will be
(Font)              scaled to size 9.09996pt on input line 114.
LaTeX Font Info:    Font shape `T1/Merriwthr-OsF/m/it' will be
(Font)              scaled to size 13.0pt on input line 114.
LaTeX Font Info:    Font shape `T1/Merriwthr-OsF/m/it' will be
(Font)              scaled to size 10.83287pt on input line 114.
LaTeX Font Info:    Font shape `T1/Merriwthr-OsF/m/it' will be
(Font)              scaled to size 9.09996pt on input line 114.
LaTeX Font Info:    Trying to load font information for TS1+Merriwthr-OsF
on in
put line 114.
(c:/texlive/2024/texmf-dist/tex/latex/merriweather/TS1Merriwthr-OsF.fd
File: TS1Merriwthr-OsF.fd 2020/08/30 (autoinst) Font definitions for
TS1/Merriw
thr-OsF.
)
LaTeX Font Info:    Font shape `TS1/Merriwthr-OsF/m/n' will be
(Font)              scaled to size 10.83287pt on input line 114.
Package microtype Info: Loading generic protrusion settings for font
family
(microtype)                `Merriwthr-OsF' (encoding: TS1).
(microtype)                For optimal results, create family-specific
settings.
(microtype)                See the microtype manual for details.
LaTeX Font Info:    Font shape `T1/Merriwthr-OsF/m/n' will be
(Font)              scaled to size 9.0pt on input line 114.
LaTeX Font Info:    Font shape `T1/Merriwthr-OsF/m/up' will be
(Font)              scaled to size 9.0pt on input line 114.
LaTeX Font Info:    Font shape `T1/Merriwthr-OsF/m/up' will be
(Font)              scaled to size 7.0pt on input line 114.
LaTeX Font Info:    Font shape `T1/Merriwthr-OsF/m/up' will be
(Font)              scaled to size 5.0pt on input line 114.
LaTeX Font Info:    External font `lmex10' loaded for size
(Font)              <9> on input line 114.
LaTeX Font Info:    External font `lmex10' loaded for size
(Font)              <7> on input line 114.
LaTeX Font Info:    External font `lmex10' loaded for size
```

```
(Font)              <5> on input line 114.
LaTeX Font Info:    Font shape `T1/Merriwthr-OsF/m/n' will be
(Font)              scaled to size 7.0pt on input line 114.
LaTeX Font Info:    Font shape `T1/Merriwthr-OsF/m/n' will be
(Font)              scaled to size 5.0pt on input line 114.
LaTeX Font Info:    Font shape `T1/Merriwthr-OsF/m/it' will be
(Font)              scaled to size 9.0pt on input line 114.
LaTeX Font Info:    Font shape `T1/Merriwthr-OsF/m/it' will be
(Font)              scaled to size 7.0pt on input line 114.
LaTeX Font Info:    Font shape `T1/Merriwthr-OsF/m/it' will be
(Font)              scaled to size 5.0pt on input line 114.
LaTeX Font Info:    Font shape `T1/Merriwthr-OsF/m/n' will be
(Font)              scaled to size 6.5pt on input line 114.
LaTeX Font Info:    Calculating math sizes for size <6.5> on input line
114.
LaTeX Font Info:    Font shape `T1/Merriwthr-OsF/m/up' will be
(Font)              scaled to size 6.5pt on input line 114.
LaTeX Font Info:    Font shape `T1/Merriwthr-OsF/m/up' will be
(Font)              scaled to size 5.41643pt on input line 114.
LaTeX Font Info:    Font shape `T1/Merriwthr-OsF/m/up' will be
(Font)              scaled to size 4.54997pt on input line 114.
LaTeX Font Info:    External font `lmex10' loaded for size
(Font)              <6.5> on input line 114.
LaTeX Font Info:    External font `lmex10' loaded for size
(Font)              <5.41643> on input line 114.
LaTeX Font Info:    External font `lmex10' loaded for size
(Font)              <4.54997> on input line 114.
LaTeX Font Info:    Font shape `T1/Merriwthr-OsF/m/n' will be
(Font)              scaled to size 5.41643pt on input line 114.
LaTeX Font Info:    Font shape `T1/Merriwthr-OsF/m/n' will be
(Font)              scaled to size 4.54997pt on input line 114.
LaTeX Font Info:    Font shape `T1/Merriwthr-OsF/m/it' will be
(Font)              scaled to size 6.5pt on input line 114.
LaTeX Font Info:    Font shape `T1/Merriwthr-OsF/m/it' will be
(Font)              scaled to size 5.41643pt on input line 114.
LaTeX Font Info:    Font shape `T1/Merriwthr-OsF/m/it' will be
(Font)              scaled to size 4.54997pt on input line 114.
LaTeX Font Info:    Font shape `TS1/Merriwthr-OsF/m/n' will be
(Font)              scaled to size 5.41643pt on input line 114.

Overfull \hbox (54.64pt too wide) in paragraph at lines 114--114
[][][]
 []

LaTeX Font Info:    Font shape `T1/Merriwthr-OsF/b/n' will be
(Font)              scaled to size 10.0pt on input line 114.
LaTeX Font Info:    Font shape `T1/Merriwthr-OsF/b/n' will be
(Font)              scaled to size 8.0pt on input line 114.

Overfull \hbox (54.64pt too wide) in paragraph at lines 114--114
[][][]
 []
```

```
Underfull \vbox (badness 10000) has occurred while \output is active []



LaTeX Font Info:    Font shape `T1/Merriwthr-OsF/b/n' will be
(Font)              scaled to size 7.5pt on input line 119.

Package natbib Warning: Citation `Delsuc2006' on page 1 undefined on
input line
 119.


Package natbib Warning: Citation `Alie2020' on page 1 undefined on input
line 1
19.


Package natbib Warning: Citation `Stolfi2015d' on page 1 undefined on
input lin
e 119.


Package natbib Warning: Citation `Hiebert2021' on page 1 undefined on
input lin
e 119.


Package natbib Warning: Citation `Manni2014c' on page 1 undefined on
input line
 120.


Package natbib Warning: Citation `Sabbadin1975' on page 1 undefined on
input li
ne 120.


Package natbib Warning: Citation `Nourizadeh2021' on page 1 undefined on
input
line 120.


Package natbib Warning: Citation `Ricci2022' on page 1 undefined on input
line
120.


LaTeX Warning: File `Figures/ImageBS1.jpg' not found on input line 123.


! Package pdftex.def Error: File `Figures/ImageBS1.jpg' not found: using
draft
setting.
```

```
See the pdftex.def package documentation for explanation.
Type  H <return>  for immediate help.
 ...

l.123 ...[width=1\linewidth]{Figures/ImageBS1.jpg}

Try typing  <return>  to proceed.
If that doesn't work, type  X <return>  to quit.

LaTeX Font Info:    Trying to load font information for T1+lmtt on input
line 1
23.
(c:/texlive/2024/texmf-dist/tex/latex/lm/t1lmtt.fd
File: t1lmtt.fd 2015/05/01 v1.6.1 Font defs for Latin Modern
)
Package microtype Info: Loading generic protrusion settings for font
family
(microtype)             `lmtt' (encoding: T1).
(microtype)             For optimal results, create family-specific
settings.
(microtype)             See the microtype manual for details.
LaTeX Font Info:    Font shape `T1/Merriwthr-OsF/m/n' will be
(Font)              scaled to size 6.0pt on input line 124.
LaTeX Font Info:    Font shape `T1/Merriwthr-OsF/b/n' will be
(Font)              scaled to size 6.0pt on input line 124.
LaTeX Font Info:    Font shape `T1/Merriwthr-OsF/m/it' will be
(Font)              scaled to size 6.0pt on input line 124.

Package natbib Warning: Citation `Laird2005a' on page 1 undefined on
input line
 127.


Package natbib Warning: Citation `Laird2005b' on page 1 undefined on
input line
 127.


Package natbib Warning: Citation `Brown2009d' on page 1 undefined on
input line
 127.


Package natbib Warning: Citation `Pancer1995' on page 1 undefined on
input line
 127.


Package natbib Warning: Citation `Stoner1996' on page 1 undefined on
input line
 127.
```

Package natbib Warning: Citation `Laird2005b' on page 1 undefined on input line
 127.

Package natbib Warning: Citation `Manni2019' on page 1 undefined on input line
128.

Package natbib Warning: Citation `Kassmer2016a' on page 1 undefined on input li
ne 128.

Package natbib Warning: Citation `Taketa2015' on page 1 undefined on input line
 128.

Package natbib Warning: Citation `Nydam2020' on page 1 undefined on input line
128.

Package natbib Warning: Citation `Epelbaum2009' on page 1 undefined on input li
ne 128.

Package natbib Warning: Citation `Gasparini2015' on page 1 undefined on input l
ine 128.

Package natbib Warning: Citation `Wawrzyniak2021a' on page 1 undefined on input
 line 128.

Package natbib Warning: Citation `Laird2005a' on page 1 undefined on input line
 128.

Package natbib Warning: Citation `Langenbacher2015a' on page 1 undefined on inp
ut line 128.

Package natbib Warning: Citation `Ricci2022' on page 1 undefined on input line
128.

```
Package natbib Warning: Citation `Manni2007' on page 1 undefined on input
line
128.


Package natbib Warning: Citation `Manni2014c' on page 1 undefined on
input line
 128.


Package natbib Warning: Citation `Rodriguez2014a' on page 1 undefined on
input
line 128.


Package natbib Warning: Citation `Campagna2016' on page 1 undefined on
input li
ne 128.


Package natbib Warning: Citation `Ricci2016' on page 1 undefined on input
line
128.


Package natbib Warning: Citation `Rosental2018' on page 1 undefined on
input li
ne 128.


Package natbib Warning: Citation `Kowarsky2021' on page 1 undefined on
input li
ne 128.


Package natbib Warning: Citation `Ricci2022' on page 1 undefined on input
line
128.


Package natbib Warning: Citation `Voskoboynik2013c' on page 1 undefined
on inpu
t line 128.


Package natbib Warning: Citation `Lawniczak2022' on page 1 undefined on
input l
ine 128.


Underfull \vbox (badness 10000) has occurred while \output is active []
```

```
LaTeX Font Info:    Font shape `T1/Merriwthr-OsF/m/n' will be
(Font)              scaled to size 7.8pt on input line 129.
LaTeX Font Info:    Font shape `T1/Merriwthr-OsF/b/n' will be
(Font)              scaled to size 7.8pt on input line 129.
[1{c:/texlive/2024/texmf-
var/fonts/map/pdftex/updmap/pdftex.map}{c:/texlive/202
4/texmf-
dist/fonts/enc/dvips/merriweather/merriwthr_posqbl.enc}{c:/texlive/2024
/texmf-dist/fonts/enc/dvips/merriweather/merriwthr_owzwzj.enc}


]
Underfull \vbox (badness 10000) has occurred while \output is active []



LaTeX Font Info:    Font shape `T1/Merriwthr-OsF/b/n' will be
(Font)              scaled to size 8.5pt on input line 133.
LaTeX Font Info:    Font shape `T1/Merriwthr-OsF/m/up' will be
(Font)              scaled to size 7.5pt on input line 134.
LaTeX Font Info:    Font shape `T1/Merriwthr-OsF/m/n' will be
(Font)              scaled to size 5.00003pt on input line 137.
LaTeX Font Info:    Font shape `T1/Merriwthr-OsF/b/n' will be
(Font)              scaled to size 7.0pt on input line 155.

Package natbib Warning: Citation `DeTomaso1998a' on page 2 undefined on
input l
ine 160.


Package natbib Warning: Citation `Voskoboynik2013c' on page 2 undefined
on inpu
t line 160.


Package natbib Warning: Citation `Cheng2021' on page 2 undefined on input
line
161.


Package natbib Warning: Citation `Bojko2022' on page 2 undefined on input
line
162.


Package natbib Warning: Citation `Li2023' on page 2 undefined on input
line 163
.


Package natbib Warning: Citation `Zhou2023' on page 2 undefined on input
line 1
63.
```

```
Package natbib Warning: Citation `Colombera1969' on page 2 undefined on
input l
ine 163.


LaTeX Warning: File `Figures/heatmap/asm_primary_hic_heatmap.pdf' not
found on
input line 170.


! Package pdftex.def Error: File
`Figures/heatmap/asm_primary_hic_heatmap.pdf'
not found: using draft setting.

See the pdftex.def package documentation for explanation.
Type  H <return>  for immediate help.
 ...

l.170 ...ures/heatmap/asm_primary_hic_heatmap.pdf}

Try typing  <return>  to proceed.
If that doesn't work, type  X <return>  to quit.


Package natbib Warning: Citation `Zeng2024' on page 2 undefined on input
line 1
71.



LaTeX Font Info:    Font shape `T1/Merriwthr-OsF/m/it' will be
(Font)              scaled to size 7.8pt on input line 173.
[2{c:/texlive/2024/texmf-dist/fonts/enc/dvips/lm/lm-ec.enc}]

LaTeX Warning: File `Figures/circos/bs_circos_plot.pdf' not found on
input line
 178.


! Package pdftex.def Error: File `Figures/circos/bs_circos_plot.pdf' not
found:
 using draft setting.

See the pdftex.def package documentation for explanation.
Type  H <return>  for immediate help.
 ...

l.178 ...e=0.4]{Figures/circos/bs_circos_plot.pdf}

Try typing  <return>  to proceed.
If that doesn't work, type  X <return>  to quit.
```

```
Package natbib Warning: Citation `Bandi2022' on page 3 undefined on input
line
180.


Package natbib Warning: Citation `Wang2012' on page 3 undefined on input
line 1
80.

LaTeX Font Info:    Font shape `T1/Merriwthr-OsF/m/n' will be
(Font)              scaled to size 6.25008pt on input line 187.

Underfull \hbox (badness 7722) in paragraph at lines 194--194
[]|\T1/Merriwthr-OsF/m/n/7 (+20) No. of an-no-tated
 []


Package natbib Warning: Citation `Simao2015' on page 3 undefined on input
line
204.


Package natbib Warning: Citation `Guiglielmoni2021' on page 3 undefined
on inpu
t line 204.


Package natbib Warning: Citation `Wang2012' on page 3 undefined on input
line 2
04.


Package natbib Warning: Citation `Simion2021c' on page 3 undefined on
input lin
e 204.


LaTeX Warning: File `Figures/pipeline/assembly_pipeline_haploid.pdf' not
found
on input line 229.


! Package pdftex.def Error: File
`Figures/pipeline/assembly_pipeline_haploid.pd
f' not found: using draft setting.

See the pdftex.def package documentation for explanation.
Type  H <return>  for immediate help.
 ...

l.229 ...s/pipeline/assembly_pipeline_haploid.pdf}

Try typing  <return>  to proceed.
```

```
If that doesn't work, type  X <return>  to quit.


LaTeX Warning: File `Figures/busco/busco_assembly.png' not found on input
line
237.


! Package pdftex.def Error: File `Figures/busco/busco_assembly.png' not
found:
using draft setting.

See the pdftex.def package documentation for explanation.
Type  H <return>  for immediate help.
 ...

l.237 ...le=0.1]{Figures/busco/busco_assembly.png}

Try typing  <return>  to proceed.
If that doesn't work, type  X <return>  to quit.


Package natbib Warning: Citation `Voskoboynik2013c' on page 3 undefined
on inpu
t line 238.


Package natbib Warning: Citation `Lopez-Legentil2006' on page 3 undefined
on in
put line 244.


Package natbib Warning: Citation `Bock2012' on page 3 undefined on input
line 2
44.


Package natbib Warning: Citation `Salonna2021' on page 3 undefined on
input lin
e 244.


Package natbib Warning: Citation `Salonna2021' on page 3 undefined on
input lin
e 244.


Package natbib Warning: Citation `Voskoboynik2013c' on page 3 undefined
on inpu
t line 244.


Package natbib Warning: Citation `Salonna2021' on page 3 undefined on
input lin
```

```
e 244.

Package natbib Warning: Citation `Voskoboynik2013c' on page 3 undefined
on inpu
t line 248.

Package natbib Warning: Citation `Blanchoud2018' on page 3 undefined on
input l
ine 250.

Package natbib Warning: Citation `Satou2019' on page 3 undefined on input
line
251.

Package natbib Warning: Citation `Bliznina2021' on page 3 undefined on
input li
ne 251.

Package natbib Warning: Citation `Castellano2023' on page 3 undefined on
input
line 251.

Underfull \hbox (badness 10000) in paragraph at lines 248--252
\T1/Merriwthr-OsF/m/up/7.5 (+20) Using a \T1/Merriwthr-OsF/m/it/7.5 (+20)
de no
vo \T1/Merriwthr-OsF/m/up/7.5 (+20) re-peat li-brary cre-ated by Re-peat-
Mod-el
er
 []

Underfull \hbox (badness 1515) in paragraph at lines 248--252
\T1/Merriwthr-OsF/m/up/7.5 (+20) that around 63% of the novel
\T1/Merriwthr-OsF
/m/it/7.5 (+20) B. schlosseri \T1/Merriwthr-OsF/m/up/7.5 (+20) col-lapsed
genom
e
 []

Package natbib Warning: Citation `Gabriel2024' on page 3 undefined on
input lin
e 253.
```

```
Package natbib Warning: Citation `Haas2003' on page 3 undefined on input line 2
53.


Package natbib Warning: Citation `Haas2008' on page 3 undefined on input line 2
53.


Package natbib Warning: Citation `Voskoboynik2013c' on page 3 undefined on inpu
t line 254.


Underfull \hbox (badness 1874) in paragraph at lines 253--257
[]\T1/Merriwthr-OsF/m/it/7.5 (+20) Ab ini-tio \T1/Merriwthr-OsF/m/up/7.5 (+20)
genome an-no-ta-tion us-ing the BRAKER3 pipeline
 []


Underfull \hbox (badness 2828) in paragraph at lines 253--257
\T1/Merriwthr-OsF/m/up/7.5 (+20) [\T1/Merriwthr-OsF/b/n/7.5 (+20) ? \T1/Merriwt
hr-OsF/m/up/7.5 (+20) ] (RRID:SCR_018964) ini-tially pre-dicted 16,966 cod-ing
 []


Underfull \vbox (badness 2057) has occurred while \output is active []


[3]

Package natbib Warning: Citation `Cantalapiedra2021' on page 4 undefined on inp
ut line 258.


Package natbib Warning: Citation `Blum2021' on page 4 undefined on input line 2
58.


Package natbib Warning: Citation `Jones2014a' on page 4 undefined on input line
 258.


LaTeX Warning: File `Figures/annotation/PIE_KOALA.png' not found on input line
301.
```

```
! Package pdftex.def Error: File `Figures/annotation/PIE_KOALA.png' not
found:
using draft setting.

See the pdftex.def package documentation for explanation.
Type  H <return>  for immediate help.
 ...

l.301 ...nwidth]{Figures/annotation/PIE_KOALA.png}

Try typing  <return>  to proceed.
If that doesn't work, type  X <return>  to quit.


Package natbib Warning: Citation `Kanehisa2016' on page 4 undefined on
input li
ne 303.


Package natbib Warning: Citation `Colombera1969' on page 4 undefined on
input l
ine 308.




Package natbib Warning: Citation `Wei2020' on page 4 undefined on input
line 31
4.


Package natbib Warning: Citation `Satou2019' on page 4 undefined on input
line
314.


Package natbib Warning: Citation `Bliznina2021' on page 4 undefined on
input li
ne 314.


Package natbib Warning: Citation `Delsuc2018a' on page 4 undefined on
input lin
e 314.


Package natbib Warning: Citation `Simakov2020' on page 4 undefined on
input lin
e 314.
```

Package natbib Warning: Citation `Schultz2023' on page 4 undefined on input lin
e 314.

Package natbib Warning: Citation `Simakov2022' on page 4 undefined on input lin
e 314.

Package natbib Warning: Citation `Lewin2025a' on page 4 undefined on input line
 314.

Package natbib Warning: Citation `Simakov2022' on page 4 undefined on input lin
e 314.

Package natbib Warning: Citation `Delsuc2018a' on page 4 undefined on input lin
e 316.

Package natbib Warning: Citation `Plessy2024' on page 4 undefined on input line
 316.

Package natbib Warning: Citation `Simakov2022' on page 4 undefined on input lin
e 316.

Package natbib Warning: Citation `Simakov2022' on page 4 undefined on input lin
e 316.

Package natbib Warning: Citation `Lewin2025a' on page 4 undefined on input line
 316.

Package natbib Warning: Citation `Lewin2025a' on page 4 undefined on input line
 316.

Package natbib Warning: Citation `Vargas-Chavez2025' on page 4 undefined on inp
ut line 316.

```
Package natbib Warning: Citation `Lewin2024' on page 4 undefined on input
line
316.


Package natbib Warning: Citation `Schultz2024' on page 4 undefined on
input lin
e 316.


Package natbib Warning: Citation `Berna2014' on page 4 undefined on input
line
316.


Underfull \vbox (badness 10000) has occurred while \output is active []



[4]

LaTeX Warning: File `Figures//Synteny/odp_CLG_ribbon_crop_tree.pdf' not
found o
n input line 321.


! Package pdftex.def Error: File
`Figures//Synteny/odp_CLG_ribbon_crop_tree.pdf
' not found: using draft setting.

See the pdftex.def package documentation for explanation.
Type  H <return>  for immediate help.
 ...

l.321 ...es//Synteny/odp_CLG_ribbon_crop_tree.pdf}

Try typing  <return>  to proceed.
If that doesn't work, type  X <return>  to quit.


Package natbib Warning: Citation `Simakov2020' on page 5 undefined on
input lin
e 322.


Package natbib Warning: Citation `Monteiro2006' on page 5 undefined on
input li
ne 327.


Package natbib Warning: Citation `DeBiasse2020' on page 5 undefined on
input li
ne 328.
```

Package natbib Warning: Citation `Sekigami2017' on page 5 undefined on input li
ne 328.

Package natbib Warning: Citation `DeBiasse2020' on page 5 undefined on input li
ne 328.

Package natbib Warning: Citation `Gaunt2022' on page 5 undefined on input line
329.

Package natbib Warning: Citation `DeBiasse2020' on page 5 undefined on input li
ne 329.

Package natbib Warning: Citation `Voskoboynik2013c' on page 5 undefined on inpu
t line 329.

Package natbib Warning: Citation `Blanchoud2018' on page 5 undefined on input l
ine 329.

Package natbib Warning: Citation `Sekigami2017' on page 5 undefined on input li
ne 329.

Package natbib Warning: Citation `Satou2019' on page 5 undefined on input line
329.

Package natbib Warning: Citation `Simakov2020' on page 5 undefined on input lin
e 329.

Package natbib Warning: Citation `Alie2020' on page 5 undefined on input line 3
29.

Package natbib Warning: Citation `Delsuc2018a' on page 5 undefined on input lin

```
e 329.


Underfull \vbox (badness 3503) has occurred while \output is active []




LaTeX Warning: File `Figures/Hox/hox_20072025_ST2.drawio.pdf' not found
on inpu
t line 336.


! Package pdftex.def Error: File
`Figures/Hox/hox_20072025_ST2.drawio.pdf' not
found: using draft setting.

See the pdftex.def package documentation for explanation.
Type  H <return>  for immediate help.
 ...

l.336 ...{Figures/Hox/hox_20072025_ST2.drawio.pdf}

Try typing  <return>  to proceed.
If that doesn't work, type  X <return>  to quit.


Package natbib Warning: Citation `Caputi2024' on page 5 undefined on
input line
 349.


Package natbib Warning: Citation `Blanchoud2018' on page 5 undefined on
input l
ine 349.


Package natbib Warning: Citation `Berna2014' on page 5 undefined on input
line
349.


Package natbib Warning: Citation `Sanges2013' on page 5 undefined on
input line
 349.


Package natbib Warning: Citation `Langenbacher2015a' on page 5 undefined
on inp
ut line 365.



[5]
```

```
Package natbib Warning: Citation `Sim2022' on page 6 undefined on input
line 36
9.


Package natbib Warning: Citation `Bolger2014a' on page 6 undefined on
input lin
e 369.



Package natbib Warning: Citation `M.Tawfeeq2025' on page 6 undefined on
input l
ine 372.


Package natbib Warning: Citation `Wang2023' on page 6 undefined on input
line 3
72.


Package natbib Warning: Citation `Vizueta2025' on page 6 undefined on
input lin
e 372.


Package natbib Warning: Citation `Schneider2012' on page 6 undefined on
input l
ine 374.


Package natbib Warning: Citation `Kokot2017' on page 6 undefined on input
line
376.


Package natbib Warning: Citation `Ranallo-Benavidez2020' on page 6
undefined on
 input line 376.


Underfull \vbox (badness 10000) has occurred while \output is active []



[6]
Underfull \vbox (badness 10000) has occurred while \output is active []
```

Package natbib Warning: Citation `Cheng2021' on page 7 undefined on input line
383.

Package natbib Warning: Citation `Huang2017' on page 7 undefined on input line
383.

Package natbib Warning: Citation `Challis2020a' on page 7 undefined on input li
ne 383.

Package natbib Warning: Citation `Camacho2009' on page 7 undefined on input lin
e 383.

Package natbib Warning: Citation `Buchfink2021' on page 7 undefined on input li
ne 383.

Package natbib Warning: Citation `Li2021' on page 7 undefined on input line 383
.

Package natbib Warning: Citation `Voskoboynik2013c' on page 7 undefined on inpu
t line 383.

Package natbib Warning: Citation `Li2023' on page 7 undefined on input line 384
.

Package natbib Warning: Citation `Ghurye2019' on page 7 undefined on input line
 384.

Package natbib Warning: Citation `Zhou2023' on page 7 undefined on input line 3
84.

Package natbib Warning: Citation `Shen2016' on page 7 undefined on input line 3
85.

Package natbib Warning: Citation `Mapleson2017' on page 7 undefined on input li
ne 385.

Package natbib Warning: Citation `Manni2021' on page 7 undefined on input line
385.

Package natbib Warning: Citation `Guan2020' on page 7 undefined on input line 3
89.

Package natbib Warning: Citation `Flynn2020' on page 7 undefined on input line
392.

Package natbib Warning: Citation `Dobin2013' on page 7 undefined on input line
392.

Package natbib Warning: Citation `Kuznetsov2023' on page 7 undefined on input l
ine 392.

Package natbib Warning: Citation `Lomsadze2005' on page 7 undefined on input li
ne 392.

Package natbib Warning: Citation `Stanke2006' on page 7 undefined on input line
 392.

Package natbib Warning: Citation `Lomsadze2014' on page 7 undefined on input li
ne 392.

Package natbib Warning: Citation `Gotoh2008' on page 7 undefined on input line
392.

Package natbib Warning: Citation `Iwata2012' on page 7 undefined on input line
392.

Package natbib Warning: Citation `Buchfink2015' on page 7 undefined on input li
ne 392.

Package natbib Warning: Citation `Bruna2020' on page 7 undefined on input line
392.

Package natbib Warning: Citation `Pertea2020' on page 7 undefined on input line
 392.

Package natbib Warning: Citation `Kovaka2019' on page 7 undefined on input line
 392.

Package natbib Warning: Citation `Stanke2008' on page 7 undefined on input line
 392.

Package natbib Warning: Citation `Hoff2019' on page 7 undefined on input line 3
92.

Package natbib Warning: Citation `Hoff2016' on page 7 undefined on input line 3
92.

Package natbib Warning: Citation `Bruna2021' on page 7 undefined on input line
392.

Package natbib Warning: Citation `Gabriel2024' on page 7 undefined on input lin
e 392.

Package natbib Warning: Citation `Haas2003' on page 7 undefined on input line 3
92.

Package natbib Warning: Citation `Haas2008' on page 7 undefined on input line 3

92.

Package natbib Warning: Citation `Rodriguez2014a' on page 7 undefined on input
line 392.

Package natbib Warning: Citation `Dobin2013' on page 7 undefined on input line
392.

Package natbib Warning: Citation `Pertea2015' on page 7 undefined on input line
392.

Package natbib Warning: Citation `Rodriguez2014a' on page 7 undefined on input
line 392.

Package natbib Warning: Citation `Ricci2016' on page 7 undefined on input line
392.

Package natbib Warning: Citation `Ricci2022' on page 7 undefined on input line
392.

Package natbib Warning: Citation `Haas2013' on page 7 undefined on input line 3
92.

Package natbib Warning: Citation `Cantalapiedra2021' on page 7 undefined on inp
ut line 393.

Package natbib Warning: Citation `Huerta-Cepas2019' on page 7 undefined on inpu
t line 393.

Package natbib Warning: Citation `Blum2021' on page 7 undefined on input line 3
93.

```
Package natbib Warning: Citation `Jones2014a' on page 7 undefined on
input line
 393.



[7]

Package natbib Warning: Citation `Dierckxsens2017' on page 8 undefined on
input
 line 399.


Package natbib Warning: Citation `Dardaillon2020' on page 8 undefined on
input
line 404.


Package natbib Warning: Citation `Bliznina2021' on page 8 undefined on
input li
ne 405.


Package natbib Warning: Citation `Castellano2023' on page 8 undefined on
input
line 405.


Package natbib Warning: Citation `Wei2020' on page 8 undefined on input
line 40
5.


Package natbib Warning: Citation `Schultz2023' on page 8 undefined on
input lin
e 405.


Underfull \hbox (badness 1609) in paragraph at lines 404--407
\T1/Merriwthr-OsF/m/up/7.5 (+20) The genome as-sem-blies and an-no-ta-
tions for
 the com-par-i-son
 []


Package natbib Warning: Citation `Salonna2021' on page 8 undefined on
input lin
e 410.


Package natbib Warning: Citation `Edgar2004' on page 8 undefined on input
line
410.
```

Package natbib Warning: Citation `Tamura2011' on page 8 undefined on input line
 410.

Package natbib Warning: Citation `Sekigami2017' on page 8 undefined on input li
ne 411.

Package natbib Warning: Citation `Edgar2004' on page 8 undefined on input line
411.

Package natbib Warning: Citation `Larsson2014' on page 8 undefined on input lin
e 411.

Package natbib Warning: Citation `Minh2020' on page 8 undefined on input line 4
11.

Package natbib Warning: Citation `Jones1992' on page 8 undefined on input line
411.

Package natbib Warning: Citation `Yang1995' on page 8 undefined on input line 4
11.

Package natbib Warning: Citation `Kalyaanamoorthy2017' on page 8 undefined on i
nput line 411.

Package natbib Warning: Citation `Schwarz1978' on page 8 undefined on input lin
e 411.

Package natbib Warning: Citation `Hoang2018' on page 8 undefined on input line
411.

Package natbib Warning: Citation `GigaDB' on page 8 undefined on input line 443
.

```
Package natbib Warning: Citation `octopus' on page 8 undefined on input
line 44
3.


Underfull \vbox (badness 10000) has occurred while \output is active []



No file main.bbl.


[8]

LaTeX Warning: File
`Figures/genomescope/illumina_100000/profile_genomescope_10
0000.png' not found on input line 481.


! Package pdftex.def Error: File
`Figures/genomescope/illumina_100000/profile_g
enomescope_100000.png' not found: using draft setting.

See the pdftex.def package documentation for explanation.
Type  H <return>  for immediate help.
 ...

l.481 ...na_100000/profile_genomescope_100000.png}
                                                   \\
Try typing  <return>  to proceed.
If that doesn't work, type  X <return>  to quit.


LaTeX Warning: File
`Figures/genomescope/illumina_100000/profile_genomescope_10
0000_log_notitle.jpg' not found on input line 483.


! Package pdftex.def Error: File
`Figures/genomescope/illumina_100000/profile_g
enomescope_100000_log_notitle.jpg' not found: using draft setting.

See the pdftex.def package documentation for explanation.
Type  H <return>  for immediate help.
 ...

l.483 ...ofile_genomescope_100000_log_notitle.jpg}

Try typing  <return>  to proceed.
If that doesn't work, type  X <return>  to quit.
```

```
LaTeX Warning: File `Figures/kat/kat_primary.png' not found on input line
497.


! Package pdftex.def Error: File `Figures/kat/kat_primary.png' not found:
using
 draft setting.

See the pdftex.def package documentation for explanation.
Type  H <return>  for immediate help.
 ...

l.497 ...aspectratio]{Figures/kat/kat_primary.png}

Try typing  <return>  to proceed.
If that doesn't work, type  X <return>  to quit.


LaTeX Warning: File `Figures/kat/kat_phased_haplotype1.png' not found on
input
line 502.


! Package pdftex.def Error: File `Figures/kat/kat_phased_haplotype1.png'
not fo
und: using draft setting.

See the pdftex.def package documentation for explanation.
Type  H <return>  for immediate help.
 ...

l.502 ...o]{Figures/kat/kat_phased_haplotype1.png}

Try typing  <return>  to proceed.
If that doesn't work, type  X <return>  to quit.


LaTeX Warning: File `Figures/kat/kat_phased_haplotype2.png' not found on
input
line 507.


! Package pdftex.def Error: File `Figures/kat/kat_phased_haplotype2.png'
not fo
und: using draft setting.

See the pdftex.def package documentation for explanation.
Type  H <return>  for immediate help.
 ...

l.507 ...o]{Figures/kat/kat_phased_haplotype2.png}

Try typing  <return>  to proceed.
If that doesn't work, type  X <return>  to quit.
```

```
[9

]

LaTeX Warning: File `Figures/Feulgen/Linear_regression.pdf' not found on input
line 524.


! Package pdftex.def Error: File `Figures/Feulgen/Linear_regression.pdf' not fo
und: using draft setting.

See the pdftex.def package documentation for explanation.
Type  H <return>  for immediate help.
 ...

l.524 ...5]{Figures/Feulgen/Linear_regression.pdf}

Try typing  <return>  to proceed.
If that doesn't work, type  X <return>  to quit.


LaTeX Warning: File `Figures/Feulgen/knowCvalues.pdf' not found on input line 5
33.


! Package pdftex.def Error: File `Figures/Feulgen/knowCvalues.pdf' not found: u
sing draft setting.

See the pdftex.def package documentation for explanation.
Type  H <return>  for immediate help.
 ...

l.533 ...le=0.35]{Figures/Feulgen/knowCvalues.pdf}

Try typing  <return>  to proceed.
If that doesn't work, type  X <return>  to quit.


LaTeX Warning: File `Figures/Feulgen/Botryllus-schloss.pdf' not found on input
line 541.


! Package pdftex.def Error: File `Figures/Feulgen/Botryllus-schloss.pdf' not fo
und: using draft setting.
```

```
See the pdftex.def package documentation for explanation.
Type  H <return>  for immediate help.
 ...

l.541 ...4]{Figures/Feulgen/Botryllus-schloss.pdf}

Try typing  <return>  to proceed.
If that doesn't work, type  X <return>  to quit.


LaTeX Warning: File `Figures/btk/bs_btk_all.png' not found on input line
555.


! Package pdftex.def Error: File `Figures/btk/bs_btk_all.png' not found:
using
draft setting.

See the pdftex.def package documentation for explanation.
Type  H <return>  for immediate help.
 ...

l.555 ...paspectratio]{Figures/btk/bs_btk_all.png}

Try typing  <return>  to proceed.
If that doesn't work, type  X <return>  to quit.


LaTeX Warning: File `Figures/Bs_clades_tree1.pdf' not found on input line
565.


! Package pdftex.def Error: File `Figures/Bs_clades_tree1.pdf' not found:
using
 draft setting.

See the pdftex.def package documentation for explanation.
Type  H <return>  for immediate help.
 ...

l.565 ...1\linewidth]{Figures/Bs_clades_tree1.pdf}

Try typing  <return>  to proceed.
If that doesn't work, type  X <return>  to quit.


Package natbib Warning: Citation `Salonna2021' on page 10 undefined on
input li
ne 566.


LaTeX Warning: File `Figures/annotation/Bs_Histo_3.pdf' not found on
input line
 574.
```

```
! Package pdftex.def Error: File `Figures/annotation/Bs_Histo_3.pdf' not
found:
 using draft setting.

See the pdftex.def package documentation for explanation.
Type  H <return>  for immediate help.
 ...

l.574 ...width]{Figures/annotation/Bs_Histo_3.pdf}

Try typing  <return>  to proceed.
If that doesn't work, type  X <return>  to quit.


Package natbib Warning: Citation `Kanehisa2016' on page 10 undefined on
input l
ine 576.


LaTeX Warning: File `Figures/pipeline/assembly_pipeline_phased.pdf' not
found o
n input line 584.


! Package pdftex.def Error: File
`Figures/pipeline/assembly_pipeline_phased.pdf
' not found: using draft setting.

See the pdftex.def package documentation for explanation.
Type  H <return>  for immediate help.
 ...

l.584 ...es/pipeline/assembly_pipeline_phased.pdf}

Try typing  <return>  to proceed.
If that doesn't work, type  X <return>  to quit.


LaTeX Warning: File `Figures/heatmap/asm_hap1_hic_heatmap.pdf' not found
on inp
ut line 594.


! Package pdftex.def Error: File
`Figures/heatmap/asm_hap1_hic_heatmap.pdf' not
 found: using draft setting.

See the pdftex.def package documentation for explanation.
Type  H <return>  for immediate help.
 ...

l.594 ...Figures/heatmap/asm_hap1_hic_heatmap.pdf}
```

```
Try typing  <return>  to proceed.
If that doesn't work, type  X <return>  to quit.

LaTeX Font Info:    Font shape `T1/Merriwthr-OsF/m/n' will be
(Font)              scaled to size 6.8438pt on input line 595.
LaTeX Font Info:    Font shape `T1/Merriwthr-OsF/b/n' will be
(Font)              scaled to size 6.8438pt on input line 595.

LaTeX Warning: File `Figures/heatmap/asm_hap2_hic_heatmap.pdf' not found
on inp
ut line 601.


! Package pdftex.def Error: File
`Figures/heatmap/asm_hap2_hic_heatmap.pdf' not
 found: using draft setting.

See the pdftex.def package documentation for explanation.
Type  H <return>  for immediate help.
 ...

l.601 ...Figures/heatmap/asm_hap2_hic_heatmap.pdf}

Try typing  <return>  to proceed.
If that doesn't work, type  X <return>  to quit.

LaTeX Font Info:    Font shape `T1/Merriwthr-OsF/b/it' will be
(Font)              scaled to size 7.0pt on input line 664.

Underfull \hbox (badness 7722) in paragraph at lines 669--669
[]|\T1/Merriwthr-OsF/m/n/7 (+20) No. of an-no-tated
 []


LaTeX Warning: File `Figures/palindromes/Bs1_palindrome_coverage.png' not
found
 on input line 685.


! Package pdftex.def Error: File
`Figures/palindromes/Bs1_palindrome_coverage.p
ng' not found: using draft setting.

See the pdftex.def package documentation for explanation.
Type  H <return>  for immediate help.
 ...

l.685 .../palindromes/Bs1_palindrome_coverage.png}

Try typing  <return>  to proceed.
If that doesn't work, type  X <return>  to quit.
```

```
Package natbib Warning: Citation `Wang2012' on page 10 undefined on input
line
687.



[10]

LaTeX Warning: File
`Figures/palindromes/Bs3_palindrome_coverage_limited.png' n
ot found on input line 698.


! Package pdftex.def Error: File
`Figures/palindromes/Bs3_palindrome_coverage_l
imited.png' not found: using draft setting.

See the pdftex.def package documentation for explanation.
Type  H <return>  for immediate help.
 ...

l.698 ...omes/Bs3_palindrome_coverage_limited.png}

Try typing  <return>  to proceed.
If that doesn't work, type  X <return>  to quit.


LaTeX Warning: File `Figures/palindromes/Bs3_palindrome_coverage.png' not
found
 on input line 704.


! Package pdftex.def Error: File
`Figures/palindromes/Bs3_palindrome_coverage.p
ng' not found: using draft setting.

See the pdftex.def package documentation for explanation.
Type  H <return>  for immediate help.
 ...

l.704 .../palindromes/Bs3_palindrome_coverage.png}

Try typing  <return>  to proceed.
If that doesn't work, type  X <return>  to quit.

LaTeX Font Info:    Font shape `TS1/Merriwthr-OsF/m/n' will be
(Font)              scaled to size 6.0pt on input line 708.

LaTeX Warning: Float too large for page by 135.4974pt on input line 710.


LaTeX Warning: File `Figures/palindromes/quarTeT.Bs3.pdf' not found on
input li
ne 719.
```

```
! Package pdftex.def Error: File `Figures/palindromes/quarTeT.Bs3.pdf'
not foun
d: using draft setting.

See the pdftex.def package documentation for explanation.
Type  H <return>  for immediate help.
 ...

l.719 ...dth]{Figures/palindromes/quarTeT.Bs3.pdf}

Try typing  <return>  to proceed.
If that doesn't work, type  X <return>  to quit.


LaTeX Warning: File `Figures/palindromes/Bs3_TAD.png' not found on input
line 7
25.


! Package pdftex.def Error: File `Figures/palindromes/Bs3_TAD.png' not
found: u
sing draft setting.

See the pdftex.def package documentation for explanation.
Type  H <return>  for immediate help.
 ...

l.725 ...newidth]{Figures/palindromes/Bs3_TAD.png}

Try typing  <return>  to proceed.
If that doesn't work, type  X <return>  to quit.


Package natbib Warning: Citation `Lin2023b' on page 11 undefined on input
line
729.


LaTeX Warning: File `Figures/karyotype/asm_all_karyotype.pdf' not found
on inpu
t line 740.


! Package pdftex.def Error: File
`Figures/karyotype/asm_all_karyotype.pdf' not
found: using draft setting.

See the pdftex.def package documentation for explanation.
Type  H <return>  for immediate help.
 ...

l.740 ...{Figures/karyotype/asm_all_karyotype.pdf}
```

```
Try typing  <return>  to proceed.
If that doesn't work, type  X <return>  to quit.


Package natbib Warning: Citation `Colombera1969' on page 11 undefined on
input
line 742.


LaTeX Warning: File `Figures/dotplot/hap1_vs_haploid.png' not found on
input li
ne 754.


! Package pdftex.def Error: File `Figures/dotplot/hap1_vs_haploid.png'
not foun
d: using draft setting.

See the pdftex.def package documentation for explanation.
Type  H <return>  for immediate help.
 ...

l.754 ...tio]{Figures/dotplot/hap1_vs_haploid.png}

Try typing  <return>  to proceed.
If that doesn't work, type  X <return>  to quit.


LaTeX Warning: File `Figures/dotplot/hap2_vs_haploid.png' not found on
input li
ne 760.


! Package pdftex.def Error: File `Figures/dotplot/hap2_vs_haploid.png'
not foun
d: using draft setting.

See the pdftex.def package documentation for explanation.
Type  H <return>  for immediate help.
 ...

l.760 ...tio]{Figures/dotplot/hap2_vs_haploid.png}

Try typing  <return>  to proceed.
If that doesn't work, type  X <return>  to quit.


LaTeX Warning: File `Figures/dotplot/map_asm_hap2_to_asm_hap1.png' not
found on
 input line 766.
```

```
! Package pdftex.def Error: File
`Figures/dotplot/map_asm_hap2_to_asm_hap1.png'
 not found: using draft setting.

See the pdftex.def package documentation for explanation.
Type  H <return>  for immediate help.
 ...

l.766 ...res/dotplot/map_asm_hap2_to_asm_hap1.png}

Try typing  <return>  to proceed.
If that doesn't work, type  X <return>  to quit.


Package natbib Warning: Citation `Cabanettes2018' on page 11 undefined on
input
 line 769.


LaTeX Warning: File
`Figures/Synteny/hap1_hap2/hap1_vs_hap2_highlighted_mcscanx
.pdf' not found on input line 776.


! Package pdftex.def Error: File
`Figures/Synteny/hap1_hap2/hap1_vs_hap2_highli
ghted_mcscanx.pdf' not found: using draft setting.

See the pdftex.def package documentation for explanation.
Type  H <return>  for immediate help.
 ...

l.776 ...ap2/hap1_vs_hap2_highlighted_mcscanx.pdf}

Try typing  <return>  to proceed.
If that doesn't work, type  X <return>  to quit.


Package natbib Warning: Citation `Bandi2022' on page 11 undefined on
input line
 778.


Package natbib Warning: Citation `Wang2012' on page 11 undefined on input
line
778.


LaTeX Warning: File `Figures/Synteny/dotplots_bs_sc_cr_od_V2.pdf' not
found on
input line 787.
```

```
! Package pdftex.def Error: File
`Figures/Synteny/dotplots_bs_sc_cr_od_V2.pdf'
not found: using draft setting.

See the pdftex.def package documentation for explanation.
Type  H <return>  for immediate help.
 ...

l.787 ...ures/Synteny/dotplots_bs_sc_cr_od_V2.pdf}

Try typing  <return>  to proceed.
If that doesn't work, type  X <return>  to quit.


Package natbib Warning: Citation `Simakov2020' on page 11 undefined on
input li
ne 789.


LaTeX Warning: File `Figures/Synteny/odp_BCnS_ribbon.pdf' not found on
input li
ne 797.


! Package pdftex.def Error: File `Figures/Synteny/odp_BCnS_ribbon.pdf'
not foun
d: using draft setting.

See the pdftex.def package documentation for explanation.
Type  H <return>  for immediate help.
 ...

l.797 ...dth]{Figures/Synteny/odp_BCnS_ribbon.pdf}

Try typing  <return>  to proceed.
If that doesn't work, type  X <return>  to quit.


Package natbib Warning: Citation `Simakov2022' on page 11 undefined on
input li
ne 798.


LaTeX Warning: File `Figures/Hox/Figure_S14_bootstraps2.pdf' not found on
input
 line 807.


! Package pdftex.def Error: File `Figures/Hox/Figure_S14_bootstraps2.pdf'
not f
ound: using draft setting.

See the pdftex.def package documentation for explanation.
Type  H <return>  for immediate help.
```

```
 ...

l.807 ...]{Figures/Hox/Figure_S14_bootstraps2.pdf}

Try typing  <return>  to proceed.
If that doesn't work, type  X <return>  to quit.


Overfull \hbox (146.64438pt too wide) in paragraph at lines 807--808
 [][]
 []


Package natbib Warning: Citation `Minh2020' on page 11 undefined on input
line
808.


Package natbib Warning: Citation `Sekigami2017' on page 11 undefined on
input l
ine 808.


LaTeX Warning: File `Figures/Hox/Bayesian_tree.pdf' not found on input
line 819
.


! Package pdftex.def Error: File `Figures/Hox/Bayesian_tree.pdf' not
found: usi
ng draft setting.

See the pdftex.def package documentation for explanation.
Type  H <return>  for immediate help.
 ...

l.819 ...linewidth]{Figures/Hox/Bayesian_tree.pdf}

Try typing  <return>  to proceed.
If that doesn't work, type  X <return>  to quit.


Overfull \hbox (146.64438pt too wide) in paragraph at lines 819--820
 [][]
 []


Package natbib Warning: Citation `Ronquist2003' on page 11 undefined on
input l
ine 820.


Package natbib Warning: Citation `Sekigami2017' on page 11 undefined on
input l
```

```
ine 820.


Package natbib Warning: There were undefined citations.



[11]

[12]

[13]

[14]

[15]

[16]

[17]

[18]

[19]

[20]

[21]

[22]

[23]

[24]

[25]
enddocument/afterlastpage: lastpage setting LastPage.
(./main.aux)
 ***********
LaTeX2e <2024-06-01> patch level 2
L3 programming layer <2020/03/25>
 ***********

LaTeX Font Warning: Some font shapes were not available, defaults
substituted.

Package rerunfilecheck Info: File `main.out' has not changed.
(rerunfilecheck)             Checksum:
0750DDA0578EBF4837FD48868422C777;1384.
 )
Here is how much of TeX's memory you used:
 36404 strings out of 473583
 771595 string characters out of 5732343
 1983908 words of memory out of 5000000
```

```
 57737 multiletter control sequences out of 15000+600000
 1965631 words of font info for 735 fonts, out of 8000000 for 9000
 1141 hyphenation exceptions out of 8191
 123i,19n,131p,2494b,960s stack positions out of
10000i,1000n,20000p,200000b,200000s
<c:/texlive/2024/texmf-dist/fonts/type1/sorkin/merriweather/Merriwthr-
Bold.pf
b><c:/texlive/2024/texmf-dist/fonts/type1/sorkin/merriweather/Merriwthr-
BoldIta
lic.pfb><c:/texlive/2024/texmf-
dist/fonts/type1/sorkin/merriweather/Merriwthr-I
talic.pfb><c:/texlive/2024/texmf-
dist/fonts/type1/sorkin/merriweather/Merriwthr
-Regular.pfb><c:/texlive/2024/texmf-
dist/fonts/type1/public/amsfonts/euler/eusm
7.pfb><c:/texlive/2024/texmf-dist/fonts/type1/public/lm/lmtt8.pfb>
Output written on main.pdf (25 pages, 359461 bytes).
PDF statistics:
 299 PDF objects out of 1000 (max. 8388607)
 256 compressed objects within 3 object streams
 72 named destinations out of 1000 (max. 500000)
 215645 words of extra memory for PDF output out of 221844 (max.
10000000)
```

Figure 1

Click here to access/download;LaTeX - Figure (eps, ps, etc.);ImageBS1.jpg

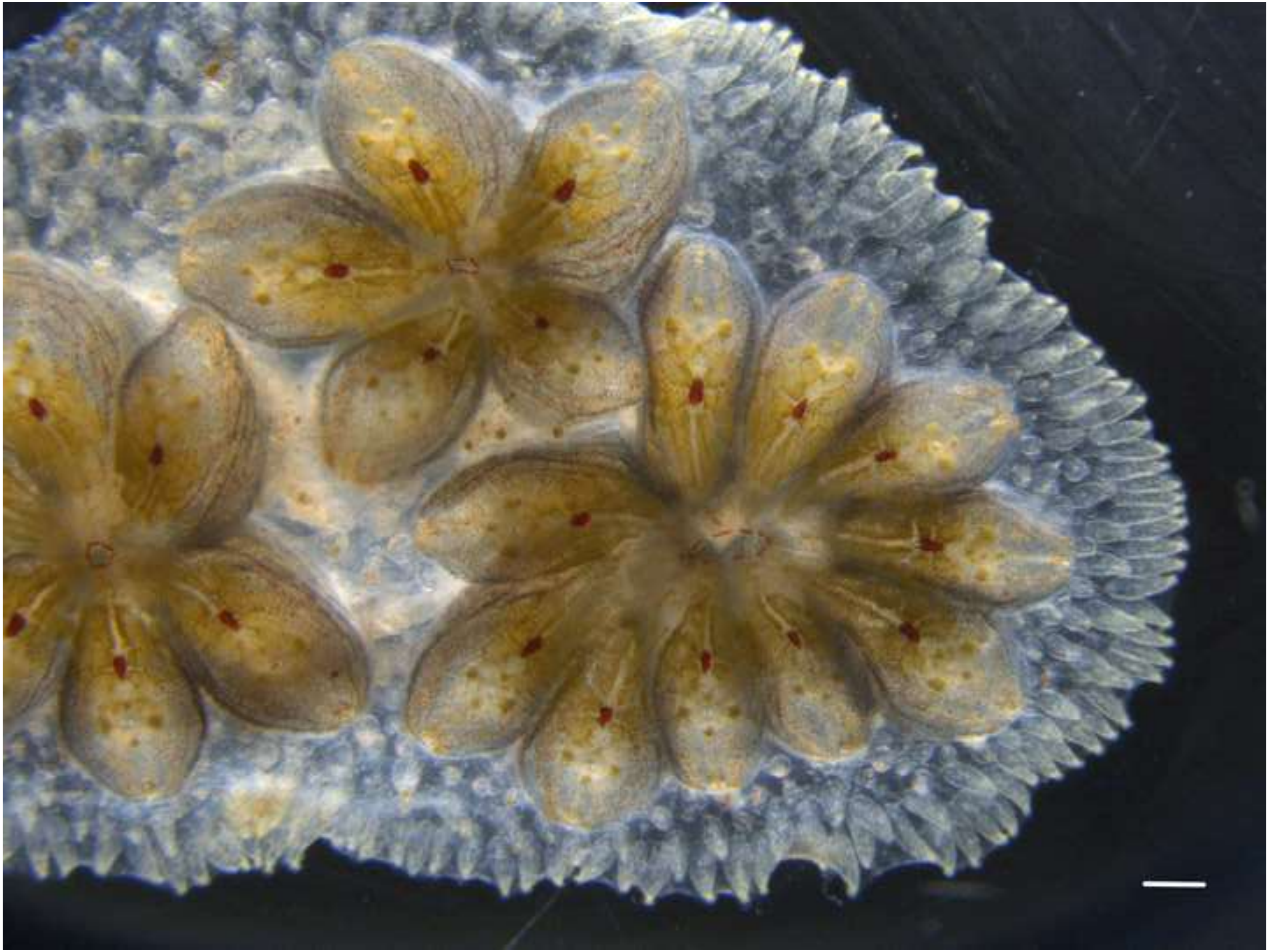

Figure 2

Click here to access/download;LaTeX - Figure (eps, ps;

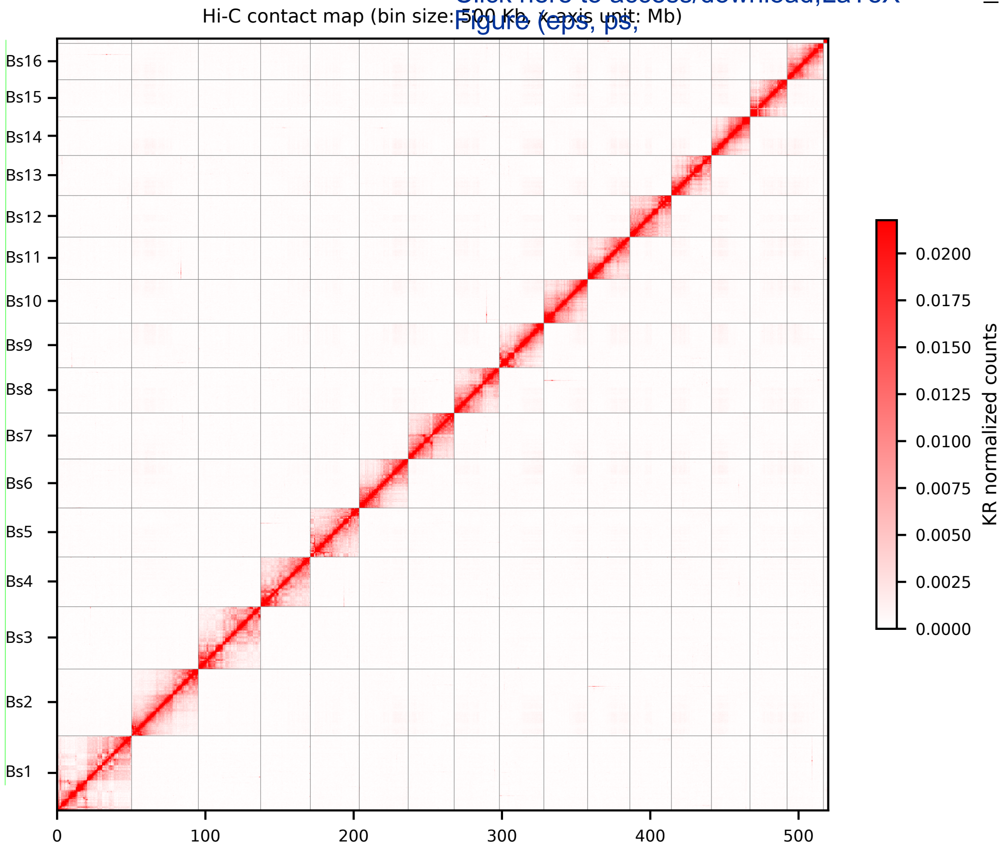

Figure 3

Click here to access/download;LaTeX - Figure (eps, ps, etc.);bs_circos_plot.pdf

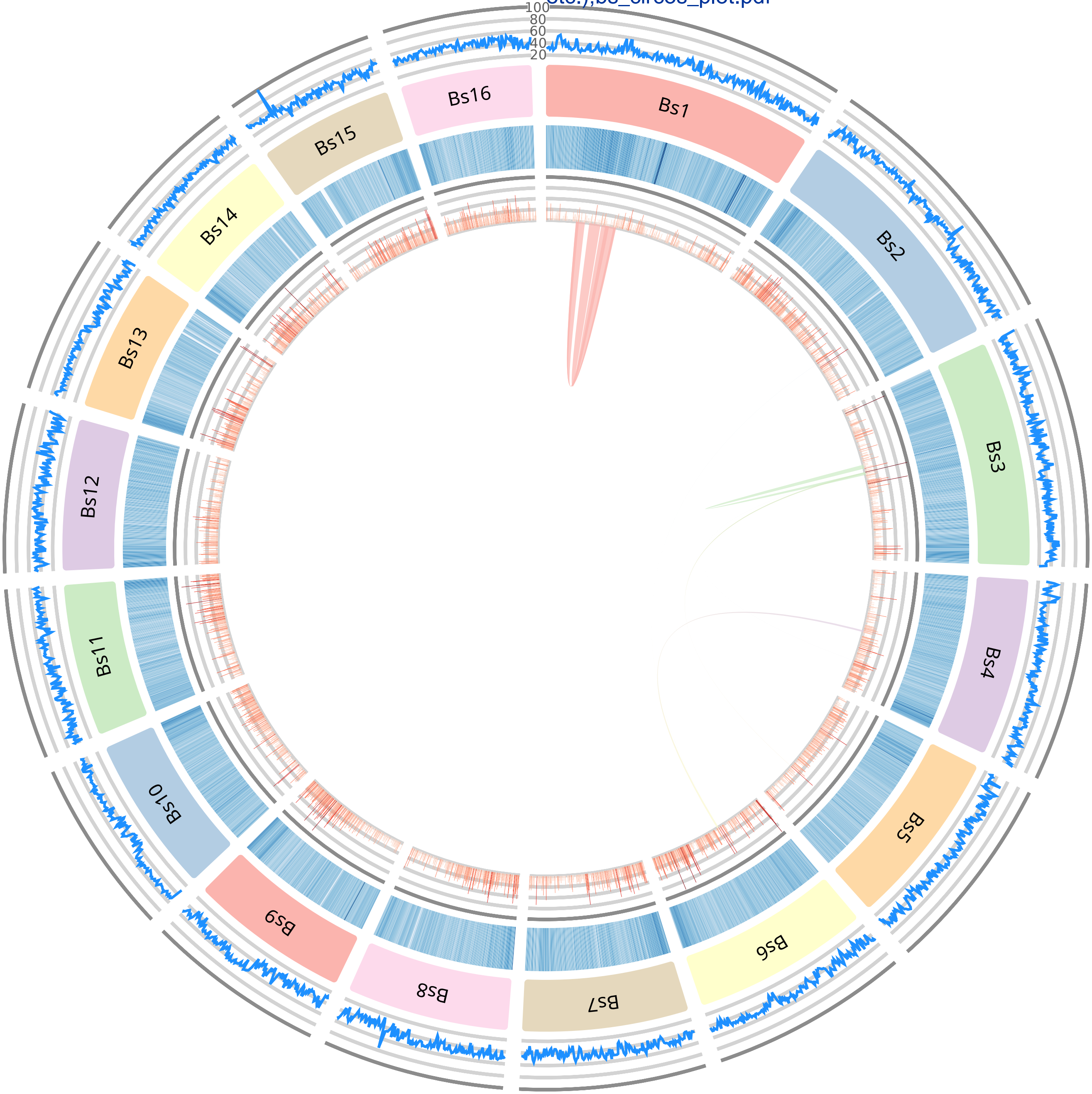

Figure 4

Click here to access/download;LaTeX - Figure

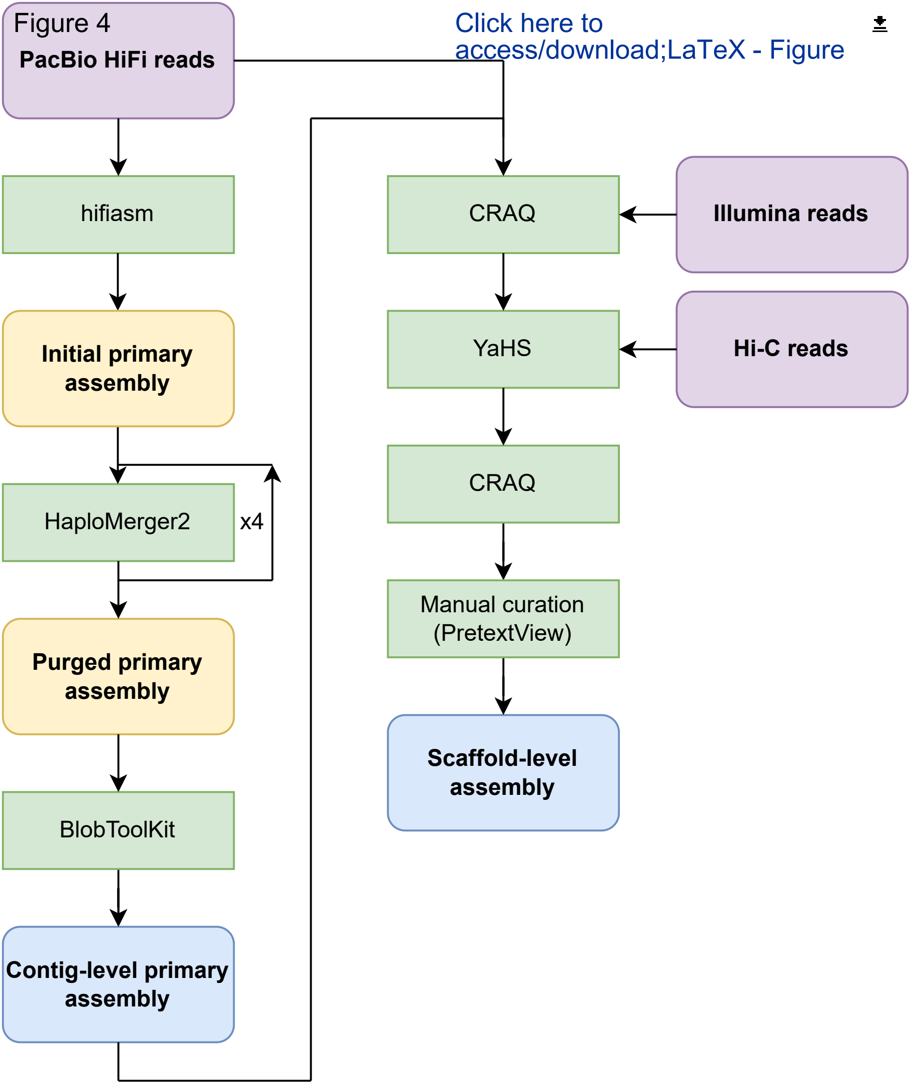

Figure 5

Click here to access/download;LaTeX - Figure (eps, ps, etc.);busco_assembly.png

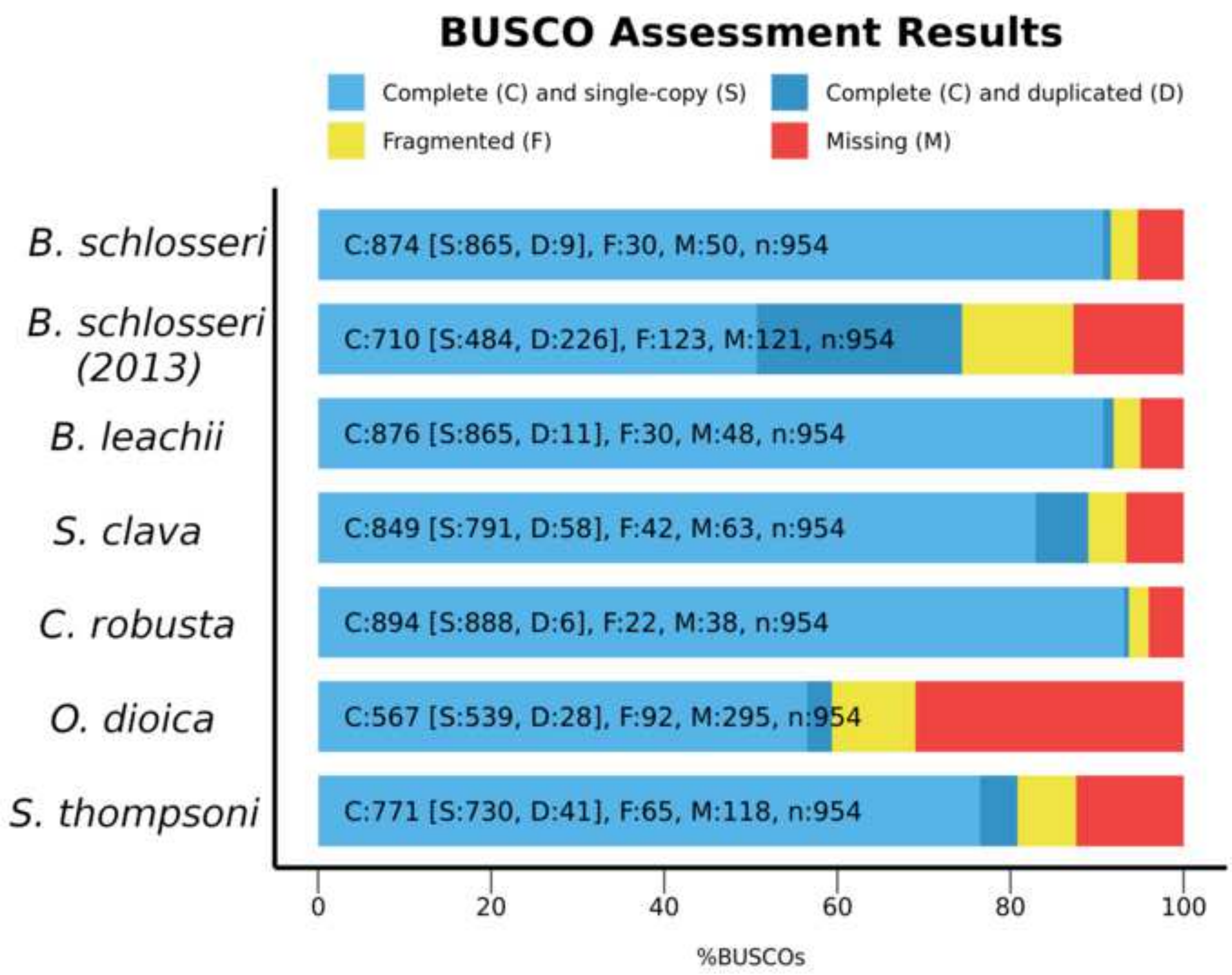

Figure 6

Click here to access/download;LaTeX - Figure (eps, ps, etc.);PIE_KOALA.png

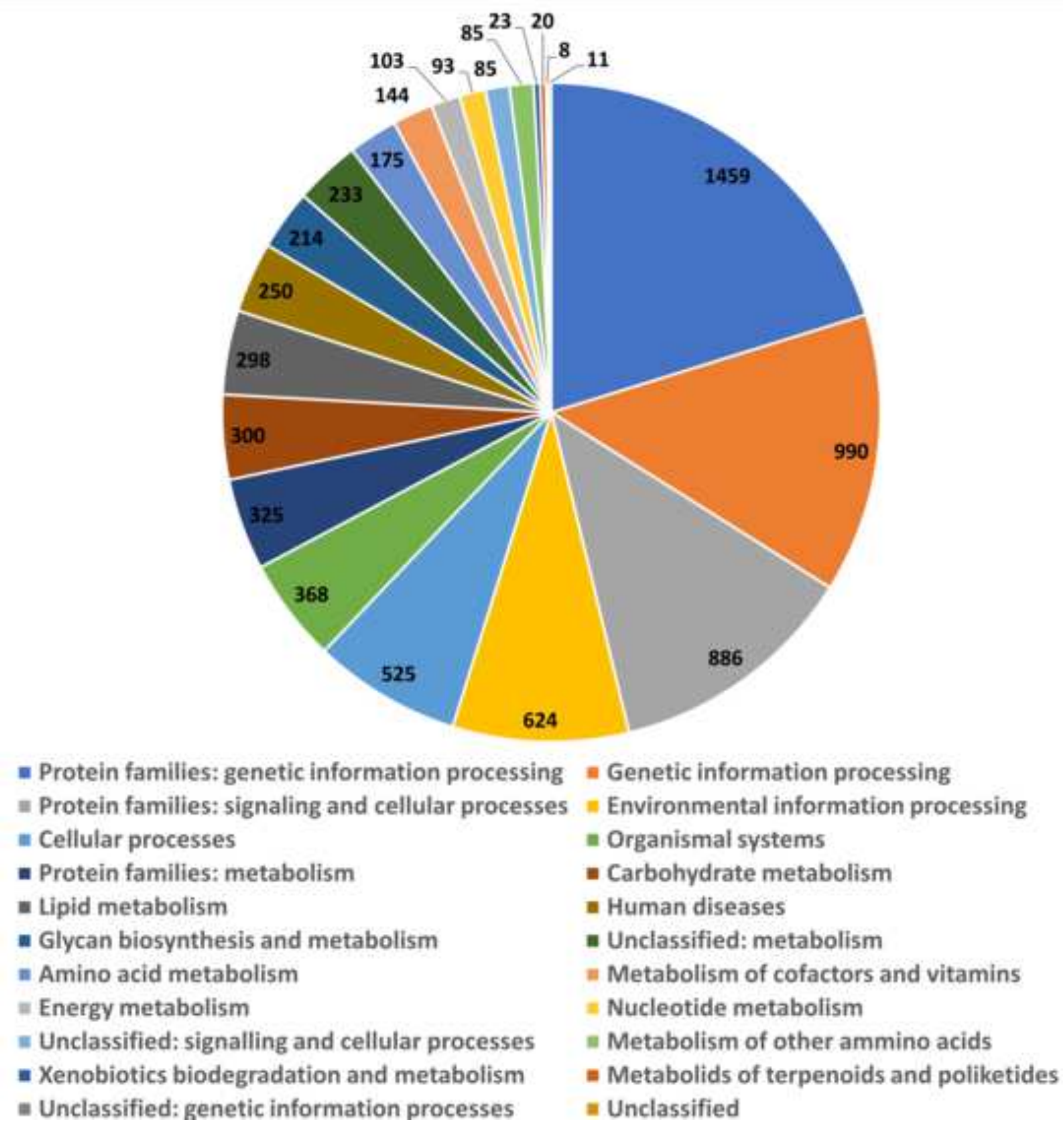

Figure 7

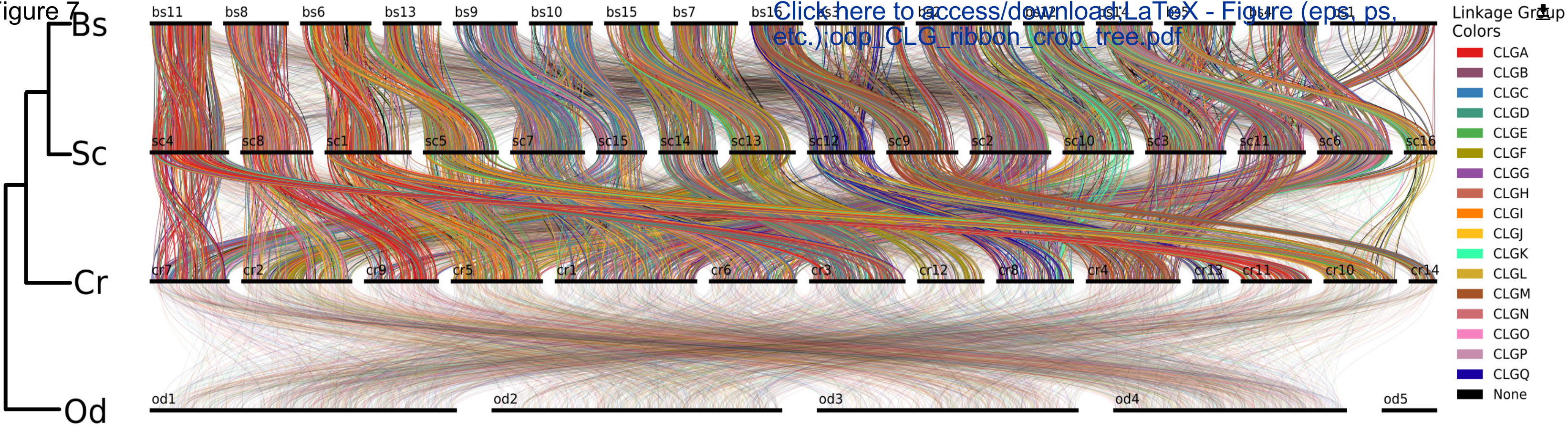

Click here to access/download;LaTeX - Figure (eps, ps, etc.);odp_CLG_ribbon_crop_tree.pdf

Figure 8

Click here to access/download;LaTeX - Figure (eps, ps, etc.);hox_20072025_ST2.drawio.pdf

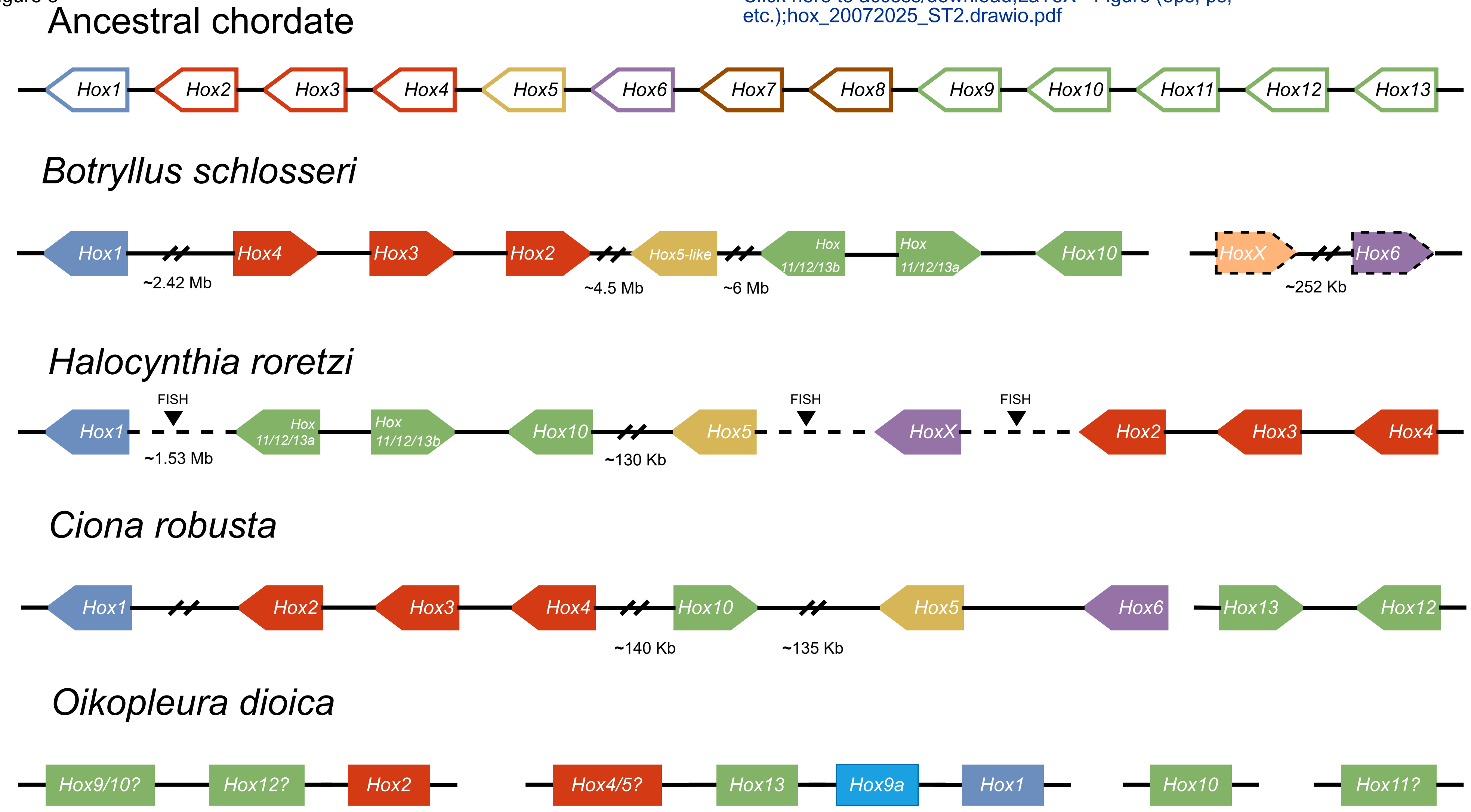

Supp. Figure 1a

Click here to access/download;LaTeX - Figure (eps, ps, etc.);profile_genomescope_100000.png

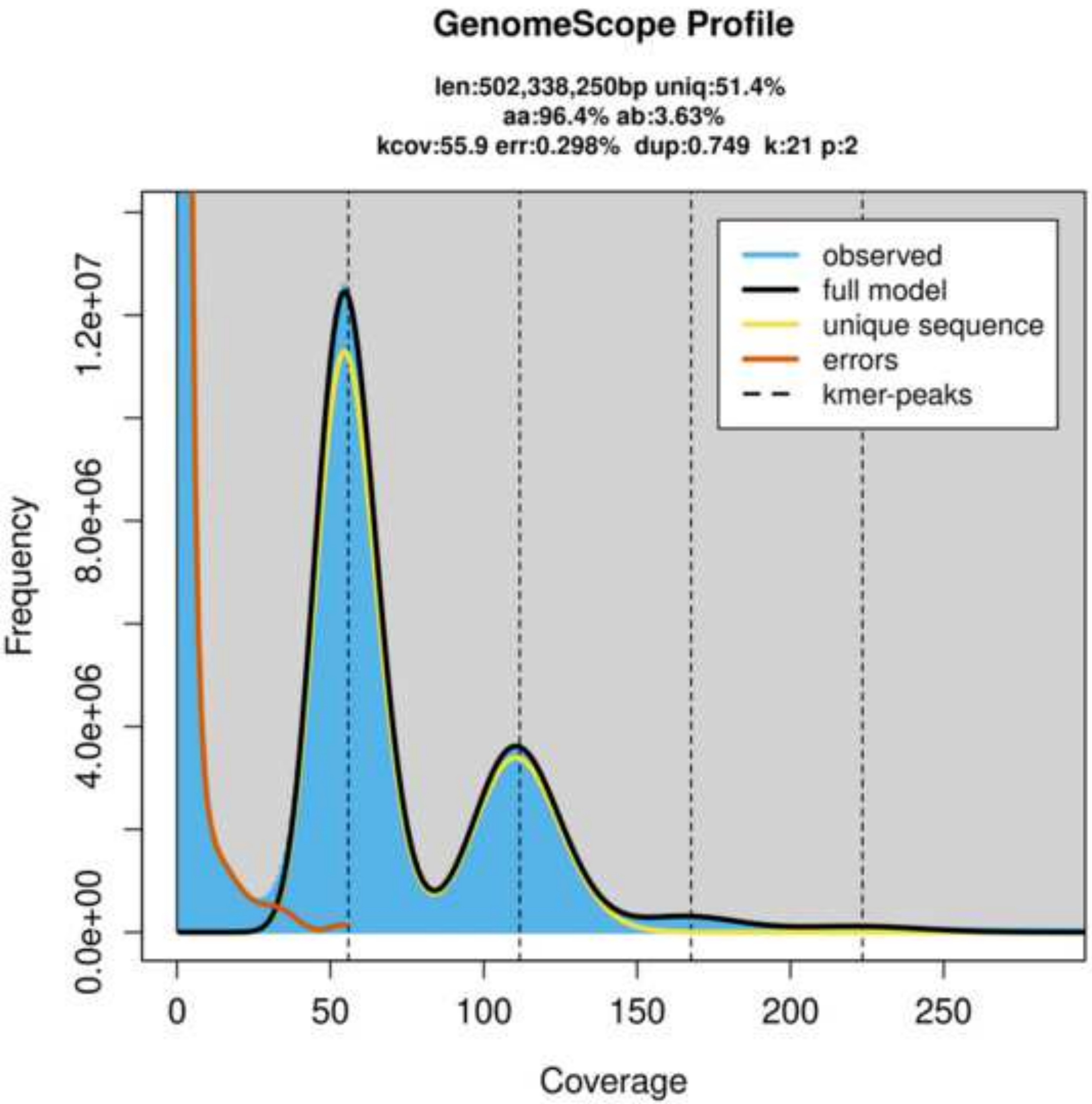

Supp. Figure 1b

Click here to access/download;LaTeX - Figure (eps, ps, etc.);profile_genomescope_100000_log_notitle.jpg

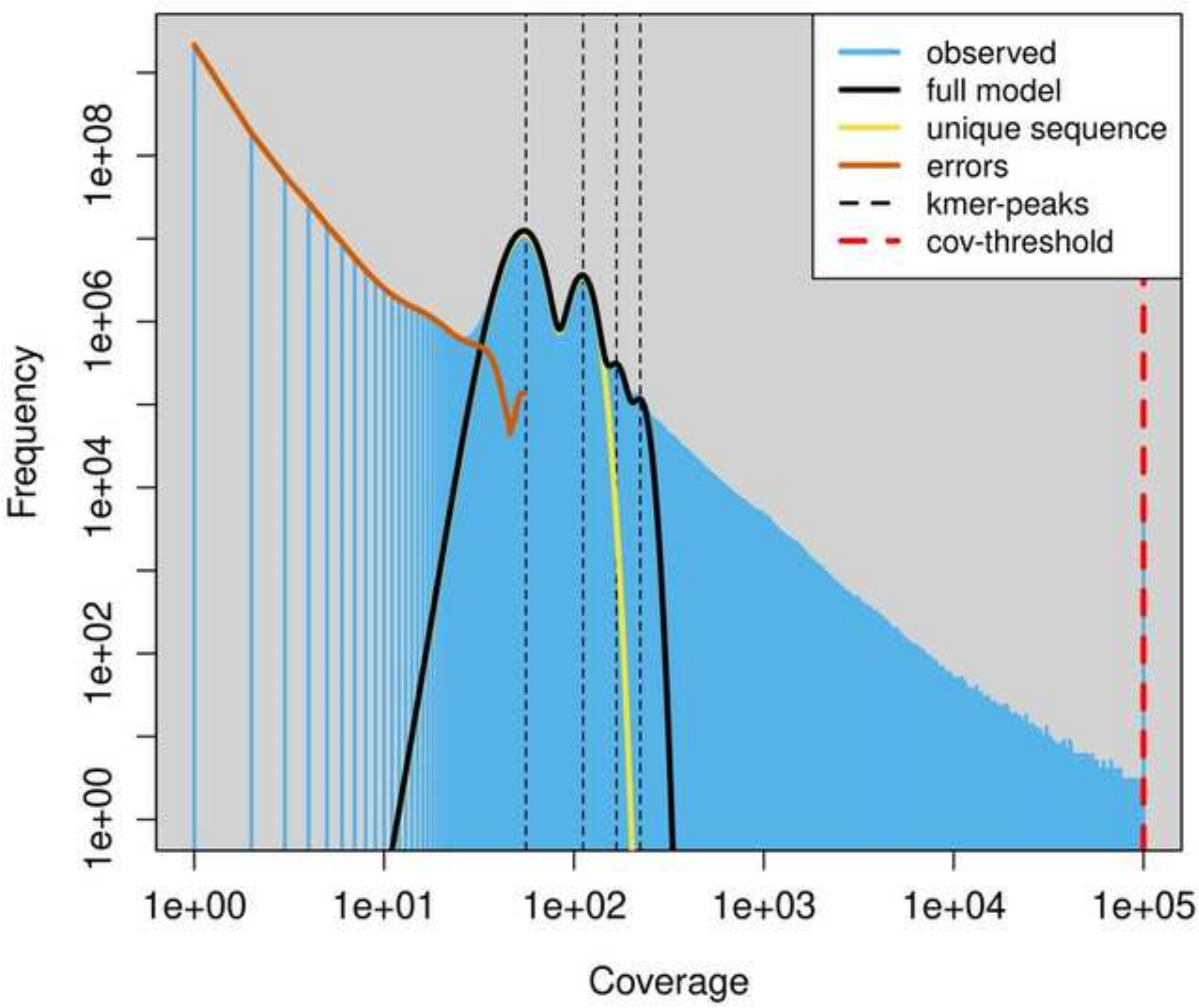

Supp. Figure 2a

Click here to access/download;LaTeX - Figure (eps, ps, etc.);kat_primary.png

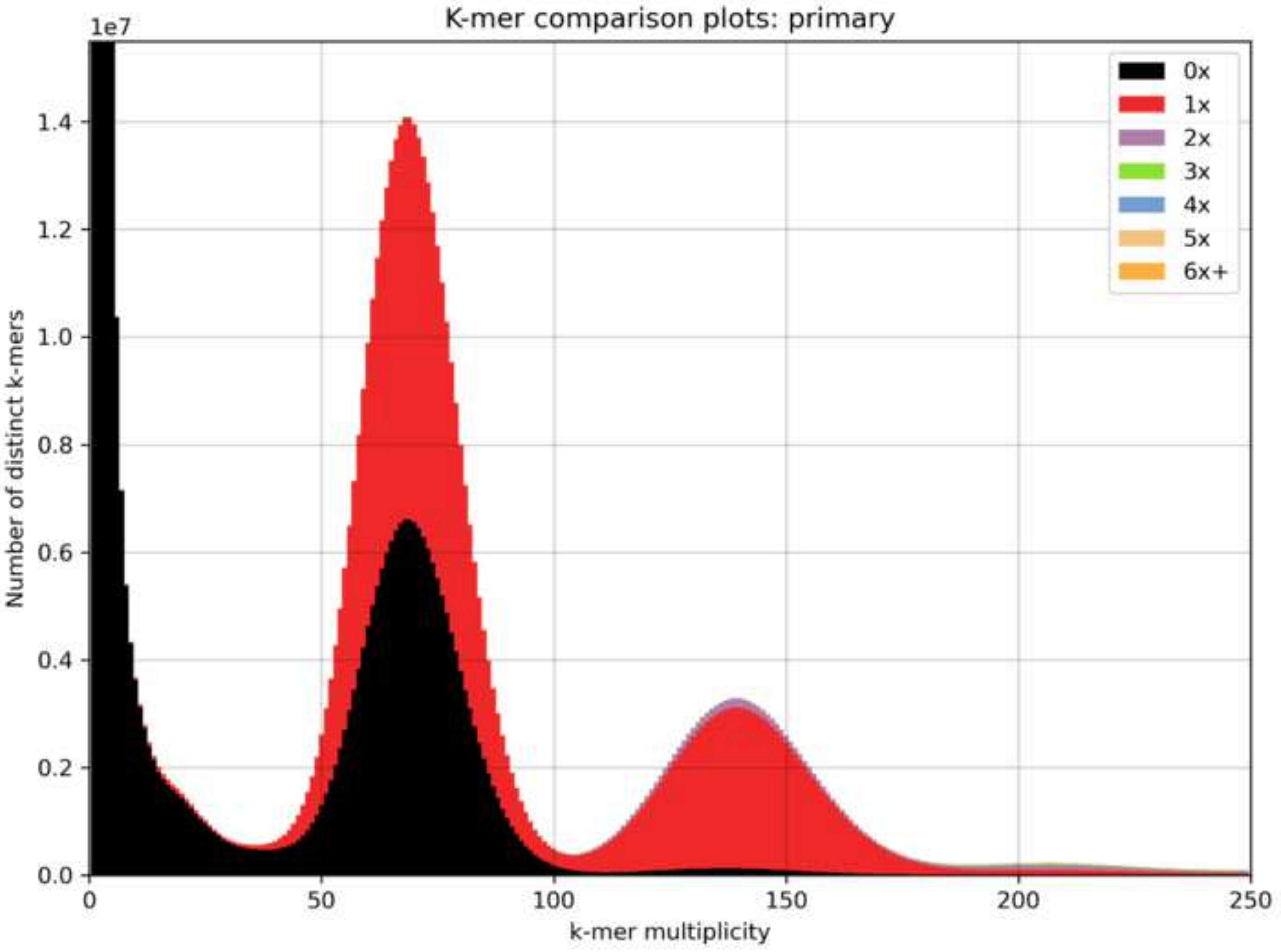

Supp. Figure 2b

Click here to access/download;LaTeX - Figure (eps, ps, etc.);kat_phased_haplotype1.png

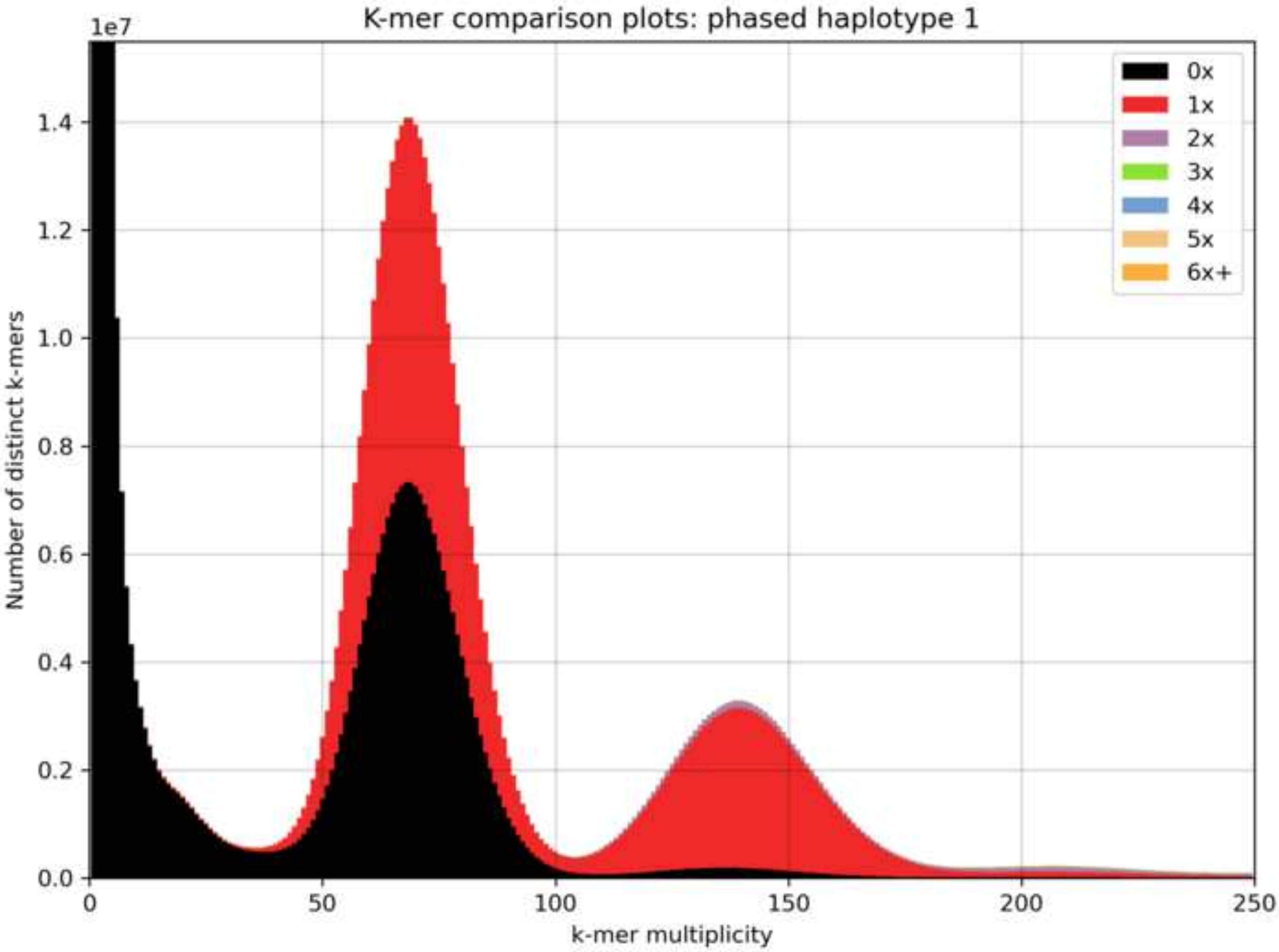

Supp. Figure 2c

Click here to access/download;LaTeX - Figure (eps, ps, etc.);kat_phased_haplotype2.png

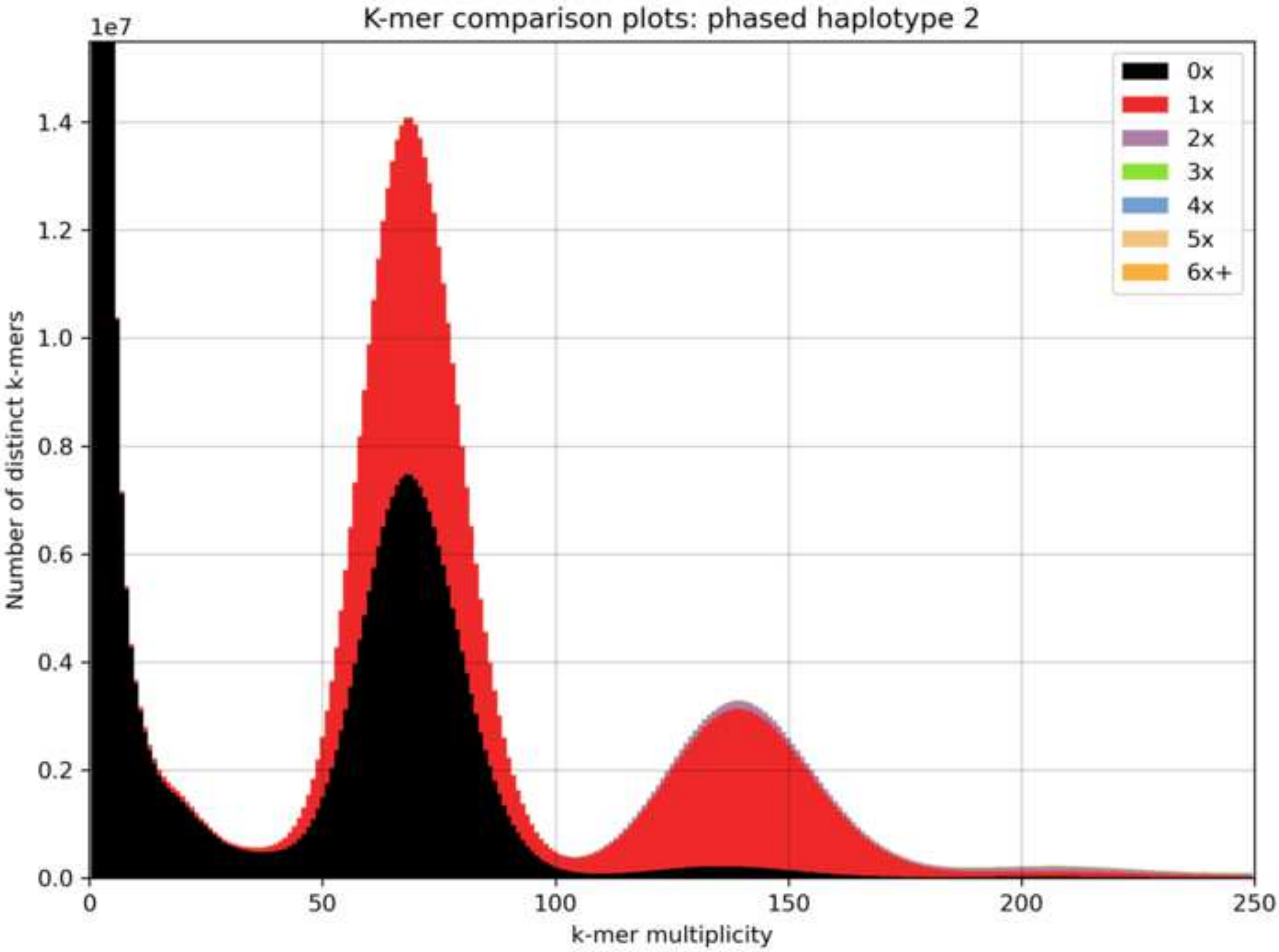

Supp. Figure 3

Click here to access/download;LaTeX - Figure (eps, ps, etc.);Linear_regression.pdf

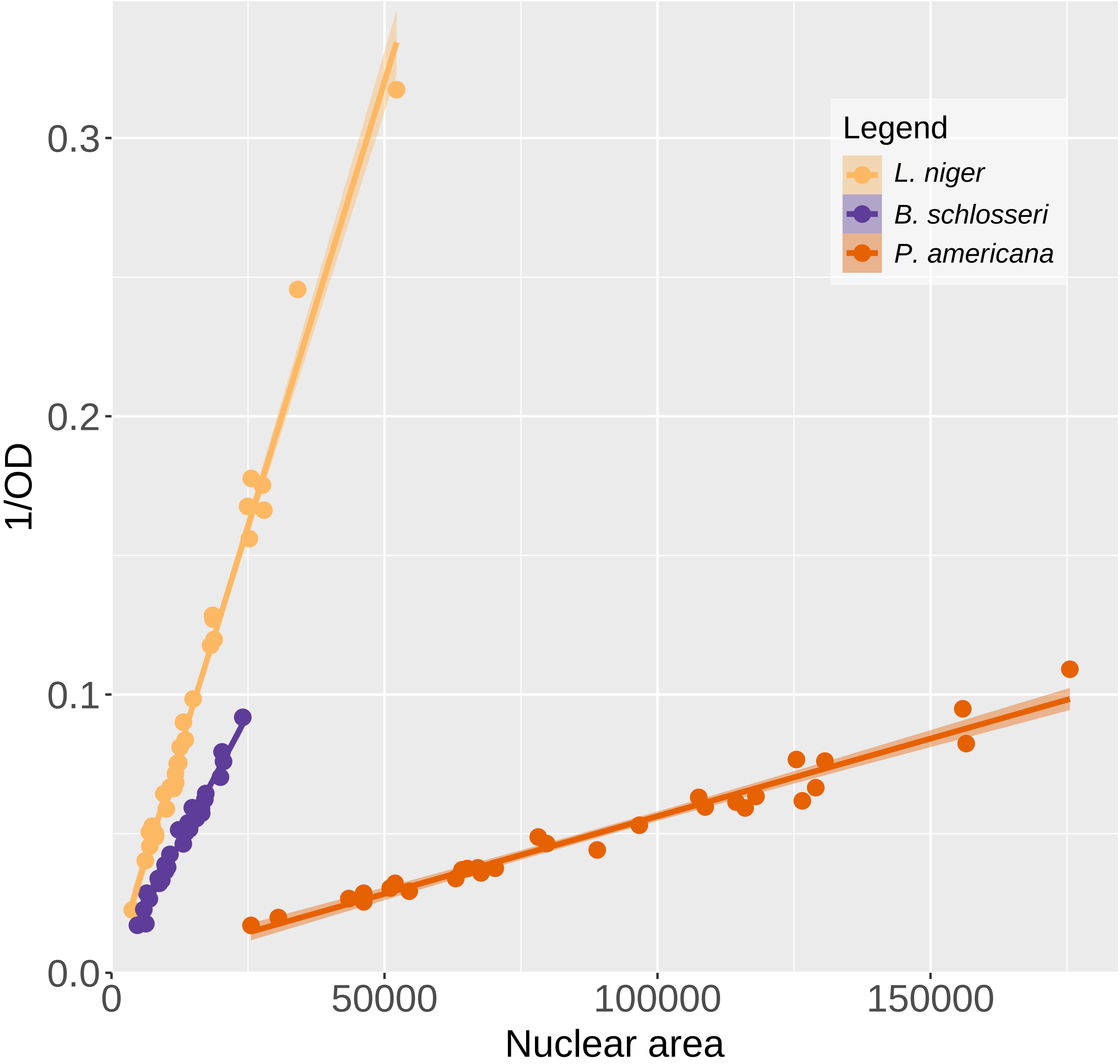

Supp. Figure 4

Click here to access/download;LaTeX - Figure (eps, ps, etc.);knowCvalues.pdf

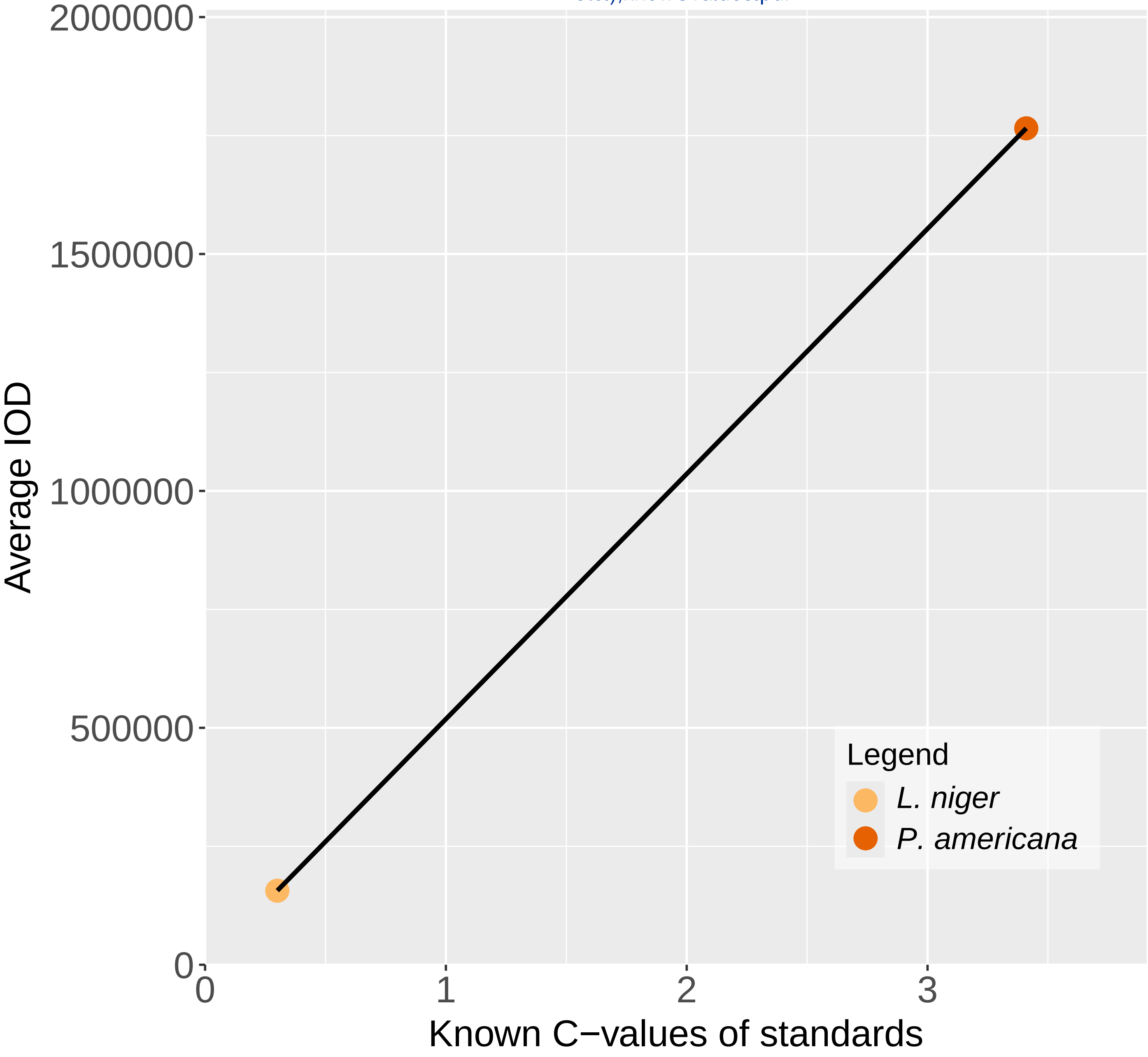

Supp. Figure 5

Click here to access/download;LaTeX - Figure (eps, ps, etc.);Botryllus-schloss.pdf

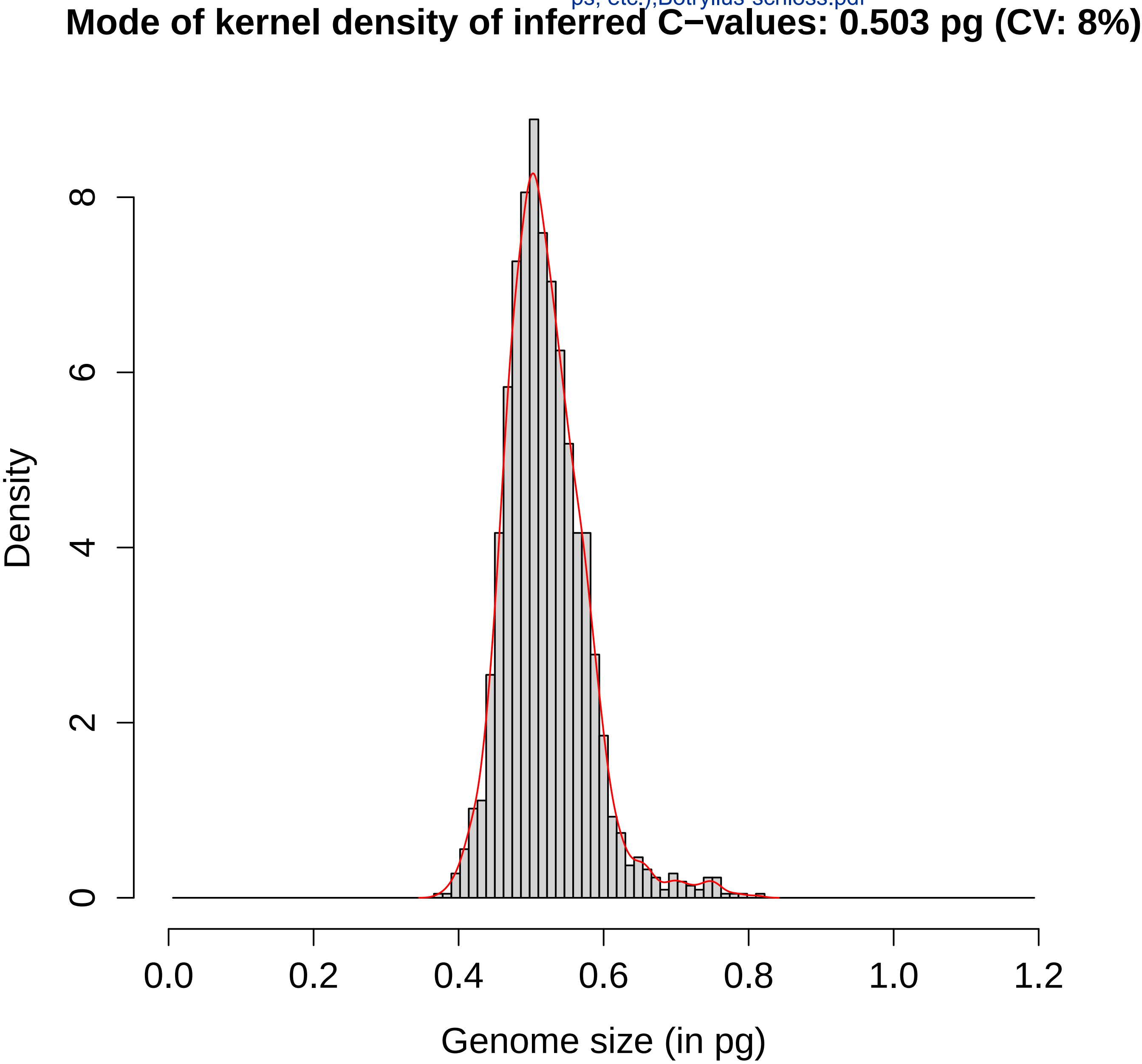

Supp. Figure 6

Click here to access/download;LaTeX - Figure (eps, ps, etc.);bs_btk_all.png

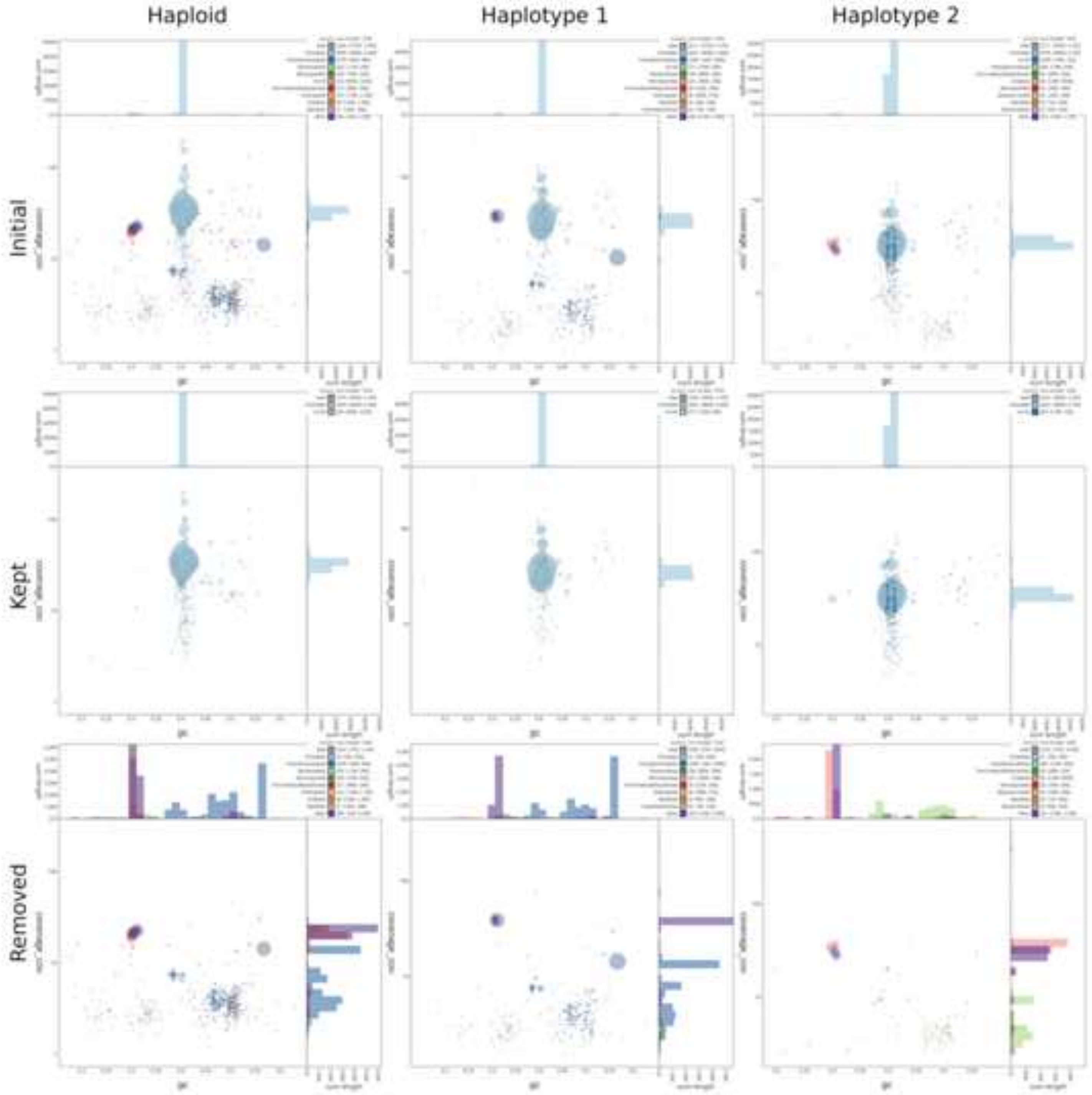

Supp. Figure 7

Click here to access/download;LaTeX - Figure (eps, ps, etc.);Bs_clades_tree1.pdf

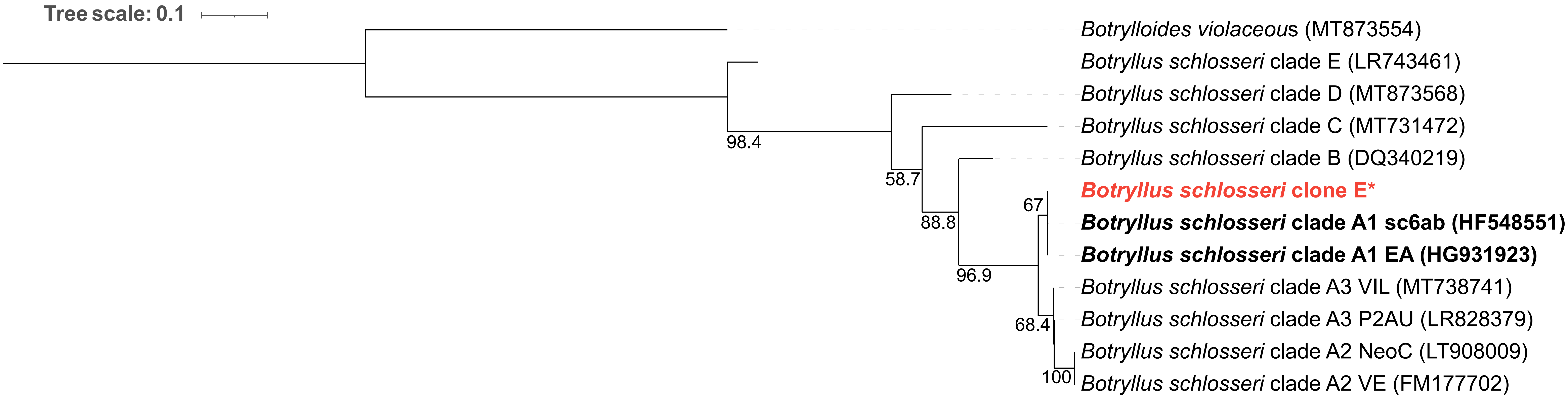

Supp. Figure 8

Click here to access/download;LaTeX - Figure (eps, ps, etc.);Bs_Histo_3.pdf

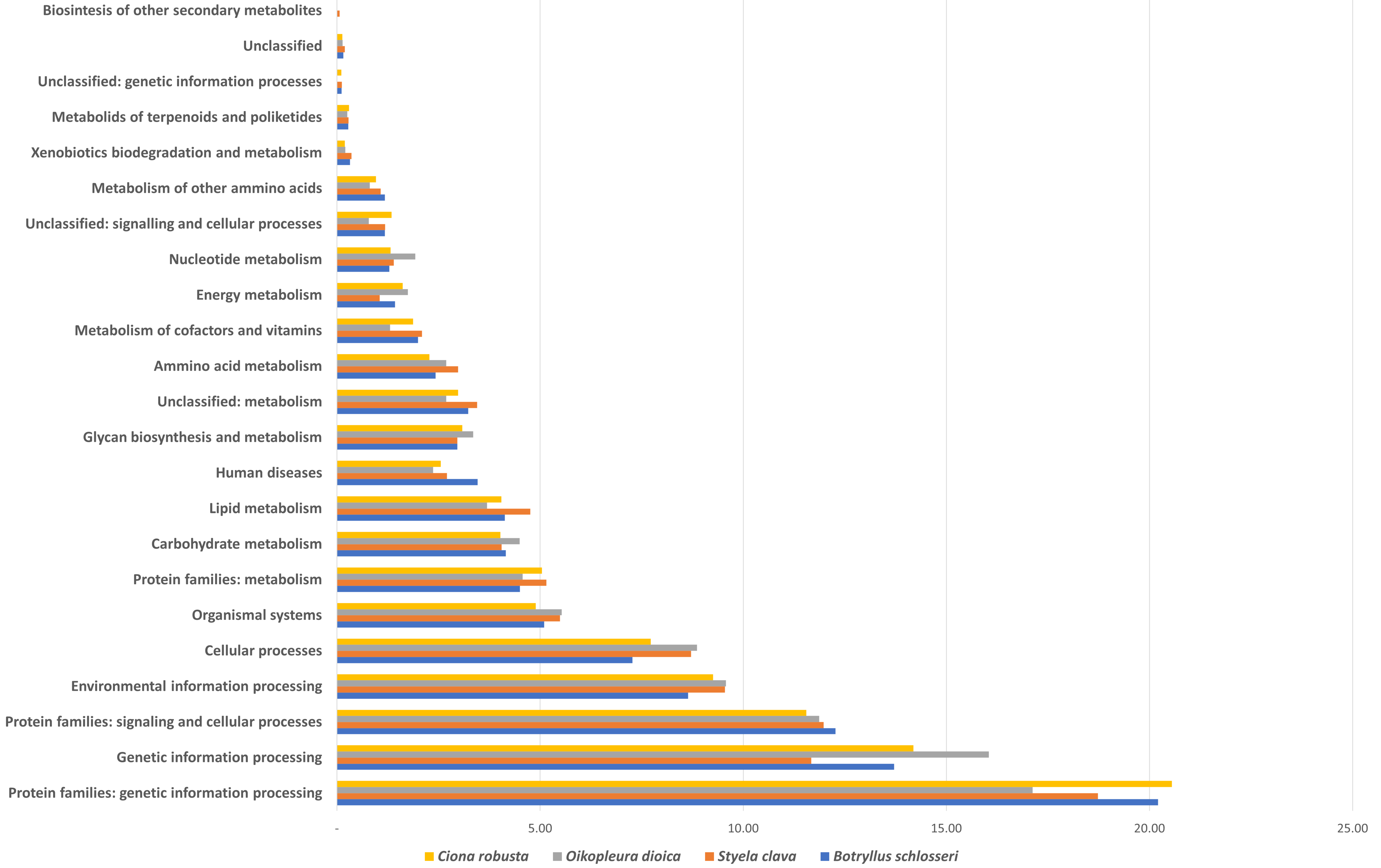

Supp. Figure 9

Click here to access/download;L

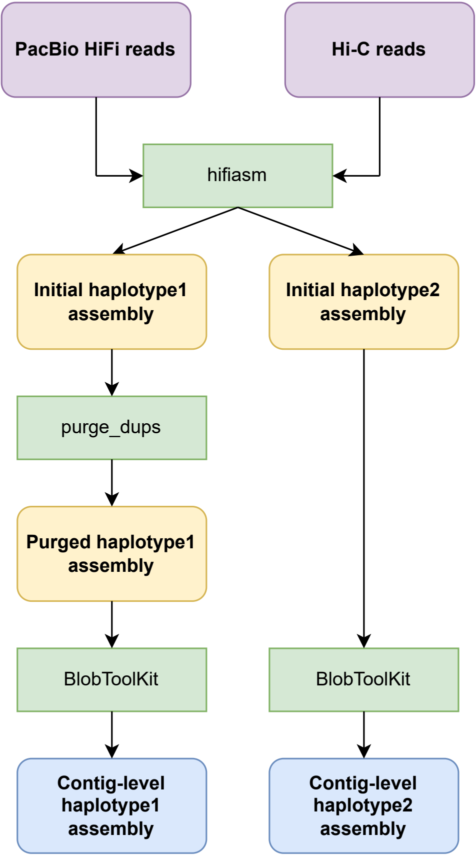

Supp. Figure 10a

Click here to access/download;LaTeX - Figure (eps, ps;

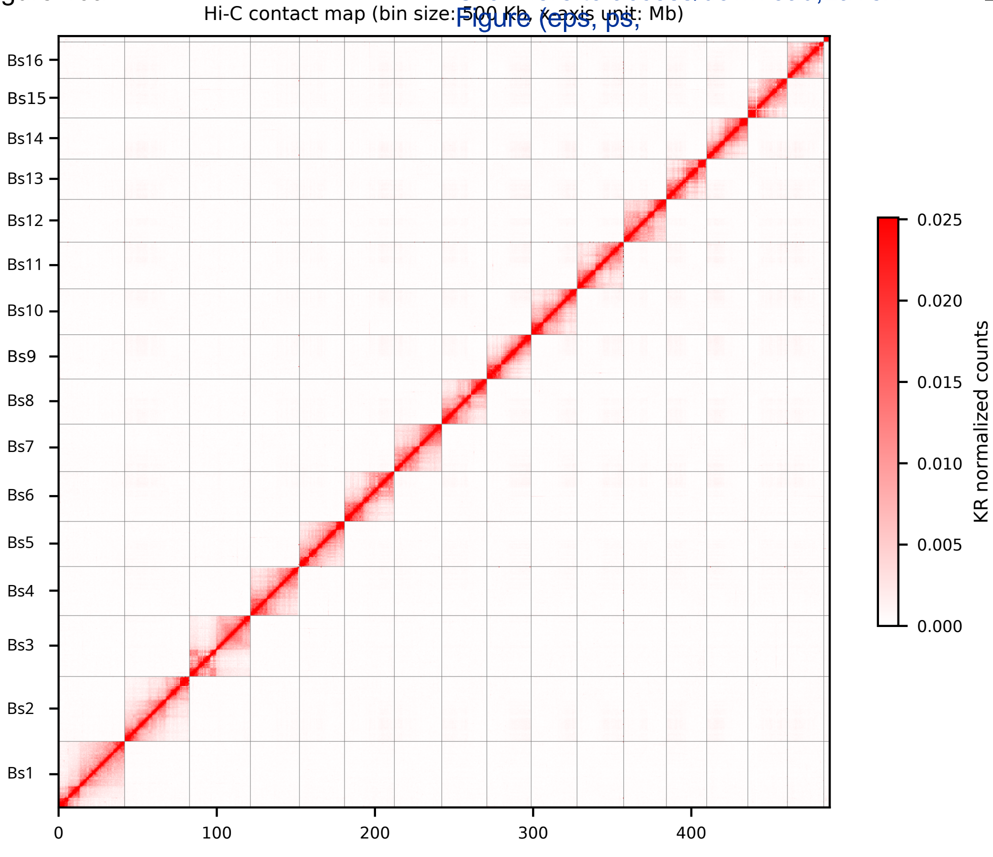

Supp. Figure 10b

Click here to access/download;LaTeX - Figure (eps, ps;

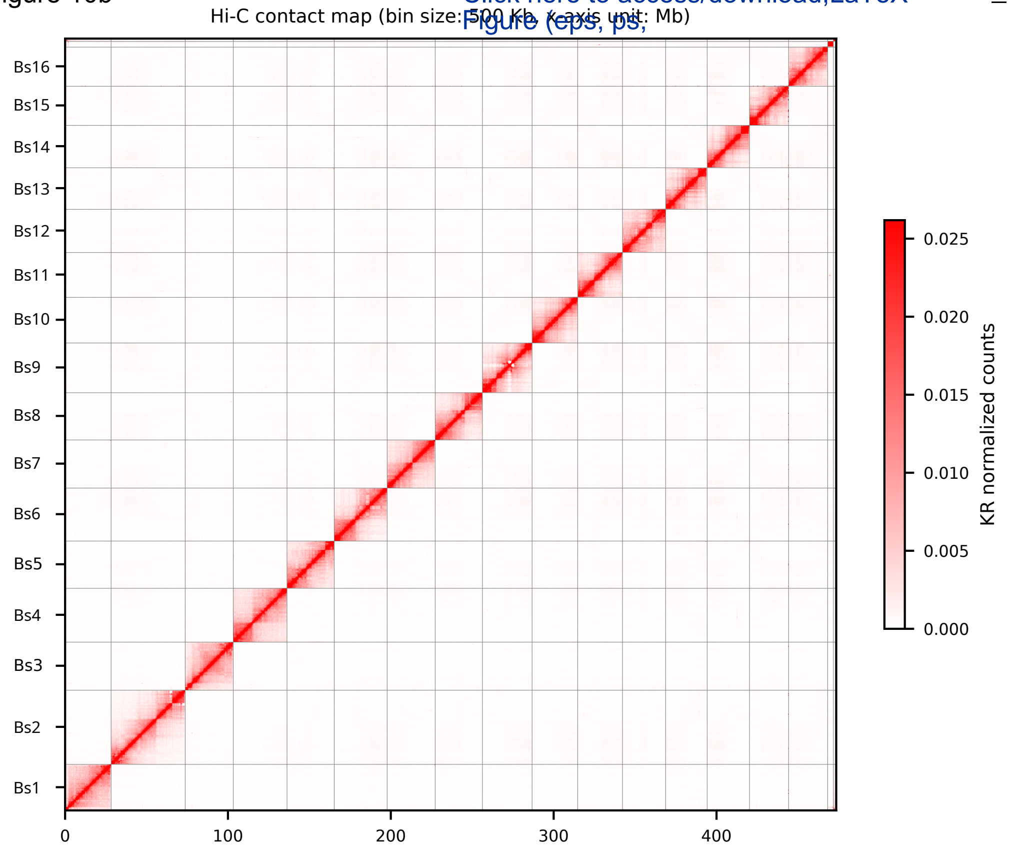

Supp. Figure 11

Click here to access/download;LaTeX - Figure (eps, ps, etc.);Bs1_palindrome_coverage.png

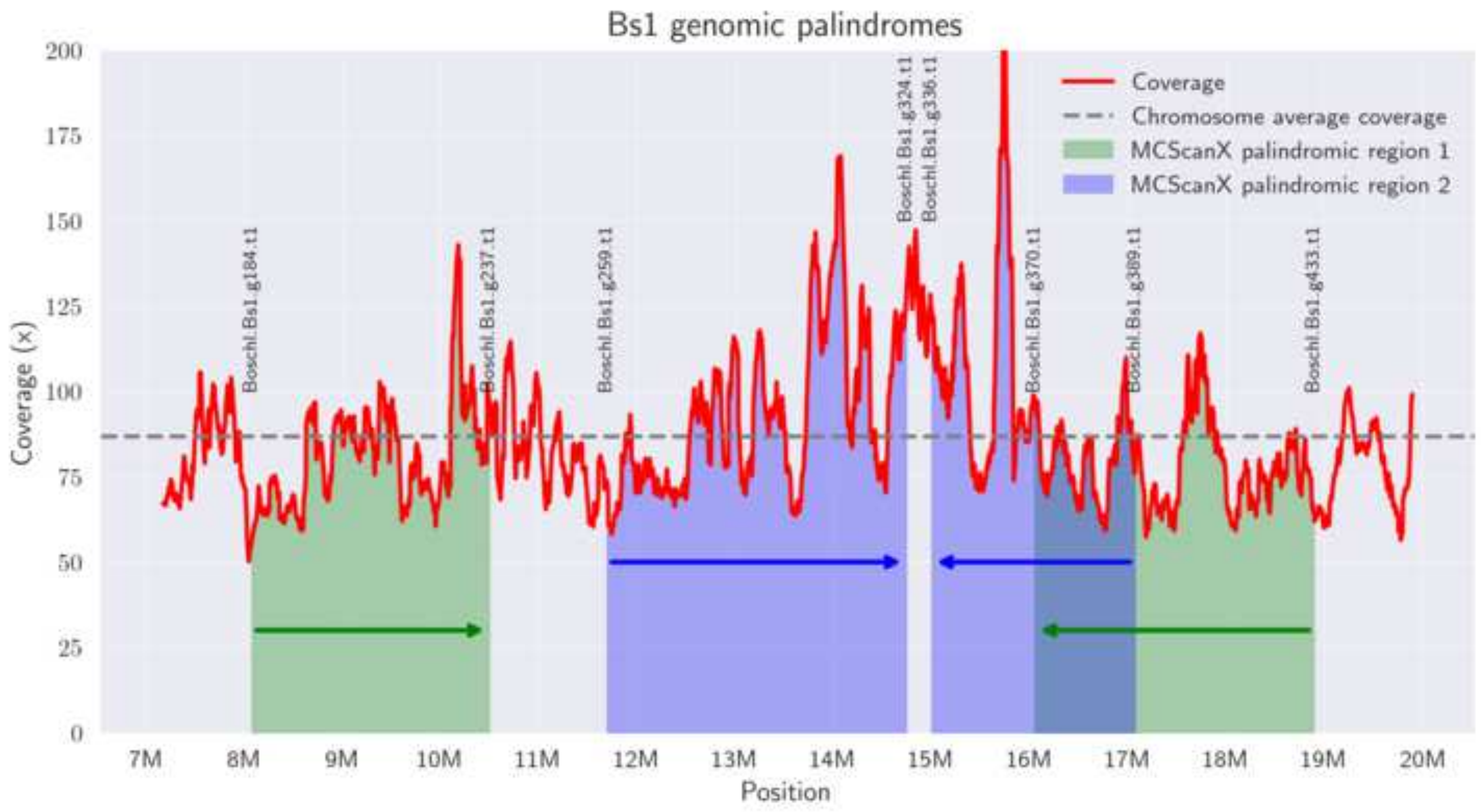

Supp. Figure 12a

Click here to access/download;LaTeX - Figure (eps, ps, etc.);Bs3_palindrome_coverage_limited.png

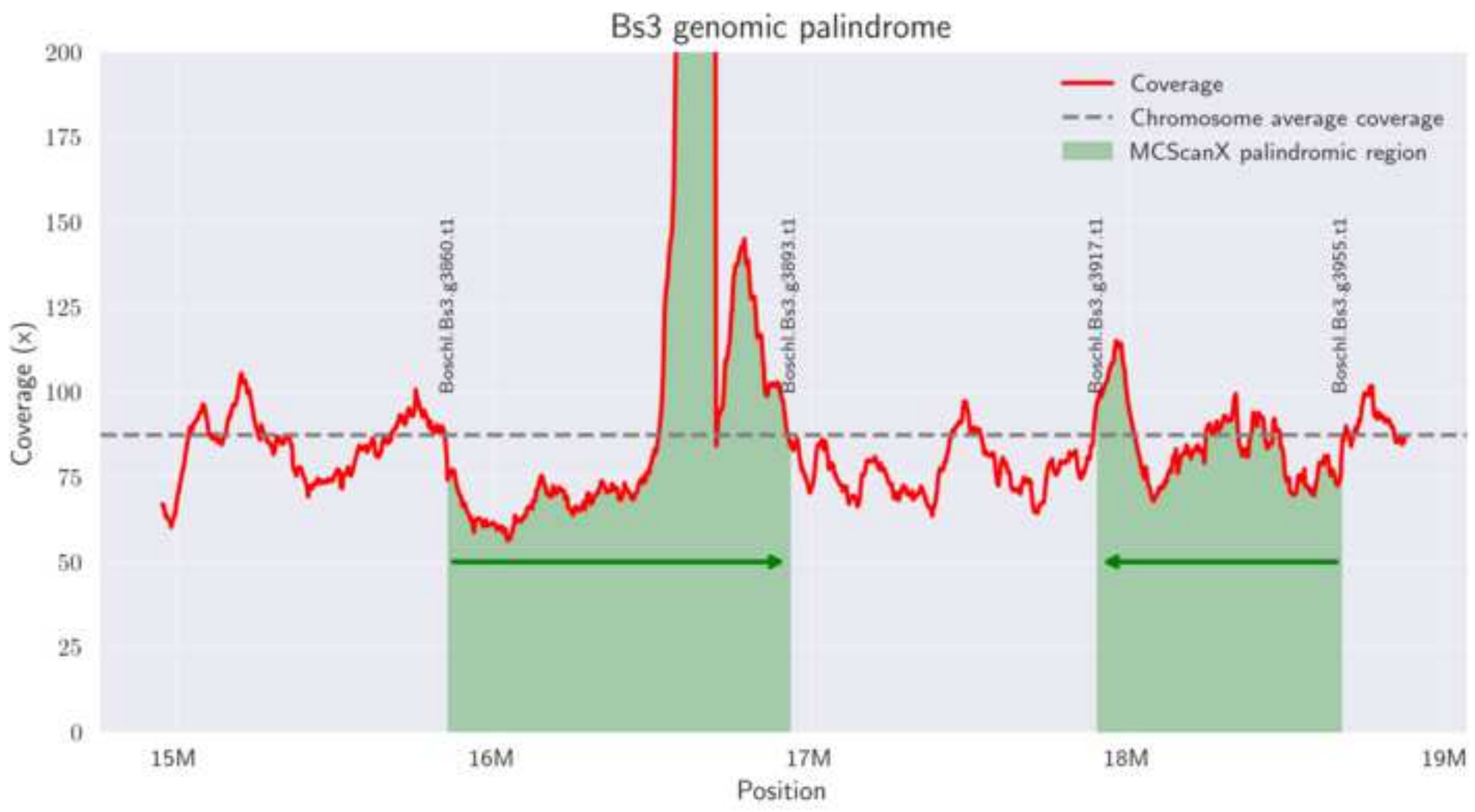

Supp. Figure 12b

Click here to access/download;LaTeX - Figure (eps, ps, etc.);Bs3_palindrome_coverage.png

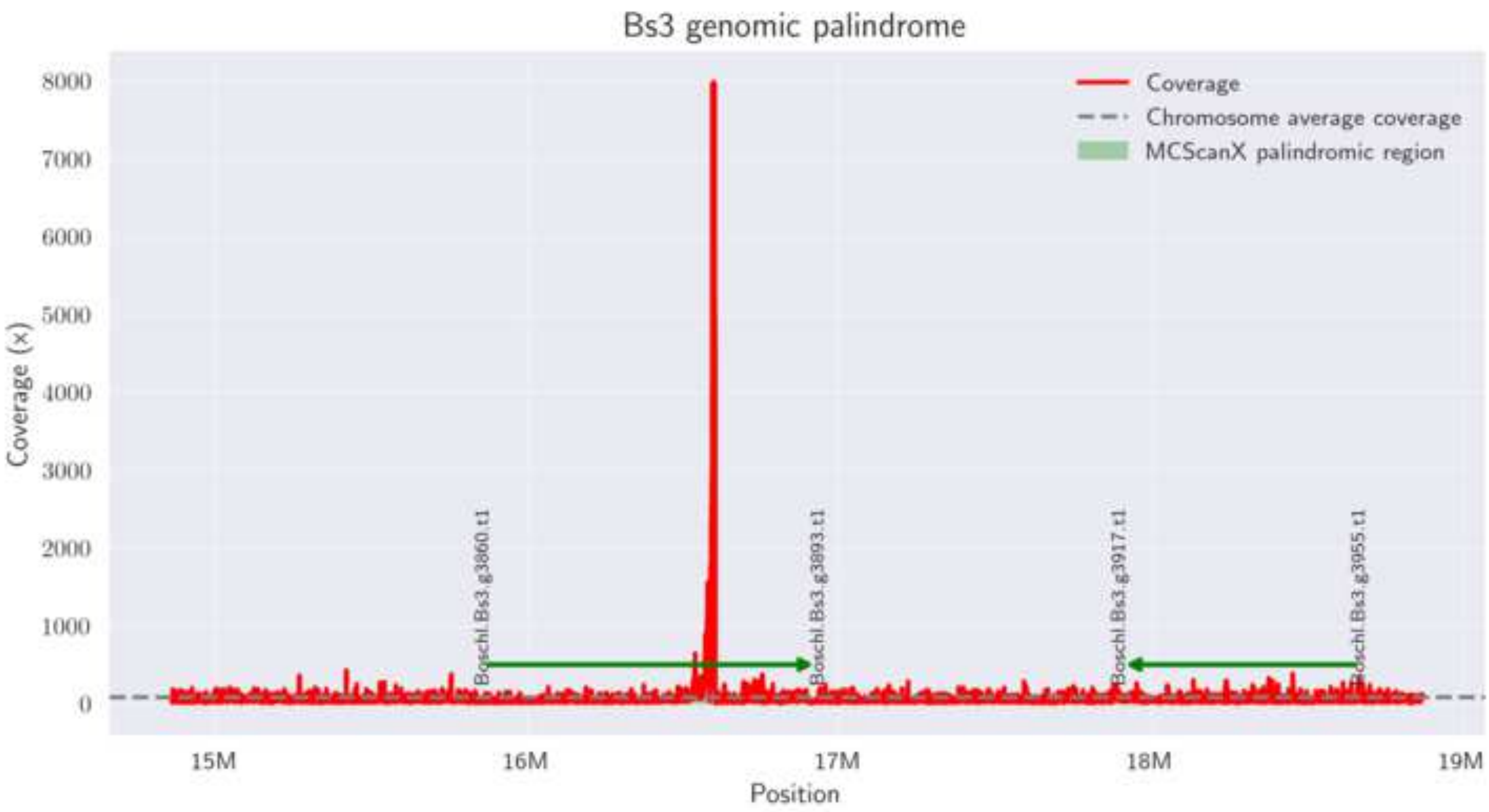

Supp. Figure 13a

Click here to access/download;LaTeX - Figure (eps, ps, etc.);quarTeT.Bs3.pdf

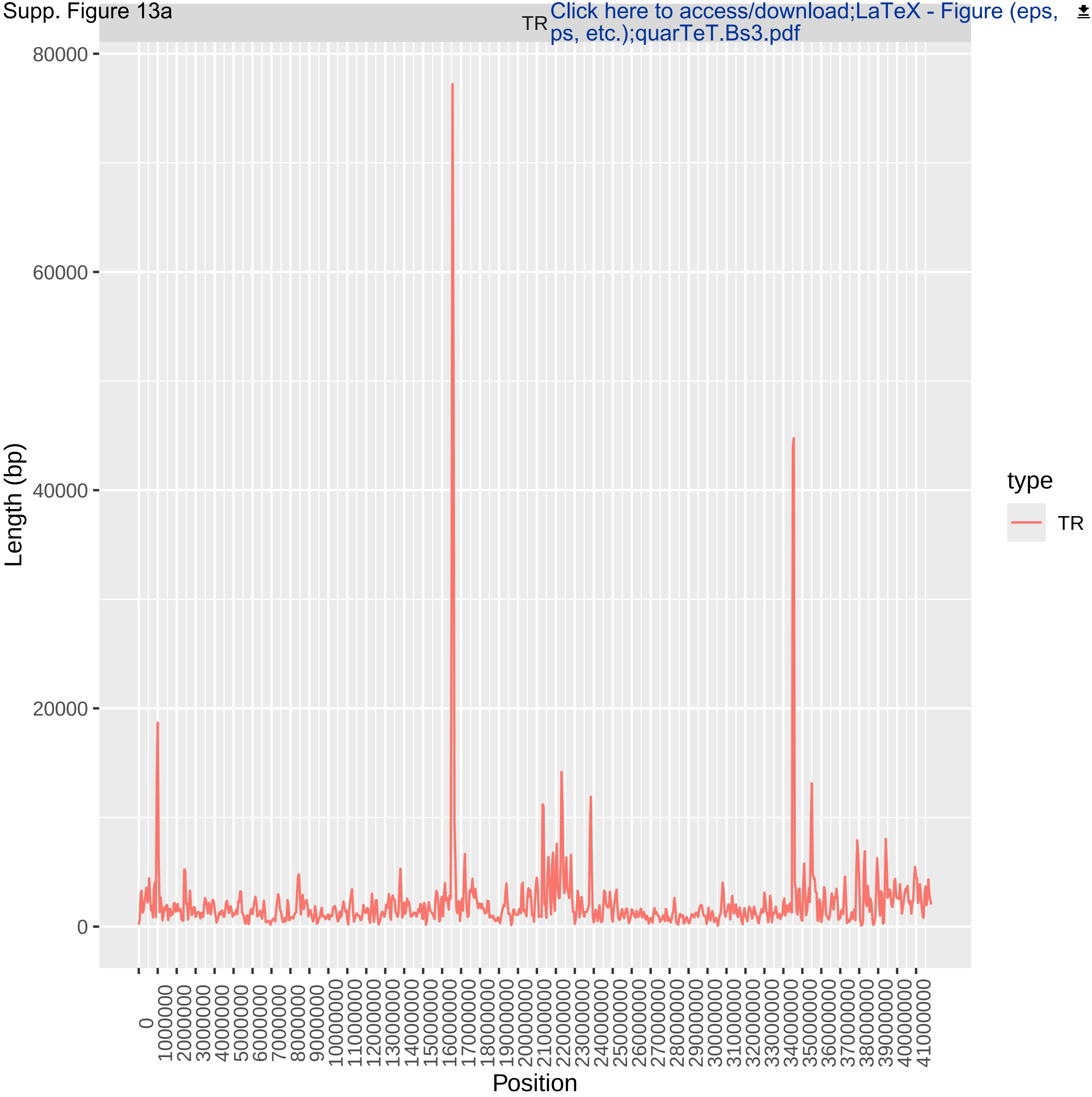

Supp. Figure 13b

Click here to access/download;LaTeX - Figure (eps, ps, etc.);Bs3_TAD.png

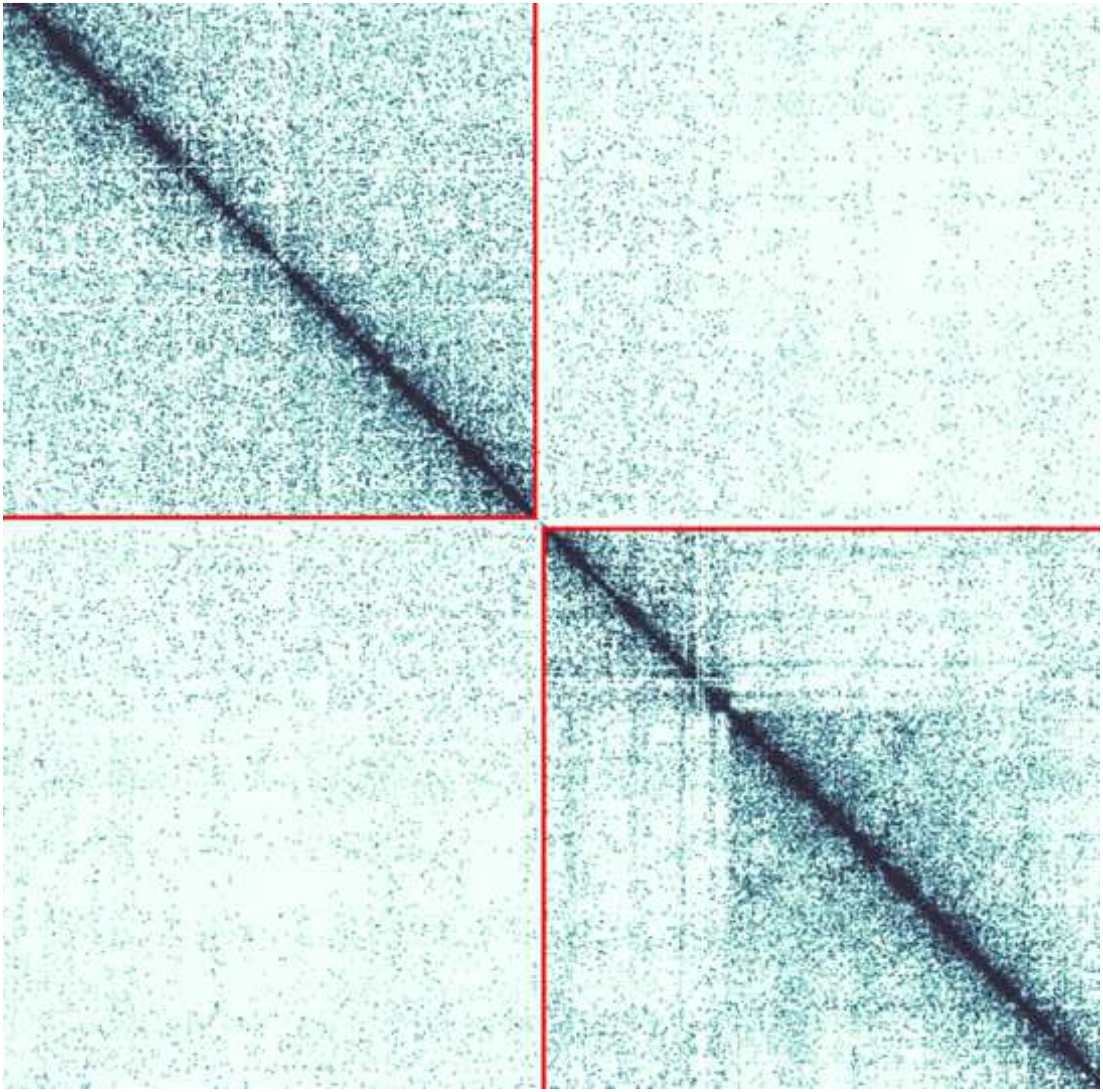

Supp. Figure 14

Click here to access/download;LaTeX - Figure (eps, ps, etc.);asm_all_karyotype.pdf

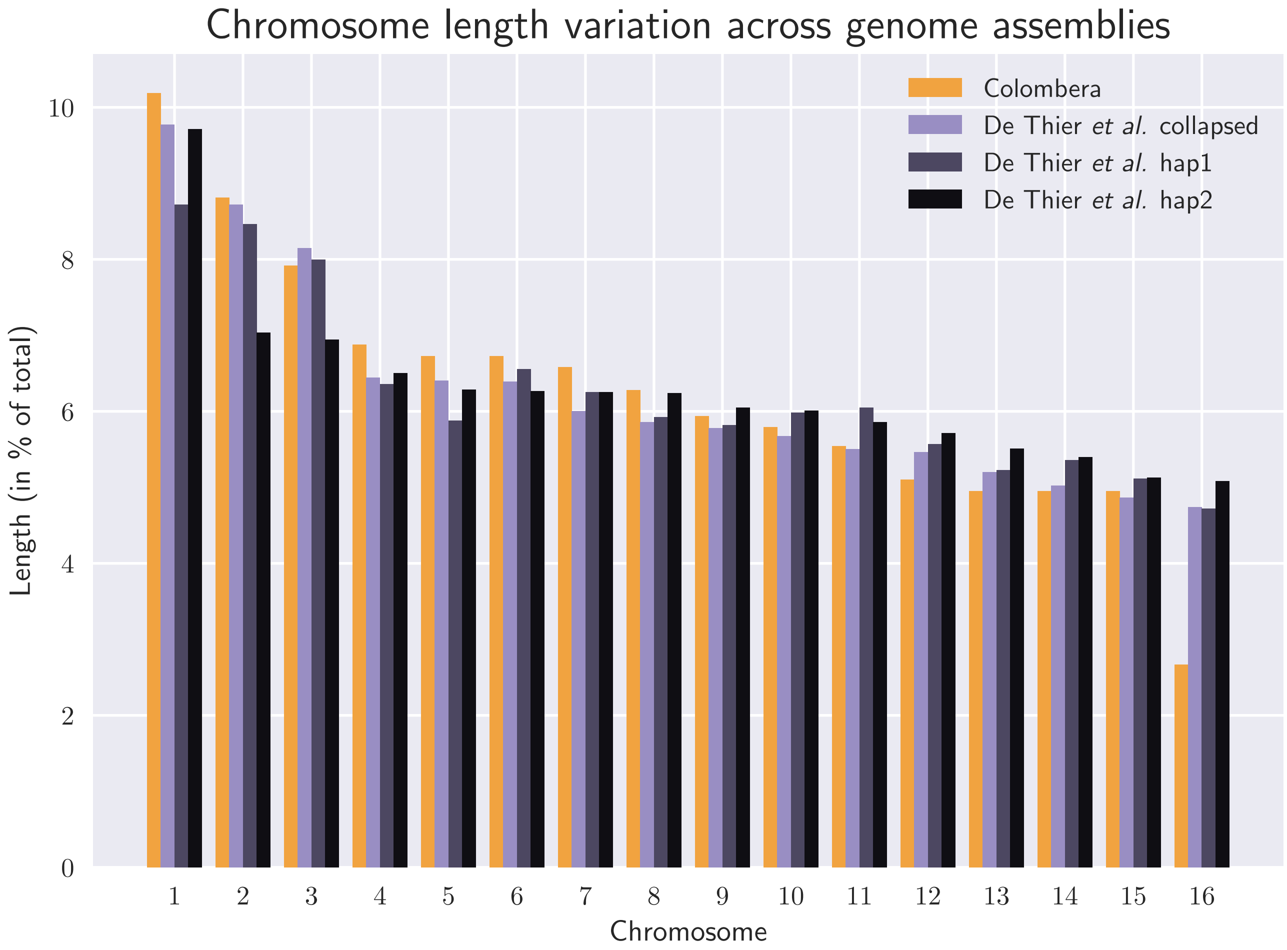

Supp. Figure 15a

Click here to access/download;LaTeX - Figure (eps, ps, etc.);hap1_vs_haploid.png

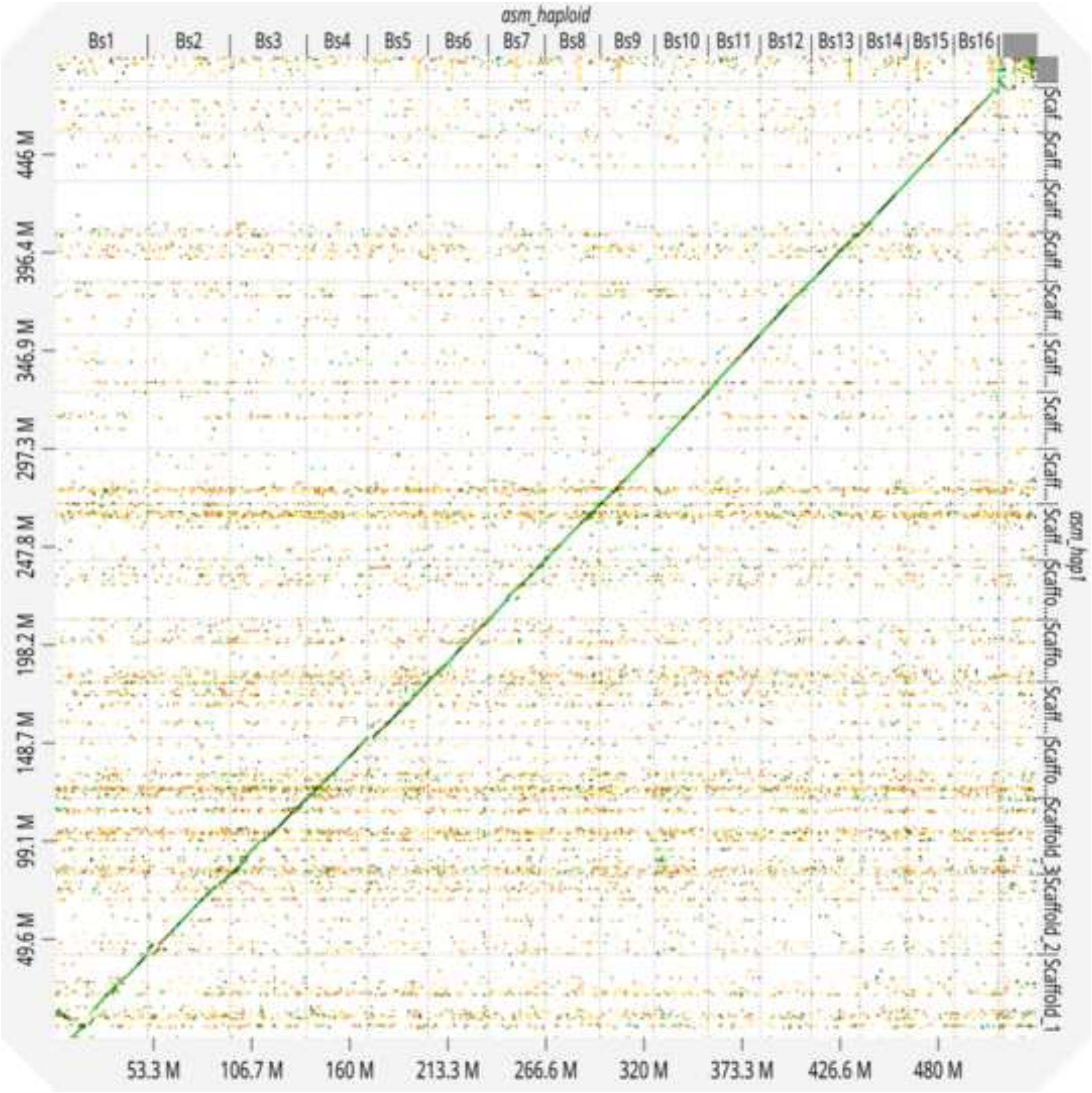

Supp. Figure 15b

Click here to access/download;LaTeX - Figure (eps, ps, etc.);hap2_vs_haploid.png

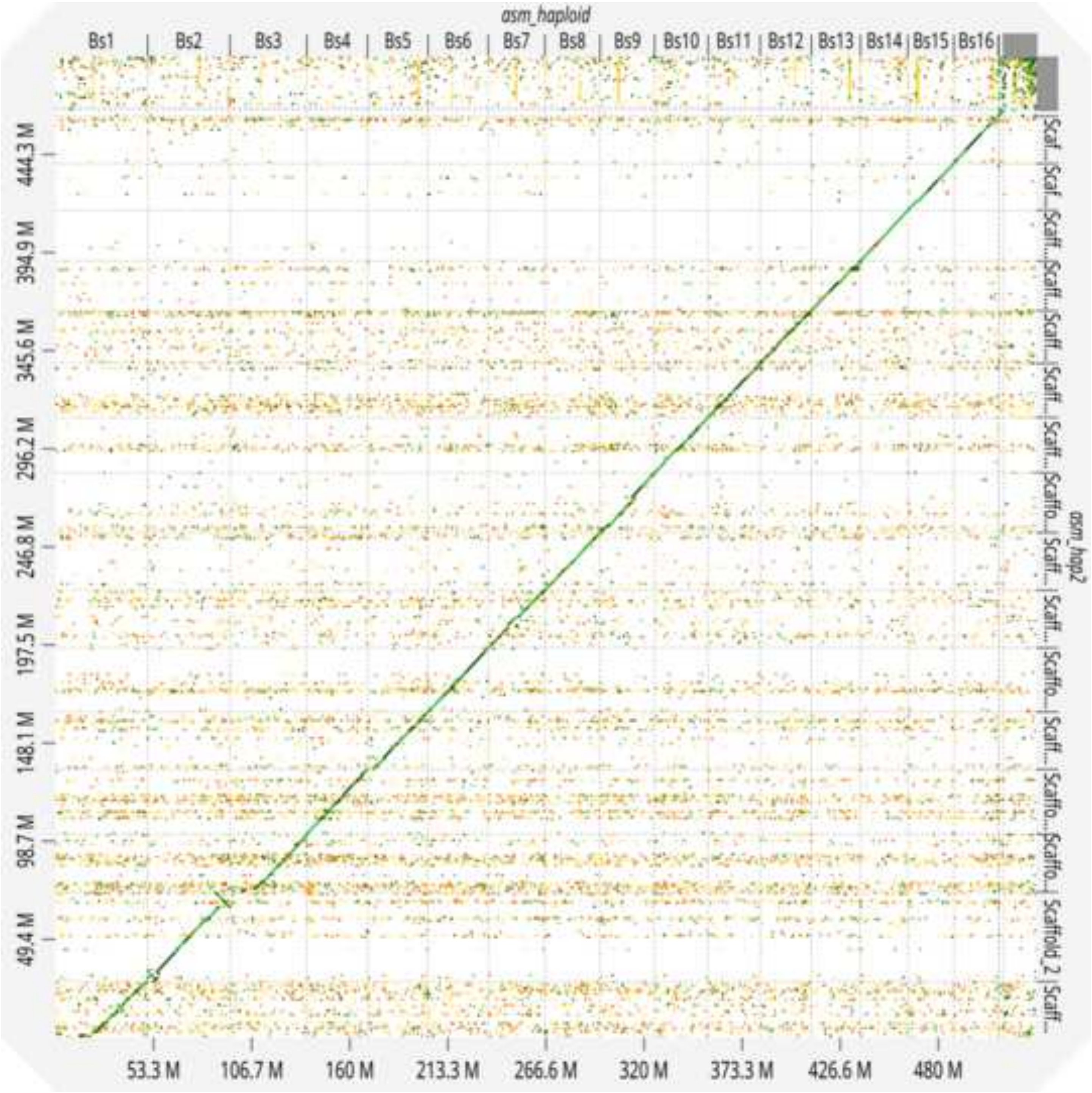

Supp. Figure 15c

Click here to access/download;LaTeX - Figure (eps, ps, etc.);map_asm_hap2_to_asm_hap1.png

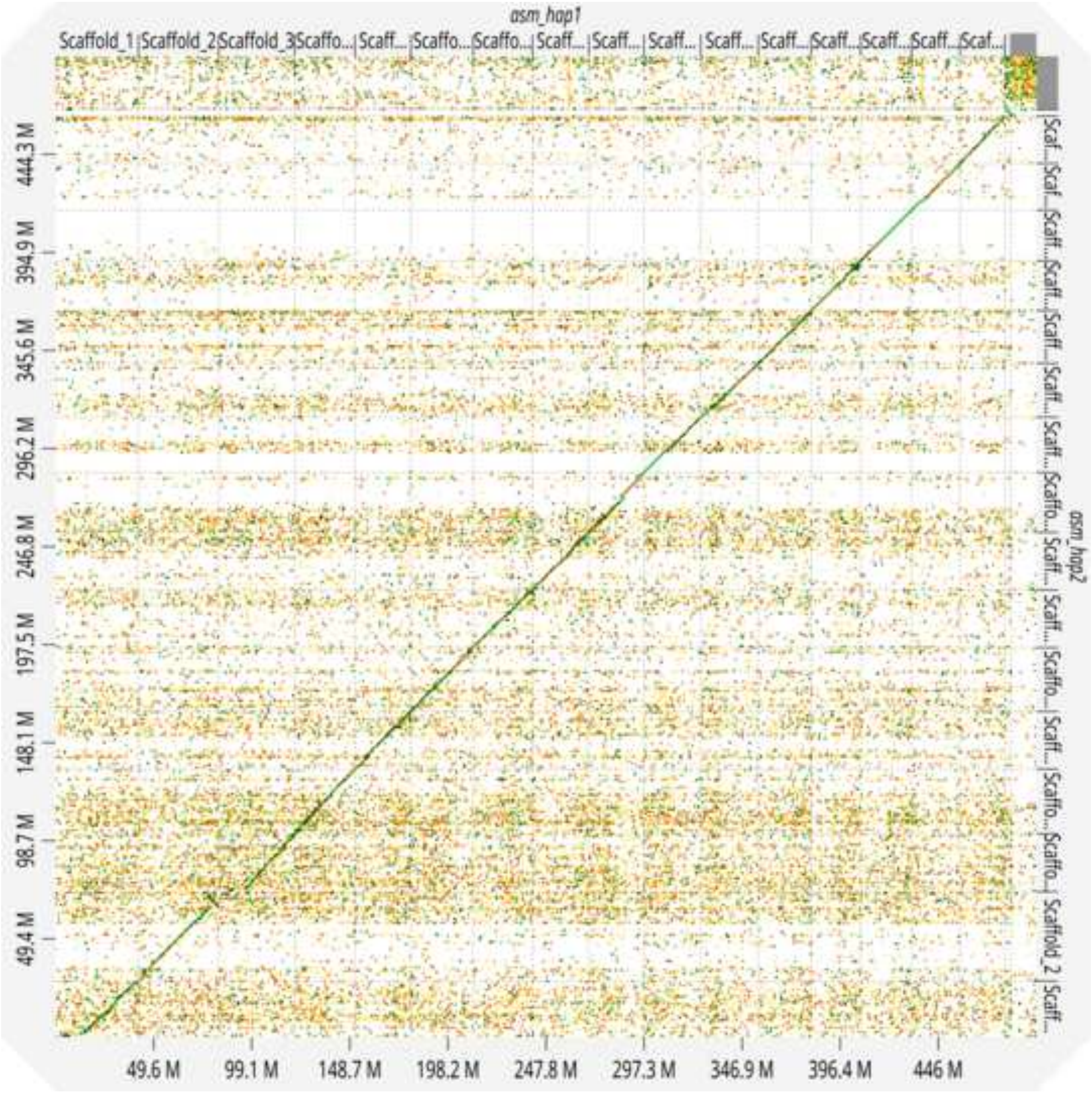

Supp. Figure 16

Click here to access/download;LaTeX - Figure (eps, ps,

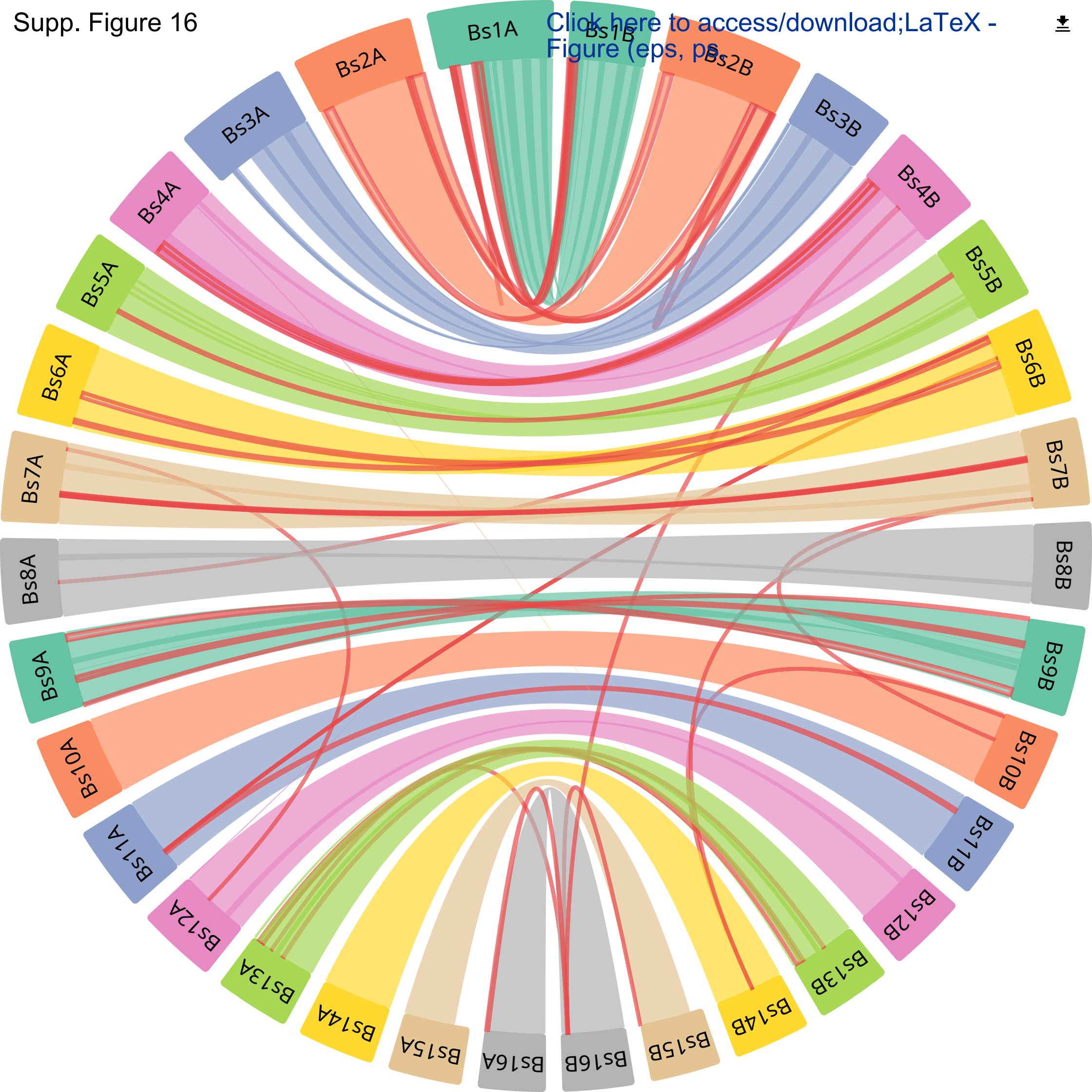

Click here to access/download;LaTeX - Figure (eps, ps, etc.);dotplots_bs_sc_cr_od_V2.pdf

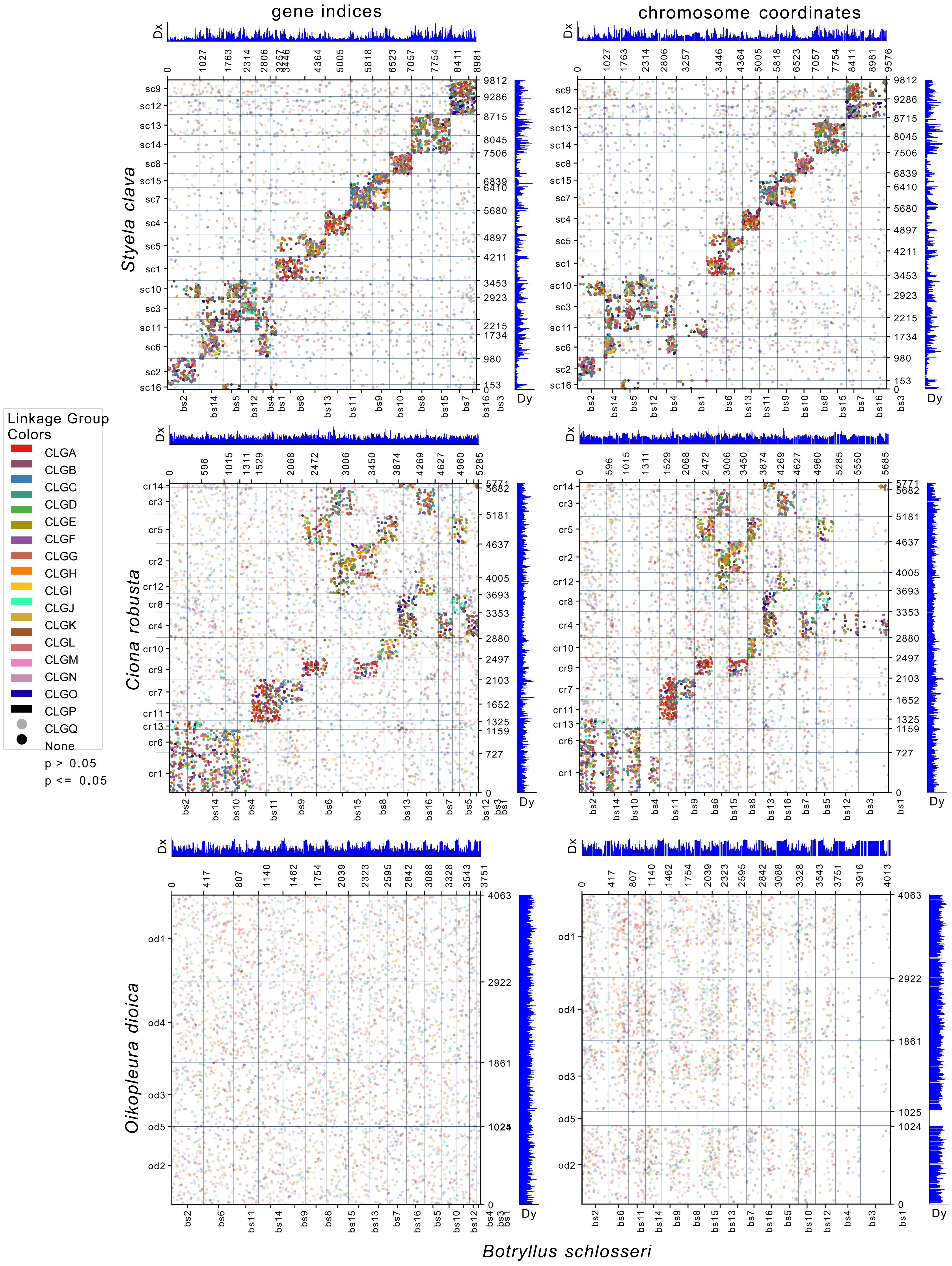

Supp. Figure 18

Click here to access/download;LaTeX - Figure (eps, ps, etc.);odp_BCnS_ribbon.pdf

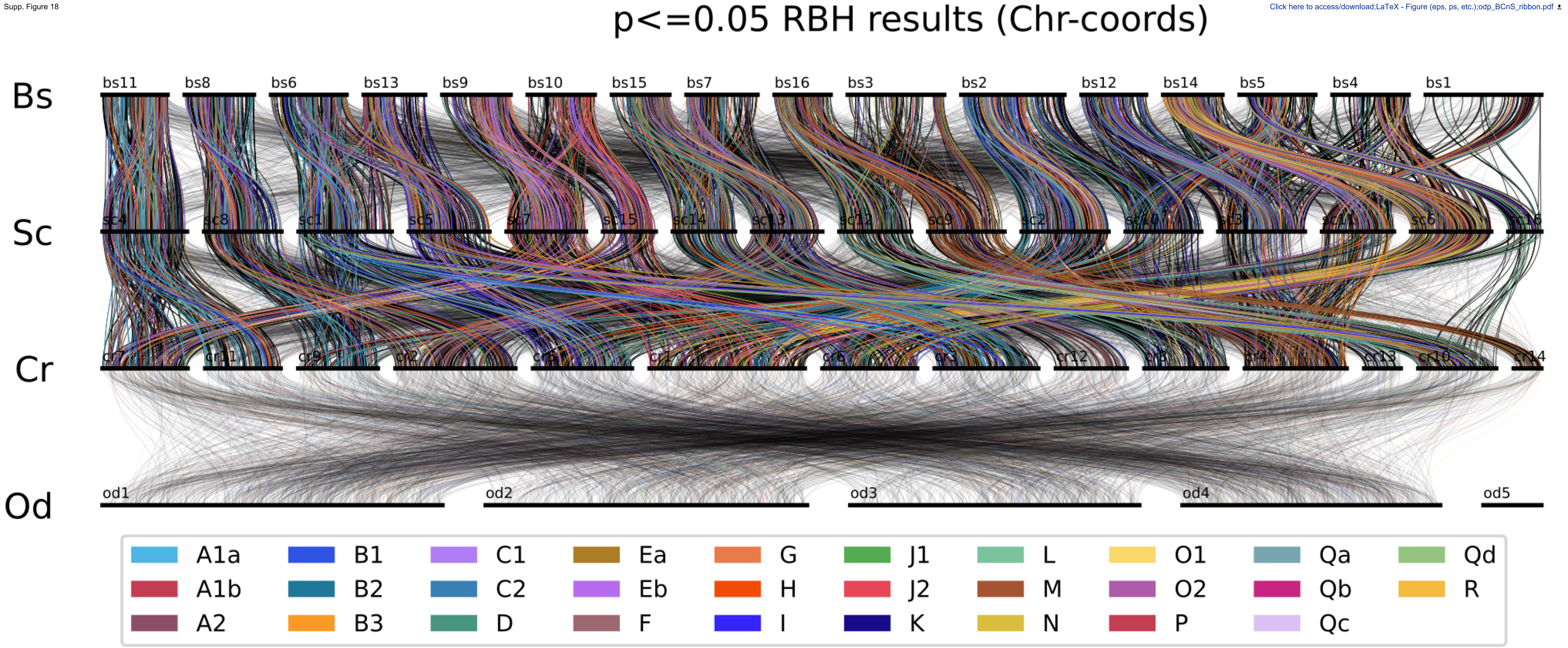

Supp. Figure 19

Click here to access/download;LaTeX - Figure (eps, ps, etc.);Figure_S14_bootstraps2.pdf

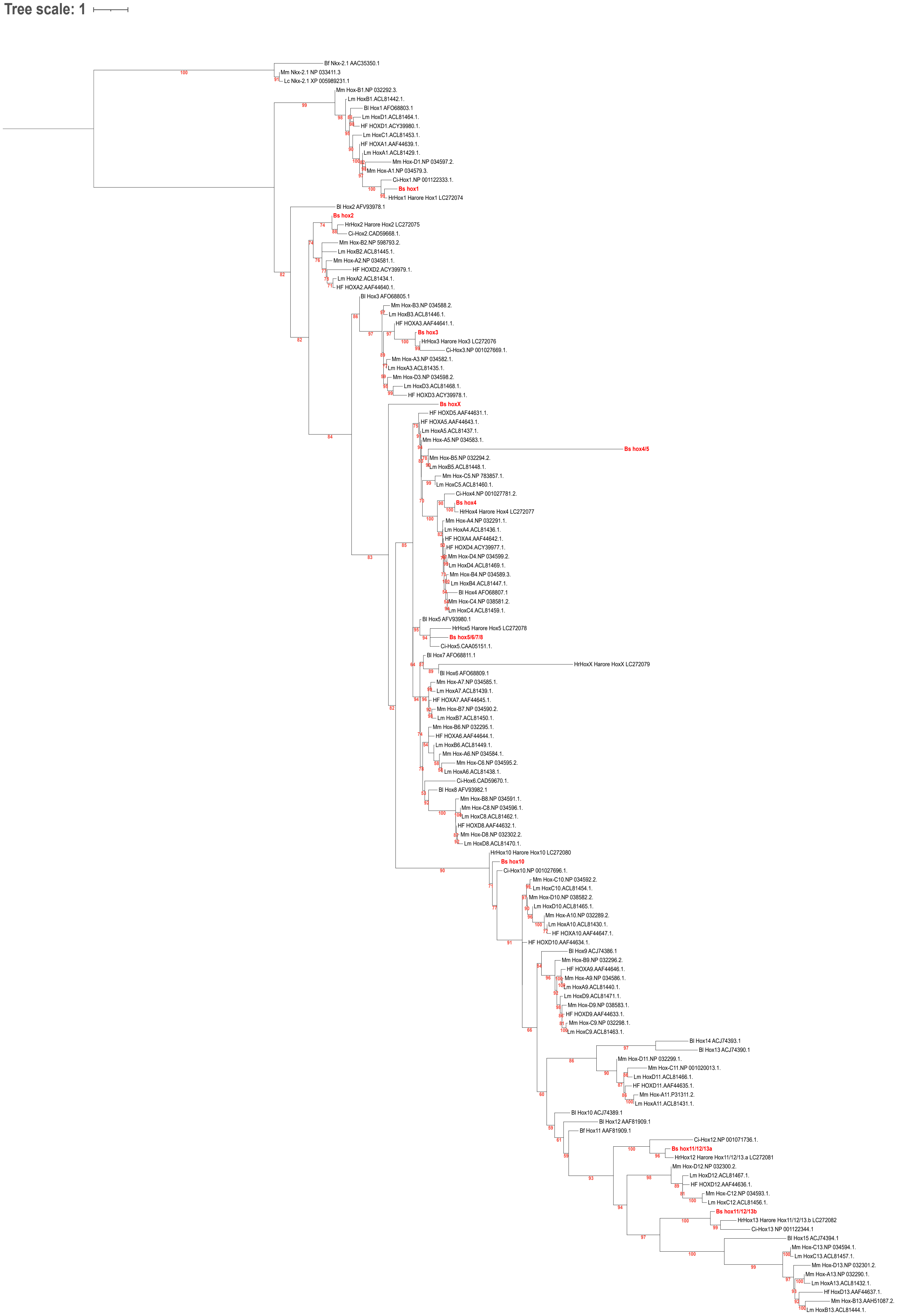

Click here to access/download;LaTeX - Figure (eps, ps, etc.);Bayesian_tree.pdf

Supp. Figure 20

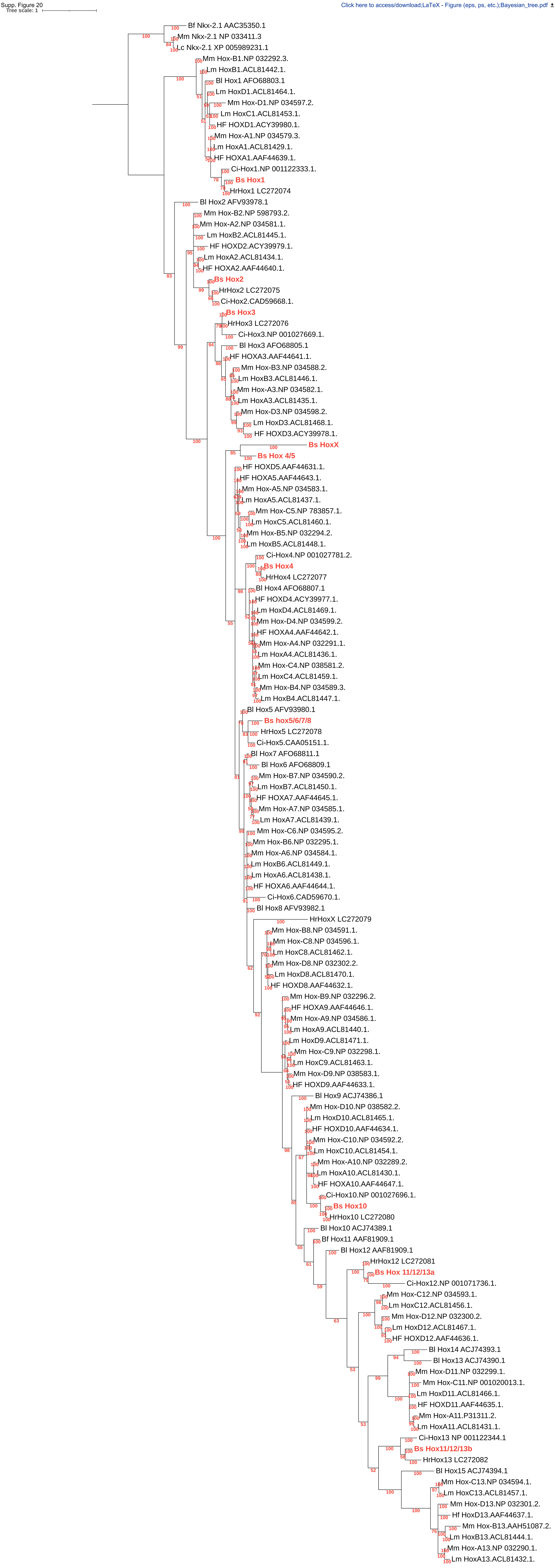

Manuscript revised (pdf)
Click here to access/download;Manuscript;De_Thier_et_al_22_7_2025.pdf

GigaScience, 2025, 1–27

doi: xx.xxxx/xxxx
Manuscript in Preparation
Paper

PAPER

First chromosome-level genome assembly of the colonial chordate model *Botryllus schlosseri* (Tunicata)

Olivier De Thier [1,2], Marie Lebel [3], Mohammed M.Tawfeeq [1,2], Roland Faure [1,2], Philippe Dru [3], Simon Blanchoud [4], Alexandre Alié [3], Federico D. Brown [5], Jean-François Flot [1,2*] and Stefano Tiozzo [3*]

[1]Evolutionary Biology & Ecology, C.P. 160/12, Université libre de Bruxelles (ULB), Avenue F.D. Roosevelt 50, B-1050 Brussels, Belgium and [2]Interuniversity Institute of Bioinformatics in Brussels – (IB)^2, Brussels, Belgium and [3]CNRS, Laboratoire de Biologie du Développement de Villefranche Sur-mer (LBDV), Sorbonne Université, Paris, France and [4]Department of Biology, University of Fribourg, Fribourg, Switzerland and [5]Departmento de Zoologia, Instituto de Biociências, Universidade de São Paulo, São Paulo - SP, Brazil

*jean-francois.flot@ulb.be; stefano.tiozzo@imev-mer.fr

Abstract

Background: *Botryllus schlosseri* (Tunicata) is a colonial, laboratory model tunicate recognized for its remarkable developmental diversity, its regenerative abilities, and its peculiar genetically determined allorecognition system governed by a polymorphic locus controlling chimerism and cell parasitism. **Results**: We report the first chromosome-level genome assembly of *B. schlosseri* sub-clade A1. By integrating long and short reads with Hi-C scaffolding, we produced both a phased diploid genome assembly and a conventional collapsed consensus sequence of 533 Mb. Of this total length, 96% belonged to 16 chromosome-scale scaffolds, with a BUSCO completeness score of 91.4%. We then compared our assembly with other high-quality tunicate genomes, revealing some synteny conservation but also extensive genomic rearrangements and a general loss of colinearity. **Conclusions**: The chromosome-level resolution of this assembly enhances our understanding of genome organization in colonial modular organisms. Comparative analyses highlight the dynamic nature of tunicate genomes, with conserved macrosynteny yet extensive microsyntenic rearrangements and scrambling, underscoring their rapid evolutionary trajectory. This high-quality genome assembly provides a valuable resource for exploring the unique biological features of colonial chordates, including their exceptional regenerative abilities and complex allorecognition system.

Key words: budding; regeneration; chimerism; ascidian; coloniality; model organism

Introduction

Each member of the colony is an individual animal, but the colony is another individual animal, not like the sum of its individuals [...]. So a man of individualistic reason, if he must ask, "Which is the animal?" must abandon his particular kind of reason and say, "Why, it's two animals and they aren't alike any more than the cells of my body are like me. I am much more than the sum of my cells, and, for all I know, they are much more than the division of me."

—John Steinbeck *The Log from the Sea of Cortez*

In the sub-phylum Tunicata, the sister group of vertebrates [1], colonial species reproduce both sexually and asexually through various forms of budding. Through budding, new functional bodies emerge from adult somatic cells and tissues. Regardless of variations in budding modes among tunicate species [2] and of whether development occurs through asexual budding or sexually via embryogenesis, the basic body plan of adult tunicates is broadly conserved across the entire sub-phylum [3]. In colonial tunicates, asexually generated individuals generally remain physically connected, forming colonies. Colony formation, clonal reproduction,

Compiled on: July 22, 2025.
Draft manuscript prepared by the author.

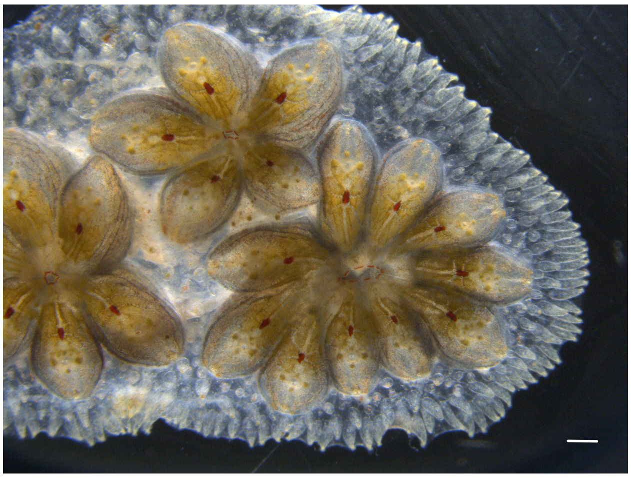

Figure 1. Colony of *Botryllus schlosseri*. Scale bar 1mm.

and modular organization have important physiological, ecological, and evolutionary implications. For example, modular organization supports rapid growth on hard, space-limited substrates, outperforming solitary forms. Morphological plasticity enables colony-level adaptation to predation, damage, or environmental changes. Furthermore, uniparental reproduction, including budding, likely provides a selective advantage for rapid colonization on invasion fronts or in disturbed habitats (reviewed in [4]). Like many other colonial tunicates, *Botryllus schlosseri* (Pallas, 1766) (NCBI:txid30301) can generate a functional adult body via three distinct developmental pathways. The first one involves sexual reproduction, where the fertilized egg passes through a larval stage and develops into an initial colony founder. The second pathway is asexual propagation, where the founder zooid continuously reproduces through palleal (aka peribranchial) budding, forming a colony of hundreds of zooids connected by the vascular system (a network of extra-corporeal vessels within a cellulose-based extracellular matrix, the so-called tunic [5]; Figure 1). Lastly, if all zooids and buds are removed from a *B. schlosseri* colony, new buds can regenerate from the vascular system in a process known as vascular budding, allowing asexual propagation and eventual colony reformation [6, 7, 8]. Zooids within a single colony are genetically identical clones. However, wild colonies often come into contact and fuse, resulting in chimeras where circulating cells carry different genotypes. These mixed pools of circulating cells contribute to sexual and, according to some authors, to asexual and regenerative development [9, 10, 11]. During chimerism, donor cells may entirely replace the host's germline or somatic cells, a phenomenon termed germ cell or somatic cell parasitism, respectively [12, 13, 10]. As a result, zooids within a chimeric colony are not always clonemates.

Botryllus schlosseri was introduced to laboratories over half a century ago [14] as a model to study asexual development, regeneration [15], allorecognition and chimerism [16, 17]. Over recent decades, a dedicated scientific community has emerged, advancing breeding techniques and developing imaging and molecular biology tools to better study this species [18, 19, 20, 9, 21, 8]. Several anatomical descriptions and staging methods have been proposed [22, 5] and extensive transcriptomic databases for various developmental stages and tissues have been generated [23, 24, 25, 26, 27, 8]. In 2013, a draft genome of *B. schlosseri* was published [28] but it lacked the completeness and continuity required by today's assembly standards [29]. In this study, we present a high-quality, chromosome-level collapsed assembly as well as a chromosome-scale haplotype-resolved assembly for *B. schlosseri*. This new resource offers a robust platform for investigating the developmental and regenerative processes, complex allorecognition, chimerism, and cell parasitism of this colonial chordate.

Results and Discussion

Sequencing and genome size estimation

Genomic DNA was extracted from a laboratory-reared colony, referred to as clone E*, derived from a single zygote and therefore non-chimeric. Sequencing libraries from clone E* yielded 489 million Illumina (short) paired-end 150 bp reads, 2.4 million PacBio HiFi (long) reads with a N50 length of ~9.5 kb (max length of ~50 kb) and 10.9 million ONT (long) reads with a N50 length of ~10.3 kb (max length of ~205 kb) (Table 1).

Technology	Tot. size (Gbp)	Number of reads	N50 (bp)	Coverage
Illumina	73.2	488,906,094	150	146
Illumina Hi-C	15.9	106,488,252	150	32
PacBio HiFi (round 1)	7.9	1,170,137	8,711	16
PacBio HiFi (round 2)	10.8	1,218,052	10,151	22
ONT (R9.4.1)	58.9	10,888,103	10,320	118

Table 1. Sequencing technologies applied to sequence *B. schlosseri*'s genome (clone E*) and related read statistics.

Based on k-mer analyses, the genome size was estimated to be around 500 Mbp with a heterozygosity of 3.63% (Figure S1), whereas Feulgen densitometry (a histochemical approach) yielded an estimate of ~492 Mbp (using 1 pg = 978 Mbp; Figure S5). Both genome size estimates were concordant but notably smaller than a previous cytofluorimetry-based estimation of 725 Mb [30] and than the first genome assembly obtained by Voskoboynik *et al.* [28], which had a size of 580 Mbp.

An initial collapsed genome assembly was obtained using hifiasm [31] (RRID:SCR_021069); it had a size of 570 Mbp and comprised 930 contigs with an N50 length of 4.9 Mbp. In this assembly, BlobToolKit (RRID:SCR_023351) identified 452 contigs (totaling 37 Mbp) as putative contamination and mitochondrial sequences (see next section), which were subsequently removed. Of these 37 Mbp, approximately half were attributed to members of the bacterial phylum Pseudomonadota (Figure S6). We identified 28 contigs that belonged to spore-forming unicellular parasites of the microsporidia group [32]. To our knowledge, this represents the first report of this fungal group in a tunicate species. However, we cannot rule out the possibility that these sequences may have been assigned incorrectly or originate from contaminants present in the water rather than from parasitized *Botryllus* tissues. The remaining contigs were corrected using CRAQ [33], which detects and breaks misassembled contigs; this raised the total number of contigs in the assembly from 478 to 516. We then performed Hi-C scaffolding using YaHS [34] (RRID:SCR_022965), which reduced the number of sequences to 256, before running CRAQ again on the scaffolded assembly: this time, 4 misassembled contigs were detected and broken. Finally, a manual curation was performed, resulting in an assembly made up of 16 major scaffolds, labelled Bs1 to Bs16, containing around 96% (513 Mbp) of the total sequence length (533 Mbp) (Tables 2 and S3, Figures 2 and 3). The number and relative lengths of these 16 major scaffolds were consistence with the published karyogram of *B. schlosseri* [35], with the exception of Bs16 that was notably longer in our assembly (Figure S14). The full assembly pipeline is summarized in Figure 4 and detailed in the Material and Methods section.

The completeness of our assembly was assessed using the Benchmarking Universal Single-Copy Orthologs tool [39] (BUSCO, RRID:SCR_015008, v5.4.4) with the metazoa_odb10 dataset, which returned a genome completeness of 91.6% (including 0.9% of duplicated marker genes), compared to 74.4% (including 23.7% of

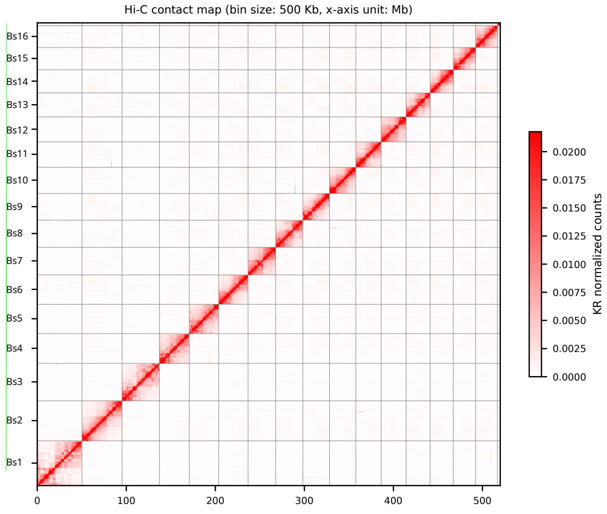

Figure 2. Hi-C heatmap of the unphased assembly of the *Botryllus schlosseri*'s genome showing sixteen chromosome-scale scaffolds. The figure was generated using the visualization module of HapHiC [36].

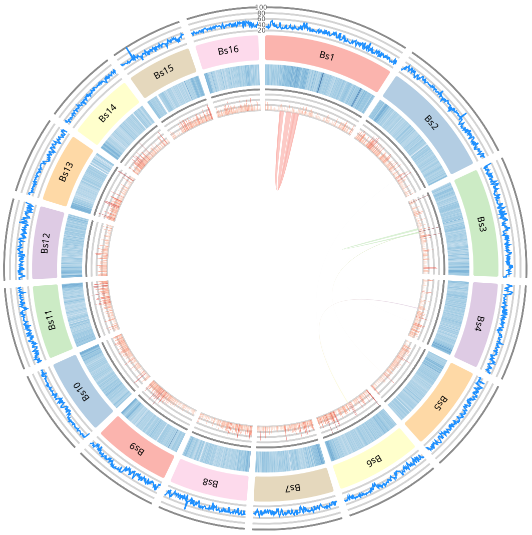

Figure 3. Circos plot of the distribution of several genomic characteristics along the 16 longest scaffolds (labelled Bs1 to Bs16) of the collapsed assembly (made using AccuSyn [37]). Each layer of the circle represents, from the inside to the outside: the synteny blocks detected by MCScanX [38]; histograms of gene density; heatmaps of the presence of repetitive elements; the scaffold names in clockwise order; and the sequencing depth of HiFi reads.

Measure	All scaffolds	16 longest scaffolds
Length (Mbp)	533	513
No. of sequences	254	16
N50 (Mbp)	30	31
GC (%)	40.52	40.46
No. of annotated genes	22,275	21,677
BUSCO Complete (Single, Duplicated)	91.6% (90.7%, 0.9%)	91.4% (90.7%, 0.7%)
BUSCO Fragmented	3.1%	3.1%
BUSCO Missing	5.3%	5.5%

Table 2. Assembly statistics for all the scaffolds and for the 16 longest ones.

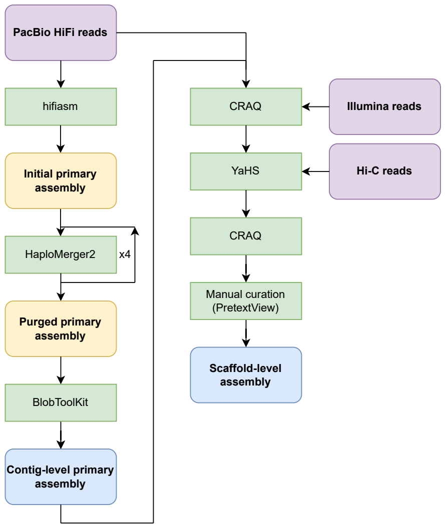

Figure 4. Assembly pipeline for the unphased genome assembly (see Material and Methods.)

duplicated marker genes) for the assembly of Voskoboynik *et al.* (Figure 5). The high duplication score of the previously available assembly indicates that its larger size (580 Mbp vs. 533 Mbp) was caused by incompletely collapsed haplotypes [40]. Synteny analysis performed using MCScanX [38] (RRID:SCR_022067) highlighted the presence of two large-scale genomic palindromes located within Bs1 and a smaller one in Bs3 (displayed in red and green in the innermost layer of Figure 3). To find out whether these palindromes may have resulted from assembly artifacts caused by uncollapsed haplotypes [41], we checked the sequencing depth profiles across these regions (Figures S11, S12, S13) as well as the localization of the duplicated BUSCO genes along the chromosomes, and did another run of CRAQ this time using ONT as long reads (with higher coverage compared with the HiFi reads used in the previous rounds). There was no significant difference in the number of duplicated BUSCO genes within Bs1 and Bs3 compared to other genomic regions, and CRAQ did not detect structural errors in these scaffolds either. This suggests that the palindromes observed are real, with potential biological significance that will require further investigation.

Molecular identification as sub-clade A1

B. schlosseri is considered a species complex comprising five genetically distinct clades (A to E), each representing a cryptic species with its own characteristic geographic distribution [42, 43]. Detailed analysis of COI mitochondrial sequences divides Clade A into three distinct sub-clades: A1, A2, and A3 [44]. The complete mitochondrial DNA of clone E* was recovered and assembled as a single

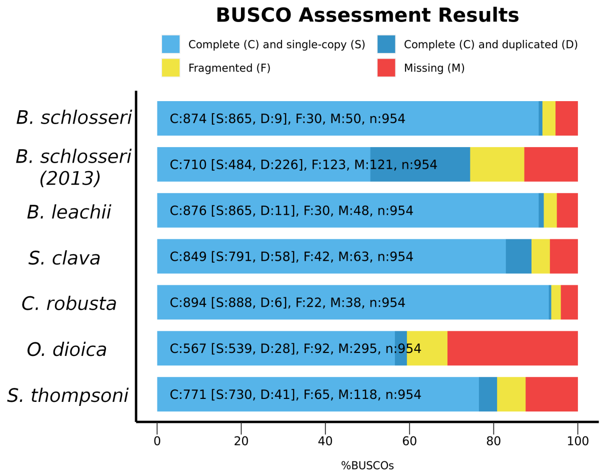

Figure 5. Orthology assignment in previous tunicate genome projects. Proportion of BUSCO genes detected or missed in the new genome assembly of *B. schlosseri* compared to the previous assembly (*B. schlosseri* (2013) [28]) and other reference genomes.

Repeat class	Percent of genome
**LINEs**	**4.52%**
LINE1	0.15%
LINE2	2.06%
**LTR elements**	**1.34%**
**DNA elements**	**7.24%**
hAT-Charlie	2.96%
TcMar-Tigger	0.01%
**Unclassified**	**46.03%**
**Total interspersed repeats**	**59.12%**
**Simple repeats**	**3.94%**
**Low complexity**	**0.02%**
**Total**	**63.09%**

Table 3. Classes of repeats in the *Botryllus schlosseri* genome. RepeatMasker summary table for the collapsed genome assembly of *Botryllus schlosseri* showing the percentages of identified repeat classes

Type	Number	Mean size (bp)	% genome
Gene	22275	8566.13	35.78
mRNA	30813	10576.62	N/A
cds	237200	199.16	8.86
Exon	241815	289.83	13.14
5' UTR	21386	432.29	1.73
3' UTR	20985	648.00	2.55
Total	574474	1143.44	N/A

Table 4. Gene predictions and annotation statistics.

circular contig. Our mitogenome assembly shares 99.95% identity with the published mitochondrial sequence assigned to the *B. schlosseri* sub-clade A1 [44]. Notably, this sub-clade includes the sc6ab specimen used by Voskoboynik *et al.* [28] to generate the previous reference assembly of *B. schlosseri*. Our mitogenome assembly further shares 99.7% nucleotide identity with that reference sequence. Phylogenetic analyses based on a COI fragment used as DNA barcode for ascidians ([44]) confirmed that sample E* belongs to sub-clade A1 (Figure S7), a group that is both widely distributed and employed as a laboratory model worldwide.

Structural and functional annotation

Using a *de novo* repeat library created by RepeatModeler (RRID:SCR_015027), RepeatMasker (RRID:SCR_012954) detected that around 63% of the novel *B. schlosseri* collapsed genome assembly consists of repetitive elements, which is close to the 65% of repeats found in the previously published assembly [28]. Most of these were interspersed repeats (see Table 3). A relatively high abundance of repetitive sequence was also reported in other colonial tunicates. For instance, *Salpa thompsoni* and *Salpa aspera*, both colonial species, possess a larger genome (742 Mb and 901 Mb, respectively) and an higher repeat content (ca. 80%) compared to solitary tunicates such as *Ciona robusta* (ca. 160 Mb, about 20–25% repeats) or *Oikopleura dioica*, which has a compact genome of 70 Mb with only ca. 15% repetitive content. This pattern suggests that colonial tunicates exhibit a greater genomic expansion and a larger repeat content than their solitary counterparts. Yet, the colonial *Botrylloides diegensis*, which carries a relatively small genome[45], and the solitary *S. clava* with 46,6% repetitive elements, represent notable exceptions. Additional high-quality genome assemblies across a broader range of tunicate species will be essential to confidently assess the possible association between coloniality and repeat content [46, 47, 48].

Ab initio genome annotation using the BRAKER3 pipeline [49] (RRID:SCR_018964) initially predicted 16,966 coding genes, after which refinement using the PASA pipeline [50, 51] (RRID:SCR_014656) finally retrieved 22,275 genes coding for 30,813 proteins (see Table 4). This number is significantly lower than originally predicted for *B. schlosseri* (38,730 predicted genes [28]), probably due to the incomplete collapse of the previous assembly. In terms of completeness of the annotation, BUSCO retrieved 92.4% complete (79.7% single, 12.7% duplicated) and 1.8% fragmented metazoan genes when given all predicted isoforms, whereas it retrieved 92% complete (91% single, 0.9% duplicated) and 1.8% fragmented metazoan marker genes when filtered to only keep the longest isoform. Running BUSCO directly on the scaffold sequences yielded similar results (data not shown).

The functional annotation and orthology assignment [52], coupled with annotation of protein domains, motifs, and functional sites [53, 54], were written into gff3 and Genbank files. KEGG route-mapping assigned 7,221 genes over the annotated entries and distributed them across 21 KEGG categories (Figure 6). Among them, the most prevalent ones include KEGG hierarchies dealing with genetic information processing (2449/7219, 22,92%), such as DNA replication, repair, recombination, transcription, translation and regulation of gene expression; signaling and cellular processes (886/7219, 12,27%); and environmental information processing (674/7219, 8,64%) such as various cellular processes and signaling pathways involved in sensing, transducing (i.e. MAPK signaling, PI3K-Akt signaling and cAMP signaling), responses to external signals (i.e. G-protein coupled receptors, receptor tyrosine kinases, and cytokine receptors), intracellular communication and cell motility. The KEGG annotations provided for *B. schlosseri* are consistent and coherent with the functional annotation of the published complete genomes of other ascidian tunicates such as *Styela clava*, *Ciona robusta* and *Oikopleura dioica* (Figure S8).

Haplotype-resolved assembly

Given its heterozygosity level exceeding 3%, haplotype-resolved assemblies of *B. schlosseri* are crucial for studying differences between homologous chromosomes, such as structural variations. Using hifiasm with direct integration of Hi-C reads and subsequent scaffolding (Figure S9), we generated a pair of chromosome-scale, haplotype-resolved assemblies (haplotype 1 and haplotype 2), each organized into 16 major scaffolds (see Figure S10). With respective sizes of 496 Mbp and 494 Mbp, these assemblies are smaller than the collapsed assembly (533 Mbp). When considering only the 16 longest scaffolds, the sizes decrease to 480 Mbp for haplotype 1 and 464 Mbp for haplotype 2, compared to 513 Mbp for the collapsed assembly. Additionally, their BUSCO com-

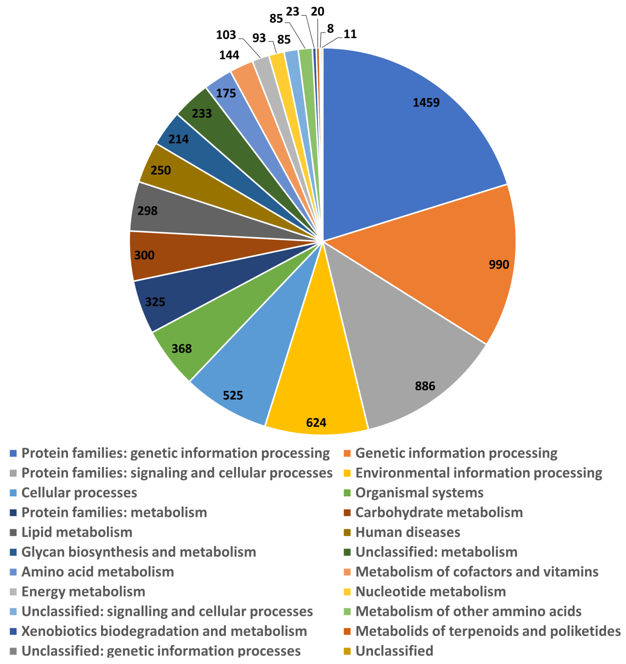

Figure 6. Pie chart of the assignation of the annotated genes of *Botryllus schlosseri* to KEGG functional categories using BlastKOALA [55].

pleteness scores are lower, with values of 90.9% and 91.2%, respectively, compared to 91.6% for the collapsed assembly. This is further reflected in their annotation results, where fewer genes were identified: 21,802 and 21,831 for haplotype 1 and haplotype 2, respectively, versus 22,275 for the collapsed assembly (see Table S1). The observed differences in metrics, where the results for the haplotype-resolved assemblies are inferior to those for the collapsed assembly, may be attributed to misassemblies, particularly deletions. For example, when comparing the putative chromosome lengths (see Table S2) for chromosomes 1 and 3, we observe a significant disparity in sizes between the two haplotypes, which may be attributed to incomplete sequence reconstructions during the assembly process. Such anomalies may additionally be observed when comparing the putative chromosome lengths of all assemblies with the karyogram of *B. schlosseri*, as described by Colombera [35] (see Figure S14). Notably, the sizes of the collapsed assembly appear to more closely match the expected distribution compared to the phased haplotypes. Furthermore, multiple structural variations between the two haplotypes, particularly small inversions (see Figures S15 and S16), seem to be present in the majority of the homologous chromosomes. However, as with the observed putative deletions, these may result from misassemblies and require further validation to enhance the quality of the haplotype-resolved assembly.

Synteny analyses

To assess macrosynteny conservation between *Botryllus schlosseri* and other tunicates, we selected genomes that met two specific criteria: they were assembled at the chromosome level, ensuring comparable high-quality structural information, and they represented, as much as possible, the breadth of diversity within the tunicate subphylum. *Styela clava* [56] belongs to the same order as *Botryllus* (Stolidobranchia), *Ciona robusta* [46] to a different order (Phlebobranchia), and *Oikopleura dioica* [47] to a different class of tunicates (Appendicularia) [57]. We used 17 groups of orthologous genes identified by Simakov *et al.* as ancestral chordate linkage groups (CLGs) [58]. These groups of genes are thought to have remained physically linked since the divergence of the Olfactores lineage (which includes both vertebrates and tunicates) from cephalochordates. However, Oxford dot plots [59] revealed a general loss of syntenic equivalence [60] among tunicate genomes, even between *B. schlosseri* and *S. clava*, which share the same haploid chromosome number of 16. Despite this identical number of chromosomes, the comparison between the two stolidobranchs showed extensive chromosome rearrangements, including fissions and fusions with mixing [61, 60] (Figures 7 and S17). These rearrangements are even more pronounced in *C. robusta*, which has a haploid chromosome number of 14. The overall random distribution of ortholog pairs within blocks points to significant order scrambling, resulting in a loss of colinearity (*i.e.* the sequential order of genes along the same chromosome); the comparison with *Oikopleura dioica* shows a complete breakdown of both macrosynteny and colinearity, with CLGs fully scrambled and dispersed. The latter result is consistent with the very long and fast-evolving branch of Appendicularia compared to other tunicates [57] as well as with the extreme genome scrambling rate of Appendicularia compared to other tunicates and mammals [62]. The same analyses using a set of 29 linkage groups generally conserved among bilaterians, cnidarians and sponges [60] yielded similar results (Figure S18). The extensive physical linkage of groups of orthologous genes has been shown to be conserved across highly divergent bilaterian phyla, including Chordata, Echinodermata, Mollusca, and Nemertea [60, 61]. Notably, our preliminary synteny analyses across four tunicate species reveal a highly dynamic genomic landscape, where syntenic equivalence, defined as one-to-one chromosomal correspondence regardless of gene order, is largely disrupted, even among species within the same family. Frequent chromosomal fission and fusion events further underscore the rapid evolutionary turnover of tunicate genomes. The increasing erosion of macrosynteny with phylogenetic distance suggests that patterns of conserved chromosomal linkage could serve as informative characters for phylogenetic inference. Interestingly, a similar pattern of genome rearrangements was recently reported in Bryozoa [61] and in clitellate annelids [63, 64, 65], pointing to a potential parallel and independent loss of the ancestral bilaterian genome architecture in these lineages and in tunicates. These observations raise compelling questions about the underlying mechanisms driving such rearrangements, which may reflect a relaxation of the selective constraints typically maintaining gene order in other metazoan groups [66] .

Hox gene analyses

Hox genes are a subset of homeobox genes that play important developmental roles in the specification of body segments along the anterior-posterior axis. Their arrangement into a syntenic cluster colinear with gene expression is conserved across Bilateria, with some exceptions [67]. In the new collapsed assembly, we retrieved ten *B. schlosseri* Hox genes, which is consistent with draft genomes of other ascidian tunicates [68]. Orthology of *B. schlosseri* Hox genes was assessed using phylogenetic analyses as in Sekigami *et al.* [69], based on Hox tree topology among the tunicates *Ciona robusta* and *Halocynthia roretzi*, the cephalochordate *Branchiostoma lanceolatum* and three vertebrate species. The names of the *B. schlosseri* Hox genes were assigned based on their proximity to the ones of *C. robusta* (Figures S19, S20). However, most branches had low bootstrap support, and therefore including more tunicates as well as vertebrate species will be necessary to resolve the complex evolution of the Hox gene cluster across tunicates [68]. Although Hox genes are colinear between cephalochordates and vertebrates, it is not the case for tunicates [70]. In the tunicate species studied thus far, Hox clusters exhibit divergences in terms of colinearity and synteny relative to the ancestral chordate cluster [68]. In contrast to previous data [28, 45], our new assembly revealed that *B. schlosseri*'s Hox genes are less scattered

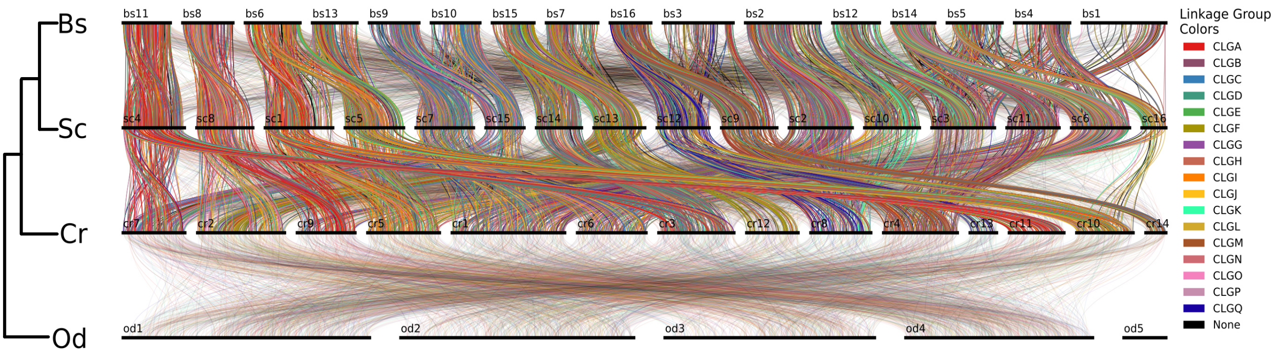

Figure 7. Synteny analyses using chordate linkage groups (CLGs) between *Botryllus schlosseri* (Bs), *Styela clava* (Sc), *Ciona robusta* (Cr) and *Oikopleura dioica* (Od). For each species, the horizontal black lines represent the chromosomes, while the colored vertical lines connect conserved orthologs between species pairs. Each color corresponds to one of the 17 ancestral CLGs identified in [58]. The opacity of the lines indicates the significance of the interaction between inter-species chromosomes, with solid colors representing significantly enriched conservation of synteny.

than previously described, suggesting improved contiguity in the new genome assembly. Eight of them are grouped on the second largest scaffold (Bs2) , yet for some of them at relatively large distance, whereas two other ones are found on the 15th largest scaffold (Bs15) (Figure 8). Comparison with two tunicate ascidians, belonging to the same (*H. roretzi* [69]) and a different (*C. robusta* [46]) order, revealed partially conserved synteny as well as inversions and transpositions across the three species (Figure 8). These observations agree with the general trend of synteny conservation despite loss of colinearity observed for CLGs [58] and are also consistent with the phylogenetic relationships among the species sequenced [2, 57]. Yet, the limited availability of chromosome-level genome assemblies continues to hinder a clear picture of the evolutionary dynamics of the Hox clusters across tunicates. Altogether, these findings show that *B. schlosseri* follows the general tunicate trend of dispersed and rearranged Hox clusters, but with a more clustered configuration than previously thought. This could reflect lineage-specific retention of partial clustering, and provides a more refined view of the dynamic genomic architecture in tunicates. While colinearity was clearly lost, partial synteny and clustering remain, offering a potential window into the mechanisms and consequences of Hox cluster disintegration during chordate evolution.

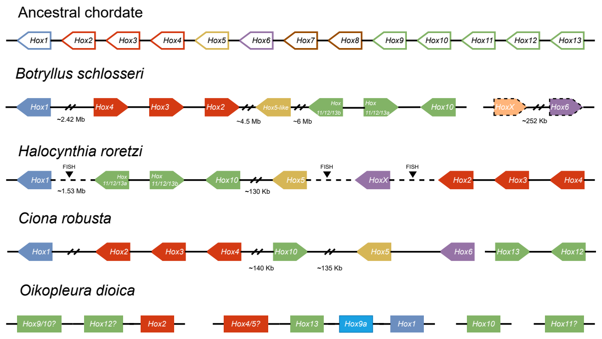

Figure 8. Representation of the Hox genes retrieved in the new assembly of *B. schlosseri* compared to the supposed original single Hox cluster of the chordate ancestor and other tunicates. Linked genes (present on the same scaffold) are connected by a solid line, while a dashed line is used when the linkage has been deduced using another method. When known, the transcription orientation is indicated by an arrow-shaped rectangle, which is surrounded by a dashed line when the Hox gene was retrieved with low confidence.

Conclusion

Tunicate genomes are known for their rapid evolution, featuring high rates of molecular divergence, extensive genomic rearrangements, and are generally remarkably compact compared to vertebrates, though genome size varies among tunicate species [71]. Additionally, while some tunicates exhibit high levels of repetitive elements, others show moderate repeat content [45], [66]. Despite these variations, tunicate genomes share conserved non-coding elements, reflecting deep regulatory constraints within this diverse subphylum [72]. Although solitary tunicates such as *Ciona* and *Oikopleura*, along with other species, have been instrumental in shaping our understanding of tunicate genomes, colonial tunicates remain relatively understudied at the genomic level. Colonial species also introduce unique biological questions related to allorecognition, asexual reproduction, and whole-body regeneration. As a widely used model for colonial tunicates, *B. schlosseri* provides an essential reference for studying these processes, making a high-quality genome assembly particularly valuable. Comparative synteny analyses highlight both conserved and highly rearranged genomic features across tunicates, reinforcing the notion of their exceptional genomic plasticity. By making this resource available, we aim to facilitate future research into the evolutionary and functional genomics of chordates, also highlighting unique adaptations that define tunicate biology.

Methods

Sampling, DNA isolation, and sequencing

Isogenic colonies of *Botryllus schlosseri* were raised on glass slides in the marine-culture system described in Langenbacher *et al.* [21]. Genomic DNA was extracted from the colony labeled E* using Qiagen's MagAttract HMW DNA Kit (67563). Libraries were prepared and sequencing was performed at Novogene (Cambridge, UK) for Illumina 2x150 bp paired-end (PE) reads, at the Next Generation Sequencing Platform of the University of Bern (Switzerland) and Leiden Genome Technology Center (Leiden, Netherlands) for HiFi PacBio long reads round 1 and round 2, respectively (PacBio Sequel II, SMRT-bell library), and at UCAGenomix (Valbonne, France) for Oxford Nanopore (ONT) long reads (on a FLO-PRO002 flow cell with R9.4.1 pore proteins, using the SQK-LSK109 ligation sequencing kit). Nanopore base calling was performed using Guppy (RRID:SCR_023196, v3.2.10). A Hi-C library was prepared using the Arima High Coverage HiC Kit (A410110) followed by the Arima HiC+ Kit (A510008, A303011) and sequenced using Illumina (2x150 bp).

Data preprocessing

PacBio HiFi reads were processed with HiFiAdapterFilt v2.0.1 [73] to remove adapter sequences, while Porechop (RRID:SCR_016967, v0.2.4) was used to trim basic adapters from ONT reads. For Illumina reads, quality trimming and adapter clipping were performed using Trimmomatic [74] (RRID:SCR_011848, v0.39) while quality check, prior to and after trimming, was done using FastQC (RRID:SCR_01458 v0.11.5).

Genome size estimation

The genome size of colony E* was measured using an improved Feulgen protocol [75] by comparison with two standards of known C-values: *Periplaneta americana* (3.41 pg) [76], and *Lasius niger* (0.30 pg) [77]. In brief, the protocol steps included: chopping the tissues of each specimen into tiny pieces using a sterilized razor blade with a few drops of 40% glacial acetic then leaving them for 48 hours in the dark; immersing the processed slides into fixation reagent (85:10:5 volumes of methanol:formaldehyde:acetic acid); then hydrolysing them (using hydrochloric acid 5M) and staining them (using Schiff's reagent).

A digital camera (5 megapixels) mounted on a compound microscope with a 100X objective was used for imaging the slides. During the photography sessions, we maintained constant camera settings for exposure and gain, white balance calibration parameters, microscope light intensity, light condenser, and focal lens positions. In the image analysis protocol, we first outlined the nuclear boundary using the polygon tool in ImageJ [78], then extracted from ImageJ the area size of the nucleus (ASN) and the mean gray value of the nucleus (GVN). Next, we outlined in ImageJ a doughnut-shaped area surrounding the same nucleus and used it to extract the mean gray value of its background (GVB). This process was repeated for up to 30 nuclei per sample. The difference between GVB and GVN is an estimate of the average optical density (OD) of a nucleus; multiplying it by its ASN yields its integrated optical density (IOD), which is proportional to the amount of DNA in this nucleus.

Comparison of IOD values of the sample with those of the standards allows to calculate the genome size of the sample, provided that two assumptions are verified: 1) all the nuclei of a given specimen contain about the same amount of DNA; 2) the IODs of nuclei of the standards are proportional to their known C-values. To check the first assumption of the method, we used a R script to plot for each specimen the 1/OD values of their nuclei vs. their ASN values and verify that the resulting linear regression passed through the origin of the plot (Figure S3). To check the second assumption, we plotted the average IOD of each standard vs. their known C-value and verified that the resulting line passed through the origin of the plot (Figure S4). As both assumptions of the method were met, we proceeded to estimating the C-value of the sample: for that, we divided the IOD of each nucleus of each standard by its known genome size, resulting in a set of 60 integrated optical densities divided by C-values (IOD/C). Finally, we used a R script to divide each of the 30 IODs of the sample by each of the 60 IOD/C values of the standards, then plotted the distribution of the resulting 1800 estimated C-values of the sample and took the mode of its Gaussian kernel density as the most likely genome size.

A genome size estimation based on the k-mer spectrum of the Illumina reads was also performed using KMC v3.2.1 [79] and the GenomeScope2.0 [80] web server, with a k-mer size of 21 and a k-mer count cutoff of 100,000.

Collapsed genome assembly

First, the PacBio HiFi reads were assembled into contigs using hifiasm [31] with the haplotype purging option disabled (option -l0 with hifiasm in HiFi-only assembly mode). Second, uncollapsed haplotypes were purged using multiple rounds of HaploMerger2 (release 20180603) [81] until the BUSCO duplication score stabilized. Third, non-metazoan contigs were identified and removed from the assemblies using BlobToolKit v4.1.5 [82]. To this aim, contigs were aligned to the NCBI nucleotide database (accessed 2023 March 18) using BLAST [83] (RRID:SCR_001653, v2.13.0+) with the blastn command, and also to the UniProt reference proteome database (accessed on 23 March 2023) using DIAMOND [84] (RRID:SCR_016071, v2.1.6); contig HiFi coverage depth was computed using minimap2 v2.24-r1122 [85]. Using the "bestsumorder" rule of BlobToolKit, only the contigs assigned to the taxon "Chordata" or without a match ("no-hit") were kept. Finally, a BLASTN search for fragments of the mitochondrial genome among the contigs was performed using the published complete mitochondrial genome of *B. schlosseri* (RefSeq NC_021463.1) [28] to remove contigs showing at least 80% coverage and identity with the query sequence.

To scaffold the assemblies, PacBio HiFi and Illumina reads were first mapped to the assemblies using minimap2. Putative misjoined regions were then identified and automatically split using CRAQ v1.0.9 [33] with default parameters except for the addition of –break. Hi-C reads were subsequently mapped to the output of CRAQ using the Arima Genomics mapping pipeline script arima_mapping_pipeline.sh [86], and YaHS v1.2 [34] was run with default parameters to scaffold the assemblies. CRAQ was then applied to the results, and finally the scaffolds were manually curated using PretextMap (RRID:SCR_022023,v0.1.9) and PretextView (RRID:SCR_022024, v0.2.5). Metrics for the assemblies were computed using SeqKit v2.3.0 [87] (parameter stats -a). The quality and completeness were checked using KAT v2.4.2 [88] on k-mers from both PacBio HiFi and Illumina reads, and BUSCO v5.4.4 [89] (using the -m genome mode) with the metazoa_odb10 dataset.

Haplotype-resolved assembly

Two haplotype-resolved assemblies (haplotype 1 and haplotype 2) were generated using hifiasm in Hi-C Integrated Assembly mode, which directly integrates Hi-C reads. To refine the assemblies, uncollapsed sequences were purged for haplotype 1 using purge_dups [90], and BlobToolKit was employed, as with the collapsed assembly, to filter out contamination, resulting in contig-level assemblies (see Figures S9 and S6). The scaffolding process for haplotype 1 and haplotype 2 followed the same method as for the collapsed assembly, with the final scaffolds ordered based on alignment to the collaped assembly rather than by descending size (see Figure S15).

Genome annotation

For all the assemblies, repetitive elements were identified using RepeatModeler and RepeatMasker pipeline. A *de novo* repeat library was generated using RepeatModeler2 v2.0.3 [91] and used as input for RepeatMasker (SCR_012954, v4.0.6) to detect, classify and soft-mask repeats in the genomic sequences. RNA-seq reads were aligned to the soft-masked assemblies using STAR v2.7.10b (default options) [92]. Based on the aligned transcripts, on a list of proteins from OrthoDB v11 for Metazoa [93] as extrinsic evidence and on the soft-masked assemblies, genes were predicted and annotated using the BRAKER3 v3.06 pipeline for RNA-Seq and protein data without training or gene prediction with untranslated regions (UTRs) parameters [94, 95, 96, 97, 98, 99, 100, 101, 102, 103, 104, 105, 106, 49]. A refinement of the initial BRAKER3 structural annotation and the addition of UTRs were then performed with an implementation the PASA pipeline v2.4.1 [50] together with EVidenceModeler (EVM) [51] (RRID:SCR_014659, v2.1.0). A

third of the RNA-seq reads of the Rodriguez *et al.* (2014) transcriptome [23] was aligned again to the assemblies and their BRAKER3 annotation using STAR (MAX_INTRON_SIZE=20000) [92] (RRID:SCR_015899, v2.7.10b) and assembled with StringTie [107] (RRID:SCR_016323, v2.2.1) using the BRAKER3 annotation as a reference. The PASA alignment assembly step was then run as described on its GitHub Wiki with the transcripts assembled by StringTie and independently with Trinity assemblies of public available RNA-seq reads [23, 25, 8]. TransDecoder [108] was run within PASA to identify coding sequences within the assembled transcripts. A consensus annotation of coding sequences (CDSs) was found by EVM by leveraging both the transcripts and coding sequences identified for each RNAseq by PASA (evidence weights: 1 for BRAKER3 input, 5 for PASA transcripts and TransDecoder CDSs). The gene models were refined, with addition of the UTRs and isoforms by running PASA genome annotation step sequentially with each previously generated PASA database (using EVM output as the first reference, then the output of the previous PASA genome annotation run). Functional annotation was performed starting from the structural annotation obtained with the BRAKER3-PASA pipeline. Eggnog-mapper [52, 109] and Interproscan [53, 54] were used for orthology-based annotation (nr, KEGG, GO terms) and for protein domains prediction respectively. Both approaches were used as input for the Funannotate pipeline (RRID:SCR_023039, v1.8.15), yielding a gff3 and a GenBank file with functional annotations.

Mitochondrial genome assembly

The mitochondrial genome was reconstructed using NOVOPlasty [110] (RRID:SCR_017335, v4.3.1). A COI fragment from *Botryllus schlosseri* clade A1 (GenBank MT731471.1) was used as a seed in combination with our Illumina reads as input.

Comparative genomics analyses

The genome assemblies and annotations for the comparison of the collapsed assembly with other tunicate species were retrieved from ANISEED [111] for *Botrylloides leachii*, *Ciona robusta*, and for the first assembly of *Botryllus schlosseri*, while *Oikopleura dioica* originates from [47], *Salpa thompsoni* from [48] and *Styela clava* from [56]. Macrosynteny analyses were performed using the odp tool [59]. For each species, analyses were based on the longest protein isoforms generated from their annotation file using the scripts agat_sp_keep_longest_isoform.pl and agat_sp_extract_sequences.pl from AGAT (RRID:SCR_027223, v0.7.0).

Phylogenetic analyses

COI fragments were retrieved from [44] and aligned with MUSCLE [112]. A maximum-likelihood tree was generated using MEGA5 [113] with the model HKY+I+G followed by 1000 bootstrap replicates. Phylogenetic analyses of *Botryllus schlosseri* Hox genes were performed using sequences retrieved from Sekigami *et al.* [69]. First, the sequences were aligned using MUSCLE [112] as implemented in AliView [114], then IQ-TREE 2 [115] was used to build a maximum-likelihood phylogeny with the best-fit model JTT+R6 [116, 117] selected by ModelFinder [118] following the Bayesian information criterion [119] and with 10,000 ultrafast bootstrap replicates [120]. The same alignment was used to build a Bayesian tree using MrBayes (RRID:SCR_012067, V.3.2.7) (gamma-distributed rate variation across sites; mixed AA substitution models).

Data availability

The genomic and transcriptomic sequence data generated in this study are available under the BioProject accessions: PRJNA1225683. The gene expression data utilized in this study are available from The Gene Expression Omnibus https://www.ncbi.nlm.nih.gov/geo/ under accessions: GSE62112, GSE193805. All additional supporting data are available in the GigaScience repository, GigaDB [121] and in Octopus [122].

Declarations

Consent for publication

Not applicable.

Competing Interests

The authors declare that they have no competing interests.

Funding

This work was supported by ANR (ANR-14-CE02-0019-01 and ANR-24-CE02-2277), INSB-DBM and Sorbonne University AAP Emergence 2021 to ST, by FAPESP 15/50164-5 & 19/06927-5 to FDB and by the Fonds de la Recherche Scientifique (F.R.S.-FNRS) via PDR grant T.0078.23 to JFF.

Author's Contributions

ODT carried out the majority of the assembly and analyses. ST, ODT, and JFF conceived the project and drafted the manuscript with the contribution of ML. MMT conducted the Feulgen analyses. SB assisted with the initial stages of the assembly and provided part of the HiFi dataset. ML and PD handled the annotation and contributed to the analyses. AA, FDB, and RF provided valuable technical and scientific insights. ST and JFF supervised the research. All authors reviewed and approved the final version of the manuscript.

Acknowledgements

We would like to thank EMBRC-France and in particular Laurent Gilletta for isolating isogenic colonies and maintaining the aquaculture system. Thanks also to the Next Generation Sequencing Platform of the University of Bern (Switzerland) for providing part of the HiFi sequencing. We thank Aaron Reinke for pointing out the presence of macrosporidia sequences in the BloobToolKit analyses, Vitoria Tobias Santos for filtering part of the RNAseq dataset used for the annotation, Carmela Gissi and Lino Ometto for useful scientific exchange as well as the three reviewers for useful feedback.

References

1. Delsuc F, Brinkmann H, Chourrout D, Philippe H. Tunicates and not cephalochordates are the closest living relatives of vertebrates. Nature 2006;439(7079):965–968.
2. Alié A, Hiebert LS, Scelzo M, Tiozzo S. The eventful history of nonembryonic development in tunicates. Journal of Experimental Zoology Part B: Molecular and Developmental Evolution 2021;336(3):250–266.
3. Stolfi A, Brown FD. Tunicata. In: Wanninger A, editor. Evolu-

tionary Developmental Biology of Invertebrates 6: Deuterostomia Vienna: Springer; 2015.p. 135–204.
4. Hiebert LS, Simpson C, Tiozzo S. Coloniality, clonality, and modularity in animals: The elephant in the room. Journal of Experimental Zoology Part B: Molecular and Developmental Evolution 2021;336(3):198–211.
5. Manni L, Gasparini F, Hotta K, Ishizuka KJ, Ricci L, Tiozzo S, et al. Ontology for the asexual development and anatomy of the colonial chordate *Botryllus schlosseri*. PLoS ONE 2014;9(5):e96434.
6. Sabbadin A, Zaniolo G, Majone F. Determination of polarity and bilateral asymmetry in palleal and vascular buds of the ascidian *Botryllus schlosseri*. Developmental Biology 1975;46(1):79–87.
7. Nourizadeh S, Kassmer S, Rodriguez D, Hiebert LS, De Tomaso AW. Whole body regeneration and developmental competition in two botryllid ascidians. EvoDevo 2021-12-15;12(1):15.
8. Ricci L, Salmon B, Olivier C, Andreoni-Pham R, Chaurasia A, Alié A, et al. The onset of whole-body regeneration in *Botryllus schlosseri*: morphological and molecular characterization. Frontiers in Cell and Developmental Biology 2022-02-14;0:173.
9. Laird DJ, De Tomaso AW, Weissman IL. Stem cells are units of natural selection in a colonial ascidian. Cell 2005;123(7):1351–1360.
10. Laird DJ, De Tomaso AW. Predatory stem cells in the non-zebrafish chordate, *Botryllus schlosseri*. Zebrafish 2005;1(4):357–361.
11. Brown FD, Tiozzo S, Roux MM, Ishizuka K, Swalla BJ, De Tomaso AW. Early lineage specification of long-lived germline precursors in the colonial ascidian *Botryllus schlosseri*. Development 2009;136(20):3485–3494.
12. Pancer Z, Gershon H, Rinkevich B. Coexistence and possible parasitism of somatic and germ cell lines in chimeras of the colonial urochordate *Botryllus schlosseri*. The Biological Bulletin 1995;189(2):106–112.
13. Stoner DS, Weissman IL. Somatic and germ cell parasitism in a colonial ascidian: possible role for a highly polymorphic allorecognition system. Proceedings of the National Academy of Sciences of the United States of America 1996;93(26):15254–15259.
14. Manni L, Anselmi C, Cima F, Gasparini F, Voskoboynik A, Martini M, et al. Sixty years of experimental studies on the blastogenesis of the colonial tunicate *Botryllus schlosseri*. Developmental Biology 2019;448(2):293–308.
15. Kassmer SH, Rodriguez D, De Tomaso AW. Colonial ascidians as model organisms for the study of germ cells, fertility, whole body regeneration, vascular biology and aging. Current Opinion in Genetics & Development 2016;39:101–106.
16. Taketa DA, De Tomaso AW. *Botryllus schlosseri* allorecognition: tackling the enigma. Developmental & Comparative Immunology 2015;48(1):254–265.
17. Nydam ML. Evolution of allorecognition in the Tunicata. Biology 2020;9(6):129.
18. Epelbaum A, Therriault TW, Paulson A, Pearce CM. Botryllid tunicates: Culture techniques and experimental procedures. Aquatic Invasions 2009;4(1):111–120.
19. Gasparini F, Manni L, Cima F, Zaniolo G, Burighel P, Caicci F, et al. Sexual and asexual reproduction in the colonial ascidian *Botryllus schlosseri*. Genesis 2015;53(1):105–120.
20. Wawrzyniak MK, Matas Serrato LA, Blanchoud S. Long-term monitoring data logs of a recirculating artificial seawater based colonial ascidian aquaculture. Data in Brief 2021-10;38:107372.
21. Langenbacher AD, Rodriguez D, Di Maio A, De Tomaso AW. Whole-mount fluorescent *in situ* hybridization staining of the colonial tunicate *Botryllus schlosseri*. genesis 2015;53(1):194–201.
22. Manni L, Zaniolo G, Cima F, Burighel P, Ballarin L. *Botryllus schlosseri*: A model ascidian for the study of asexual reproduction. Developmental Dynamics 2007;236(2):335–352.
23. Rodriguez D, Sanders EN, Farell K, Langenbacher AD, Taketa DA, Hopper MR, et al. Analysis of the basal chordate *Botryllus schlosseri* reveals a set of genes associated with fertility. BMC genomics 2014;15(1):1183.
24. Campagna D, Gasparini F, Franchi N, Vitulo N, Ballin F, Manni L, et al. Transcriptome dynamics in the asexual cycle of the chordate *Botryllus schlosseri*. BMC Genomics 2016;17(1):275.
25. Ricci L, Chaurasia A, Lapébie P, Dru P, Helm RR, Copley RR, et al. Identification of differentially expressed genes from multipotent epithelia at the onset of an asexual development. Scientific Reports 2016;6:27357.
26. Rosental B, Kowarsky M, Seita J, Corey DM, Ishizuka KJ, Palmeri KJ, et al. Complex mammalian-like haematopoietic system found in a colonial chordate. Nature 2018;564(7736):425–429.
27. Kowarsky M, Anselmi C, Hotta K, Burighel P, Zaniolo G, Caicci F, et al. Sexual and asexual development: two distinct programs producing the same tunicate. Cell Reports 2021;34(4):108681.
28. Voskoboynik A, Neff NF, Sahoo D, Newman AM, Pushkarev D, Koh W, et al. The genome sequence of the colonial chordate, *Botryllus schlosseri*. eLife 2013;2:e00569.
29. Lawniczak MKN, Durbin R, Flicek P, Lindblad-Toh K, Wei X, Archibald JM, et al. Standards recommendations for the Earth BioGenome Project. Proceedings of the National Academy of Sciences 2022;119(4):e2115639118.
30. De Tomaso AW, Saito Y, Ishizuka KJ, Palmeri KJ, Weissman IL. Mapping the genome of a model protochordate. I. A low resolution genetic map encompassing the fusion/histocompatibility (Fu/HC) locus of *Botryllus schlosseri*. Genetics 1998;149(1):277–287.
31. Cheng H, Concepcion GT, Feng X, Zhang H, Li H. Haplotype-resolved de novo assembly using phased assembly graphs with hifiasm. Nature Methods 2021;18(2):170–175.
32. Bojko J, Reinke AW, Stentiford GD, Williams B, Rogers MSJ, Bass D. Microsporidia: a new taxonomic, evolutionary, and ecological synthesis. Trends in Parasitology 2022;38(8):642–659.
33. Li K, Xu P, Wang J, Yi X, Jiao Y. Identification of errors in draft genome assemblies at single-nucleotide resolution for quality assessment and improvement. Nature Communications 2023;14(1):6556.
34. Zhou C, McCarthy SA, Durbin R. YaHS: yet another Hi-C scaffolding tool. Bioinformatics 2023;39(1):btac808.
35. Colombera D. The karyology of the colonial ascidian *Botryllus schlosseri* (Pallas). Caryologia 1969;22(4):339–349.
36. Zeng X, Yi Z, Zhang X, Du Y, Li Y, Zhou Z, et al. Chromosome-level scaffolding of haplotype-resolved assemblies using Hi-C data without reference genomes. Nature Plants 2024;10(8):1184–1200.
37. Bandi V, Gutwin C, Siri JN, Neufeld E, Sharpe A, Parkin I. Visualization tools for genomic conservation. In: Edwards D, editor. Plant Bioinformatics: Methods and Protocols New York, NY: Springer US; 2022.p. 285–308.
38. Wang Y, Tang H, DeBarry JD, Tan X, Li J, Wang X, et al. MCScanX: a toolkit for detection and evolutionary analysis of gene synteny and collinearity. Nucleic Acids Research 2012;40(7):e49.
39. Simão FA, Waterhouse RM, Ioannidis P, Kriventseva EV, Zdobnov EM. BUSCO: assessing genome assembly and annotation completeness with single-copy orthologs. Bioinformatics 2015;31(19):3210–3212.
40. Guiglielmoni N, Houtain A, Derzelle A, Van Doninck K, Flot JF. Overcoming uncollapsed haplotypes in long-read as-

semblies of non-model organisms. BMC Bioinformatics 2021;22(1):303.
41. Simion P, Narayan J, Houtain A, Derzelle A, Baudry L, Nicolas E, et al. Chromosome-level genome assembly reveals homologous chromosomes and recombination in asexual rotifer *Adineta vaga*. Science Advances 2021;7(41):eabg4216.
42. López-Legentil S, Turon X, Planes S. Genetic structure of the star sea squirt, *Botryllus schlosseri* , introduced in southern European harbours. Molecular Ecology 2006;15(13):3957–3967.
43. Bock DG, MacIsaac HJ, Cristescu ME. Multilocus genetic analyses differentiate between widespread and spatially restricted cryptic species in a model ascidian. Proceedings of the Royal Society B: Biological Sciences 2012;279(1737):2377–2385.
44. Salonna M, Gasparini F, Huchon D, Montesanto F, Haddas-Sasson M, Ekins M, et al. An elongated COI fragment to discriminate botryllid species and as an improved ascidian DNA barcode. Scientific Reports 2021;11(1):4078.
45. Blanchoud S, Rutherford K, Zondag L, Gemmell NJ, Wilson MJ. *De novo* draft assembly of the *Botrylloides leachii* genome provides further insight into tunicate evolution. Scientific Reports 2018;8(1):5518.
46. Satou Y, Nakamura R, Yu D, Yoshida R, Hamada M, Fujie M, et al. A nearly complete genome of *Ciona intestinalis* type A (*C. robusta*) reveals the contribution of inversion to chromosomal evolution in the genus *Ciona*. Genome Biology and Evolution 2019;11(11):3144–3157.
47. Bliznina A, Masunaga A, Mansfield MJ, Tan Y, Liu AW, West C, et al. Telomere-to-telomere assembly of the genome of an individual *Oikopleura dioica* from Okinawa using Nanopore-based sequencing. BMC Genomics 2021;22(1):222.
48. Castellano KR, Batta-Lona P, Bucklin A, O'Neill RJ. *Salpa* genome and developmental transcriptome analyses reveal molecular flexibility enabling reproductive success in a rapidly changing environment. Scientific Reports 2023;13(1):21056.
49. Gabriel L, Brůna T, Hoff KJ, Ebel M, Lomsadze A, Borodovsky M, et al. BRAKER3: Fully automated genome annotation using RNA-seq and protein evidence with GeneMark-ETP, AUGUSTUS, and TSEBRA. Genome Res 2024;34(5):769–777.
50. Haas BJ. Improving the *Arabidopsis* genome annotation using maximal transcript alignment assemblies. Nucleic Acids Research 2003;31(19):5654–5666.
51. Haas BJ, Salzberg SL, Zhu W, Pertea M, Allen JE, Orvis J, et al. Automated eukaryotic gene structure annotation using EVidenceModeler and the Program to Assemble Spliced Alignments. Genome Biology 2008;9(1):R7.
52. Cantalapiedra CP, Hernández-Plaza A, Letunic I, Bork P, Huerta-Cepas J. eggNOG-mapper v2: functional annotation, orthology assignments, and domain prediction at the metagenomic scale. Molecular Biology and Evolution 2021;38(12):5825–5829.
53. Blum M, Chang HY, Chuguransky S, Grego T, Kandasaamy S, Mitchell A, et al. The InterPro protein families and domains database: 20 years on. Nucleic Acids Research 2021;49(D1):D344–D354.
54. Jones P, Binns D, Chang HY, Fraser M, Li W, McAnulla C, et al. InterProScan 5: genome-scale protein function classification. Bioinformatics 2014;30(9):1236–1240.
55. Kanehisa M, Sato Y, Morishima K. BlastKOALA and GhostKOALA: KEGG tools for functional characterization of genome and metagenome sequences. Journal of Molecular Biology 2016;428(4):726–731.
56. Wei J, Zhang J, Lu Q, Ren P, Guo X, Wang J, et al. Genomic basis of environmental adaptation in the leathery sea squirt (*Styela clava*). Molecular Ecology Resources 2020;20(5):1414–1431.
57. Delsuc F, Philippe H, Tsagkogeorga G, Simion P, Tilak MK, Turon X, et al. A phylogenomic framework and timescale for comparative studies of tunicates. BMC Biology 2018;16(1):1–14.
58. Simakov O, Marlétaz F, Yue JX, O'Connell B, Jenkins J, Brandt A, et al. Deeply conserved synteny resolves early events in vertebrate evolution. Nature Ecology & Evolution 2020-06;4(6):820–830.
59. Schultz DT, Haddock SHD, Bredeson JV, Green RE, Simakov O, Rokhsar DS. Ancient gene linkages support ctenophores as sister to other animals. Nature 2023;618(7963):110–117.
60. Simakov O, Bredeson J, Berkoff K, Marletaz F, Mitros T, Schultz DT, et al. Deeply conserved synteny and the evolution of metazoan chromosomes. Science Advances 2022;8(5):eabi5884.
61. Lewin TD, Liao IJY, Chen ME, Bishop JDD, Holland PWH, Luo YJ. Fusion, fission, and scrambling of the bilaterian genome in Bryozoa. Genome Research 2025;35(1):78–92.
62. Plessy C, Mansfield MJ, Bliznina A, Masunaga A, West C, Tan Y, et al. Extreme genome scrambling in marine planktonic *Oikopleura dioica* cryptic species. Genome Research 2024;34(3):426–440.
63. Vargas-Chávez C, Benítez-Álvarez L, Martínez-Redondo GI, Álvarez-González L, Salces-Ortiz J, Eleftheriadi K, et al. An episodic burst of massive genomic rearrangements and the origin of non-marine annelids. Nature Ecology & Evolution 2025;9(7):1263–1279.
64. Lewin TD, Liao IJY, Luo YJ. Annelid comparative genomics and the evolution of massive lineage-specific genome rearrangement in bilaterians. Molecular Biology and Evolution 2024;41(9):msae172.
65. Schultz D, Heath-Heckman E, Winchell C, Kuo DH, Yu Ys, Oberauer F, et al. Acceleration of genome rearrangement in clitellate annelids. bioRxiv 2024; doi:10.1101/2024.05.12.593736.
66. Berna L, Alvarez-Valin F. Evolutionary genomics of fast evolving tunicates. Genome Biology and Evolution 2014;6(7):1724–1738.
67. Monteiro AS, Ferrier DEK. Hox genes are not always Colinear. International Journal of Biological Sciences 2006;2(3):95–103.
68. DeBiasse MB, Colgan WN, Harris L, Davidson B, Ryan JF. Inferring tunicate relationships and the evolution of the tunicate Hox cluster with the genome of *Corella inflata*. Genome Biology and Evolution 2020;12(6):948–964.
69. Sekigami Y, Kobayashi T, Omi A, Nishitsuji K, Ikuta T, Fujiyama A, et al. Hox gene cluster of the ascidian, *Halocynthia roretzi*, reveals multiple ancient steps of cluster disintegration during ascidian evolution. Zoological Letters 2017;3:17.
70. Gaunt SJ. Seeking sense in the Hox gene cluster. Journal of Developmental Biology 2022;10(4):48.
71. Caputi L. Evolutionary genomics of tunicates. Science Reviews Biology 2024;3(2):22–32.
72. Sanges R, Hadzhiev Y, Gueroult-Bellone M, Roure A, Ferg M, Meola N, et al. Highly conserved elements discovered in vertebrates are present in non-syntenic loci of tunicates, act as enhancers and can be transcribed during development. Nucleic Acids Research 2013;41(6):3600–3618.
73. Sim SB, Corpuz RL, Simmonds TJ, Geib SM. HiFiAdapterFilt, a memory efficient read processing pipeline, prevents occurrence of adapter sequence in PacBio HiFi reads and their negative impacts on genome assembly. BMC Genomics 2022;23(1):157.
74. Bolger AM, Lohse M, Usadel B. Trimmomatic: a flexible trimmer for Illumina sequence data. Bioinformatics 2014;30(15):2114–2120.
75. M.Tawfeeq M, Swaelus U, Rodriguez Gaudray F, Ennes Silva F, Grumiau L, Verdebout T, et al. Refining Feulgen: low-cost and accurate genome size measurements for everyone.

bioRxiv 2025; doi:10.1101/2025.XX.XX.XXXXXX.
76. Wang L, Xiong Q, Saelim N, Wang L, Nong W, Wan ATY, et al. Genome assembly and annotation of *Periplaneta americana* reveal a comprehensive cockroach allergen profile. Allergy 2023;78(4):1088–1103.
77. Vizueta J, Xiong Z, Ding G, Larsen RS, Ran H, Gao Q, et al. Adaptive radiation and social evolution of the ant. Cell 2025;.
78. Schneider CA, Rasband WS, Eliceiri KW. NIH Image to ImageJ: 25 years of image analysis. Nature Methods 2012;9(7):671–675.
79. Kokot M, Długosz M, Deorowicz S. KMC 3: counting and manipulating k-mer statistics. Bioinformatics 2017;33(17):2759–2761.
80. Ranallo-Benavidez TR, Jaron KS, Schatz MC. GenomeScope 2.0 and Smudgeplot for reference-free profiling of polyploid genomes. Nature Communications 2020;11(1):1432.
81. Huang S, Kang M, Xu A. HaploMerger2: rebuilding both haploid sub-assemblies from high-heterozygosity diploid genome assembly. Bioinformatics 2017;33(16):2577–2579.
82. Challis R, Richards E, Rajan J, Cochrane G, Blaxter M. BlobToolKit – interactive quality assessment of genome assemblies. G3: Genes|Genomes|Genetics 2020;10(4):1361–1374.
83. Camacho C, Coulouris G, Avagyan V, Ma N, Papadopoulos J, Bealer K, et al. BLAST+: architecture and applications. BMC Bioinformatics 2009;10(1):421.
84. Buchfink B, Reuter K, Drost HG. Sensitive protein alignments at tree-of-life scale using DIAMOND. Nature Methods 2021;18(4):366–368.
85. Li H. New strategies to improve minimap2 alignment accuracy. Bioinformatics 2021;37(23):4572–4574.
86. Ghurye J, Rhie A, Walenz BP, Schmitt A, Selvaraj S, Pop M, et al. Integrating Hi-C links with assembly graphs for chromosome-scale assembly. PLOS Computational Biology 2019;15(8):e1007273.
87. Shen W, Le S, Li Y, Hu F. SeqKit: a cross-platform and ultrafast toolkit for FASTA/Q file manipulation. PLoS ONE 2016;11(10):e0163962.
88. Mapleson D, Garcia Accinelli G, Kettleborough G, Wright J, Clavijo BJ. KAT: a K-mer analysis toolkit to quality control NGS datasets and genome assemblies. Bioinformatics 2017;33(4):574–576.
89. Manni M, Berkeley MR, Seppey M, Simão FA, Zdobnov EM. BUSCO update: novel and streamlined workflows along with broader and deeper phylogenetic coverage for scoring of eukaryotic, prokaryotic, and viral genomes. Molecular Biology and Evolution 2021;38(10):4647–4654.
90. Guan D, McCarthy SA, Wood J, Howe K, Wang Y, Durbin R. Identifying and removing haplotypic duplication in primary genome assemblies. Bioinformatics 2020;36(9):2896–2898.
91. Flynn JM, Hubley R, Goubert C, Rosen J, Clark AG, Feschotte C, et al. RepeatModeler2 for automated genomic discovery of transposable element families. Proceedings of the National Academy of Sciences 2020;117(17):9451–9457.
92. Dobin A, Davis CA, Schlesinger F, Drenkow J, Zaleski C, Jha S, et al. STAR: ultrafast universal RNA-seq aligner. Bioinformatics 2013;29(1):15–21.
93. Kuznetsov D, Tegenfeldt F, Manni M, Seppey M, Berkeley M, Kriventseva EV, et al. OrthoDB v11: annotation of orthologs in the widest sampling of organismal diversity. Nucleic Acids Research 2023;51(D1):D445–D451.
94. Lomsadze A. Gene identification in novel eukaryotic genomes by self-training algorithm. Nucleic Acids Research 2005;33(20):6494–6506.
95. Stanke M, Schöffmann O, Morgenstern B, Waack S. Gene prediction in eukaryotes with a generalized hidden Markov model that uses hints from external sources. BMC Bioinformatics 2006;7(1):62.
96. Lomsadze A, Burns PD, Borodovsky M. Integration of mapped RNA-Seq reads into automatic training of eukaryotic gene finding algorithm. Nucleic Acids Research 2014;42(15):e119–e119.
97. Gotoh O. A space-efficient and accurate method for mapping and aligning cDNA sequences onto genomic sequence. Nucleic Acids Research 2008;36(8):2630–2638.
98. Iwata H, Gotoh O. Benchmarking spliced alignment programs including Spaln2, an extended version of Spaln that incorporates additional species-specific features. Nucleic Acids Research 2012;40(20):e161–e161.
99. Buchfink B, Xie C, Huson DH. Fast and sensitive protein alignment using DIAMOND. Nature Methods 2015;12(1):59–60.
100. Brůna T, Lomsadze A, Borodovsky M. GeneMark-EP+: eukaryotic gene prediction with self-training in the space of genes and proteins. NAR Genomics and Bioinformatics 2020;2(2):lqaa026.
101. Pertea G, Pertea M. GFF Utilities: GffRead and GffCompare. F1000Research 2020;9:ISCB Comm J–304.
102. Kovaka S, Zimin AV, Pertea GM, Razaghi R, Salzberg SL, Pertea M. Transcriptome assembly from long-read RNA-seq alignments with StringTie2. Genome Biology 2019;20(1):278.
103. Stanke M, Diekhans M, Baertsch R, Haussler D. Using native and syntenically mapped cDNA alignments to improve *de novo* gene finding. Bioinformatics 2008;24(5):637–644.
104. Hoff KJ, Lomsadze A, Borodovsky M, Stanke M, Kollmar M. Whole-genome annotation with BRAKER. In: Gene prediction: methods and protocols No. 1962 in Methods in Molecular Biology, Springer; 2019.p. 65–95.
105. Hoff KJ, Lange S, Lomsadze A, Borodovsky M, Stanke M. BRAKER1: unsupervised RNA-Seq-based genome annotation with GeneMark-ET and AUGUSTUS. Bioinformatics 2016;32(5):767–769.
106. Brůna T, Hoff KJ, Lomsadze A, Stanke M, Borodovsky M. BRAKER2: automatic eukaryotic genome annotation with GeneMark-EP+ and AUGUSTUS supported by a protein database. NAR Genomics and Bioinformatics 2021;3(1):lqaa108.
107. Pertea M, Pertea GM, Antonescu CM, Chang TC, Mendell JT, Salzberg SL. StringTie enables improved reconstruction of a transcriptome from RNA-seq reads. Nature Biotechnology 2015;33(3):290–295.
108. Haas BJ, Papanicolaou A, Yassour M, Grabherr M, Blood PD, Bowden J, et al. *De novo* transcript sequence reconstruction from RNA-seq using the Trinity platform for reference generation and analysis. Nature Protocols 2013;8(8):1494–1512.
109. Huerta-Cepas J, Szklarczyk D, Heller D, Hernández-Plaza A, Forslund SK, Cook H, et al. eggNOG 5.0: a hierarchical, functionally and phylogenetically annotated orthology resource based on 5090 organisms and 2502 viruses. Nucleic Acids Research 2019;47(D1):D309–D314.
110. Dierckxsens N, Mardulyn P, Smits G. NOVOPlasty: de novo assembly of organelle genomes from whole genome data. Nucleic Acids Research 2017;45(4):e18.
111. Dardaillon J, Dauga D, Simion P, Faure E, Onuma TA, DeBiasse MB, et al. ANISEED 2019: 4D exploration of genetic data for an extended range of tunicates. Nucleic Acids Research 2020;48(D1):D668–D675.
112. Edgar RC. MUSCLE: multiple sequence alignment with high accuracy and high throughput. Nucleic Acids Research 2004;32(5):1792–1797.
113. Tamura K, Peterson D, Peterson N, Stecher G, Nei M, Kumar S. MEGA5: Molecular Evolutionary Genetics Analysis using maximum likelihood, evolutionary distance, and maximum parsimony methods. Molecular Biology and Evolution 2011;28(10):2731–2739.
114. Larsson A. AliView: a fast and lightweight alignment viewer and editor for large datasets. Bioinformatics

2014;30(22):3276–3278.

115. Minh BQ, Schmidt HA, Chernomor O, Schrempf D, Woodhams MD, von Haeseler A, et al. IQ-TREE 2: new models and efficient methods for phylogenetic inference in the genomic era. Molecular Biology and Evolution 2020;37(5):1530–1534.
116. Jones DT, Taylor WR, Thornton JM. The rapid generation of mutation data matrices from protein sequences. Bioinformatics 1992;8(3):275–282.
117. Yang Z. A space-time process model for the evolution of DNA sequences. Genetics 1995;139(2):993–1005.
118. Kalyaanamoorthy S, Minh BQ, Wong TKF, von Haeseler A, Jermiin LS. ModelFinder: fast model selection for accurate phylogenetic estimates. Nature Methods 2017;14(6):587–589.
119. Schwarz G. Estimating the dimension of a model. The Annals of Statistics 1978;6(2):461–464.
120. Hoang DT, Chernomor O, von Haeseler A, Minh BQ, Vinh LS. UFBoot2: improving the ultrafast bootstrap approximation. Molecular Biology and Evolution 2018;35(2):518–522.
121. De Thier O, Lebel M, M.Tawfeeq M, Faure R, Dru P, Blanchoud S, et al., Supporting data for "First chromosome-level genome assembly of the colonial chordate model *Botryllus schlosseri* (Tunicata)". GigaScience Database; 2025. `https://doi.org/10.5524/102718`.
122. Dru P, Octopus: LBDV bioinformatics server; 2025. `https://octopus.obs-vlfr.fr/public/Botryllus_Genome_2025/`.
123. Lin Y, Ye C, Li X, Chen Q, Wu Y, Zhang F, et al. quarTeT: a telomere-to-telomere toolkit for gap-free genome assembly and centromeric repeat identification. Horticulture Research 2023;10(8):uhad127.
124. Cabanettes F, Klopp C. D-GENIES: dot plot large genomes in an interactive, efficient and simple way. PeerJ 2018;6:e4958.
125. Ronquist F, Huelsenbeck JP. MrBayes 3: Bayesian phylogenetic inference under mixed models. Bioinformatics 2003;19(12):1572–1574.

Supplementary Figures

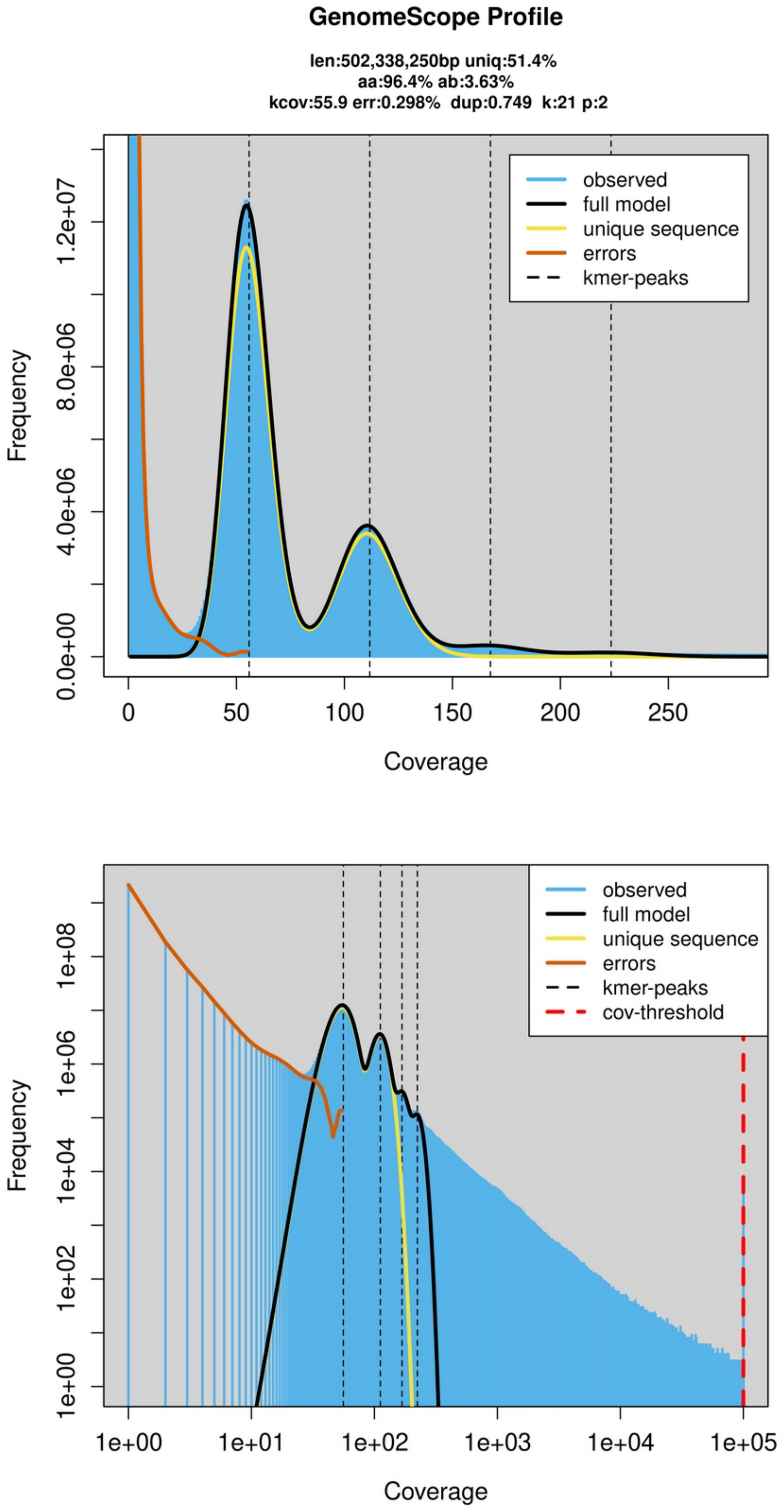

Figure S1. Genomescope2.0 results obtained with the Illumina reads; a k-mer length of 21 and a maximum counts of 100,000.

Assembly			
	**Collapsed**	**Haplotype 1**	**Haplotype 2**
Total length (Mbp)<br>(Chr. only)	533<br>(513)	496<br>(480)	494<br>(464)
No. scaffolds	254	219	410
N50 (Mbp)	30 6	29	29
GC (%)	40.52	40.52	40.53
BUSCO	C:91.6%; S:90.7%; D:0.9%<br>F:3.1%; M:5.3%	C:90.9%; S:89.9%; D:1.0%<br>F:3.6%; M:5.5%	C:91.2%; S:88.7%; D:2.5%<br>F:3.1%; M:5.7%
**Annotation**			
	**Collapsed**	**Haplotype 1**	**Haplotype 2**
No. genes	22,275	21,802	21,831
No. mRNAs	30,813	30,298	30,361
BUSCO	C:92.4%; S:79.7%; D:12.7%<br>F:1.8%; M:5.8%	C:91.6%; S:80.4%; D:11.2%<br>F:1.9%; M:6.5%	C:92.1%; S:79.1%; D:13.0%<br>F:1.6%; M:6.3%

Table S1. Metrics for the collapsed, haplotype 1, and haplotype 2 assemblies.

Chromosome	Collapsed	Haplotype 1	Haplotype 2
1	50,110	41,841 (83%)	28,053 (56%)
2	44,705	40,598 (91%)	45,038 (101%)
3	41,815	38,360 (92%)	29,155 (70%)
4	33,037	30,504 (92%)	32,607 (99%)
5	32,846	28,210 (86%)	28,942 (88%)
6	32,755	31,460 (96%)	32,176 (98%)
7	30,789	29,986 (97%)	29,040 (94%)
8	30,038	28,423 (95%)	28,982 (96%)
9	29,634	27,923 (94%)	30,153 (102%)
10	29,082	28,687 (99%)	27,860 (96%)
11	28,207	29,008 (103%)	27,153 (96%)
12	27,998	26,703 (95%)	26,494 (95%)
13	26,676	25,071 (94%)	25,027 (94%)
14	25,737	25,698 (100%)	25,542 (99%)
15	24,956	24,523 (98%)	23,565 (94%)
16	24,314	22,647 (93%)	23,761 (98%)

Table S2. Comparison of the putative chromosome sizes (in kbp) across the three different assemblies. The putative chromosomes correspond to the 16 longest scaffolds, ordered in descending size for the collapsed assembly. For the haplotype 1 and haplotype 2 assemblies, the scaffold order is based on their alignment to the collapsed assembly, with percentages in parentheses indicating their size relative to the reference collapsed assembly.

Measure	*B. schlosseri*	*S. clava*	*C. robusta*	*O. dioica*
Length (Mbp)	533	340	115	64
No. of sequences	254	211	1,272	19
N50 (Mbp)	30	21	5	16
GC (%)	40.52	35.27	34.72	41.04
No. of annotated genes	22,275	19,966	16,406	17,259
BUSCO Complete (Single, Duplicated)	91.6% (90.7%, 0.9%)	90.5% (84.3%, 6.2%)	93.7% (93.1%, 0.6%)	59.4% (56.5%, 2.9%)
BUSCO Fragmented	3.1%	3.8%	2.3%	9.6%
BUSCO Missing	5.3%	5.7%	4.0%	31.0%

Table S3. Assembly statistics of the new collapsed assembly of *Botryllis schlosseri* compared to the existing chromosome-level reference assemblies of *Styela clava*, *Ciona robusta* and *Oikopleura dioica*.

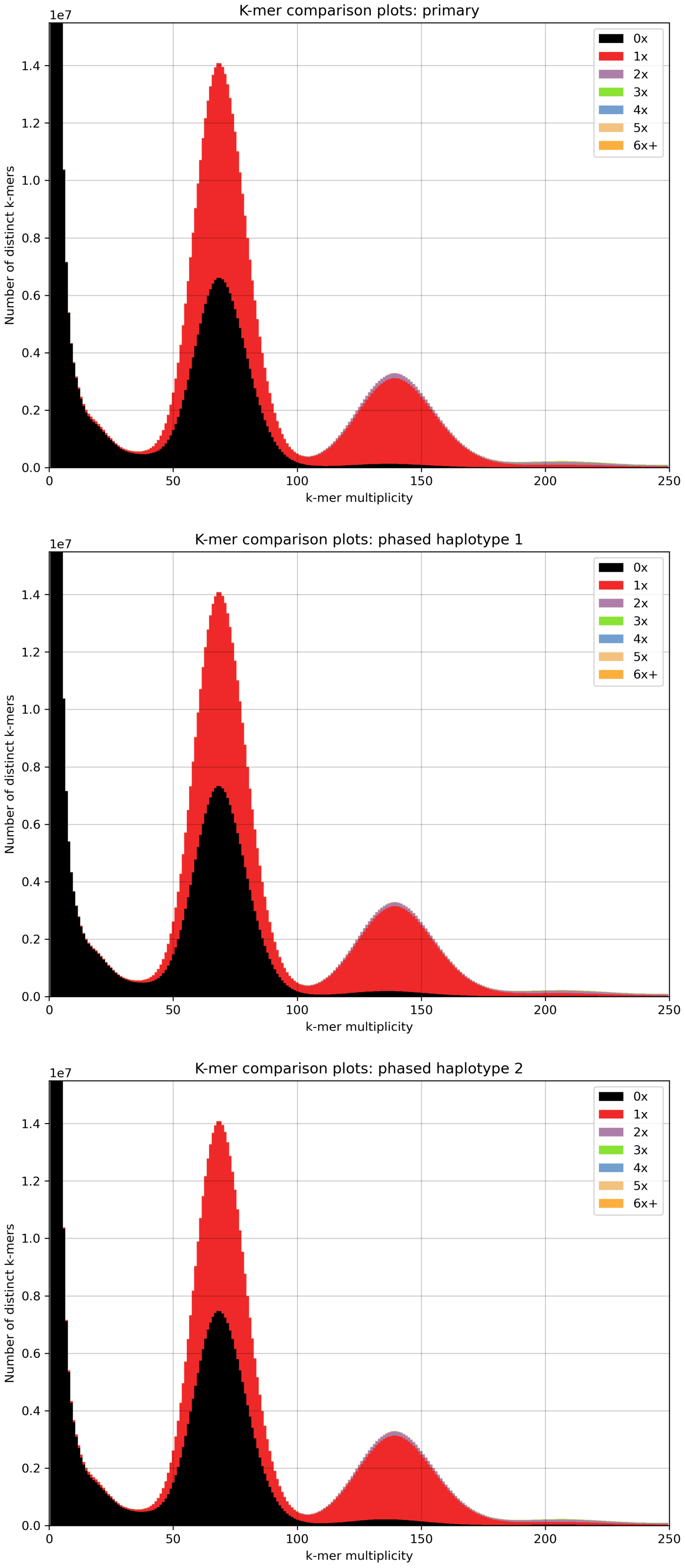

Figure S2. Output of the KAT comp tool comparing the k-mers found in the Illumina and HiFi reads to those present in the collapsed (top), haplotype 1 (middle) and haplotype 2 (bottom) assemblies of *B. schlosseri*. The k-mer completeness, based on the highest peak (corresponding here to heterozygous k-mers), is respectively (from top to bottom) 53.03%, 47.94%, and 46.92%. A perfectly correct haploid representation should have a k-mer completeness of 50%.

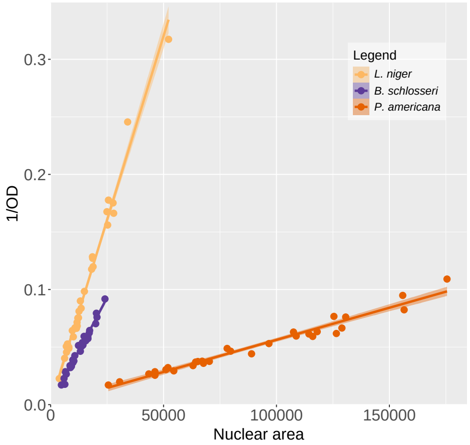

Figure S3. Linear regressions confirming that the total amount of DNA coloration per nucleus is constant for each species, regardless of nuclear size.

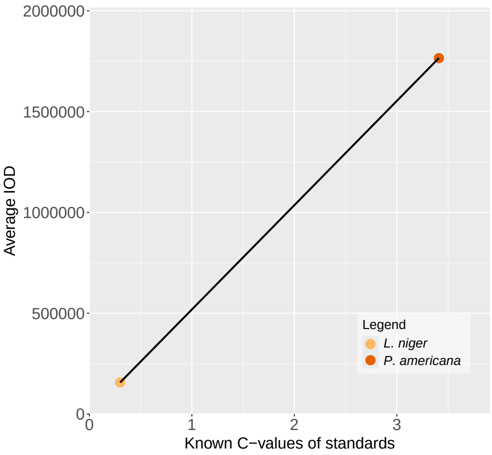

Figure S4. Linear regression confirming that the integrated optical density of each standard is proportional to its known C-value

.

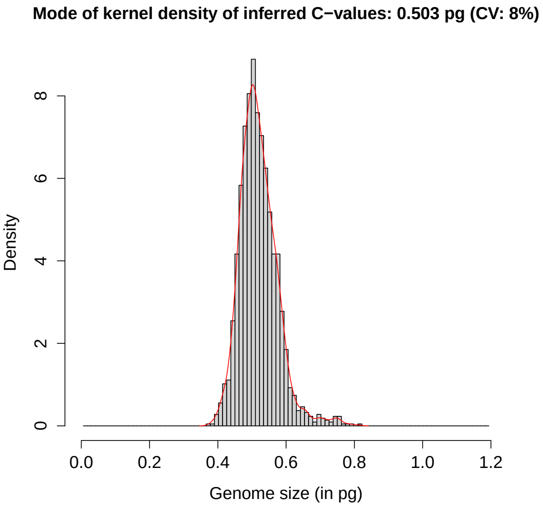

Figure S5. Genome size histogram of *Botryllus schlosseri* obtained using Feulgen microphotodensitometry.

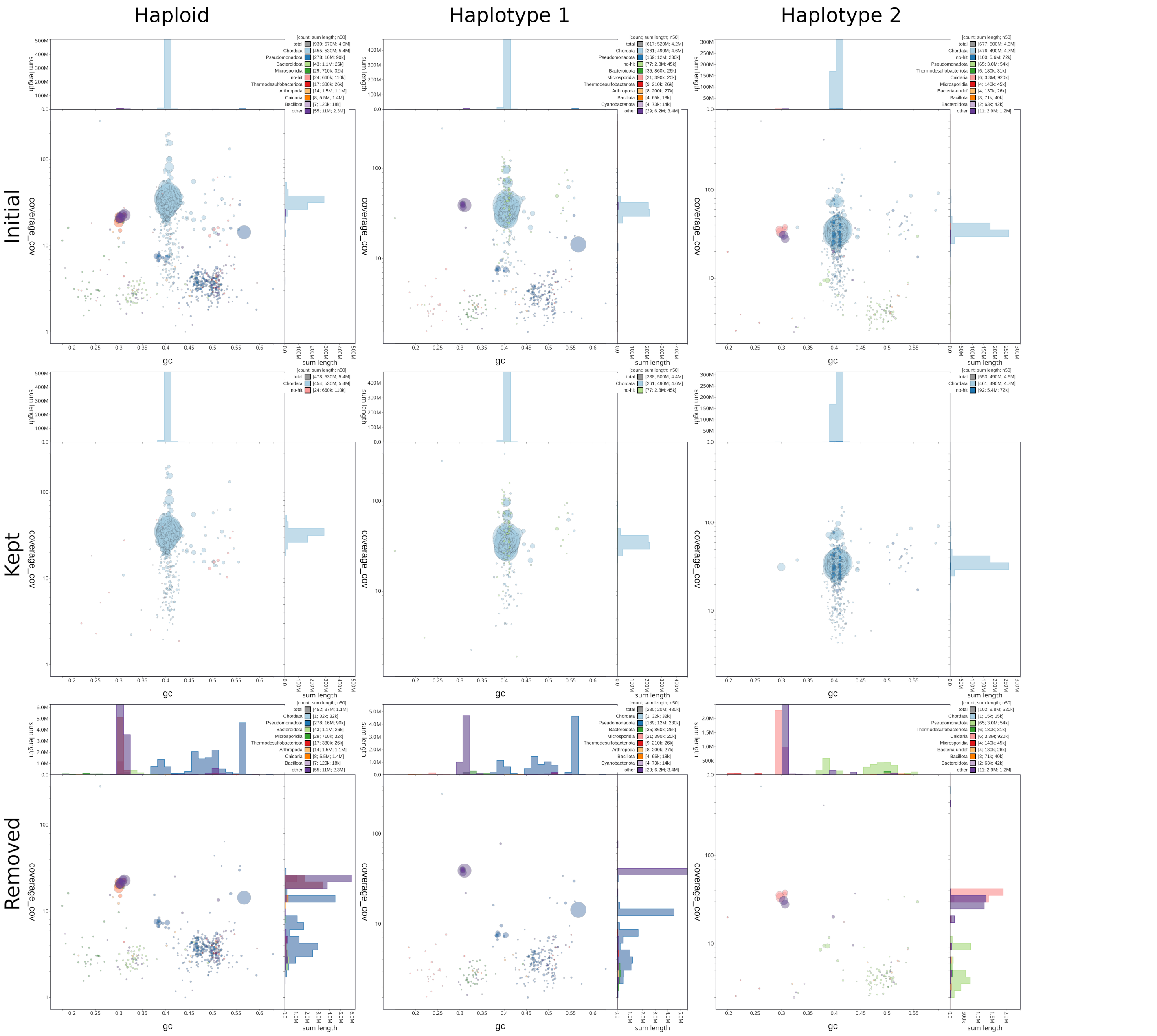

Figure S6. BlobPlots of the assemblies of *B. schlosseri*. **Initial** refers to results obtained before filtering out contamination. **Kept** represents the contigs retained in the assemblies before scaffolding, while **Removed** represents those discarded as contamination.

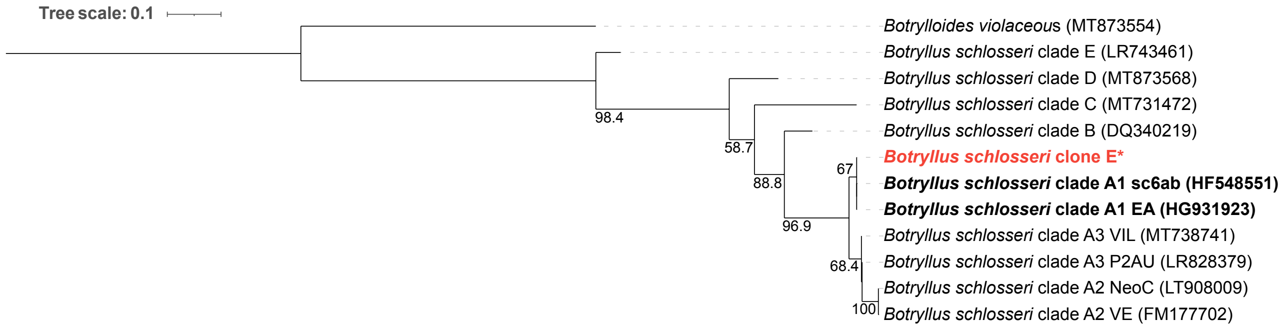

Figure S7. Maximum-likelihood tree of *Botryllus schlosseri* clades and sub-clades reconstructed from COI sequences [44]. Branches shows boostrap values. Accession ID are indicated between parenthesis.

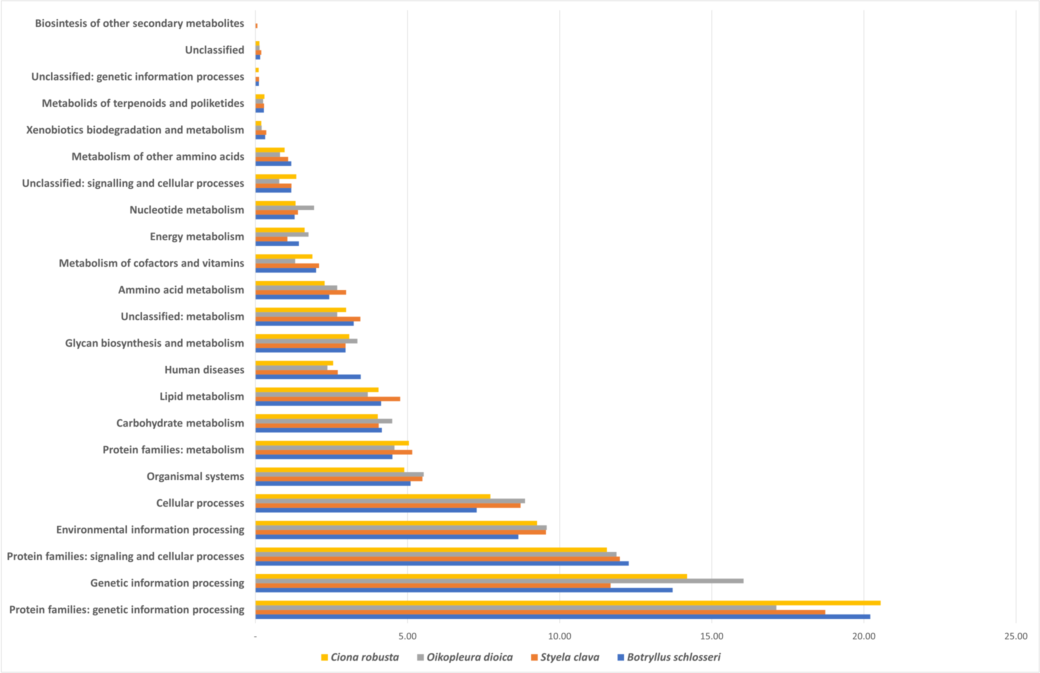

Figure S8. Comparison of the percentage of genes of *Botryllus schlosseri*, *Ciona robusta*, *Oikopleura dioica* and *Styela clava* assigned to different KEGG functional categories by BlastKOALA [55]

.

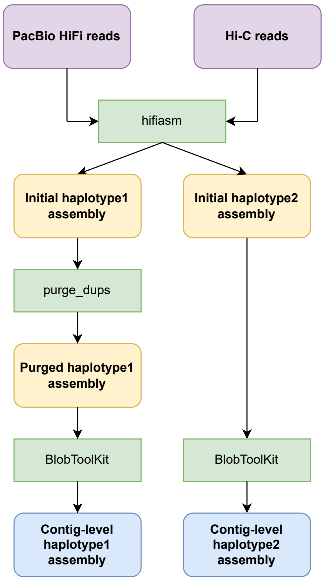

Figure S9. Assembly pipeline used to generate the contig-level assemblies of haplotype 1 and haplotype 2. The downstream steps (not shown) to produce scaffold-level assemblies are identical to those used for the collapsed assembly.

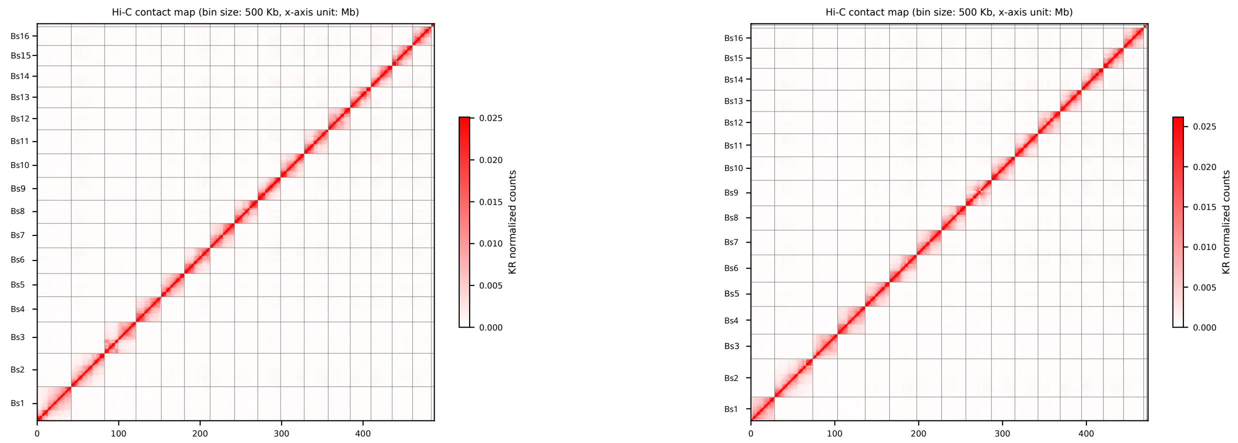

(a) Haplotype 1

(b) Haplotype 2

Figure S10. Hi-C heatmaps of the haplotype 1 (left) and haplotype 2 (right) assemblies, showing sixteen chromosome-scale scaffolds for both.

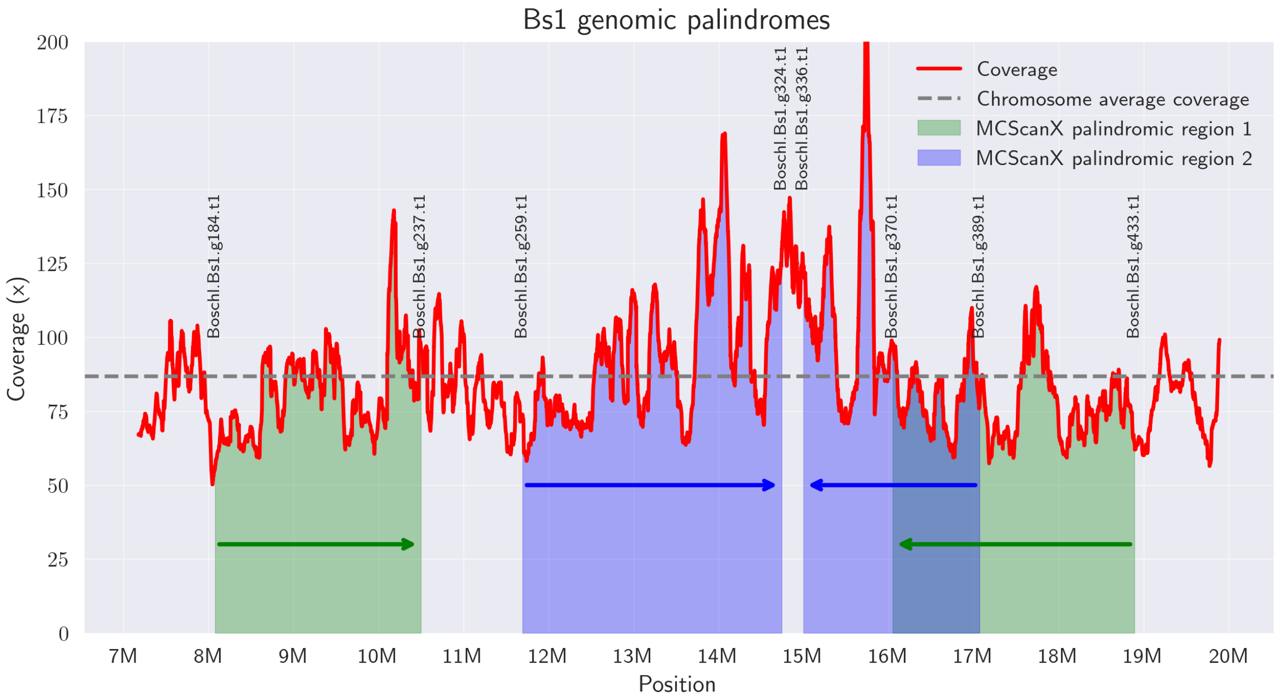

Figure S11. Representation of the two largest palindromic regions on the sequence Bs1, based on the syntenic blocks identified by MCScanX [38] (shown in green and purple). Coverage was calculated using ONT reads and the curve, which was smoothed using a rolling mean with a window size of 100,000 bp, does not show major deviations in the palindromic regions compared to the average coverage across the entire sequence (indicated by the dashed horizontal line). The gene names marking the start and end of each region are labeled. For example, the block extending from gene Boschl.Bs1.g184.t1 to Boschl.Bs1.g237.t1 (first green rightward arrow) is syntenic with the block from Boschl.Bs1.g370.t1 to Boschl.Bs1.g433.t1 (second green leftward arrow) in reverse order.

.

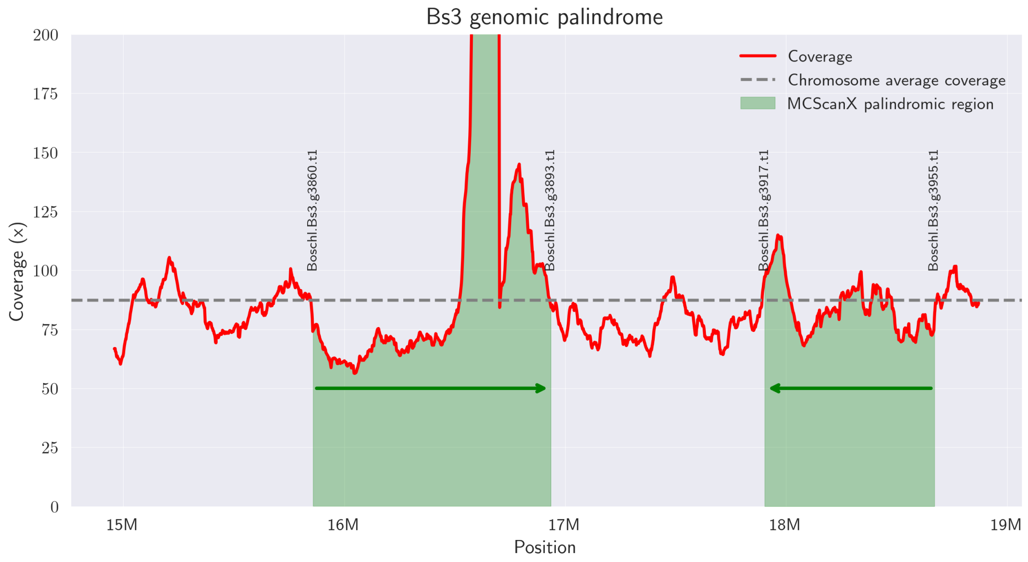

(a)

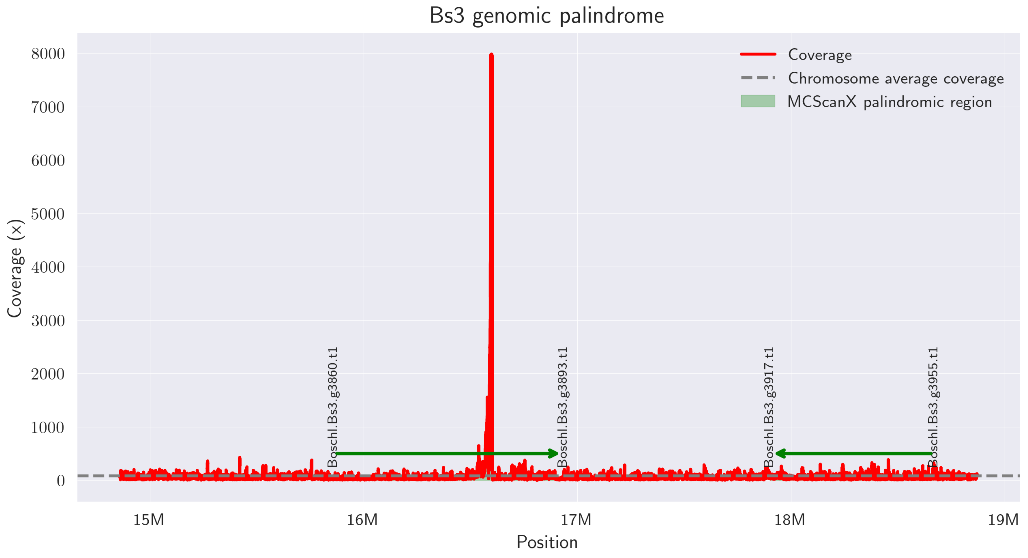

(b)

Figure S12. Representation of the large palindromic region on sequence Bs3. In **(a)**, it is plotted in the same manner as in Supplementary Figure S11. In **(b)**, the same data are shown without smoothing the coverage curve and without restricting the coverage scaling to 200×. The large peak around position 16.6 Mb corresponds to a region highly enriched in monomers likely to be centromeric repeats, and is located between two putative topologically associating domains (see Supplementary Figure S13).

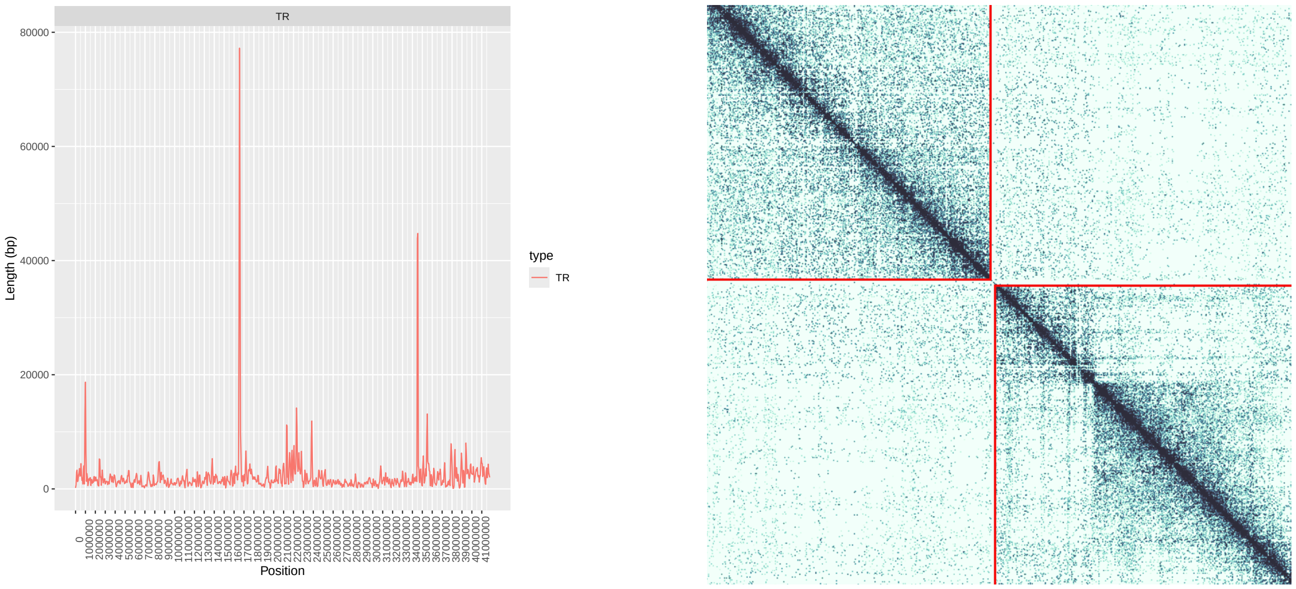

(a) **(b)**

Figure S13. (a) Tandem repeat region sizes along the sequence Bs3, based on monomers likely to be centromeric repeats and identified using quarTeT CentroMiner [123] on the collapsed assembly. A long repetitive region is observed between 16 and 17 Mb. **(b)** Zoom-in on the Hi-C heatmap of sequence Bs3, spanning from 12 Mb to 22 Mb and displayed with PretextView (RRID:SCR_022024, v0.2.5), where two putative topologically associating domains (TADs) have been manually highlighted with red lines. The gap between the two putative TADs extends approximately from 16.514 Mb to 16.595 Mb.

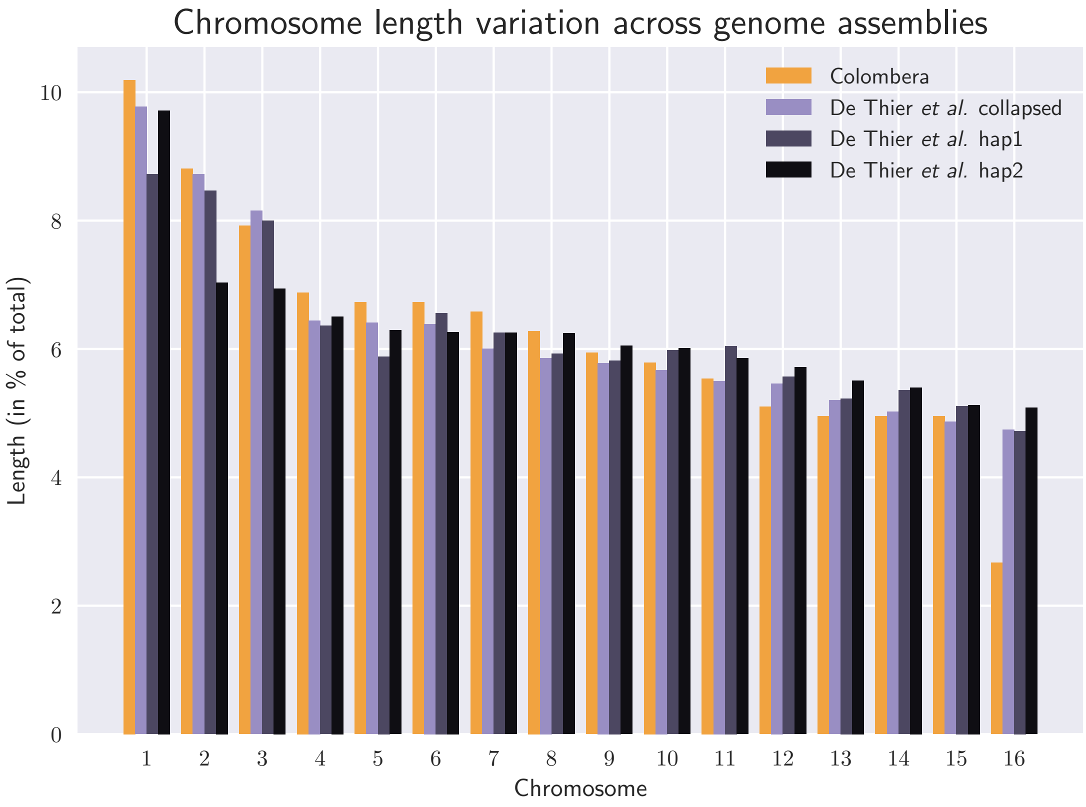

Figure S14. Comparisons between the 16 longest scaffolds from the collapsed, haplotype 1, and haplotype 2 assemblies and the karyogram of Colombera [35]. The lengths of the bars were calculated as the proportion (in percentage) of each chromosome's length relative to the total genome length. The order of scaffolds for haplotype 1 and haplotype 2 is based on the sizes of the scaffolds in descending order, rather than their alignment to the collapsed assembly.

.

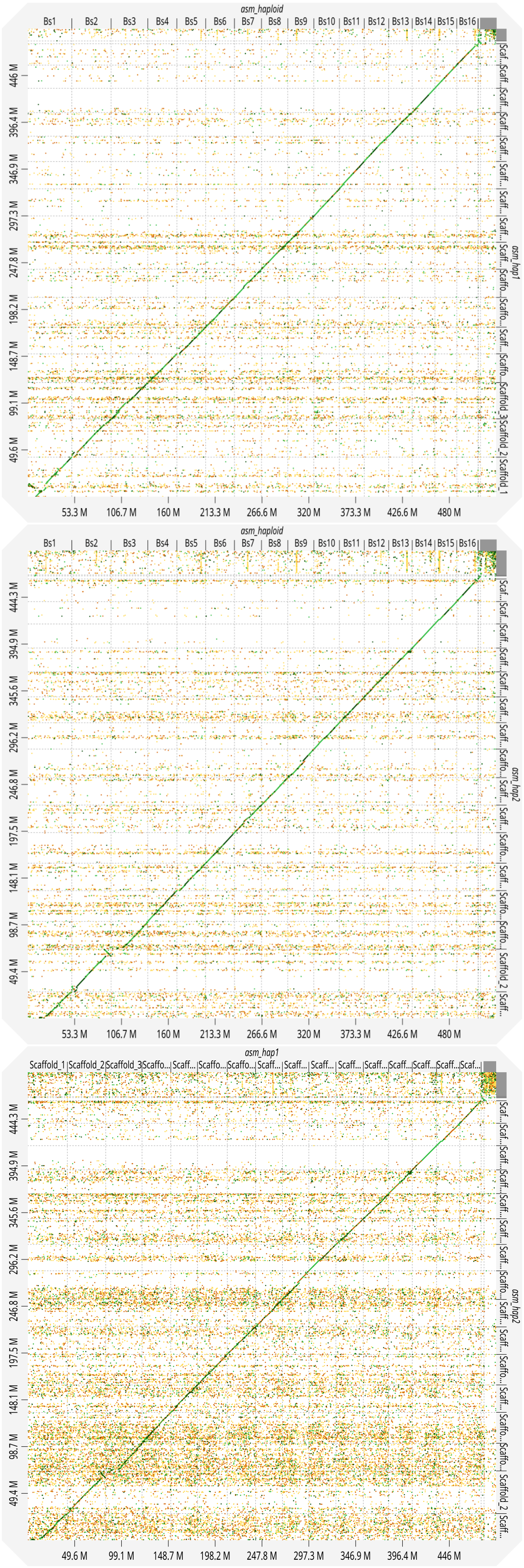

Figure S15. D-GENIES [124] dot plots of the final alignments: haplotype 1 vs. the collapsed assembly (top), haplotype 2 vs. the collapsed assembly (middle), and haplotype 1 vs. haplotype 2 (bottom). These were used to assess synteny and guide scaffold ordering.

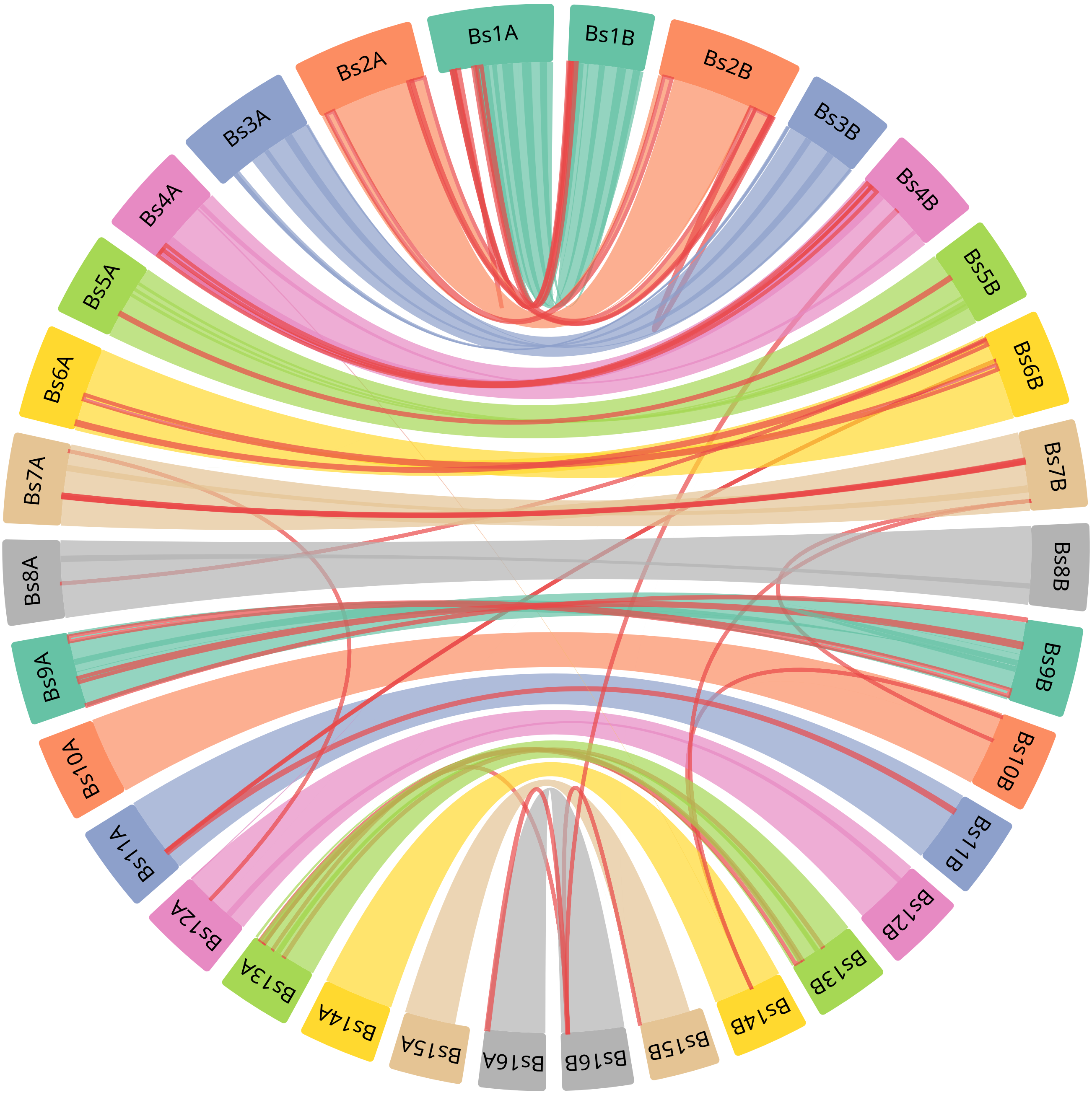

Figure S16. AccuSyn [37] representation of syntenic blocks identified using MCScanX [38] between the 16 largest scaffolds of haplotype 1 (left, with scaffold names ending in 'A') and haplotype 2 (right, with scaffold names ending in 'B') assemblies. Inverted blocks are highlighted in red.

.

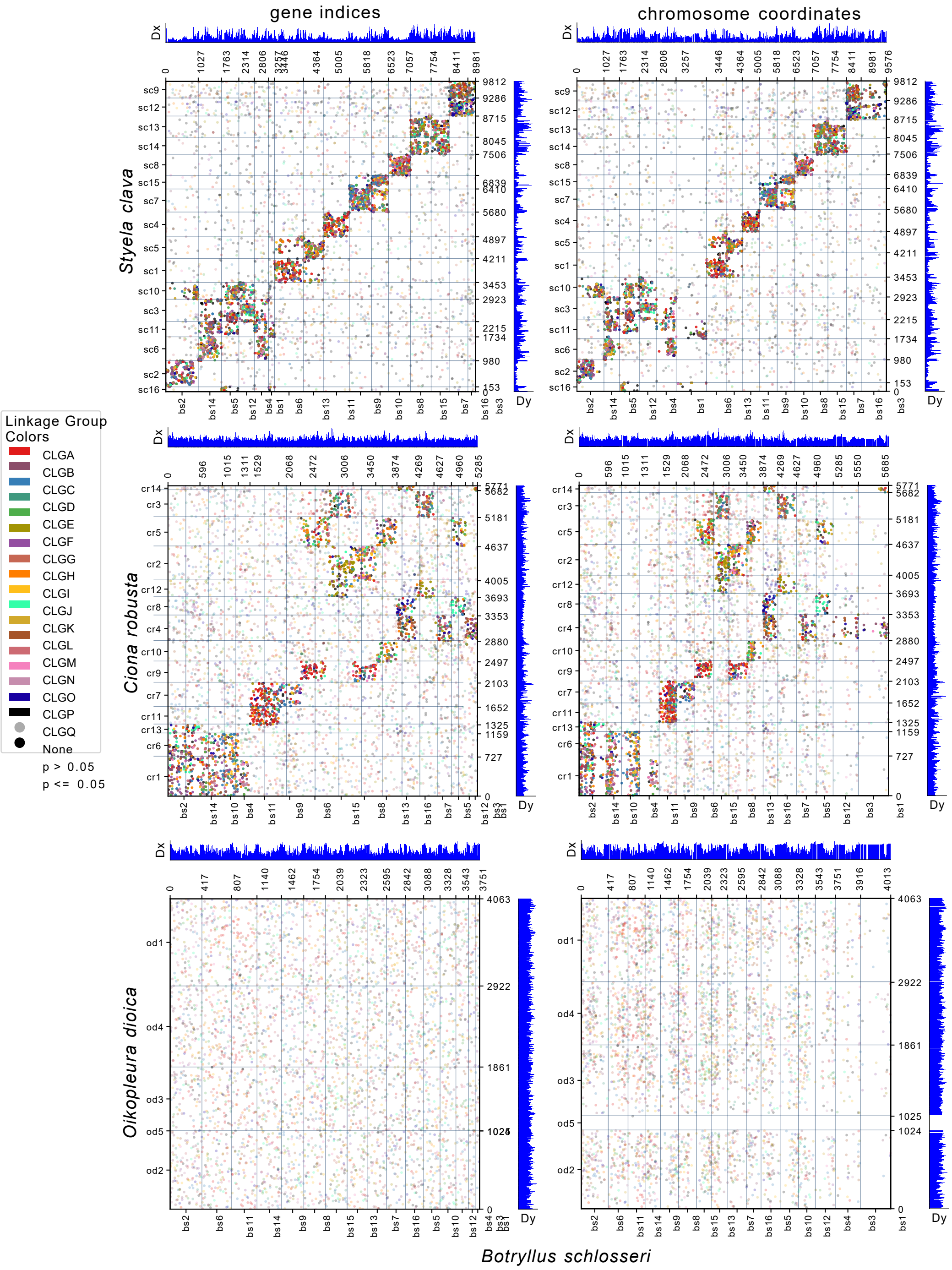

Figure S17. Investigation of synteny conservation among tunicate genomes. In the first column, dot plots depict the chromosome-scale scaffolds of *Botryllus schlosseri* (x-axis) plotted against those of *Styela clava*, *Ciona robusta* and *Oikopleura dioica* (y-axis). Each dot in the plot represents an ortholog, specifically a reciprocal best diamond blastp match between two species. The units of the x- and y-axes are the number of orthologous proteins: 9813, 5772 and 4064 orthologs found between the 16 chromosome-scale scaffolds of *B. schlosseri* and the 16 of *S. clava*, the 14 of *C. robusta* and the 5 of *O. dioica*, respectively. If there were chromosome breaks, Fisher's exact test (FET) was used to calculate the significance of the interactions between the sub-chromosomal pieces. Otherwise, FET was calculated on whole chromosomes. The opacity of the dots depicts the significance of FET. Dots that are a solid color are in cells with a FET p-value less than or equal to 0.05. Dots that are translucent are in cells with a FET p-value greater than 0.05. Dx and Dy values allow to pinpoint places where there may be sudden breaks in synteny [58] The second column of the figure depicts the same information as the first one, but plotted following chromosome base pair coordinates rather than gene index. This is better suited for visualizing gene-poor regions of the chromosomes.

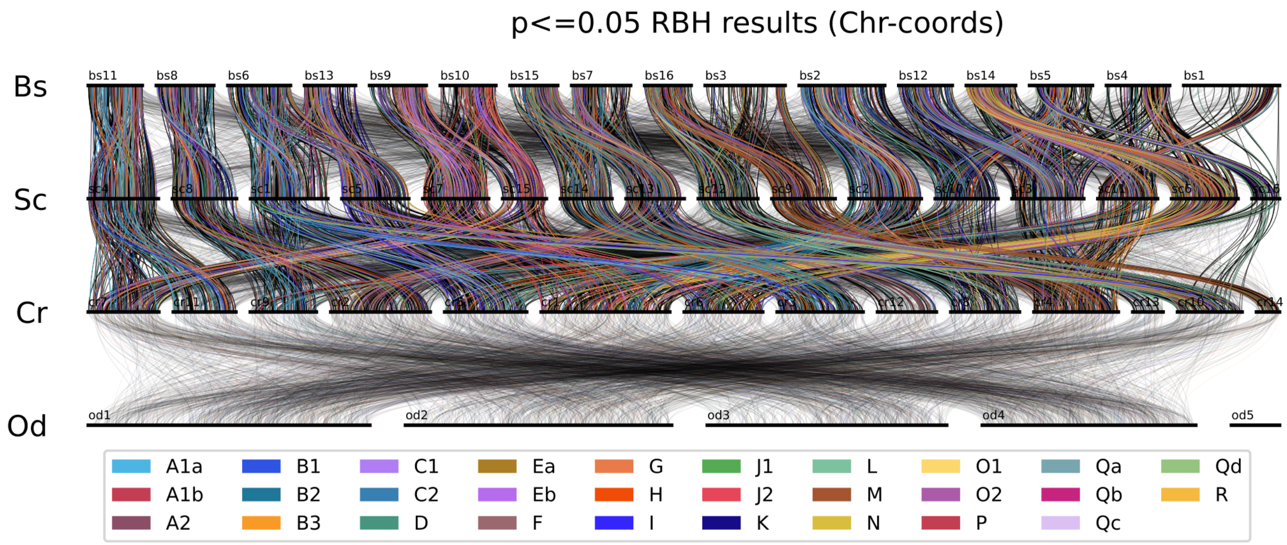

Figure S18. Synteny conservation of bilaterians, cnidarians and sponges linkage groups (BCnS LGs) between *Botryllus schlosseri* (Bs), *Styela clava* (Sc), *Ciona robusta* (Cr) and *Oikopleura dioica* (Od). For each species, the horizontal black lines represent the chromosomes, while the colored vertical lines connect conserved orthologs between species pairs. Each color corresponds to one of the 29 ancestral BCnS LGs identified in [60]. The opacity of the lines indicates the significance of the interaction between inter-species chromosomes, with solid colors representing significantly enriched conservation of synteny.

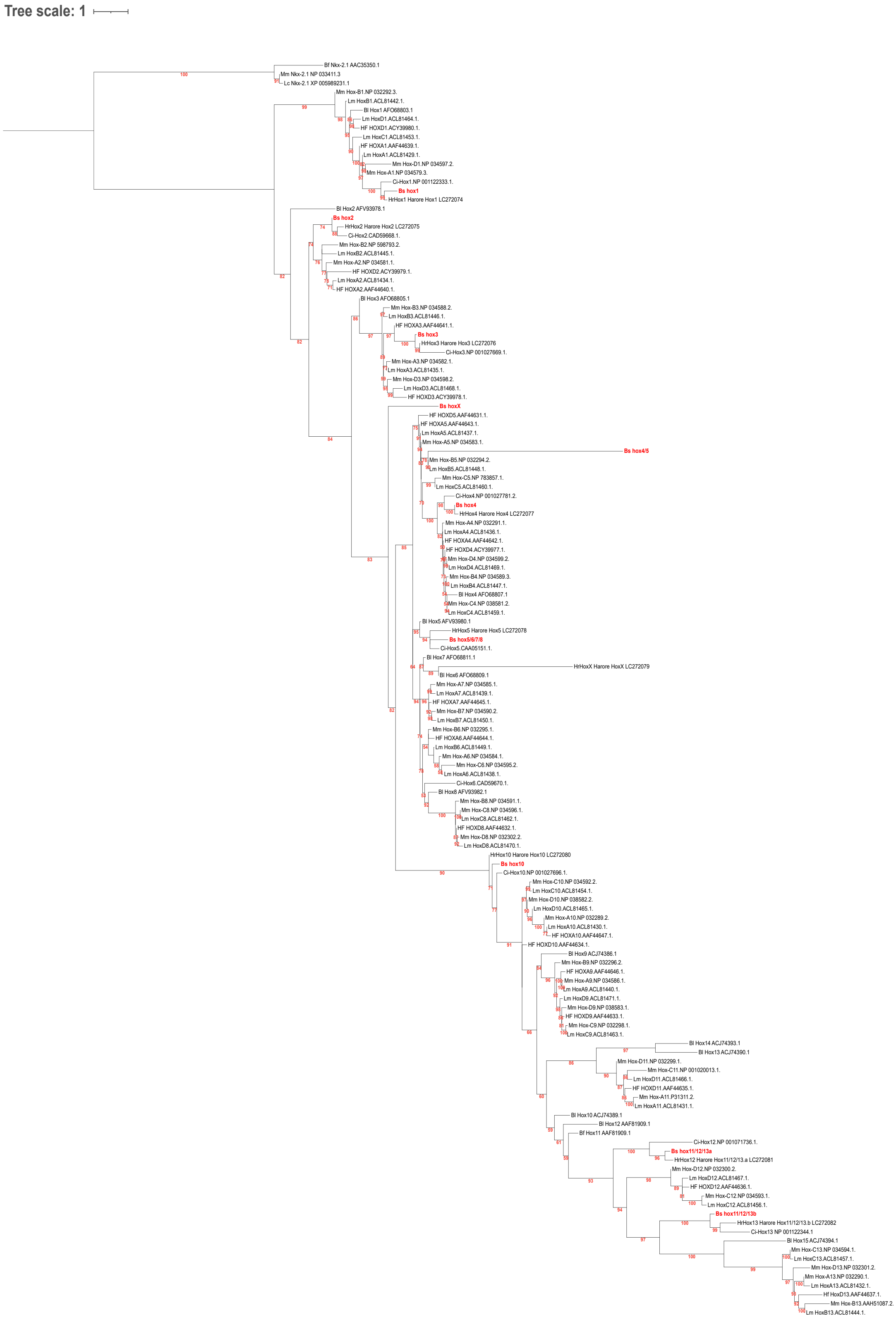

Figure S19. Phylogenetic analyses of Hox genes candidates of *Botryllus schlosseri*. The ML tree was generated using IQ-TREE 2 [115] by adding the *B. schlosseri* sequences to the alignment of Sekigami et al. 2017 [69] and keeping the homeodomains as well as the flanking 20 N-terminal and seven C-terminal aminoacids. Ultrafast bootstrap values are shown in red.

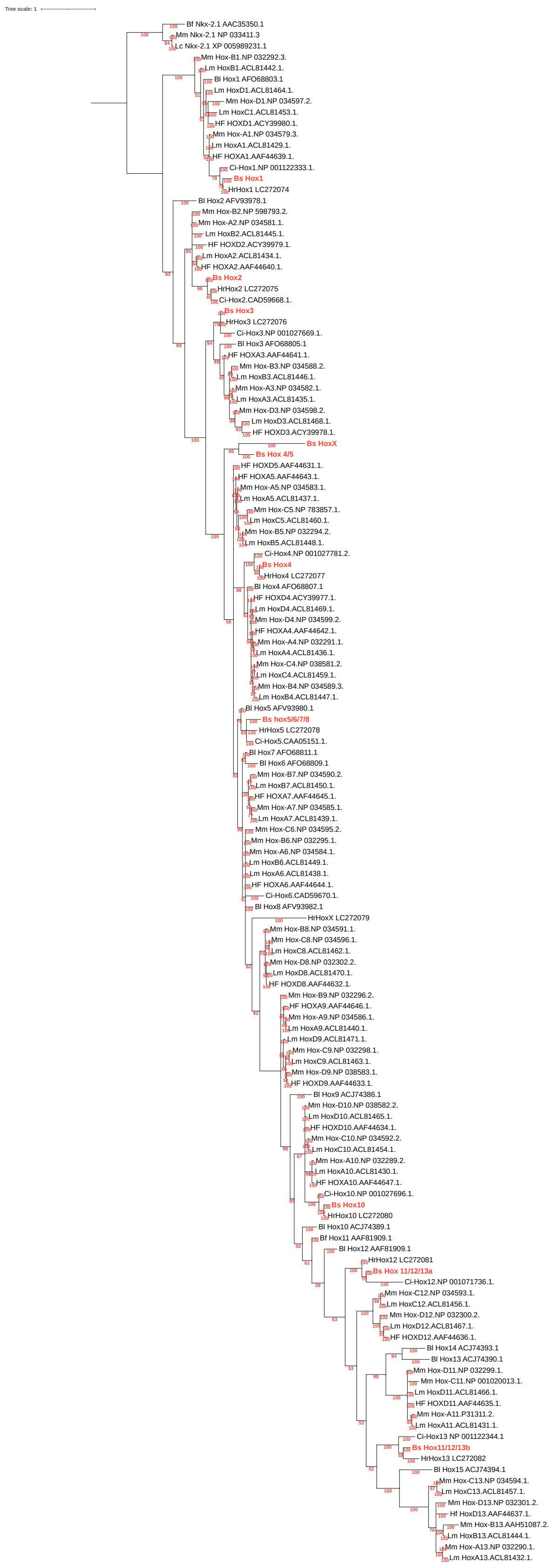

Figure S20. Phylogenetic analyses of Hox genes candidates of *Botryllus schlosseri*. The Bayesian tree was generated using MrBayes [125] by adding the *B. schlosseri* sequences to the alignment of Sekigami et al. 2017 [69] and keeping the homeodomains as well as the flanking 20 N-terminal and seven C-terminal aminoacids. Posterior probabilities are shown in red.

Supplementary File 1

Click here to access/download

Supplementary Material

Supp_file_1_bschlosseri_asm_sup_tables.xls

Supplementary File 2

Click here to access/download
Supplementary Material
Supp_file_2_chr_size.ods

BastKoala KEGG Bs (Supp. Fig. 8)

Click here to access/download
Supplementary Material
BlastKoala_Botryllus_schlosseri.txt

BastKoala KEGG Cr (Supp. Fig. 8)

Click here to access/download
Supplementary Material
BlastKoala_Ciona_robusta.txt

BastKoala KEGG Od (Supp. Fig. 8)

Click here to access/download
Supplementary Material
BlastKoala_Oikopleura_dioica.txt

BastKoala KEGG Sc (Supp. Fig. 8)

Click here to access/download
Supplementary Material
BlastKoala_styela_clava.txt

List cobionts (Supp. Fig. 6)

Click here to access/download
Supplementary Material
cobionts.tex
