# Supplementary material for: First chromosome-level genome assembly of the colonial chordate model Botryllus schlosseri (Tunicata)
Source: Gigascience. 2025 Sep 18;14:giaf097. doi: 10.1093/gigascience/giaf097 (PMC12448946; doi:10.1093/gigascience/giaf097)
Supplement: giaf097_Authors_Response_To_Reviewer_Comments_Original_Submission [file giaf097_authors_response_to_reviewer_comments_original_submission.pdf]

Author's Response To Reviewer Comments

Replies to reviewers' comments (see also attached file)

We sincerely appreciate the reviewers' insightful and constructive feedback. Below, we respond to each comment point by point, outlining the corresponding revisions and clarifications made to enhance the manuscript. In addition to addressing the reviewers' comments, we made a minor revision to the section on genome size estimation using Feulgen staining ; this is because a genome assembly of the Lasius niger standard we used is about to be published, indicating a genome size of 0.30 pg instead of the value of 0.32 pg assumed previously. We also added two extra figures to that part. Finally, we took the opportunity to correct a few typos and refine some imprecise definitions. For ease of review, all modifications in the manuscript main text are highlighted in red.

Reviewer 1
In this manuscript, De Thier and colleagues reported the chromosomal level genome assembly of tunicate Botryllus schlosseri (Pallas, 1766) sub-clade A1. The methods used in this study are standard. B. schlosseri has been used as laboratory model in certain places to understand asexual development and regeneration for decades. Despite there was a draft quality genome published a decade ago (eLife 2013, 2:e00569), the authors here produced a high-quality phased genome based on modern technologies. In terms of genomic resources for this laboratory model, this is important and useful. The authors have also carried out analyses, including repeats, synteny, and Hox cluster genes. I also think some of these results are interesting. Below are my comments and suggestions for the authors to consider which hopefully can further improve the manuscript.

1. Given the authors merged the results and discussion into one section, I would expect more discussion for several parts, including: - a. Repeats - For now, the analysis is quite standard and the main text is relatively descriptive. The question to me is what have we learnt from understanding the repeats from B. schlosseri genome? The authors should tell the readers. - b. Synteny analyses - This is an interesting finding. Extensive chromosomal rearrangement has also been discovered in other animals in recent. Can the authors further discuss these events? - c. Hox gene analyses - Again, it is quite descriptive. Tunicates are well known for dispersed Hox cluster for decades. So what have we learnt from the situation of B. schlosseri which I would be glad to see if the authors can discuss them.

Given the editors' decision to classify the manuscript as a 'Data Note' article due to the "lack of biological validation", we initially revised the discussion to align to this format, resulting in a more concise discussion. Nonetheless, we followed reviewer's suggestions and expanded the discussion to emphasize the significance of repetitive elements in B. schlosseri, particularly their potential role in genome plasticity and evolutionary adaptation. We also compared our findings to recent studies reporting chromosomal rearrangements in tunicates and other metazoans. Additionally, we provide further context on the organization of the Hox cluster in B. schlosseri, highlighting its implications in comparison to other tunicate species.

2. Figure S14 - The authors should also show the bootstrap values on the key nodes.

Bootstrap values have been added on the former Figure S14, now Figure S19

- In addition, the authors should also use one more method to construct the Hox gene tree in addition to Maximum Likelihood method.

A tree built using a Bayesian approach has been added as Figure S20

Reviewer 2
De Thier et al. present a high-quality chromosome scale de novo assembly of the tunicate Botryllus schlosseri from mainly PacBio HiFi and Arima Hi-C reads. Further WGS Illumina and ONT data was applied to resolve assembly errors or support the correctness of the assembly structure. Structural and functional annotations are conducted thoroughly. Downstream analyses include a synteny comparison of different Tunicata based on ancestral linkage groups and Hox genes.
The manuscript is well written and methods are mostly described to ensure reproducibility.
Despite the good shape of the manuscript, I would like to give some remarks, which should be addressed in a revised manuscript before publication.
General remarks
I like the quote in the beginning of the introduction.
The authors conducted downstream analyses with different related tunicate genome assemblies on chromosome level. For assembly metrics, there is a comparison regarding BUSCO assessment only. I would point out the high quality of the B. schlosseri assembly in Table 2 and 4 by comparison with the other chromosome level and annotated tunicate genome assemblies as well.

Table S3 has been added, comparing the metrics of B.schlosseri, Styela clava, Ciona robusta, and Oikopleura dioica.

I am not an expert regarding tunicates, so please excuse my basic, curiosity driven question: In the results section "The laboratory model Sub-clade A1" you state that a part of COI is used as a barcode to differentiate ascidian species. In the introduction you state that wild colonies are able to fuse resulting in mixed genotypes. Since sample E* derived from the wild at some point, it might be theoretically possible to have not only mixed nuclear genotypes but mixed mitotypes too. Depending on how old sample E* is and how fast fixation of a mitotype can happen within a colony, this might be reflected in your data. Furthermore, this thought could be expanded to nuclear genotypes, which could hamper scientific findings.

Clone E* is isogenic and, barring the unlikely accumulation of somatic mutations, possesses a single mitochondrial genome. We have revised the text to clarify our rationale for selecting clone E*

Contamination filtering was based on a sequence similarity search and taxonomic assignment of blobtools only. Despite blobtools/blobtoolkit was applied I was not able to find a blobplot in the supplemental files. I would like to encourage the authors to add blobplots before and after contamination filtering at least to the supplement. In my opinion, blobplots are most powerful when considering GC content and coverage in the first place - especially, when dealing with taxa, which are underrepresented in public databases. Therefore, using taxonomic assignment only for contamination filtering might generate false positives (e.g. conserved sequences across the tree of life with taxonomic assignment different than Chordata but with similar GC and coverage as the target) and false negatives (e.g. short sequences of the assembly, which couldn’t be assigned with different GC and coverage as the target).

The Blobplots for the collapsed as well as for each of the two haplotypes (before and after filtering) are in Figure S6

In the paragraphs "Results and Discussion" (Haplotype-resolved assembly) as well as in "Methods" (Haploid genome assembly) you use the term "haploid assembly" multiple times. I find this term misleading, since the genome is not haploid and the assembly represents both haplotypes at the same time. I assume that primary contigs from hifiasm were used to generate this assembly. Therefore, I would suggest to e.g. call this assembly "based on primary contigs", "non phased", "haplotype mixed" or "haplotype unresolved" (as opposite to "haplotype 2resolved").

The term "haploid assembly" has been replaced with "collapsed assembly" to prevent misinterpretation

(following the terminology advocated by Heng Li, https://lh3.github.io/2021/04/17/concepts-in-phased-assemblies).

Particular remarks
Results and Discussion
Sequencing and genome size estimation
Table 1 Please specify what "round 1" and "round 2" are referring to. Was one library sequenced twice or were two different libraries created and sequenced?

This information has been updated in the "Methods" section

Haploid genome assembly
“We identified 28 contigs that belong to spore-forming unicellular parasites of the microsporidia group [32]. This represents the first report of this fungal group in a tunicate species.” Is this identification based on blobtools taxonomic assignment? This is not described in the methods. Furthermore, can you rule out that identification or taxonomic assignment is false positive? If not you should tune down the second sentence and maybe discuss this.

The text has been modified accordingly

“We then performed Hi-C scaffolding using YaHS [34], which reduced the number of contigs to 256, before [...]” Technically, scaffolding with yahs can only increase the number of contigs because original (hifiasm) contigs are split because of the Hi-C signal (at least as long the option –no-contig-ec isn’t applied). I would substitute "contigs" with "sequences".

The text have been modified accordingly

“Finally, a manual curation was performed, resulting in an assembly made up of 16 major scaffolds [...]” Is there any previous study on the karyotype of B. schlosseri? If so, citing it here would strengthen your results. Otherwise, I would recommend to state the karyotypes or the number of chromosome scale scaffolds of other tunicates here and discuss, if your findings are in line.

Previous studies discussing B.schlosseri have been cited (Colombera 1969), discussed on page 4 and cited in Figure S14

Table 2 Please substitute "No. of scaffolds" with "No. of sequences". Please add the contig N50 values. As pointed out above, I would like to see a comparison to the other chromosome level tunicate genome assemblies here, instead of showing basically the same stats twice.

The text have been modified accordingly

“[. . .] highlighted the presence of two large-scale genomic palindromes located within Bs1 and a smaller one in Bs3 (Figure 3).” The figure shows the presence but maybe you can highlight them in the figure and the caption even more? “To find out whether these palindromes may result from assembly artifacts [40], we checked the localization of the duplicated BUSCO genes along the chromosomes and did another run of CRAQ [...]” You could support your findings by showing an even coverage distribution within the palindromes, which is similar to the coverage distribution of whole assembly. Either as a histogram or a zoomed in version of the read coverage across reference as in the outer layer of the circos plot could show this nicely.

The text have been modified to highlight them more. The sequencing depth profiles have been added as supplementary figures.

Methods
Sampling, DNA isolation, and sequencing
"HiFi PacBio long reads" Please provide more details on how PacBio libraries (was it actually

one library sequenced twice or two different libraries?) were created and sequenced. Were low or ultra-low protocols used? On which machine was sequencing conducted?

These information have been updated in the text

RNA-seq data
3Is downloading public data a method? In any case you should cite the original papers and provide a list of accession numbers (supplement) but I would remove this paragraph and add the information to the paragraph "Genome annotation", e.g. "Public available RNA-seq reads [23, 25, 8] were aligned to the soft-masked assemblies [...]"

The text have been modified accordingly

Data preprocessing
Depending on how the PacBio libraries were created and which PacBio machine was utilized for sequencing, you should state how HiFi calling was conducted (e.g. Sequel II) and how PCR adapter and duplicates were filtered out (e.g. ultra-low).

The information has been updated in the session "Methods"

Haploid genome assembly
“To this aim, contigs were aligned to the NCBI nucleotide database (accessed 2023 March 18) using BLAST+ [78]” Please state the version of BLAST+.

v2.13.0+, the text have been modified accordingly

“Finally, a BLASTN search for fragments of the mitochondrial genome among the contigs was performed using the published complete mitochondrial genome of B. schlosseri (RefSeq NC_021463.1) [28].”
Were the fragments filtered out based on the blast search? Please explain what was done in detail. Which hits were considered (e.g. cutoffs)? The mitochondrial genome of E* was assembled with NOVOPlasty, which is by the way not stated in the methods but in the results only. Was the assembled mt genome of E* added to the assembly, once the fragments were filtered out?

The mitochondrial genome assembly has been added in its own subsection in the session "Methods". BLAST cutoffs to identify the mtDNA fragments have been added, the mtDNA assembly and identified fragments were removed from the assemblies (the text have been modified accordingly).

Haplotype-resolved assembly
If I understand correctly, the rapid curation pipeline was applied but no dual-curation was conducted. When aiming for haplotype-resolved assemblies, I would recommend to apply this method, e.g. concatenating both haplotypes and creating a combined contact map of haplotype 1 and 2, which can be curated as usual, with the advantage of being able to exchange (parts of) sequences between the haplotypes. In some cases phasing from hifiasm is not correct and can be easily corrected with this approach.

The steps to generate the haplotype 1 and haplotype 2 assemblies were similar to those used for the unphased assembly, following the initial assembly and haplotype purging. Each haplotype assembly was treated individually thereafter. An attempt was made to scaffold both haplotypes together by concatenating them prior to scaffolding, but the results were less satisfactory:
• Only 30 major scaffolds were produced (instead of the expected 32), as 2 scaffolds contained merged sequences from both haplotype 1 and haplotype 2.
• After manual curation to confidently recover 2×16 major scaffolds, the total assemblies sizes were 439 Mbp and 444 Mbp, both smaller than the expected 500 Mbp, and also smaller than the size obtained by scaffolding the haplotypes individually (480 Mbp and 464 Mbp).

• Structural variations and discrepancies in putative chromosome lengths, relative to the B. schlosseri karyogram, were still observed. This suggests that concatenation did not resolve potential misassemblies in our case.

Reviewer 3
In this MS entitled ’First chromosome-level genome assembly of the colonial chordate model Botryllus schlosseri (Tunicata)’, Olivier De Thier and colleagues report the first chromosomescale assembly of this colonial ascidian specie, paying special attention to differences with previous published assemblies and importantly between haplotypes. The MS is very well written, very easy and pleasant to read. This provides data of great quality and very relevant not only for the ascidian/tunicate community, but to the field of genome structural evolution. I firmly recommend it for publication, although I think that the authors could discuss it in deeper detail. Specially, I miss for instance a more elaborate discussion of the results in our understanding of the similarities and differences between clades that have been published in the last years (I have not been able to find some relevant articles in this regard cited in the bibliography).

We have revised the text and included three bibliographical references to better clarify the distinctions between clades. A detailed discussion of their differences and geographic distribution, however, lies beyond the scope of this manuscript.

I also feel that a deeper analysis of the differences between haplotypes could be very interesting, unless they are artifactual effects of the assemblies. As mentioned below, unless this is part of a longer story for a different MS beyond the scope of this one, I encourage the authors to validate some of the differences they find between haplotypes, and try to correlate the structural variations, with differences in gene counts between haplotypes, and to explore whether these differences could be correlated with aspects of biological relevance. I miss, for instance, Venn diagrams with gene contents between previous assemblies, and the haplotypes/haploid genome here reported. In any case,

Since it remains uncertain which differences between haplotypes arise from assembly or phasing artifacts, and which reflect true biological variation, we chose not to expand this analysis further at this stage. A more comprehensive examination and characterization of haplotype differences will be addressed in a future study.

I firmly recommend this MS for publications, since most of my suggestions are not intended to interrogate the results of the MS, but to improve it, but I also understand that some may go beyond the scope of this MS.
Minor points: Introduction Page 1: "the basic body plan of adult tunicates is highly conserved across the entire subphylum [3]". This sentence, which could be OK for ascidians, probably provides a highly simplified vision of Tunicate adult morphologies, specially comparing the divergent morphologies of Thaliaceans and Appendicularians. Please, elaborate the sentence.

The main text has been modified

To understand the comparisons between the data of this MS and previously reported genomes, it seems crucial to understand well the meaning of the "clades and subclades". Please, include in the introduction (or where needed), how are defined those clades, which are their origins and biological/geographical differences, . . . and all the critical information that will specially help non-tunicate readers to understand the results.

The results-discussion section "The laboratory model Sub-clade A1" has been modified accordingly.

Results: The authors refer to the presence of large-scale genomic palindromes in Bs1 and Bs3. But it is unclear what are these structures. I suggest to please provide some more detailed explanation about the palindromic nature of these regions.

Additional details on the palindromic nature of these regions, along with their coverage, are now provided in Supplementary Figures S11, S12, and S13.

The data of haplotype-resolved assemblies is very interesting. I wonder if it is possible to 5somehow measure the amount of heterozygosity between haplotype 1 and 2, and those versus the previous versions of the genome, to better understand intra and inter-variation between subclades?

A rough estimation of heterozygosity between two haplotypes can be obtained by mapping one haplotype assembly onto the other, performing variant calling, and dividing the number of identified SNPs by the size of the smaller haplotype assembly. However, this mapping-based approach is likely to underestimate the true heterozygosity level, as it may miss divergent regions (such as structural variants between haplotypes or collapsed regions if we consider the unphased assembly). In contrast, a k-mer-based approach is more suitable and less biased for this task, although it requires high-quality reads and sufficient even sequencing coverage (at least 25x for Genomescope). For instance, we estimated a heterozygosity level of 1.28% by counting SNPs called after mapping haplotype 2 onto haplotype 1; 1.6% by calling SNPs from Illumina reads mapped to the unphased assembly; and 3.63% using a k-mer-based approach. Attempting to estimate the heterozygosity level from the previous genome assembly by Voskoboynik et al. is unlikely to yield an accurate value for comparison, as their assembly was generated using reads from different colonies (Sc6a-b and 356a) and involved different sequencing technologies, resulting in varying levels of coverage and evenness.

The differences of the size of some regions between Colombera and this study, and even between haplotypes 1 and 2, are very interesting. I would find more informative to merge the three graphs of Figure S9 into one single graph, so we can also easily compare the different in sizes of the haplotypes with the haploid.

The three graphs have been merged in a single figure in the text.

If some of those differences are actually due to deletions, that would deserve further analysis. If this analysis is not part of another ongoing project that will be published somewhere else, I suggest identifying with a dot-plot some of those differences, specially between haplotypes, and validate with long-reads crossing those regions whether some of the deletions are real or artifactual. Please, include the dotplot graph together with the two haplotypes in figure S10.

Former Figure S10 (now Figure S15) has been modified accordingly

In those cases that could be real, it would be very interesting what genes are gone, and if those are not placed somewhere else in the genome as result of translocations, or those genes are actually gone and could explain some of the differences reported in the gen count between haplotypes.

A more in-depth examination and description of haplotype differences will be addressed in a future study.

The authors mentioned the presence of multiple structural variations, although some of which could be artifactual of miss-assemblies. Interestingly, the plot of the synteny blocks between the two haplotypes in figure S11 shows some of those structural variations, including cases of:
- deletions: for instance, there are "blank" regions in Bs1A and Bs3A with no lines, which may reflect areas that are not present in the haplotype B. - duplications and translocations within chromosomes or between chromosomes of different haplotypes. Just looking to this plot, I wonder how the distribution of chromosomes between haplotypes is done. For instance, I see that Bs7B shares a duplicated synteny block with chromosomes Bs10B and Bs14B, but not with Bs10A and Bs10B, which means that the duplications are intra-haplotype present in B but not in A. But I wonder if it is possible that Bs10B and Bs14B could be in fact switched to

haplotype A, and therefore there would be no duplication nor deletion in one of the haplotypes, just a simple translocation. I may be wrong in the interpretation, but I'm curious to understand the graph.

The interpretation of the structural variations from the graph seems correct. However, without sequencing reads from the two parental genomes of clone E*, which are unfortunately now untraceable, and without using techniques such as trio binning (https://doi.org/10.1038/nbt.
64277), we cannot accurately separate chromosomes into haplotype-specific sets corresponding to each parent. As a result, haplotypes A and B consist of arbitrarily selected chromosomes from both parents, and we cannot verify whether translocations between haplotypes should be favored over duplications within a haplotype as the more likely explanation.

In any case, again, as mentioned above, it would be worthy to validate some of those variations with long reads, which could illuminate the biological relevance between the haplotypes and discard potential artifactual errors of the assemblies.
I notice that in figures 7 and S13, some lines are thicker than others. Is this because many "thin" lines are overlapped, and they look like a "thick" line. Otherwise, the visual effect of different thicknesses could be misleading. Please, clarify.

The "thick" lines correspond to significant interactions between species, a more detailed explanation has been added in the figure captions.

In the analysis of the Hox cluster the authors say "[. . .] our new assembly revealed that B. schlosseri's Hox genes are not scattered. Instead, eight of them were clustered on the second largest scaffold (Bs2), whereas two other ones are found on the 15th largest scaffold (Bs15)." Generally, the description of the Hox gene in a cluster refers to the fact they are in the vicinity, with near not many other genes in between Hox genes. Therefore, I would not describe that eight Hox genes are clustered by the simple fact that they are in the same chromosome (maybe even in different arms).

We agree with the Reviewer correction. The text has been modified accordingly

Editorial Manager®

Cookie Preference Center

We use cookies which are necessary to make our site work. We may also use additional cookies to analyze, improve and personalize our content and your digital experience. For more information, see our Cookie Policy.

You may choose not to allow some types of cookies. However, blocking some types may impact your experience of our site and the services we are able to offer. See the different category headings below to find out more or change your settings.

Allow all

Manage Consent Preferences

Strictly Necessary Cookies

Always active

These cookies are necessary for the website to function and cannot be switched off in our systems. They are usually only set in response to actions made by you which amount to a request for services, such as setting your privacy preferences, logging in or filling in forms. You can set your browser to block or alert you about these cookies, but some parts of the site will not then work.

Functional Cookies

These cookies enable the website to provide enhanced functionality and personalisation. They may be set by us or by third party providers whose services we have added to our pages. If you do not allow these cookies then some or all of these services may not function properly.

Performance Cookies

These cookies allow us to count visits and traffic sources so we can measure and improve the performance of our site. They help us to know which pages are the most and least popular and see how visitors move around the site.

Targeting Cookies

These cookies may be set through our site by our advertising partners. They may be used by those companies to build a profile of your interests and show you relevant adverts on other sites. If you do not allow these cookies, you will experience less targeted advertising.

Cookie List

Clear

Apply Cancel

Consent Leg.Interest

Confirm my choices

Powered by onetrust
