# Supplementary material for: First chromosome-level genome assembly of the colonial chordate model Botryllus schlosseri (Tunicata)
Source: Gigascience. 2025 Sep 18;14:giaf097. doi: 10.1093/gigascience/giaf097 (PMC12448946; doi:10.1093/gigascience/giaf097)
Supplement: giaf097_Reviewer_1_Report_Original_Submission — Jerome Hui -- 3/26/2025 [file giaf097_reviewer_1_report_original_submission.pdf]

**Reviewer Report**

**Title: First chromosome-level genome assembly of the colonial chordate model Botryllus schlosseri (Tunicata)**

**Version: Original Submission** **Date:** 3/26/2025

**Reviewer name: Jerome Hui**

**Reviewer Comments to Author:**

In this manuscript, De Thier and colleagues reported the chromosomal level genome assembly of tunicate Botryllus schlosseri (Pallas, 1766) sub-clade A1. The methods used in this study are standard. B. schlosseri has been used as laboratory model in certain places to understand asexual development and regeneration for decades. Despite there was a draft quality genome published a decade ago (eLife 2013, 2:e00569), the authors here produced a high-quality phased genome based on modern technologies. In terms of genomic resources for this laboratory model, this is important and useful. The authors have also carried out analyses, including repeats, synteny, and Hox cluster genes. I also think some of these results are interesting. Below are my comments and suggestions for the authors to consider which hopefully can further improve the manuscript.1. Given the authors merged the results and discussion into one section, I would expect more discussion for several parts, including:- a. Repeats - For now, the analysis is quite standard and the main text is relatively descriptive. The question to me is what have we learnt from understanding the repeats from B. schlosseri genome? The authors should tell the readers.- b. Synteny analyses - This is an interesting finding. Extensive chromosomal rearrangement has also been discovered in other animals in recent. Can the authors further discuss these events?- c. Hox gene analyses - Again, it is quite descriptive. Tunicates are well known for dispersed Hox cluster for decades. So what have we learnt from the situation of B. schlosseri which I would be glad to see if the authors can discuss them.2. Figure S14- The authors should also show the bootstrap values on the key nodes.- In addition, the authors should also use one more method to construct the Hox gene tree in addition to Maximum Likelihood method.

**Level of Interest**

Please indicate how interesting you found the manuscript: Choose an item.

**Quality of Written English**

Please indicate the quality of language in the manuscript: Choose an item.

**Declaration of Competing Interests**

Please complete a declaration of competing interests, considering the following questions:

- Have you in the past five years received reimbursements, fees, funding, or salary from an organisation that may in any way gain or lose financially from the publication of this manuscript, either now or in the future?
- Do you hold any stocks or shares in an organisation that may in any way gain or lose financially from the publication of this manuscript, either now or in the future?
- Do you hold or are you currently applying for any patents relating to the content of the manuscript?
- Have you received reimbursements, fees, funding, or salary from an organization that holds or has applied for patents relating to the content of the manuscript?
- Do you have any other financial competing interests?
- Do you have any non-financial competing interests in relation to this paper?

If you can answer no to all of the above, write 'I declare that I have no competing interests' below. If your reply is yes to any, please give details below.

I declare that I have no competing interests

I agree to the open peer review policy of the journal. I understand that my name will be included on my report to the authors and, if the manuscript is accepted for publication, my named report including any attachments I upload will be posted on the website along with the authors' responses. I agree for my report to be made available under an Open Access Creative Commons CC-BY license (http://creativecommons.org/licenses/by/4.0/). I understand that any comments which I do not wish to be included in my named report can be included as confidential comments to the editors, which will not be published.

Choose an item.

To further support our reviewers, we have joined with Publons, where you can gain additional credit to further highlight your hard work (see: https://publons.com/journal/530/gigascience). On publication of this paper, your review will be automatically added to Publons, you can then choose whether or not to claim your Publons credit. I understand this statement.

Yes Choose an item.
