# Supplementary material for: First chromosome-level genome assembly of the colonial chordate model Botryllus schlosseri (Tunicata)
Source: Gigascience. 2025 Sep 18;14:giaf097. doi: 10.1093/gigascience/giaf097 (PMC12448946; doi:10.1093/gigascience/giaf097)
Supplement: giaf097_Reviewer_2_Report_Original_Submission — Tilman Schell -- 3/26/2025 [file giaf097_reviewer_2_report_original_submission.pdf]

Reviewer Report

Title: First chromosome-level genome assembly of the colonial chordate model Botryllus schlosseri (Tunicata)

Version: Original Submission **Date:** 3/26/2025

Reviewer name: Tilman Schell

Reviewer Comments to Author:

Review ofFirst chromosome-level genome assembly of the colonial chordate model Botryllus schlosseri (Tunicata)fromOlivier De Thier, Marie Lebel, Mohammed M. Tawfeeq, Roland Faure, Philippe Dru, Simon Blanchoud, Alexandre AliÃ©, Federico D. Brown, Jean-FranÃ§ois Flot and Stefano TiozzoComments to the authorsDe Thier et al. present a high-quality chromosome scale de novo assembly of the tunicate Botryllus schlosseri from mainly PacBio HiFi and Arima Hi-C reads. Further WGS Illumina and ONT data was applied to resolve assembly errors or support the correctness of the assembly structure. Structural and functional annotations are conducted thoroughly. Downstream analyses include a synteny comparison of different Tunicata based on ancestral linkage groups and Hox genes.The manuscript is well written and methods are mostly described to ensure reproducibility. Despite the good shape of the manuscript, I would like to give some remarks, which should be addressed in a revised manuscript before publication.General remarksI like the quote in the beginning of the introduction.The authors conducted downstream analyses with different related tunicate genome assemblies on chromosome level. For assembly metrics, there is a comparison regarding BUSCO assessment only. I would point out the high quality of the B. schlosseri assembly in Table 2 and 4 by comparison with the other chromosome level and annotated tunicate genome assemblies as well.I am not an expert regarding tunicates, so please excuse my basic, curiosity driven question: In the results section "The laboratory model Sub-clade A1" you state that a part of COI is used as a barcode to differentiate ascidian species. In the introduction you state that wild colonies are able to fuse resulting in mixed genotypes. Since sample E* derived from the wild at some point, it might be theoretically possible to have not only mixed nuclear genotypes but mixed mitotypes too. Depending on how old sample E* is and how fast fixation of a mitotype can happen within a colony, this might be reflected in your data. Furthermore, this thought could be expanded to nuclear genotypes, which could hamper scientific findings.Contamination filtering was based on a sequence similarity search and taxonomic assignment of blobtools only. Despite blobtools/blobtoolkit was applied I was not able to find a blobplot in the supplemental files. I would like to encourage the authors to add blobplots before and after contamination filtering at least to the supplement. In my opinion, blobplots are most powerful when considering GC content and coverage in the first place - especially, when dealing with taxa, which are underrepresented in public databases. Therefore, using taxonomic assignment only for contamination filtering might generate false positives (e.g. conserved sequences across the tree of life with taxonomic assignment different than Chordata but with similar GC and coverage as the target) and false negatives (e.g. short sequences of the assembly, which couldn't be assigned with different GC and coverage as the target).In the paragraphs "Results and Discussion" (Haplotype-resolved assembly) as well as in "Methods" (Haploid genome assembly) you use

the term "haploid assembly" multiple times. I find this term misleading, since the genome is not haploid and the assembly represents both haplotypes at the same time. I assume that primary contigs from hifiasm were used to generate this assembly. Therefore, I would suggest to e.g. call this assembly "based on primary contigs", "non phased", "haplotype mixed" or "haplotype unresolved" (as opposite to "haplotype resolved").Particular remarksResults and DiscussionSequencing and genome size estimationTable 1Please specify what "round 1" and "round 2" are referring to. Was one library sequenced twice or were two different libraries created and sequenced?Haploid genome assembly"We identified 28 contigs that belong to spore-forming unicellular parasites of the microsporidia group [32]. This represents the first report of this fungal group in a tunicate species."Is this identification based on blobtools taxonomic assignment? This is not described in the methods.Furthermore, can you rule out that identification or taxonomic assignment is false positive? If not you should tune down the second sentence and maybe discuss this."We then performed Hi-C scaffolding using YaHS [34], which reduced the number of contigs to 256, before [...]"Technically, scaffolding with yahs can only increase the number of contigs because original (hifiasm) contigs are split because of the Hi-C signal (at least as long the option --no-contig-ec isn't applied). I would substitute "contigs" with "sequences"."Finally, a manual curation was performed, resulting in an assembly made up of 16 major scaffolds [...]"Is there any previous study on the karyotype of B. schlosseri? If so, citing it here would strengthen your results. Otherwise, I would recommend to state the karyotypes or the number of chromosome scale scaffolds of other tunicates here and discuss, if your findings are in line.Table 2Please substitute "No. of scaffolds" with "No. of sequences".Please add the contig N50 values.As pointed out above, I would like to see a comparison to the other chromosome level tunicate genome assemblies here, instead of showing basically the same stats twice."[…] highlighted the presence of two large-scale genomic palindromes located within Bs1 and a smaller one in Bs3 (Figure 3)."The figure shows the presence but maybe you can highlight them in the figure and the caption even more?"To find out whether these palindromes may result from assembly artifacts [40], we checked the localization of the duplicated BUSCO genes along the chromosomes and did another run of CRAQ [...]"You could support your findings by showing an even coverage distribution within the palindromes, which is similar to the coverage distribution of whole assembly. Either as a histogram or a zoomed in version of the read coverage across reference as in the outer layer of the circos plot could show this nicely.MethodsSampling, DNA isolation, and sequencing"HiFi PacBio long reads"Please provide more details on how PacBio libraries (was it actually one library sequenced twice or two different libraries?) were created and sequenced. Were low or ultra-low protocols used? On which machine was sequencing conducted?RNA-seq dataIs downloading public data a method? In any case you should cite the original papers and provide a list of accession numbers (supplement) but I would remove this paragraph and add the information to the paragraph "Genome annotation", e.g. "Public available RNA-seq reads [23, 25, 8] were aligned to the soft-masked assemblies [...]"Data preprocessingDepending on how the PacBio libraries were created and which PacBio machine was utilized for sequencing, you should state how HiFi calling was conducted (e.g. Sequel II) and how PCR adapter and duplicates were filtered out (e.g. ultra-low).Haploid genome assembly"To this aim, contigs were aligned to the NCBI nucleotide database (accessed 2023 March 18) using BLAST+ [78]"Please state the version of BLAST+."Finally, a BLASTN search for fragments of the mitochondrial genome among the contigs was performed using the published complete mitochondrial genome of B. schlosseri (RefSeq NC_021463.1) [28]."Were the fragments filtered out based on the blast search?

Please explain what was done in detail. Which hits were considered (e.g. cutoffs)? The mitochondrial genome of E* was assembled with NOVOPlasty, which is by the way not stated in the methods but in the results only. Was the assembled mt genome of E* added to the assembly, once the fragments were filtered out?Haplotype-resolved assemblyIf I understand correctly, the rapid curation pipeline was applied but no dual-curation was conducted. When aiming for haplotype-resolved assemblies, I would recommend to apply this method, e.g. concatenating both haplotypes and creating a combined contact map of haplotype 1 and 2, which can be curated as usual, with the advantage of being able to exchange (parts of) sequences between the haplotypes. In some cases phasing from hifiasm is not correct and can be easily corrected with this approach.

Level of Interest

Please indicate how interesting you found the manuscript: Choose an item.

Quality of Written English

Please indicate the quality of language in the manuscript: Choose an item.

Declaration of Competing Interests

Please complete a declaration of competing interests, considering the following questions:

- Have you in the past five years received reimbursements, fees, funding, or salary from an organisation that may in any way gain or lose financially from the publication of this manuscript, either now or in the future?
- Do you hold any stocks or shares in an organisation that may in any way gain or lose financially from the publication of this manuscript, either now or in the future?
- Do you hold or are you currently applying for any patents relating to the content of the manuscript?
- Have you received reimbursements, fees, funding, or salary from an organization that holds or has applied for patents relating to the content of the manuscript?
- Do you have any other financial competing interests?
- Do you have any non-financial competing interests in relation to this paper?

If you can answer no to all of the above, write 'I declare that I have no competing interests' below. If your reply is yes to any, please give details below.

I declare that I have no competing interests

I agree to the open peer review policy of the journal. I understand that my name will be included on my report to the authors and, if the manuscript is accepted for publication, my named report including any attachments I upload will be posted on the website along with the authors' responses. I agree for my

report to be made available under an Open Access Creative Commons CC-BY license (http://creativecommons.org/licenses/by/4.0/). I understand that any comments which I do not wish to be included in my named report can be included as confidential comments to the editors, which will not be published.

Choose an item.

To further support our reviewers, we have joined with Publons, where you can gain additional credit to further highlight your hard work (see: https://publons.com/journal/530/gigascience). On publication of this paper, your review will be automatically added to Publons, you can then choose whether or not to claim your Publons credit. I understand this statement.

Yes Choose an item.
