# Supplementary material for: First chromosome-level genome assembly of the colonial chordate model Botryllus schlosseri (Tunicata)
Source: Gigascience. 2025 Sep 18;14:giaf097. doi: 10.1093/gigascience/giaf097 (PMC12448946; doi:10.1093/gigascience/giaf097)
Supplement: giaf097_Reviewer_3_Report_Original_Submission — Cristian Canestro -- 4/2/2025 [file giaf097_reviewer_3_report_original_submission.pdf]

Reviewer Report

Title: First chromosome-level genome assembly of the colonial chordate model Botryllus schlosseri (Tunicata)

Version: Original Submission **Date:** 4/2/2025

Reviewer name: Cristian Canestro

Reviewer Comments to Author:

TO THE AUTHORSIn this MS entitled 'First chromosome-level genome assembly of the colonial chordate model Botryllus schlosseri (Tunicata)', Olivier De Thier and colleagues report the first chromosome-scale assembly of this colonial ascidian specie, paying special attention to differences with previous published assemblies and importantly between haplotypes. The MS is very well written, very easy and pleasant to read. This provides data of great quality and very relevant not only for the ascidian/tunicate community, but to the field of genome structural evolution. I firmly recommend it for publication, although I think that the authors could discuss it in deeper detail. Specially, I miss for instance a more elaborate discussion of the results in our understanding of the similarities and differences between clades that have been published in the last years (I have not been able to find some relevant articles in this regard cited in the bibliography). I also feel that a deeper analysis of the differences between haplotypes could be very interesting, unless they are artifactual effects of the assemblies. As mentioned below, unless this is part of a longer story for a different MS beyond the scope of this one, I encourage the authors to validate some of the differences they find between haplotypes, and try to correlate the structural variations, with differences in gene counts between haplotypes, and to explore whether these differences could be correlated with aspects of biological relevance. I miss, for instance, Venn diagrams with gene contents between previous assemblies, and the haplotypes/haploid genome here reported. In any case, I firmly recommend this MS for publications, since most of my suggestions are not intended to interrogate the results of the MS, but to improve it, but I also understand that some may go beyond the scope of this MS.Minor points:IntroductionPage 1: "the basic body plan of adult tunicates is highly conserved across the entire subphylum [3]". This sentence, which could be OK for ascidians, probably provides a highly simplified vision of Tunicate adult morphologies, specially comparing the divergent morphologies of Thaliaceans and Appendicularians. Please, elaborate the sentence.To understand the comparisons between the data of this MS and previously reported genomes, it seems crucial to understand well the meaning of the "clades and subclades". Please, include in the introduction (or where needed), how are defined those clades, which are their origins and biological/geographical differences, … and all the critical information that will specially help non-tunicate readers to understand the results.Results:The authors refer to the presence of large-scale genomic palindromes in Bs1 and Bs3. But it is unclear what are these structures. I suggest to please provide some more detailed explanation about the palindromic nature of these regions.The data of haplotype-resolved assemblies is very interesting. I wonder if it is possible to somehow measure the amount of heterozygosity between haplotype 1 and 2, and those versus the previous versions of the genome, to better understand intra and inter-variation between subclades?The differences of the size of some regions between Colombera

and this study, and even between haplotypes 1 and 2, are very interesting. I would find more informative to merge the three graphs of Figure S9 into one single graph, so we can also easily compare the different in sizes of the haplotypes with the haploid. If some of those differences are actually due to deletions, that would deserve further analysis. If this analysis is not part of another ongoing project that will be published somewhere else, I suggest identifying with a dot-plot some of those differences, specially between haplotypes, and validate with long-reads crossing those regions whether some of the deletions are real or artifactual. Please, include the dotplot graph together with the two haplotypes in figure S10. In those cases that could be real, it would be very interesting what genes are gone, and if those are not placed somewhere else in the genome as result of translocations, or those genes are actually gone and could explain some of the differences reported in the gen count between haplotypes.The authors mentioned the presence of multiple structural variations, although some of which could be artifactual of miss-assemblies. Interestingly, the plot of the synteny blocks between the two haplotypes in figure S11 shows some of those structural variations, including cases of:- deletions: for instance, there are "blank" regions in Bs1A and Bs3A with no lines, which may reflect areas that are not present in the haplotype B.- duplications and translocations within chromosomes or between chromosomes of different haplotypes.Just looking to this plot, I wonder how the distribution of chromosomes between haplotypes is done. For instance, I see that Bs7B shares a duplicated synteny block with chromosomes Bs10B and Bs14B, but not with Bs10A and Bs10B, which means that the duplications are intra-haplotype present in B but not in A. But I wonder if it is possible that Bs10B and Bs14B could be in fact switched to haplotype A, and therefore there would be no duplication nor deletion in one of the haplotypes, just a simple translocation. I may be wrong in the interpretation, but I'm curious to understand the graph.In any case, again, as mentioned above, it would be worthy to validate some of those variations with long reads, which could illuminate the biological relevance between the haplotypes and discard potential artifactual errors of the assemblies.I notice that in figures 7 and S13, some lines are thicker than others. Is this because many "thin" lines are overlapped, and they look like a "thick" line. Otherwise, the visual effect of different thicknesses could be misleading. Please, clarify.In the analysis of the Hox cluster the authors say "[…] our new assembly revealed that B. schlosseri's Hox genes are not scattered. Instead, eight of them were clustered on the second largest scaffold (Bs2), whereas two other ones are found on the 15th largest scaffold (Bs15)." Generally, the description of the Hox gene in a cluster refers to the fact they are in the vicinity, with near not many other genes in between Hox genes. Therefore, I would not describe that eight Hox genes are clustered by the simple fact that they are in the same chromosome (maybe even in different arms).

Level of Interest

Please indicate how interesting you found the manuscript: Choose an item.

Quality of Written English

Please indicate the quality of language in the manuscript: Choose an item.

Declaration of Competing Interests

Please complete a declaration of competing interests, considering the following questions:

- Have you in the past five years received reimbursements, fees, funding, or salary from an organisation that may in any way gain or lose financially from the publication of this manuscript, either now or in the future?
- Do you hold any stocks or shares in an organisation that may in any way gain or lose financially from the publication of this manuscript, either now or in the future?
- Do you hold or are you currently applying for any patents relating to the content of the manuscript?
- Have you received reimbursements, fees, funding, or salary from an organization that holds or has applied for patents relating to the content of the manuscript?
- Do you have any other financial competing interests?
- Do you have any non-financial competing interests in relation to this paper?

If you can answer no to all of the above, write 'I declare that I have no competing interests' below. If your reply is yes to any, please give details below.

I declare that I have no competing interests

I agree to the open peer review policy of the journal. I understand that my name will be included on my report to the authors and, if the manuscript is accepted for publication, my named report including any attachments I upload will be posted on the website along with the authors' responses. I agree for my report to be made available under an Open Access Creative Commons CC-BY license (http://creativecommons.org/licenses/by/4.0/). I understand that any comments which I do not wish to be included in my named report can be included as confidential comments to the editors, which will not be published.

Choose an item.

To further support our reviewers, we have joined with Publons, where you can gain additional credit to further highlight your hard work (see: https://publons.com/journal/530/gigascience). On publication of this paper, your review will be automatically added to Publons, you can then choose whether or not to claim your Publons credit. I understand this statement.

Yes Choose an item.
